# Local structure order parameters and site fingerprints for quantification of coordination environment and crystal structure similarity†

**DOI:** 10.1039/c9ra07755c

**Published:** 2020-02-07

**Authors:** Nils E. R. Zimmermann, Anubhav Jain

**Affiliations:** Energy Technology Area, Lawrence Berkeley National Laboratory Berkeley California 94720 USA nils.e.r.zimmermann@gmail.com +49 177 9077 532; Energy Technology Area, Lawrence Berkeley National Laboratory Berkeley California 94720 USA

## Abstract

Structure characterization and classification is frequently based on local environment information of all or selected atomic sites in the crystal structure. Therefore, reliable and robust procedures to find coordinated neighbors and to evaluate the resulting coordination pattern (*e.g.*, tetrahedral, square planar) are critically important for both traditional and machine learning approaches that aim to exploit site or structure information for predicting materials properties. Here, we introduce new local structure order parameters (LoStOPs) that are specifically designed to rapidly detect highly symmetric local coordination environments (*e.g.*, Platonic solids such as a tetrahedron or an octahedron) as well as less symmetric ones (*e.g.*, Johnson solids such as a square pyramid). Furthermore, we introduce a Monte Carlo optimization approach to ensure that the different LoStOPs are comparable with each other. We then apply the new local environment descriptors to define site and structure fingerprints and to measure similarity between 61 known coordination environments and 40 commonly studied crystal structures, respectively. After extensive testing and optimization, we determine the most accurate structure similarity assessment procedure to compute all 2.45 billion structure similarities between each pair of the ≈70 000 materials that are currently present in the Materials Project database.

## Abstract 2

Structure characterization and classification is frequently based on local environment information of all or selected atomic sites in the crystal structure.

## Graphical abstract

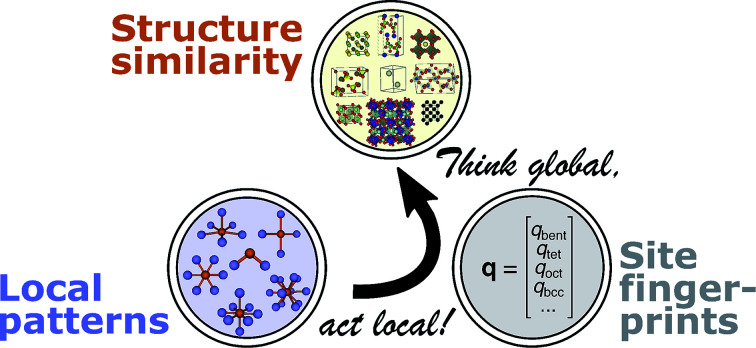

## 1 Introduction

**Fig. 1:**
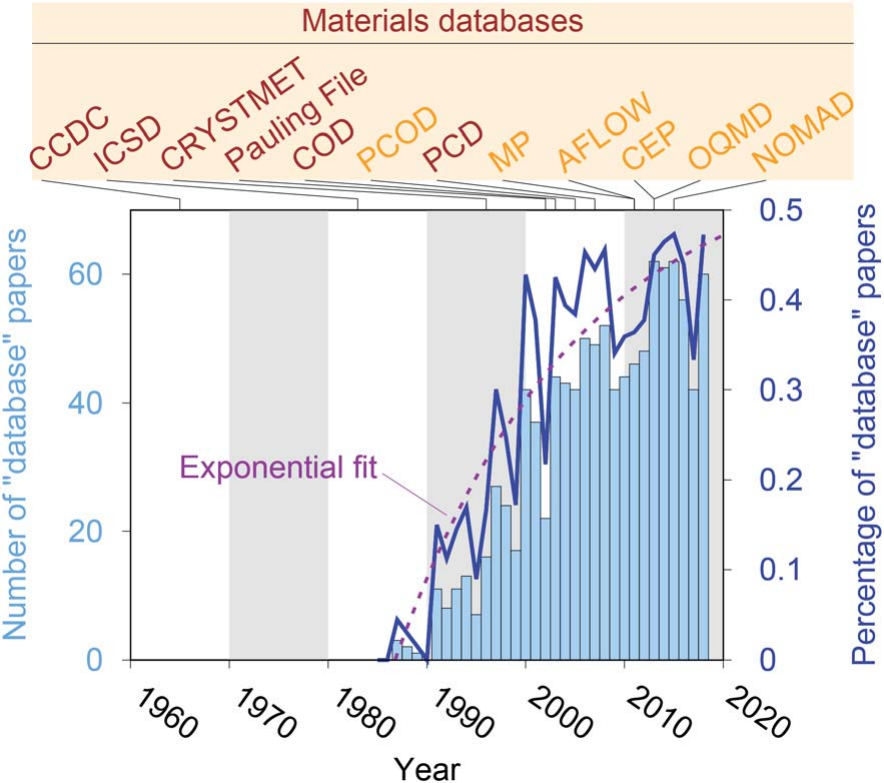
Publication statistics for research articles that include “database” as keyword in the topic field within the Web of Science™ Core Collection for category “Chemistry, Inorganic & Nuclear.” Data was retrieved on November 23, 2019. Furthermore, we highlight the inception years of established materials databases.

Crystal structure databases^1–16^ play an increasingly important role in materials science, chemistry, and related fields. Publication statistics gathered from Web of Science^17^ on November 23, 2019, indicate that this trend started in the early 1990s (Fig. 1) and that the underlying potential is still not exhausted. The steady increase is (most likely) linked to continuously increasing computing power and memory storage, and it has fostered the creation of many different crystallographic databases that catalog existing materials such as the Cambridge Crystallographic Data Centre (CCDC) in 1965,^1^ the Inorganic Crystal Structure Database (ICSD) in 1983,^2,3^ CRYSTMET in 1993,^4,5^ Pauling File in 2002,^6,7^ the Crystallography Open Database (COD) in 2003^8^ (previously known as “The *American Mineralogist* crystal structure database”^9^), and the Pearson's Crystal Data (PCD) in 2007.^10^ Furthermore, computational databases, which mainly use crystallographic databases as a source, and fully hypothetical structure databases are currently being created more and more: the Predicted Crystallography Open Database (PCOD) appeared in 2005, the Materials Project (MP) database^11,12^ and the Automatic FLOW for Materials Discovery database (AFLOW) in 2011,^13^ the Harvard Clean Energy Project (CEP)^14^ and the Open Quantum Materials Database (OQMD) in 2013,^15^ as well as the Novel Materials Discovery Laboratory (NOMAD) in 2015.^16^

As computational resources still continue to grow^18^ and to become more omnipresent and accessible, the computational chemistry, physics, and materials science communities have focused their efforts more and more on automation tools for materials database analysis and on employing statistical and machine learning (SML)^19,20^ to help expedite materials discoveries and chemical innovations.^21^ This includes, for example, predicting properties (*e.g.*, formation energies, crystal structure dimensionalities, phase diagrams, band gaps, elastic moduli, ionic conductivity) of diverse materials from classes and families such as AX binary compounds,^22^ M_2_AX ternary phases,^23^ delafossite and related layered phases of composition ABX_2_,^24^ conventional^25^ and double perovskite halides (or elpasolites),^26^ zeolites^27,28^ and other silicates,^29^ and other inorganic materials^30–36^ as well as polymers;^37^ indicating possible synthesis approaches by screening and predicting synthesis parameters and reactions of inorganic materials,^38,39^ metal–organic frameworks,^40^ and organic molecules;^41,42^ generating interatomic potentials;^43–46^ and expediting *ab initio*^47–50^ calculations.^51–55^

**Fig. 2:**
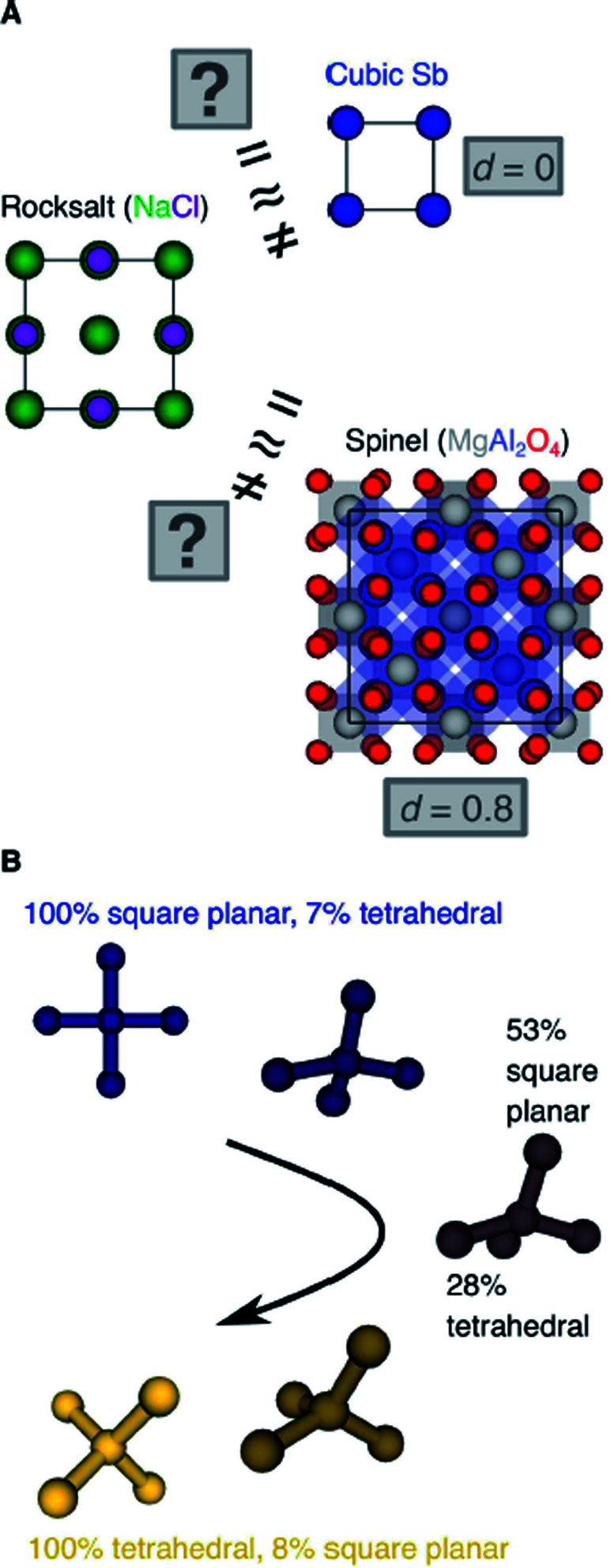
(A) Is rocksalt structurally (more) similar to cubic Sb or to MgAl_2_O_4_-spinel? (B) Can we create descriptors which measure the degrees of resembling perfect coordination environments in a comparable way?

Another important scientific problem is, in this context, the classification and categorization of entire crystal structures and the assessment of similarity between two materials (Fig. 2A).^56–58^ Conventionally, crystal structures are characterized by their chemistry, crystal system, and space group. Other schemes employ the coordination number and pattern of the constituting atomic sites.^59^ Because of the plethora of ways to classify structures, defining and automatically finding prototype structures is currently a very active research area.^60–62^ In particular, the usage of coordination number and pattern has culminated in a larger current effort of the community to leverage fingerprinting.^58,63–66^ This is the process of combining crucial information about the structure and/or its constituting local environments around each atom into a vector that represents the structure as a whole and includes, for example: a two-dimensional fingerprint based on simulated diffraction patterns;^57^ the Coulomb matrix;^67^ a many-body tensor representation;^68^ deep tensor neural networks;^45^ Voronoi tessellation;^69^ radial distribution functions with^70^ and without^71^ incorporating partial atomic charges; and local environment-based crystal fingerprints.^72^

Crystal structure classification approaches that are specifically based on coordination information of the constituent atomic sites have the advantage that, apart from globally classifying the structure, they carry easily interpretable local information. This facilitates to ensure causality between the descriptor–property relation and the underlying mechanism,^73^ when using the corresponding local coordination descriptors for design rules or machine learning applications. A critically important ingredient is then the effectiveness of and comparability across coordination site descriptors (Fig. 2B) and the resulting site fingerprints that characterize the coordination environment.

To address this we introduce here (i) a new neighbor finding method, (ii) several new local structure order parameters (LoStOPs), which are optimized with a tailor-made Monte Carlo procedure, as well as (iii) new site fingerprint and (iv) new structure fingerprints (Section 2), which are freely available through the python^74^ packages pymatgen^75,76^ and matminer.^65,77^ We extensively test and optimize the new tools on known coordination environments and commonly investigated crystalline prototype materials with the main purpose of assessing site and structure similarity (Section 3).

## 2 Methodology

In this section, we introduce our methods for finding neighbors of a given central site in a crystal structure,^78–80^ for performing pattern matching on the resulting coordination environment,^81,82^ and for using that information to generate fingerprints that aim to characterize coordination environments and, ultimately, entire crystal structures on the basis of the coordination descriptors. Furthermore, we optimize our novel coordination environment descriptors [local structural order parameters (LoStOPs)] to improve inter-motif comparability,^83^ and we describe the similarity measures that we test for comparing site and structure fingerprints. All of the methods and algorithms listed here are freely available through the local_env module in pymatgen^75,76^ and the featurizers module in matminer.^65,77^

### 2.1 Neighbor finding approaches

Two general and very popular neighbor finding approaches are employed in this work, which use (1) distance^80^ and (2) topology-based,^78,79^ information, respectively, to decide which neighbors are included in the near neighbor list. In both cases, an initial tentative neighbor list is constructed with a large hard cutoff radius (typically: 7–10 ångström), which can, however, be dynamically increased, as we explain below. The methods are therefore approaches how to prune this initial (long) neighbor list.

**Fig. 3:**
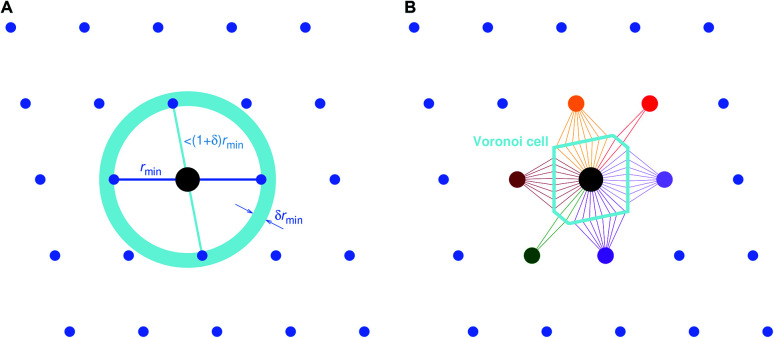
Basic neighbor finding approaches used and investigated in this work: (A) minimum distance^80^ and (B) Voronoi decomposition-based^78,79^ near neighbor finding.

The first approach, which we call “minimum distance” neighbor finding (MDNF), consists of 3 basic steps (Fig. 3A):

• Find neighbor *k* that has the smallest distance, *d*_min_ = *d*_*k*_ = min({*d*_*j*_}), to (central) site *i*, given all distances, {*d*_*j*_}, from the tentative neighbor list.

• Divide all distances, {*d*_*j*_}, by *d*_min_, thus, yielding fractional distances, {*d̃*_*j*_} = {*d*_*j*_/*d*_min_}.

• Include neighbors that are at most 1.1 fractional distances away from the central site^80^ (*i.e.*, *d̃* ≤ 1.1).

We choose the label “minimum distance” over “relative distance” for this neighbor finding approach in order to avoid confusion with similar methods^80^ that use bond lengths^84^ and/or atomic/ionic radii^85^ to compute dimensionless (relative) neighbor distances.

The second approach (VNF) uses Voronoi decomposition^78,79^ to identify neighbors from the tentative list by employing the solid angle as a weighting measure.^86^ In this case, we search for the largest solid angle among all tentative neighbors, divide all solid angles by this maximum angle, and use a threshold (typically, 0.05) to cut out all neighbors that have a fractional solid angle that is smaller than the threshold.

**Fig. 4:**
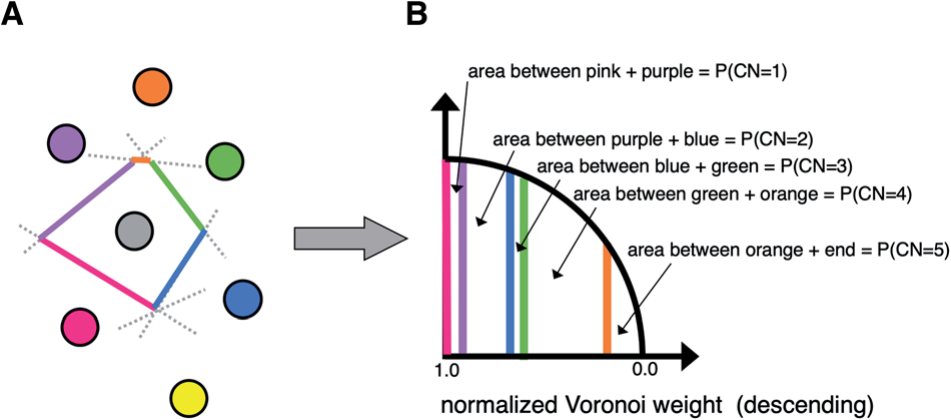
Schematic of the novel neighbor-finding approach, “CrystalNN”. (A) An example neighbor environment; the Voronoi weights of each neighboring site can be considered proportional to the length of its corresponding colored lined segment. (B) Demonstrating the calculation of coordination probabilities from the normalized Voronoi weights. For example, the section of the semicircle between the pink and purple segments indicates the small probability that only the strongest neighbor (pink) should be considered a neighbor. The area between the purple and blue segment indicates the larger probability that the system should be 2-coordinated (the two strongest neighbors, pink and purple). The highest coordination probability for this particular system would be 4-fold coordinated, corresponding to pink, purple, blue and green sites being neighbors.

A third neighbor-finding scheme, which we label “CrystalNN” (CNN), assigns probabilities to multiple coordination environments (Fig. 4). The algorithm begins similarly to the Voronoi strategy in which each neighbor is assigned a weight based on solid angle and facet area. Next, all weights are normalized such that the maximum weight is 1. All distinct values of normalized weight (multiple neighbor sites can belong to the same weight) are arranged from 0 to 1. For each distinct weight, the probability of all sites with that weight or greater being considered neighbors is proportional to the integral of the area under a curve (in our case, a semicircle that has a value of zero at zero weight and a value of 1 at a weight of 1) starting from 0 and ending in that weight. Thus, neighbors with higher weights are given a larger “section of the pie” in terms of likelihood to be part of the coordination environment. Also note that this method computes coordination likelihoods, *w*_CN=*i*_, on the basis of these neighbor coordination probabilities, which quantify the probability that a given coordination environment should be considered *i*-fold coordinated. In an upcoming paper, we investigate the performance of various existing methods^79,80,84,85,87,88^ and CNN to predict coordination numbers in inorganic materials, where our novel method performs particularly well.

### 2.2 Local structure order parameters

We use local structure order parameters (LoStOPs) such as those introduced by Peters^81^ (*q*_bcc_) and by Zimmermann *et al.*^80,82^ (*q*_tet_ and *q*_oct_) to determine the degree to which the angles in a given observed coordination environment agree with those in the perfect target environment. The LoStOPs are being increasingly exploited for rating the feasibility^89,90^ of zeolites,^27,28^ finding interstitials and evaluating diffusional pathways in materials,^80^ hierarchically visualizing structural similarity,^91^ and predicting the magnetic ordering of inorganic materials.^92^

In this work, we vastly extend the existing library from 3 to 20 by introducing new LoStOPs (Table 1). The new LoStOPs permit the detection of both highly symmetric motifs (*e.g.*, Platonic solids^93^ such as a tetrahedron or an octahedron) as well as less symmetric motifs (*e.g.*, Johnson solids^94^ such as a square pyramid). This is possible because we change our ansatz slightly while keeping the key ideas.

**Table 1:** New and existing local structure order parameters (LoStOPs)

Coordination environment (CE)	LoStOP	Coordination number (CN)	Reference
Single bond	*q*_sgl_bd_	1	This work
Bent bonds	*q*_bent_	2	This work
Trigonal planar	*q*_tri_plan_	3	This work
T-shape	*q*_T_	3	This work
Square planar	*q*_sq_plan_	4	This work
Square non-coplanar	*q*_sq_	4	This work
Tetrahedral	*q*_tet_	4	82
See–saw^a^	*q*_see_saw_rect_	4	This work
Trigonal pyramid	*q*_tri_pyr_	4	This work
Pentagonal planar	*q*_pent_plan_	5	This work
Square pyramid	*q*_sq_pyr_	5	This work
Trigonal bipyramid	*q*_tri_bipyr_	5	This work
Hexagonal planar	*q*_hex_plan_	6	This work
Pentagonal pyramid	*q*_pent_pyr_	6	This work
Octahedral	*q*_oct_	6	82
Hexagonal pyramid	*q*_hex_pyr_	7	This work
Pentagonal bipyramid	*q*_pent_bipyr_	7	This work
Hexagonal bipyramid	*q*_hex_bipyr_	8	This work
BCC	*q*_bcc_	8	81
Cuboctahedron	*q*_cuboct_	12	This work

a The target see–saw motif has a 90° angle instead of the typical 120° as explained in the text.

**Fig. 5:**
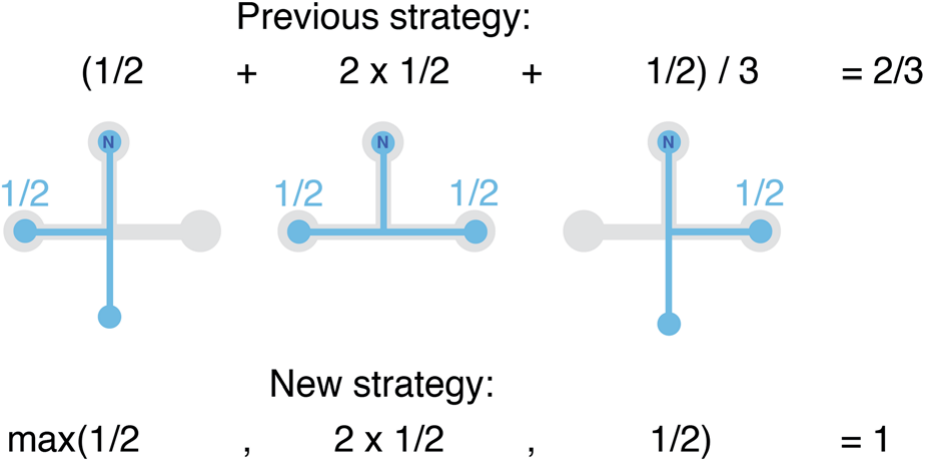
Visual depiction of change in the computation of local structure order parameters: going from the previous strategy of averaging (top) to the new strategy of finding the maximal resemblance, given all possible rotations.

We still test whether a given coordination environment (*e.g.*, the blue T-motif in Fig. 5) resembles a perfect target motif (gray “template” in Fig. 5) by using the neighbors of the central site to locally set up spherical coordinate systems.^81,82^ And, we still check all permutations explicitly; that is, we use each neighbor as a tentative North pole for creating the coordinate system (marked with a dark-blue “N” in each configuration) and each remaining neighbor as a prime meridian. We also stay with the general strategy of using cosine functions and Gaussian kernels [without normalization constant 
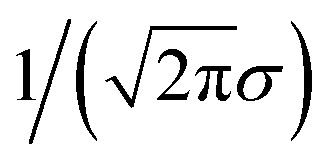
] to penalize positions of the remaining neighbors that are not at expected positions. The crucial difference to our earlier motif resemblance metrics^80–82^ is that we do not average over all permutations anymore. Instead, we use the highest motif resemblance,

1 *q*_*m*_ = max({*q*_*m*,*j*_})

given all the individual resemblance values, *q*_*m*,*j*_, each of which is obtained with one single neighbor *j* as the North pole for resemblance evaluation to motif type *m* around central site *i* (Fig. 5). For example, the LoStOP for the T-shaped coordination environment, *q*_T_, is given by:2
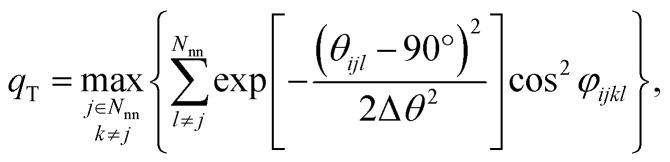
where *N*_nn_ is the number of those near neighbors that are to be considered for LoStOP calculation, *θ*_*ijl*_ is the polar angle of neighbor *l* (*i.e.*, the angle between North pole neighbor *j*, central site *i*, and neighbor *l*), Δ*θ* is the parameter penalizing positions of *l* that are not forming a perfect 90° angle with the bond between *i* and *j*, and neighbor *k* is used to construct the prime meridian; hence, *φ*_*ijkl*_ is zero for *l* = *k*. Note that we also redefine the tetrahedral and octahedral LoStOPs using the new ansatz regarding the permutations.

**Fig. 6:**
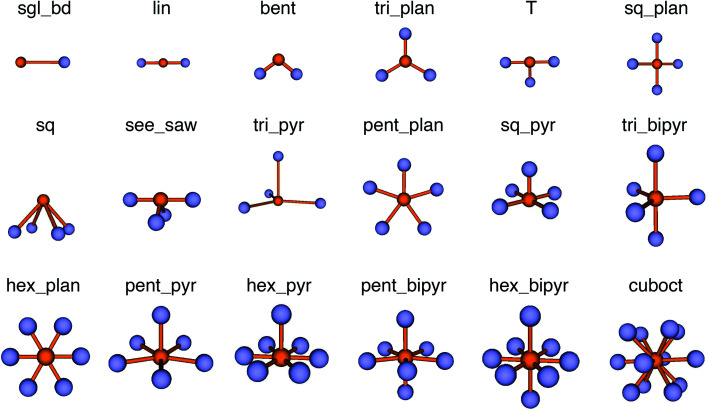
Coordination motifs for which we introduce new local structure order parameters (LoStOPs) along with their labels that we use in this work.

The new LoStOPs allow detection of even more motifs than suggested by Table 1 (*cf.*, Fig. 6) because the linear/bent LoStOP can be used for various bent angles, the trigonal bipyramid LoStOP can be used for regular see–saw motifs, and we can use *q*_tet_ to detect triangular non-coplanar arrangements with tetrahedral angles. Later, when we define site fingerprints, we define multiple instances of the bent LoStOP with different target angles. Note that the new LoStOPs, just as the originals ones, are invariant to translational and rotational operations, which represents an important prerequisite to be used as an element of a numerical materials fingerprint.^63^ Furthermore, the new LoStOPs still smoothly vary between 0 and 1, and a value of 1 flags, as usual, perfect resemblance with the underlying target motif, whereas 0 indicates no resemblance or match.

#### 2.2.1 Optimization

Any LoStOP computation requires the *a priori* choice of certain calculation parameters such as the penalty for the neighbor that is closest to the South pole position in an octahedron-like environment to not exactly form a straight line with the (tentative) North pole neighbor and the central site. Our goal for setting these parameters was to maximize the comparability between different motifs (*e.g.*, degree of resembling a perfect tetrahedron equals degree of a perfect square pyramid). Consequently, we had to make a decision about which distortion in one motif (*e.g.*, moving one of the non-North pole neighbors in a tetrahedron slightly towards the South pole position) should be compared to another distortion in a different motif (*e.g.*, moving two neighbors in the basal plane of the square pyramid closer together). Identifying all possible elemental distortions would have been unfeasible, notwithstanding intermediate distortion pathways (*cf.*, Fig. 4 in ref. 80). Also, it would have been somewhat arbitrary to weight the different elemental and intermediate distortions for their occurence or relevance: should they contribute equally or are some less likely or less important than others?

To circumvent these issues we decided to employ a numerical procedure that leverages our recently introduced Einstein crystal test rig procedure.^80^ This order parameter testing framework assigns Gaussian-distributed random perturbations to all sites in the initially perfect coordination environment using the polar form of Box–Muller transforms^95^ as implemented in numpy.^96^ The resulting spatial distributions of the atoms around their perfect motif positions resemble those in an Einstein crystal or molecule.^97–99^ The atom displacement distribution width, *σ*_EM_, is an input parameter and provides a well-controllable way to *a priori* define the average distortion degree of the entire coordination environment. Thus, it also provides a definition for comparing distortion states between different motifs. Furthermore, because we aim at the assessment of real materials and their coordination patterns and because those can be subject to thermal fluctuations, we, hence, use a physically meaningful reference model.

**Fig. 7:**
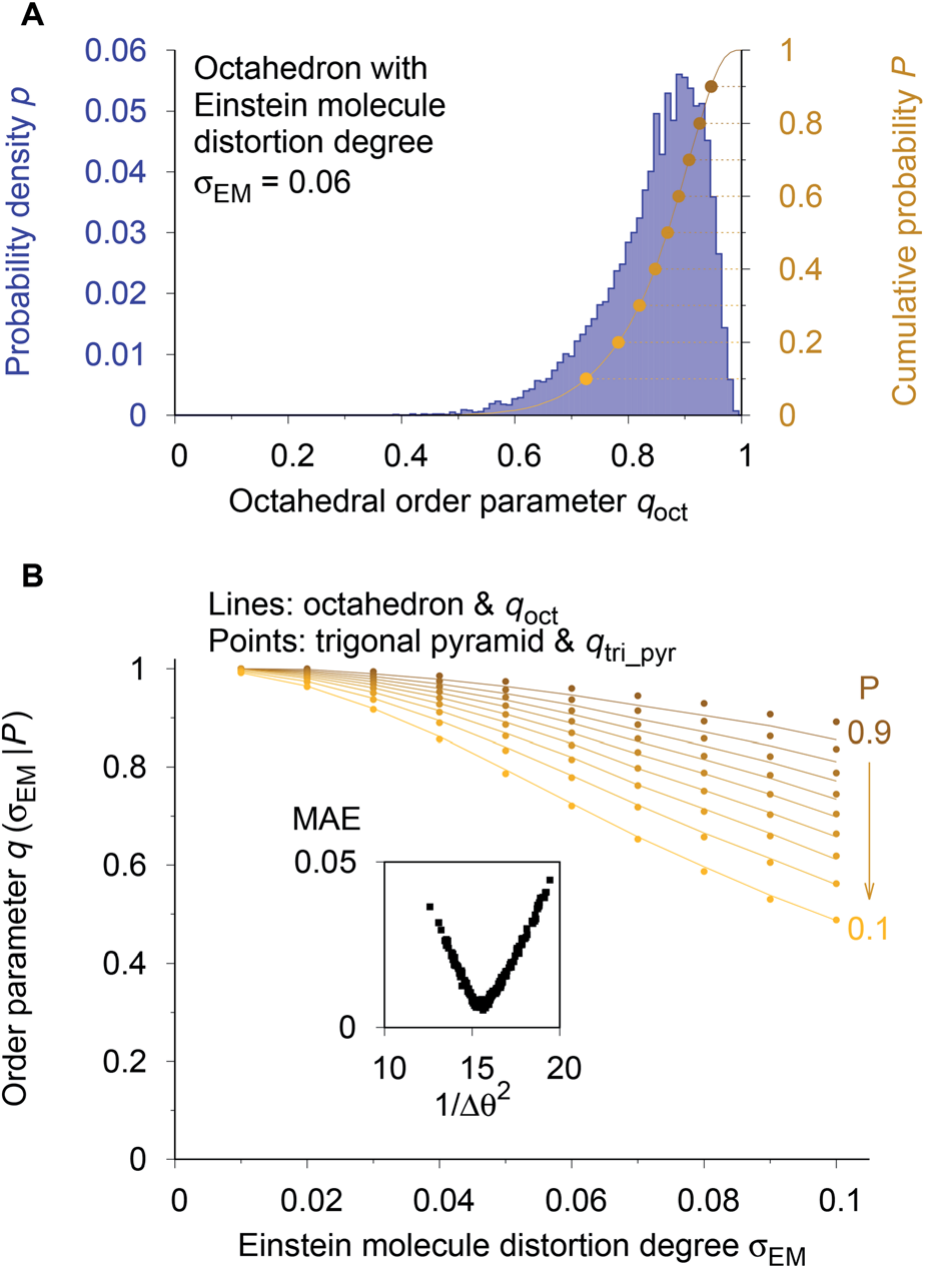
Procedure for optimizing the calculation parameters to maximize inter-motif comparability. (A) The cumulative probability distributions of the octahedral LoStOP as resulting from random displacements (Einstein crystal test rig; *cf.*, ref. 80) are calculated for different Einstein molecule displacement degrees, *σ*_EM_, to give the baselines. (B) The parameters involved in the calculation of any and all other LoStOPs (here: trigonal pyramid) are optimized by minimizing the difference of curves obtained from plotting the LoStOP given a specific cumulative probability value, *q*(*σ*_EM_|*P*), as it varies with Einstein molecule displacement degree, *σ*_EM_. The inset shows the mean absolute error, MAE, *vs.* the (inverse and squared) parameter variation, 1/Δ*θ*^2^ for the case of *q*_tri_pyr_.

As a reference point, we use the Einstein molecule response behavior of the octahedral order parameter, *q*_oct_. For varying Einstein molecule distortion degrees (*σ*_EM_ = 0.01⋯0.1 in 0.01 increments), we compute histograms of the order parameter by perturbing all atoms in an octahedral coordination motif 10 000 times. From each density distribution *p*(*q*_oct_|*σ*_EM_) (blue line in Fig. 7A), we compute cumulative probability distributions *P*(*q*_oct_|*σ*_EM_) (gold-brown line and points), which are smooth functions of *q*_oct_. To reduce the number of data points we consider only 9 values of the cumulative distributions in the following: the points at *P* = 0.1, 0.2, 0.3, …, 0.9. Below, we use and refer to these points in an inverse manner: the order parameter values as a function of the Einstein molecule distortion degree for a given cumulative probability value, *q*(*σ*_EM_|*P*).

Using the octahedral order parameter results as reference data, our optimization procedure for any remaining order parameter (*e.g.*, trigonal pyramid, *q*_tri_pyr_) is a basic Monte Carlo approach and consists of following steps:

(1) Create a set of new trial calculation parameters (*e.g.*, for *q*_tri_pyr_, the penalty, Δ*θ*, for polar angles being different than their expected values of 90°) by randomly perturbing the current parameters within a pre-defined maximum range.

(2) Compute the *q*(*σ*_EM_|*P*) data (in Fig. 7B: gold-brown points) with the motif for which the OP is designed (here: *q*_tri_pyr_ and a trigonal pyramid).

(3) Calculate the mean absolute error (MAE) between these data points and the octahedral reference at the same Einstein molecule distortion degrees (*i.e.*, sum up the absolute difference between points and lines in Fig. 7B).

(4) Compare the new MAE, Δ_new_, to the MAE resulting from the previous parameter set, Δ_old_. If exp[−*k*_MC_(Δ_new_ − Δ_old_)] is larger than a random number drawn from a uniform distribution, then save the new trial parameter set; else, continue to use the previous parameter set.

(5) Go to step 1.

We usually perform 2000 MC trials and, in each separate trial, we compute the *q*_*i*_(*σ*_EM_|*P*) data by 1000 Einstein molecule perturbations. We carry out at least two optimization runs. In the first optimization, we used a small MC parameter, *k*_MC_ = 100, and a larger maximum parameter perturbation range, which allowed sampling of a larger parameter space. The second run proceeded with a larger MC parameter and a smaller maximum perturbation range, which forced the system to quickly go to the (nearest) MAE minimum. The first optimization served as a check whether or not there is another minimum that might even have a lower MAE than the one that is closest to the initial state during optimization. Finally, the inset of Fig. 7B indicates that the procedure is appropriate because we obtain a convex parameter space.

### 2.3 Site fingerprint

We define site fingerprints on the basis of the above described coordination likelihood and local structure order parameters. The site fingerprints are single-column vectors, the elements of which exhibit an ordered structure. Specifically, the different coordination features are arranged in blocks that represent well-defined coordination numbers so that a site fingerprint **v** can be written as:

3 **v** = [**v**_1_, **v**_2_, …, **v**_12_]^T^,

where **v**_*i*_ denote the (sub)fingerprint that contains all coordination features fulfilling the condition that the coordination number is *i*. For example, **v**_4_ can carry the likelihood of being 4-fold coordinated, the square planar LoStOP, and the tetrahedral LoStOP, but it cannot carry the likelihood of being 3-fold coordinated or the bcc (8-fold coordinated) LoStOP. While it might be necessary to increase the length to also include **v**_13_ and **v**_14_, our tests have shown that there is little benefit of doing so because the vast majority of sites in crystal structures typically have a coordination number ≤12. In the following, we describe specific details of three site fingerprint (CrystalNNFingerprint, OPSiteFingerprint, and ChemEnvFingerprint) as they are currently implemented in matminer^65,77^ (version: 0.3.3). Note that there is a mutual strategy in computing the first two fingerprints: grouping the features according to their underlying coordination number, as outlined in eqn (3).

The CrystalNNFingerprint (CNN fingerprint) computes the fingerprint of a given site *i* as follows. First, we choose the coordination features to be included (*e.g.*, *w*_CN=4_, *q*_sq_plan_, and *q*_tet_). Second, the neighbors of site *i* are determined with the CrystalNN neighbor-finding algorithm. Then, we loop over all theoretically possible coordination numbers between 1 and the maximum of the set of coordination numbers that underly all chosen features from step 1. For each coordination number *j*, we compute the coordination likelihood, *w*_CN=*j*_. Subsequently, we compute each LoStOP (*e.g.*, *q*_tet_) that is to be considered for this coordination number (here: CN = 4) using *j* neighbors (here: 4) with the highest coordination weight, we multiply the value with *w*_CN=*j*_ (here: *q*_tet_ × *w*_CN=4_), and we then add the resulting value to the (growing) site fingerprint vector. If there is no feature type for a given coordination number *j*, a single zero entry is added for the entire *j*-block. Furthermore, note that the implementation of the CNN site fingerprint in matminer^65,77^ features two convenient presets for rapid calculation setup: the “cn” preset only computes coordination likelihoods (*w*_CN=*j*_), whereas the “ops” preset adds all available LoStOP features in addition to the *w*_*j*_'s.

Similar to the CNN site fingerprint, the OPSiteFingerprint (OPS fingerprint) first requires the choice of the features to be included. The main differences to the CNN fingerprint are:

(1) The minimum distance neighbor finding method is used with the default fractional cutoff (1.1).

(2) A more elaborate binning scheme is used to determine whether neighbors belong to the same shell or not, which basically employs a bin width variation approach.

(3) Based on the variational neighbor finding results, multiple values for a given LoStOP are obtained, from which the most stable (*i.e.*, most frequently occurring) value is extracted *via* histogramming.

(4) Coordination weights (*w*_*j*_) are not used (*i.e.*, the fingerprint fully relies on the LoStOP features). However, an additional distance variation factor, *f*_d_, can be chosen which is, for a motif with *N*_nn_ neighbors around central site *i* given by:4
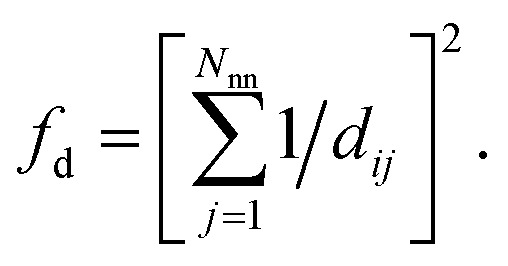


Lastly, the ChemEnvFingerprint (CE fingerprint) makes full use of the ChemEnv module in pymatgen,^75,76^ which provides alternative tools for automatically identifying the coordination environments of atoms in materials.^100^ Two presets are available (“simple” and “multi_weights”), which directly relate to the corresponding ChemEnv strategies. The principal neighbor finding approach is Voronoi decomposition (VNN). The features that are computed with the CE fingerprint are the continuous symmetry measures^101^ (CSM) between a given motif and all available ideal coordination environments supported by ChemEnv.^100^ In particular, the considered environments are: (1) single neighbor (S:1), (2) linear (L:2), (3) angular (A:2), (4) trigonal plane (TL:3), (5) triangular non-coplanar (TY:3), (6) T-shape (TS:3), (7) tetrahedron (T:4), (8) square plane (S:4), (9) square non-coplanar (SY:4), (10) see–saw (SS:4), (11) pentagonal plane (PP:5), (12) square pyramid (S:5), (13) trigonal bipyramid (T:5), (14) octahedron (O:6), (15) trigonal prism (T:6), (16) pentagonal pyramid (PP:6), (17) pentagonal bipyramid (PB:7), (18) square-face capped trigonal prism (ST:7), (19) end-trigonal-face capped trigonal prism (ET:7), (20) face-capped octahedron (FO:7), (21) cube (C:8), (22) square antiprism (SA:8), (23) square-face bicapped trigonal prism (SBT:8), (24) triangular-face bicapped trigonal prism (TBT:8), (25) dodecahedron with triangular faces (DD:8), (26) dodecahedron with triangular faces—p2345 plane normalized (DDPN:8), (27) hexagonal bipyramid (HB:8), (28) bicapped octahedron (opposed cap faces) (BO_1:8), (29) bicapped octahedron (cap faces with one atom in common) (BO_2:8), (30) bicapped octahedron (cap faces with one edge in common) (BO_3:8), (31) triangular cupola (TC:9), (32) tricapped triangular prism (three square-face caps) (TT_1:9), (33) tricapped triangular prism (two square-face caps and one triangular-face cap) (TT_2:9), (34) tricapped triangular prism (one square-face cap and two triangular-face caps) (TT_3:9), (35) heptagonal dipyramid (HD:9), (36) tridiminished icosahedron (TI:9), (37) square-face monocapped antiprism (SMA:9), (38) square-face capped square prism (SS:9), (39) tricapped octahedron (all 3 cap faces share one atom) (TO_1:9), (40) tricapped octahedron (cap faces are aligned) (TO_2:9), (41) tricapped octahedron (all 3 cap faces are sharing one edge of a face) (TO_3:9), (42) pentagonal prism (PP:10), (43) pentagonal antiprism (PA:10), (44) square-face bicapped square antiprism (SBSA:10), (45) metabidiminished icosahedron (MI:10), (46) bicapped square prism (opposite faces) (BS_1:10), (47) bicapped square prism (adjacent faces) (BS_2:10), (48) trigonal-face bicapped square antiprism (TBSA:10), (49) pentagonal-face capped pentagonal antiprism (PCPA:11), (50) hendecahedron (H:11), (51) sphenoid hendecahedron (SH:11), (52) diminished icosahedron (DI:11), (53) icosahedron (I:12), (54) pentagonal-face bicapped pentagonal prism (PBP:12) (55) truncated tetrahedron (TT:12), (56) cuboctahedron (C:12), (57) anticuboctahedron (AC:12), (58) square cupola (SC:12), (59) hexagonal prism (HP:12), (60) hexagonal antiprism (HA:12), and (61) square-face capped hexagonal prism (SH:13).

### 2.4 Structure fingerprint

**Fig. 8:**
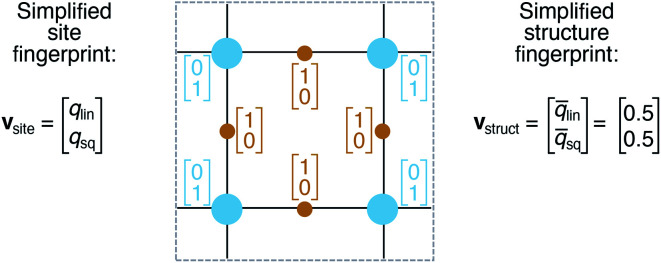
Illustration of site and structure fingerprints.

On the basis of the site fingerprints of all atoms in a crystal structure we can compute meaningful structure fingerprints (Fig. 8). Our approach consists of four steps:

(1) Choose a site fingerprint type.

(2) Choose the statistics to be computed [*e.g.*, only the mean or the mean, the standard deviation, the minimum, and the maximum].

(3) Compute the site fingerprint feature vector of each atom in a structure. Note that all feature vectors have the same length and that each element in one site fingerprint is of the same type (*e.g.*, tetrahedral LoStOP value) as the element at the same location in a fingerprint of another site.

(4) Calculate statistics across all values of a given feature vector element type (*e.g.*, tetrahedral LoStOP value) and arrange them to a new fingerprint that is representative of the coordination patterns in the entire structure.

In analogy to the site fingerprint, the structure fingerprints are arranged in an ascending order with respect to the coordination number underlying the different feature vector elements (*e.g.*, first come the statistics values from feature types relating to CN = 1, then CN = 2, …). For example, if we consider a generic site fingerprint that is only based on site features *w*_CN=1_, *q*_sgl_bd_|CN = 1, *w*_CN=2_, and *q*_L_|CN = 2 and if we, furthermore, only consider the mean, 
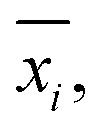
 and the standard deviation, *σ*_*x*_*i*__, as statistics types to be computed, we obtain:

5 **v** = [*w̄*_CN=1_, *σ*_*w*_CN=1__, *q̄*_sgl_bd_|CN = 1, *σ*_*q*_sgl_bd_|CN=1_, *w̄*_CN=2_, *σ*_*w*_CN=2__, *q̄*_L_|CN = 2, *σ*_*q*_L_|CN=2_]^T^

### 2.5 Similarity measures

We consider three common similarity measures for comparing two fingerprint vectors: the Euclidean distance, the dot product, and the cosine similarity.

The Euclidean distance, *d*, is defined as the *L*^2^ norm of the fingerprint difference vector, **v**_*i*_ − **v**_*j*_:

6 *d* = ‖**v**_*i*_−**v**_*j*_‖

7
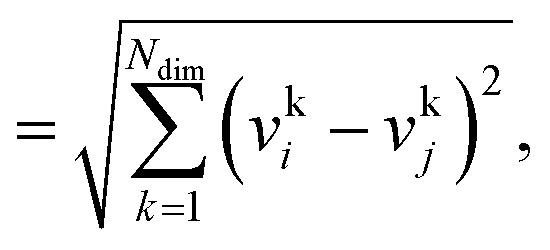
where **v**_*i*_ denotes fingerprint vector *i* and *N*_dim_ the size (or, number of elements) of the vector. The distance can assume values between 0, indicating highest possible similarity, and 
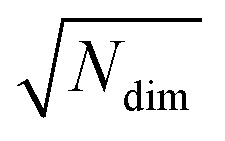
 for lowest theoretically possible similarity, the latter for the condition that the vector elements are constrained between 0 and 1. The *L*^2^ norm is a frequently used (dis)similarity measure (*cf.*, ref. 73 and references therein).

The dot product, *s*_dot_, is defined by:

8 *s*_dot_ = **v**_*i*_·**v**_*j*_

9
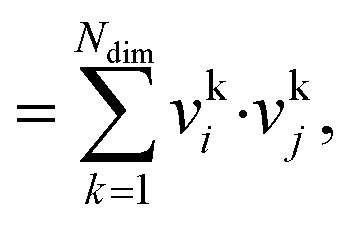
whereas the cosine similarity, *s*_cos_, is given by:10
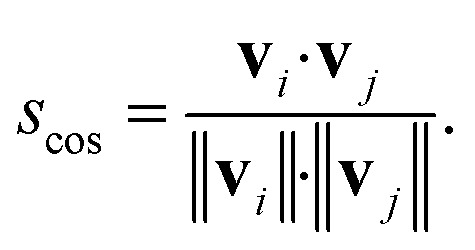


Conceptually, the cosine similarity is especially attractive because we operate in positive space (*i.e.*, vector elements are between 0 and 1) and the similarity measure itself will thus also assume values between 0 and 1. However, note that there should be straightforward ways to convert the distance from a non-normalized dissimilarity measure to a normalized similarity measure; for example, *via*:

11 *s*_dist_ = exp(−*d*).

Finally, we measure the dissimilarity between two probability density distributions *p*_1_ and *p*_2_*via* the overlapping coefficient (OVL),^102^ which is defined by the following integral:12
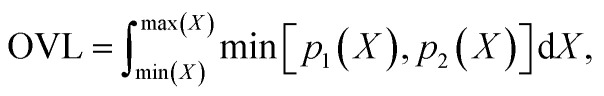
where *X* denotes the variable or observable over which the two distributions are defined.

## 3 Results & discussion

In this section, we present the benchmarking results for (i) using site fingerprints that are based on coordination number likelihood and the local structure order parameters (LoStOPs) to distinguish different local coordination environments and (ii) using structure fingerprints that are, in turn, based on site fingerprints to distinguish different prototype crystal structures.

### 3.1 Site fingerprints

We systematically test the performance of the here introduced site descriptors [local structure order parameters (LoStOPs)] in conjunction with the concept of a site fingerprint to help distinguishing different local coordination environments by employing 61 standard coordination motifs.^103,104^ The motifs are available *via* the ChemEnv module^100^ in pymatgen.^75,76^ In order to focus on the LoStOPs performance we neglect the coordination number likelihood, and we decouple the pattern matching part (*i.e.*, LoStOP calculation) from the neighbor finding part. The latter is possible because the separate motifs are well-defined by identification of a central site and the neighboring sites as provided by the ChemEnv module.^100^ The (simplified) site fingerprint that we use to distinguish unique coordination environments is a vector, **v**^OP^, with 37 components. The vector elements, *v*^OP^_*i*_, are zero if the (observed) motif coordination number is not equal to the target coordination number, whereas the vector element is *q*_*j*_ if the coordination numbers are equal:13
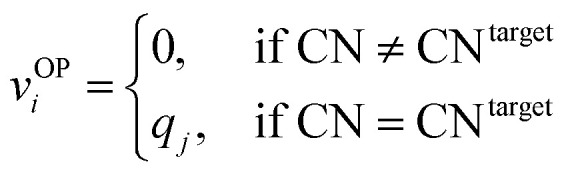


Since we only have a single motif-specific order parameter for coordination numbers beyond 8, we additionally evaluate the bond-orientational order parameters^105^*q*_2_, *q*_4_, and *q*_6_ for coordination numbers from 8 to 13 because those OPs are also helpful in discerning different structural motifs.^80,106,107^ Thus, the resulting vector is:

14 **v**^OP^ = [*q*_sgl_bd_|CN = 1, *q*_bent_(90°)|CN = 2, *q*_bent_(104.45°)|CN = 2, *q*_bent_(120°)|CN = 2, *q*_bent_(150°)|CN = 2, *q*_bent_(180°)|CN = 2, *q*_tri_plan_|CN = 3, *q*_tet_|CN = 3, *q*_T_|CN = 3, *q*_sq_plan_|CN = 4, *q*_tet_|CN = 4, *q*_see_saw_rect_|CN = 4, *q*_tri_bipyr_|CN = 4, *q*_tri_pyr_|CN = 4, *q*_pent_plan_|CN = 5, *q*_sq_pyr_|CN = 5, *q*_tri_bipyr_|CN = 5, *q*_hex_plan_|CN = 6, *q*_oct_|CN = 6, *q*_pent_pyr_|CN = 6, *q*_hex_pyr_|CN = 7, *q*_pent_bipyr_|CN = 7, *q*_bcc_|CN = 8, *q*_hex_bipyr_|CN = 8, *q*_2_|CN = 9, *q*_4_|CN = 9, *q*_6_|CN = 9, *q*_2_|CN = 10, *q*_4_|CN = 10, *q*_6_|CN = 10, *q*_2_|CN = 11, *q*_4_|CN = 11, *q*_6_|CN = 11, *q*_cuboct_|CN = 12, *q*_2_|CN = 12, *q*_4_|CN = 12, *q*_6_|CN = 12]^T^.

where *q*_bent_(*α*) refers to the bent LoStOP with a target angle of *α*, *q*_tet_|CN = 3, aims at identifying trigonal non-coplanar environments, and *q*_tri_bipyr_|CN = 4 should work together with *q*_tri_pyr_|CN = 4 to identify regular see–saw motifs (angle: 120°).

**Fig. 9:**
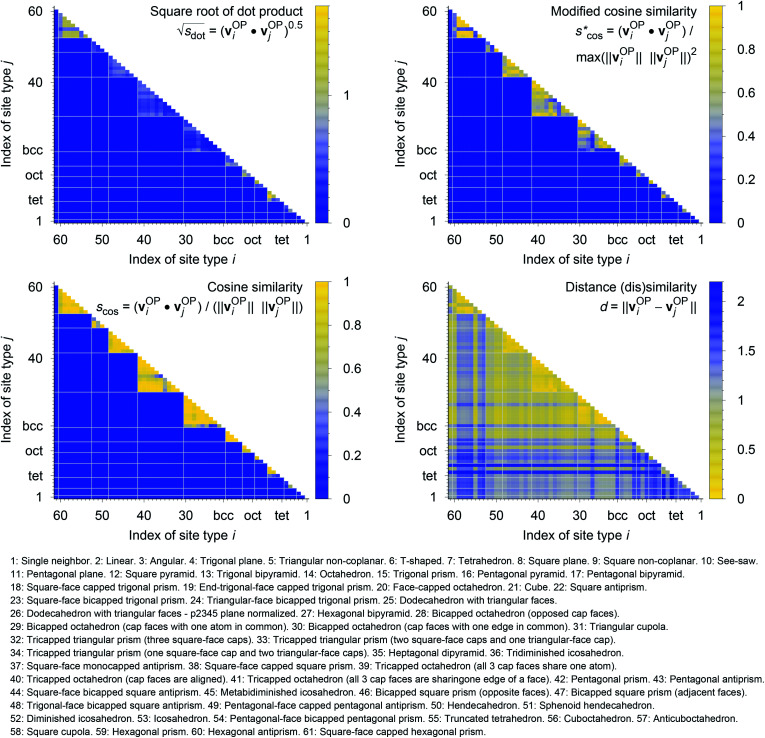
(Dis)similarity between 61 standard coordination environments based on a simplified site fingerprint, **v**^OP^, that is defined by local structure order parameters only; top left: square root of the dot product, 
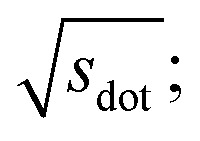
 top right: modified cosine similarity, 
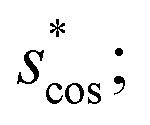
 bottom left: cosine similarity, *s*_cos_; bottom right: distance dissimilarity, *d*. The names of the coordination environments are given beneath the plots along with the respective indices used for plotting purposes. For clarity, the white grid lines separate coordination environments with different coordination numbers.

In Fig. 9, we compare results obtained using four site fingerprint similarity metrics: the square root of the dot product, 
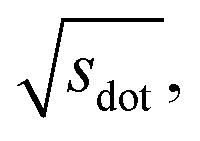
 (top left), the modified (top right) and conventional (bottom left) cosine similarity, 
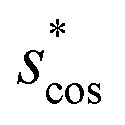
 and *s*_cos_, respectively, and the distance (or *L*^2^ norm), *d*, (bottom right). By construction of the simplified site fingerprint **v**^OP^, the square root of the dot product and the cosine similarities are zero (blue color) for any two site fingerprints obtained from coordination environments that have different coordination numbers [*e.g.*, tetrahedron (CN = 4) *vs.* octahedron (CN = 6)] because those are strictly orthogonal. The conventional cosine similarity is, however, in many cases close to unity (yellow color) when we consider coordination environments which have the same coordination number (*cf.*, yellow/gray triangles along the diagonal in bottom left panel).

To understand this potentially unexpected behavior let us consider some concrete examples: the 6-fold coordinated motifs octahedron, pentagonal pyramid, and trigonal prism. The three non-zero elements of the fingerprints are *q*_hex_plan_|CN = 6, *q*_oct_|CN = 6, as well as *q*_pent_pyr_|CN = 6. Removing the zero entries, the resulting motif fingerprints are:**v**^OP^_oct_ = [0.20, 1.00, 0.50]^T^,**v**^OP^_pent_pyr_ = [0.10, 0.50, 1.00]^T^, and**v**^OP^_trig_prism_ = [0.03, 0.28, 0.48]^T^.

Thus, the dot products are 1.02, 0.53, and 0.62 for motif pairs oct–pent_pyr, oct–trig_prism, and trig_prism–pent_pyr, respectively, whereas the corresponding cosine similarities are 0.8, 0.83, and 0.997. Since the maximum dot product is around 3.2 given our motif test set, all three dot products are small compared to this upper bound. On the contrary, the cosine similarities are all close to the inherent upper bound of 1. Evidently, the normalization term ‖**v**^OP^_*i*_‖ × ‖**v**^OP^_*j*_‖ changes the dot products in an undesirable manner, which is true in particular for the last pair (trig_prism–pent_pyr). The problem is that the “length” of the fingerprint carries actually important information. The “angle” or alignment of the fingerprints alone is, thus, not sufficient information for similarity purposes (it is however crucially important and works well if we consider coordination environments with different CN, as we have seen). To clarify this point further consider the pair pentagonal pyramid–trigonal prism. The fingerprint of the first motif is approximately the same as the fingerprint of the second motif multiplied by 2: **v**^OP^_pent_pyr_ ≈ 2 × **v**^OP^_trig_prism_. Although the two fingerprints are parallel, they have very different interpretations. The first fingerprint, **v**^OP^_pent_pyr_, has an order parameter that equals 1 and, thus, flags a perfect motif, whereas all elements of the second fingerprint, **v**^OP^_trig_prism_ are markedly smaller than 1 (maximum: ≈0.5), thus, signifying no perfect motif match (given our set of LoStOPs). Hence, the cosine similarity can be a misleading similarity metric, especially if we consider same-coordination number motifs. Before moving on, we like to highlight at this point the nice symmetric behavior of *q*_pent_pyr_ and *q*_oct_ : *q*_pent_pyr_ gives a value of 0.5 for a perfect octahedron and, *q*_oct_, in turn, gives 0.5 for a perfect pentagonal pyramid.

Omitting the normalization term of the cosine similarity but taking the square root of the dot product to preserve the units, we obtain a similarity metric that can alleviate the problem with putatively similar same-coordination number environments (*cf.*, top left panel in Fig. 9), as can replacing the typical normalization constant ‖**v**^OP^_*i*_‖·‖**v**^OP^_*j*_‖ with max^2^(‖**v**^OP^_*i*_‖, ‖**v**^OP^_*j*_‖) to some extent (top right panel). So, choosing the square root of the dot product instead of the cosine similarity has here the advantage that the vast majority of coordination environments are identified as dissimilar, as expected. On the other hand, we lose the nice behavior of the similarity metric to be constrained between zero and unity. The majority of square root of the dot products for same-coordination number motifs are ≤1. Thus, 
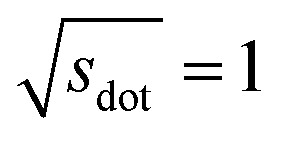
 seems to be a reasonable *ad-hoc* threshold value for distinguishing any coordination environments by means of the here introduced site fingerprints and the square root of the dot product similarity metric.

Finally, the distance dissimilarity metric (bottom right panel), for which we expected high values for the majority of cases (*i.e.*, also blue color because the range was inverted), gives a rather diffuse picture. A subtle trend might be seen because high (≈2: blue) and moderate (≈1: gray) values are predominant along the axes, whereas small distances (≈0: yellow) accumulate in the center of the graph. But the overall results strongly suggest that the square root of the dot product is the best similarity metric for coordination environment comparison using the here introduced order parameter-based site fingerprints.

The detailed analysis of the site fingerprints underscores that the LoStOPs can reliably distinguish between motifs sharing the same coordination number (*e.g.*, tetrahedron *vs.* square planar). This capability has already been exploited to develop a computational tool that can automatically detect sites in crystal structures (*e.g.*, “tet” and “oct”) and translate the information into human readable text (*e.g.*, “This site is a tetrahedral site”),^108^ thus, demonstrating the unique power of the here introduced tools and concepts.

### 3.2 Structure fingerprints

The order parameter-based site fingerprints work well for distinguishing many different isolated coordination environments, especially if the square root of the dot product is used as a similarity metric. In this section, we aim to leverage this newly discovered powerful capability to accomplish a more complex, yet exceptionally important, task in materials science: automatically distinguishing and quantitatively comparing different (prototype) crystal structures using the earlier introduced concept of structure fingerprints.

**Fig. 10:**
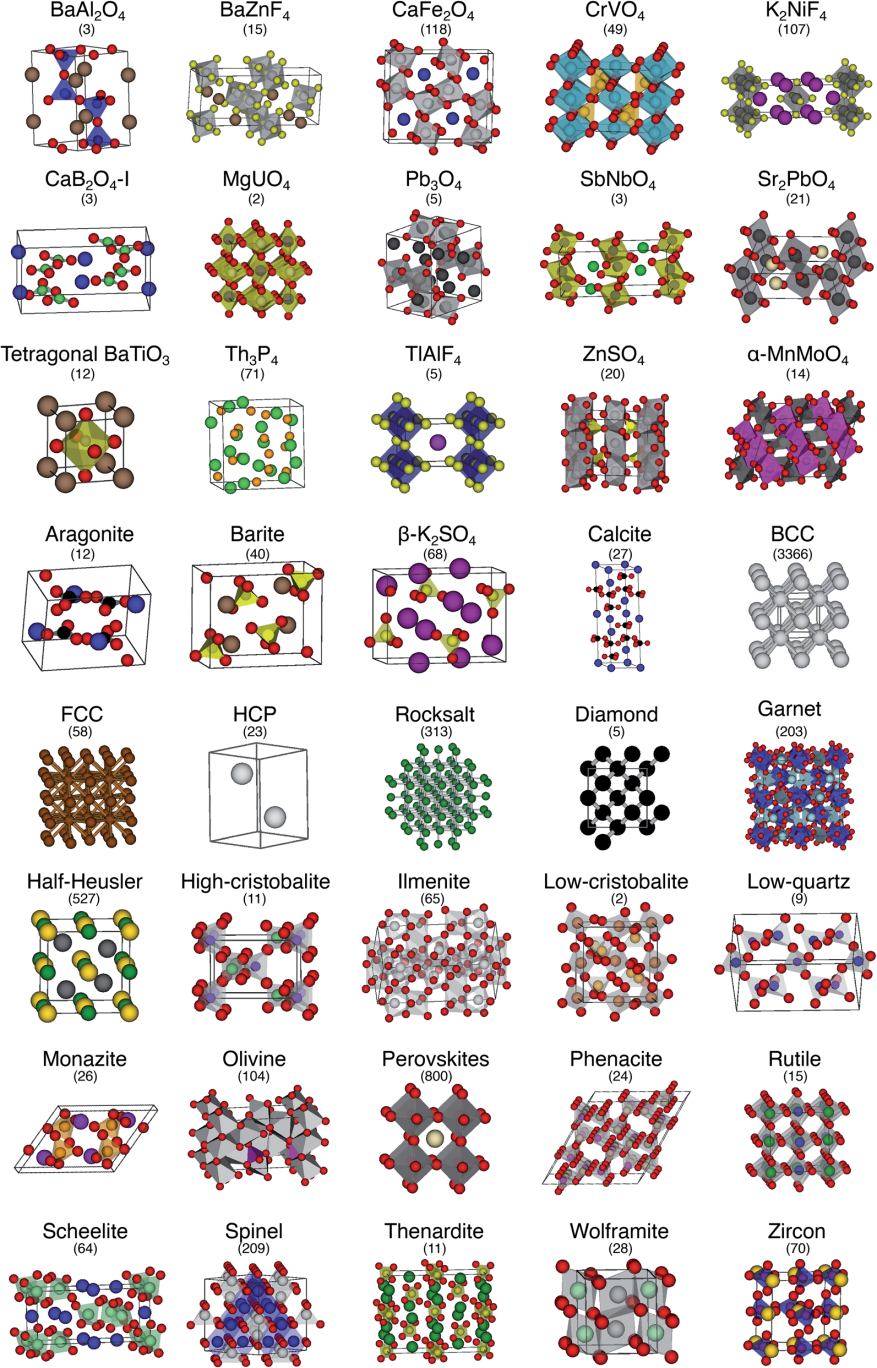
The 40 prototype structures considered in this work for testing the performance of structure similarity assessment with our novel structure fingerprints. In parentheses, we provide the number of materials that we found in the MP database^11,12^ for a given prototype group.

In order to thoroughly test our structure fingerprints we have constructed a benchmark test set consisting of 40 groups of relevant prototype structures (Fig. 10) ranging from metals over semiconductors to insulators. Some of the groups stem from one of our previous papers,^80^ but we have systematically and significantly increased the number of groups using a canonical reference: “The Major Ternary Structure Families” by Muller and Roy.^59^ We included the main structure of any structure family given by ref. 59. For example, we included regular spinel, but we did not include spinels with Jahn–Teller distortions such as hausmannite. In the future, we will further increase the test set. Given a structure prototype described in ref. 59, we proceed by identifying the respective structure in the Materials Project^11^ database—typically *via* the chemical formula and the space group. Subsequently, we use pymatgen's structure matcher in conjunction with a framework comparator for finding more structures of the same (framework) prototype but with different chemistry, as we did previously.^80^ This procedure yields 6528 structures, each of which belongs to one of the 40 prototype groups and all together amounting to a test set that comprises almost 10% of the entire MP database^11^ (currently:^12^ 69 640 inorganic structures).

We consider the cosine similarity and the distance dissimilarity metrics for testing the performance of distinguishing prototype structure groups on the basis of our structure fingerprints. Furthermore, we test 3 different site fingerprint definitions with different options and presets, resulting in 8 distinctly different site fingerprint types, and we investigate 7 different combinations of tentatively relevant statistics types: the mean of each coordination feature across all sites; mean and maximum; mean and minimum; mean and standard deviation; mean, standard deviation, and minimum; mean, standard deviation, and maximum; as well as mean, standard deviation, minimum, and maximum. In summary, we thus test 2 × 8 × 7 = 112 different ways of assessing structure similarity for each of the 40 structure groups separately and for all groups together. Note that each structure group is weighted equally; that is, we remove the bias of a group that has many structure members (*e.g.*, bcc + CsCl + Heusler: 3366) in comparison to a group that only features a few structures (low-cristobalite: 2).

**Fig. 11:**
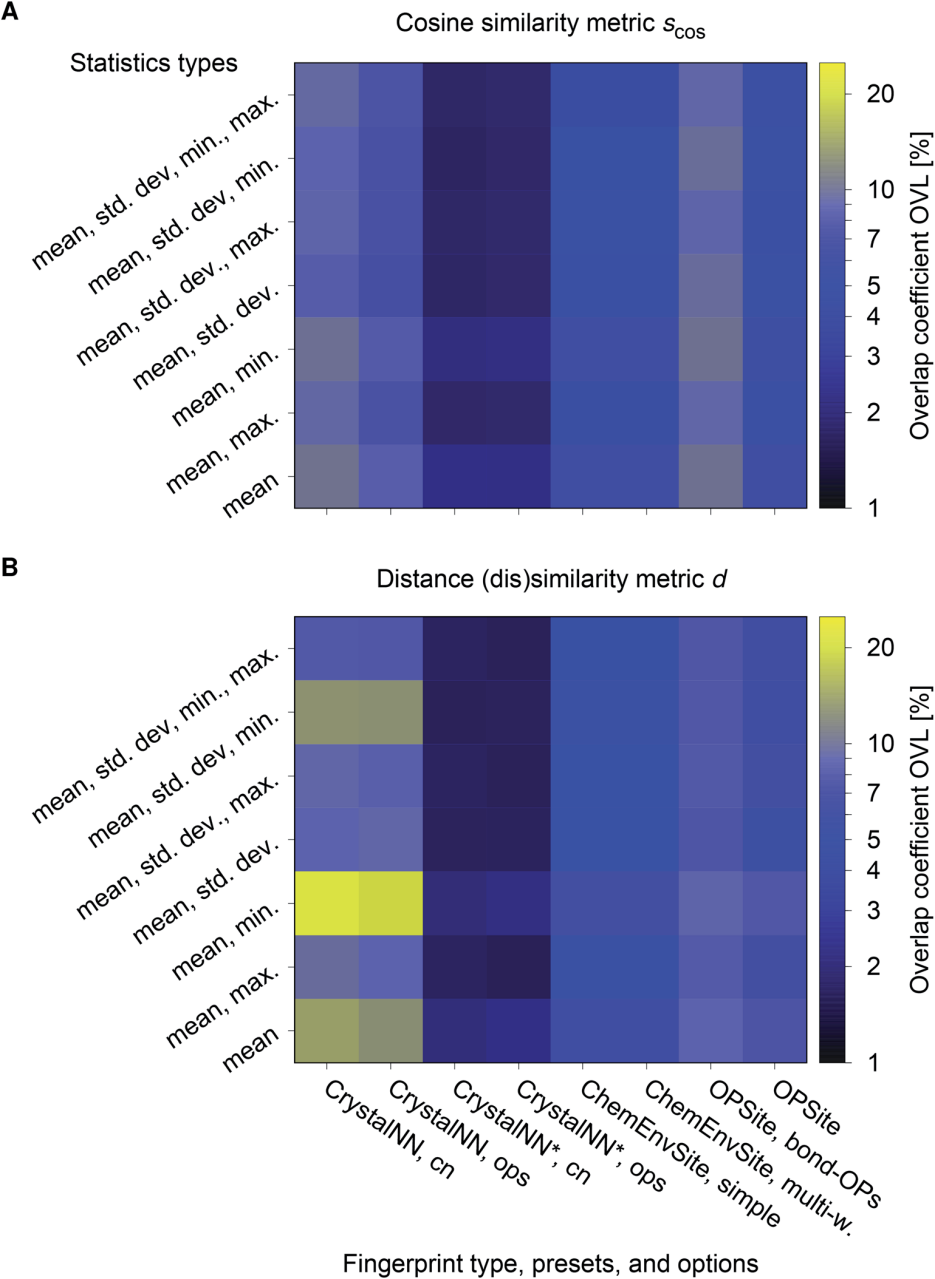
Overlapping coefficient, OVL, as a function of (i) the underlying site fingerprint and (ii) the statistics run across all site fingerprints in a structure, both together centrally define a given structure fingerprint, and (iii) as obtained by using the cosine similarity, *s*_cos_, (A) and the distance (dis)similarity metric, *d*, (B) for comparing two structure, respectively. Note that “CrystalNN, cn” and “CrystalNN, ops” refer to the CrystalNNFingerprint in matminer using preset “cn” and “ops,” respectively, an additional asterisk indicates that the two available fingerprint options are set to none, “ChemEnvSite, simple” and “ChemEnvSite, multi-weight” refer to the ChemEnvSiteFingerprint in matminer using the “simple” and “multi-weight” preset, respectively, “OPSite” and “OPSite, bond-OPs” refer to the OPSiteFingerprint with the default OP set and using bond-orientational order parameters^105^ for all coordination numbers tested (*i.e.*, CN = 1, …, 12), and “std. dev.”, “min.”, as well as “max.” refer to standard deviation, minimum, and maximum, respectively.

The best performing combination (OVL: 1.9%) is the CrystalNNFingerprint with “ops” preset and all additional flags turned off, mean and maximum as statistics types, as well as distance as comparison metric (Fig. 11). The mean as statistics type alone already provides excellent results (OVL: 2.6%), which can be regarded as a reflection of Pauling's 5th rule, the Rule of Parsimony:^109^ “the number of essentially different kinds of constituents in a crystal tends to be small.” Furthermore, it is interesting that the “cn” preset variant, which uses only coordination number likelihoods, *w*_CN=*j*_, and, thus, no order parameters at all, performs similarly well (OVL: 1.8%) while having 183/72 = 2.5 times less features in the structure fingerprint. Concepts like the Bayesian^110^ or Akaike information criterion^111^ that aim at guiding model selection might suggest here that we added too many parameters to our model. However, we stress that the local structure order parameter features are not simply “random” features that we test in a “black box” approach. The features have clear and, most importantly, desirable interpretations or capabilities: they can reliably identify coordination environments, as the previous section unequivocally highlighted. Since the structure fingerprint results underscore that the most basic coordination information suffices to reliably distinguish different structures, this hints at a different problem.

The issue seems to be that our test set might not be diverse enough in order to make the LoStOPs more critical components of the structure fingerprints. This can be understood when recalling that occurrence statistics of different coordination motifs are not uniform, but there are certain motifs that are observed disproportionately more frequently. In particular, tetrahedra and octahedra are the most frequently occurring coordination motifs for anions. For example, we found in the structures that we used from ref. 59 that 23 structures have (anion) octahedral sites, 21 structures with tetrahedral sites, as well as 3 with trigonal planar and with 2 trigonal non-coplanar sites. Furthermore, almost all of these tetrahedral and octahedral sites are nearly perfect Platonic polyhedra. Consequently, the issue or bias that there are only a few important perfect motifs and that the vast majority of coordination environments considered in the previous section do not occur frequently enough is a natural external condition in the present context. Nonetheless, our results indicate that the key to a reliable coordination environment analysis^80^ and, thus, to a coordination environment-based structure similarity assessment lies in the accuracy of the neighbor finding method.

**Fig. 12:**
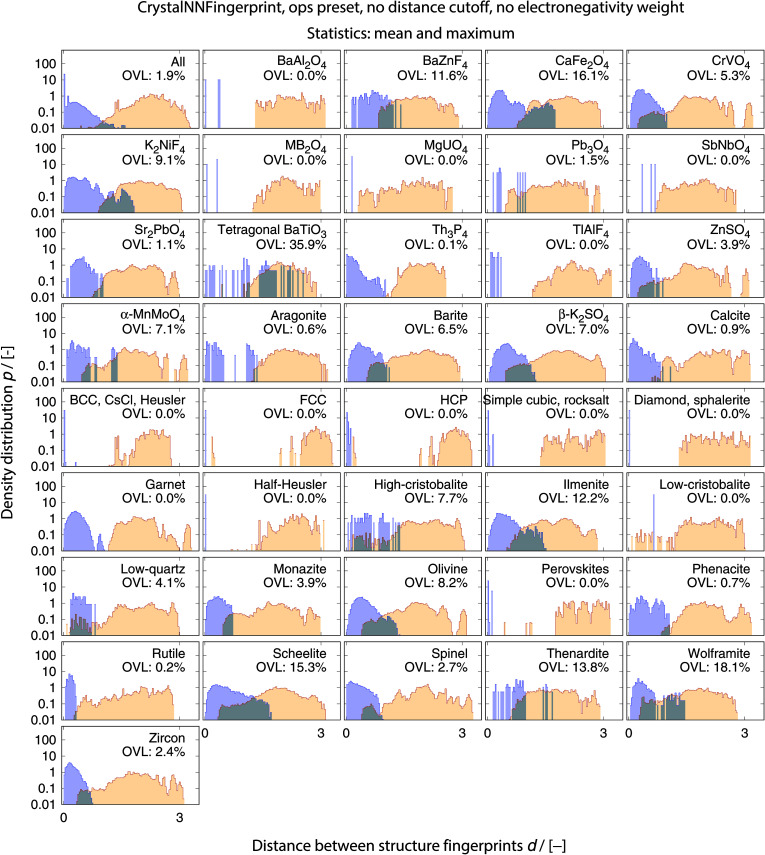
Distributions of distances between structure fingerprints, ‖**v**_struct,*i*_ − **v**_struct,*j*_‖, for measuring dissimilarities between structures that belong to the same prototype group (blue) and between structures that belong to different prototype groups (orange) for the optimal fingerprint settings, *etc.* The overlapping coefficients (OVLs), which quantify the overlap between the two distributions in each panel, are also provided. The top left panel represents results averaged over data from all prototype structure groups, whereas the remaining panels display the results for a specific target prototype structure group.

The full collection of results for structure group (dis)similarity is provided in the ESI.† In Fig. 12, we present the data using the setup that gives the smallest overall overlapping coefficient. The blue lines indicate the distributions obtained from calculating distances between fingerprints from structure of the same prototype group, whereas the orange lines are the distribution of structure fingerprint distances to members of other prototype groups. The results for each prototype group separately underline that our approach using the overlapping coefficient is a valuable choice because, in most cases, there is a nice consistent separation between the blue area on the left and orange area in the center and on the right. Very few groups exhibit slightly misleading results such as low-cristobalite (right column, 6th panel from the top). Despite the very low OVL (0.5%) observed for this group, there are a lot of unlike structures having a smaller distance to a member of the low-cristobalite group than the peak location representing the (little) distance variation within the low-cristobalite prototype group itself.

We emphasize that our new structure similarity quantification procedure has a very important advantage to conventional structure matching or similarity methods. It does not only provide a global similarity value (*e.g.*, RMSD). The separate elements of the fingerprints and, thus, difference vectors also carry local coordination information in a very compact way, which can be readily used in machine learning approaches and in formulating design rules. For example, consider diamond (C, mp-66), rocksalt (NaCl, mp-22862), and α-iron (Fe, mp-13) as well as a Heusler (Cu_2_MnSn, mp-22221), a spinel (MgAlO_3_, mp-3536), and a perovskite material (CaTiO_3_, mp-5827). Ignoring zero entries, the values of the best performing fingerprint for similarity assessment (“CrystallNN*” with “ops” preset and mean and maximum values) are provided in Table 2.

**Table 2:** Examples of structure fingerprints

Structure	Leading fingerprint elements		
Diamond^a^	*w̄*_CN=4_ = 1	*q̄*_tet_|CN = 4 = 1	*q̄*_tri_pyr_|CN = 4 = 0.25
	*q̄*_see_saw_|CN = 4 = 0.23	*q̄*_sq_plan_|CN = 4 = 0.08	*q̄*_see_saw_rect_|CN = 4 = 0.01
Rocksalt^a^	*w̄*_CN=6_ = 1	*q̄*_oct_|CN = 6 = 1	*q̄*_pent_pyr_|CN = 6 = 0.5
	*q̄*_hex_plan_|CN = 6 = 0.2		
α-iron^a^	*w̄*_CN=8_ = 0.58	*q̄*_bcc_|CN = 8 = 0.58	*q̄*_hex_bipyr_|CN = 8 = 0.25
	*w̄*_CN=14_ = 0.42		
Heusler (Cu_2_MnSn)	*w̄*_CN=4_ = 0.09	max(*w*_CN=4_) = 0.18	*q̄*_tet_|CN = 4 = 0.09
	max(*q*_tet_|CN = 4) = 0.18	*q̄*_see_saw_|CN = 4 = 0.02	max(*q*_see_saw_|CN = 4) = 0.04
	*q̄*_tri_pyr_|CN = 4 = 0.02	max(*q*_tri_pyr_|CN = 4) = 0.04	*q̄*_sq_plan_|CN = 4 = 0.01
	max(*q*_sq_plan_|CN = 4) = 0.01	*w̄*_CN=8_ = 0.54	max(*w*_CN=8_) = 0.6
	*q̄*_bcc_|CN = 8 = 0.54	max(*q*_bcc_|CN = 8) = 0.6	*q̄*_hex_bipyr_|CN = 8 = 0.24
	max(*q*_hex_bipyr_|CN = 8) = 0.26	*w̄*_CN=14_ = 0.37	max(*w*_CN=14_) = 0.6
Spinel (MgAgO_3_)	*w̄*_CN=1_ = 0.01	max(*w*_CN=1_) = 0.02	*w̄*_CN=4_ = 0.7
	max(*w*_CN=4_) = 1	*q̄*_tet_|CN = 4 = 0.43	max(*q*_tet_|CN = 4) = 1
	*q̄*_tri_pyr_|CN = 4 = 0.34	max(*q*_tri_pyr_|CN = 4) = 0.53	*q̄*_see_saw_|CN = 4 = 0.33
	max(*q*_see_saw_|CN = 4) = 0.52	*q̄*_rect_see_saw_|CN = 4 = 0.23	max(*q*_rect_see_saw_|CN = 4) = 0.4
	*q̄*_sq_plan_|CN = 4 = 0.17	max(*q*_sq_plan_|CN = 4) = 0.28	*w̄*_CN=6_ = 0.29
	max(*w*_CN=6_) = 1	*q̄*_oct_|CN = 6 = 0.24	max(*q*_oct_|CN = 6) = 0.82
	*q̄*_pent_pyr_|CN = 6 = 0.13	max(*q*_pent_pyr_|CN = 6) = 0.44	*q̄*_hex_plan_|CN = 6 = 0.06
	max(*q*_hex_plan_|CN = 6) = 0.2	max(*w*_CN=8_) = 0.01	max(*q*_bcc_|CN = 8) = 0.01
Perovskite (CaTiO_3_)	*w̄*_CN=2_ = 0.31	max(*w*_CN=2_) = 0.52	*q̄*_lin_|CN = 2 = 0.31
	max(*q*_lin_|CN = 2) = 0.52	*q̄*_150°_|CN = 2 = 0.04	max(*q*_150°_|CN = 2) = 0.06
	*w̄*_CN=6_ = 0.49	max(*w*_CN=6_) = 1	*q̄*_oct_|CN = 6 = 0.49
	max(*q*_oct_|CN = 6) = 1	*q̄*_pent_pyr_|CN = 6 = 0.24	max(*q*_pent_pyr_|CN = 6) = 0.5
	*q̄*_hex_plan_|CN = 6 = 0.1	max(*q*_hex_plan_|CN = 6) = 0.2	*w̄*_CN=12_ = 0.2
	max(*w*_CN=12_) = 1	*q̄*_cuboct_|CN = 12 = 0.2	max(*q*_cuboct_|CN = 12) = 1
	*q̄*_6_|CN = 12 = 0.12	max(*q*_6_|CN = 12) = 0.58	*q̄*_4_|CN = 12 = 0.04
	max(*q*_4_|CN = 12) = 0.19		

a We omit the maximum values of the fingerprint for sake of brevity because those values were identical to the mean values.

Diamond's leading fingerprint elements are the mean 4-fold coordination likelihood and the mean tetrahedral LoStOP. Because diamond^112^ consists exclusively of tetrahedrally coordinated carbon atoms both LoStOP entries are 1, whereas all other 4-fold coordinated LoStOPs are much smaller (≤0.25). Similarly, rocksalt^113^ has only octahedrally coordinated Na^+^ and Cl^−^ ions, for which reason the mean 6-fold coordination likelihood and the mean octahedral LoStOP is 1 and all other 6-fold coordination descriptors considerably smaller.

All sites in α-iron^114^ and the full Heusler material^115^ Cu_2_MnSn are expected to be coordinated 8-fold in a body centered cubic manner. Among the leading fingerprint elements, we find in fact *w̄*_CN=8_ and *q̄*_bcc_|CN = 8, but their deviation from 1 (0.58 and 0.54, respectively) and a slightly higher maximum value together with max(*w*_CN=14_) flag a special case. The second nearest neighbors in BCC structures are quite close to a given site in comparison to nearest neighbors,^80^ and the structure produces rather large Voronoi facets for those second shell atoms.^107^ Hence, it is not unreasonable to consider the second nearest neighbors as being directly coordinated to a given site—only with a smaller coordination likelihood. Also note that, in the case of α-iron, the 6 second nearest neighbors exhibit an as small contribution as 0.21 to the 14-fold coordination likelihood if the contribution of the 8 nearest neighbors can be set to *w*_CN=8_ = 0.58.

The MgAlO_3_ spinel structure possesses perfect MgO_4_ tetrahedra, which share corners with slightly distorted AlO_6_ octahedra.^59^ Since the oxygen atoms are 4-fold coordinated just as the Mg ions,^59^ 80% of the sites are 4-fold coordinated, which explains the high value of *w̄*_CN=4_ = 0.7 (all Mg and O site *w*_CN=4_ ≥ 0.98). The fact that the mean tetrahedrality is markedly larger than 1/5 indicates that there are more sites that have a certain tetrahedral character than only Mg sites with *q*_tet_ = 1: the oxygen sites with *q*_tet_ ≈ 0.5. However, several of the maximum values of the other 4-fold coordination motif LoStOPs are also around 0.5, thus, underlining that the oxygen atoms have no distinctly identifiable coordination motif. The distorted AlO_6_ octahedra are clearly represented by max(*w*_CN=6_) = 1 and max(*q*_oct_|CN = 6) = 0.83.

Ultimately, ideal perovskite materials such as CaTiO_3_ have Ca atoms that are 12-fold coordinated by oxygen atoms in a cuboctahedron fashion, perfect TiO_6_ octahedra, and oxygen atoms that are considered 6-fold coordinated (2 Ti atoms are nearest neighbors forming linear bonds, but the 4 next nearest Ca neighbors are also considered coordinated to oxygen atoms).^59^ All these features are nicely reproduced by our structure fingerprint. 60% of the sites (oxygen atoms) can to some degree (*w*_CN=2_ ≈ 0.5) be considered 2-fold coordinated (0.6 × *w*_CN=2_ ≈ *w̄*_CN=2_ = 0.31). And, we can infer the linear bond character of these sites by comparing max(*w*_CN=2_) and max(*q*_lin_|CN = 2) values. The perfect TiO_6_ octahedron results in max(*w*_CN=6_) = max(*q*_oct_|CN = 6) = 1, whereas the corresponding mean values of ≈0.5 indicate that more sites than the Ti atoms possess distinct octahedral coordination geometry: the oxygen atoms. Finally, *w̄*_CN=12_ = 0.2 and max(*w*_CN=12_) = 1 clearly highlight the 20% of sites (Ca) that are perfectly 12-fold coordinated, and *q̄*_cuboct_|CN = 12 = 0.2 and max(*q*_cuboct_|CN = 12) = 1 that the coordination geometry is a perfect cuboctahedron.

### 3.3 Discussion

Two aspects of our work require additional discussion: our novel neighbor-finding algorithm “CrystallNN” (CNN) and the metric used to assess similarity—especially between structures.

#### 3.3.1 Novel neighbor-finding method

The structure similarity analysis suggests that CNN is a valuable new way of finding neighbors and computing coordination numbers. It yields better structure similarity assessments than the minimum-distance near-neighbor finding method used in the OPSite structure fingerprint and the conventional Voronoi method used by the ChemEnvSite fingerprint (Fig. 11). Here, it does not matter whether or not to use our new order parameters for structure similarity assessment, given our structure test set. Essentially, the usage of CNN-based coordination likelihoods lends the CrystallNN structure fingerprint its similarity assessment power. Note also that we are currently investigating the performance of CNN for predicting coordination numbers in greater detail and that CNN frequently outperforms conventional approaches in the upcoming work.

We introduced, to the best of our knowledge, a new element to the neighbor-finding issue: the coordination likelihood computation using the normalized Voronoi weights in conjunction with a semicircle. We invented this approach in an adaptive data analysis^116^ fashion. Given a small number of pathological cases, we optimized our algorithm *via* trial-and-error. We stress here however that we did not use the entire benchmark test set for structure similarity assessment (Fig. 10). Therefore, the algorithm development procedure resembled a model selection process, where we took care about not using the same test set for testing.

The new element of our method improved structure similarity assessment. However, we have to underscore that the approach might appear purely algorithmic in nature. It lacks any theory or physical model justifying, for example, the specific use of the semicircle. This can be interpreted as a conceptual disadvantage over more direct or intuitive approaches such as the minimum distance neighbor-finding method.

#### 3.3.2 Similarity metric issue

The results of the site fingerprints clearly indicate that dot product-based similarity metrics are advantageous for more stringently distinguishing dissimilar coordination environments. The situation is more nuanced in the case of the structure fingerprints because the overlapping coefficient (OVL) suggests that the distance (dis)similarity metric gives the best performance. However, this outcome should be viewed cautiously.

On an average, the cosine similarity yields an OVL that is ≈0.01 smaller (or ≈11%) than the distance (dis)similarity. Using only the CNN data (“CrystalNN*, ops” and “CrystalNN*, cn” in Fig. 11), the trend is opposite (≈−0.001, ≈−7%) but considerably weaker. Hence, the (dis)similarity metric does not significantly impact the outcome of the best performing group of fingerprints that we have considered. This is confirmed by comparing results of individual material prototype groups of the best combination of fingerprint and (dis)similarity metric (Fig. 12) with the same fingerprint but cosine similarity metric (ESI: Fig. 26†). The OVLs are usually almost identical. There are in fact only two cases (tetragonal BaTiO_3_ and ilmenite) for which the OVLs differ more evidently (≈−5% and ≈+4%, respectively). However, the corresponding OVLs are also exceptionally large (36% and 12%, respectively) so that we conclude that even these few extreme cases represent only moderate discrepancies between the two (dis)similarity metrics. Moreover, distribution features such as fragmentation (tetragonal BaTiO_3_) and second similarity peaks (aragonite) are similarly clearly observable with both (dis)similarity metrics.

Because normalization is very desirable in most machine learning approaches,^117^ we conclude that the cosine similarity metric is—at the very least—an often unjustifiably ignored metric. We can solidly recommend to use the cosine similarity as an *ad-hoc* assessment metric for computational materials science applications.

## 4 Conclusions

Despite the facts that coordination chemistry was already established more than a century ago^118^ and Brunner, more than 4 decades ago, still realized that “the term coordination ha[d] no satisfying definition,”^87^ determination and evaluation of coordinated neighbors in crystal structures is even today continuing to be a nontrivial scientific task—let alone automated versions of those processes. To aid in filling this gap, we have, in this work, introduced a new neighbor finding method and several new local structure order parameters (LoStOPs). We have used a Monte Carlo framework to maximize comparability between different LoStOPs. Subsequently, we have employed the new descriptors to define feature vectors that are characteristic of known coordination environments (site fingerprints and similarities) and that are capable of distinguishing commonly investigated crystalline prototype materials (structure fingerprints and similarities). In-depth testing has enabled us to give recommendations which type of fingerprint should be combined with which similarity metric in order to most reliably categorize site environments and crystal structures. We actively utilize our novel capabilities on the Materials Project^11^ website^12^ to assess the crystal structure similarity between different materials. To this end, we have computed all ≈2.45 billion structure similarity distances between each pair of the ≈70 000 materials in the MP database. The structure similarity data greatly facilitate browsing of the website and, thus, exploration of the MP materials database because it adds an intuitive connection mechanism.

We hope that our novel coordination descriptors will be helpful in the context of inverse design approaches,^119,120^ where, specifically, the distinction between perfect and slightly distorted coordination environments^121^ or building blocks^27,28^ is decisive for the target property. Finally, we believe that the here introduced methods and concepts, which are freely available through the python^74^ packages pymatgen^75,76^ and matminer,^65,77^ will greatly facilitate future data-driven and machine learning studies at the interface between materials science, chemistry, and engineering, for example, by producing standardized metadata.^122^

## Conflicts of interest

There are no conflicts to declare.

## Supplementary Material

RA-010-C9RA07755C-s001
