# Supplementary material for: Local structure order parameters and site fingerprints for quantification of coordination environment and crystal structure similarity
Source: RSC Adv. 2020 Feb 7;10(10):6063–81. doi: 10.1039/c9ra07755c (PMC9049235; doi:10.1039/c9ra07755c)
Supplement: RA-010-C9RA07755C-s001 [file RA-010-C9RA07755C-s001.pdf]

Electronic Supplementary Information: Local structure order parameters and site fingerprints for quantification of coordination environment and crystal structure similarity

Nils E. R. Zimmermann,*[a] and Anubhav Jain[b]

[a] *Energy Technology Area, Lawrence Berkeley National Laboratory, Berkeley, California 94720, United States. Tel: 0049 177 9077 532; E-mail: nils.e.r.zimmermann@gmail.com*
[b] *Energy Technology Area, Lawrence Berkeley National Laboratory, Berkeley, California 94720, United States.*

Contents

1 Additional Results 3

2 List of Symbols 116

1 Additional Results

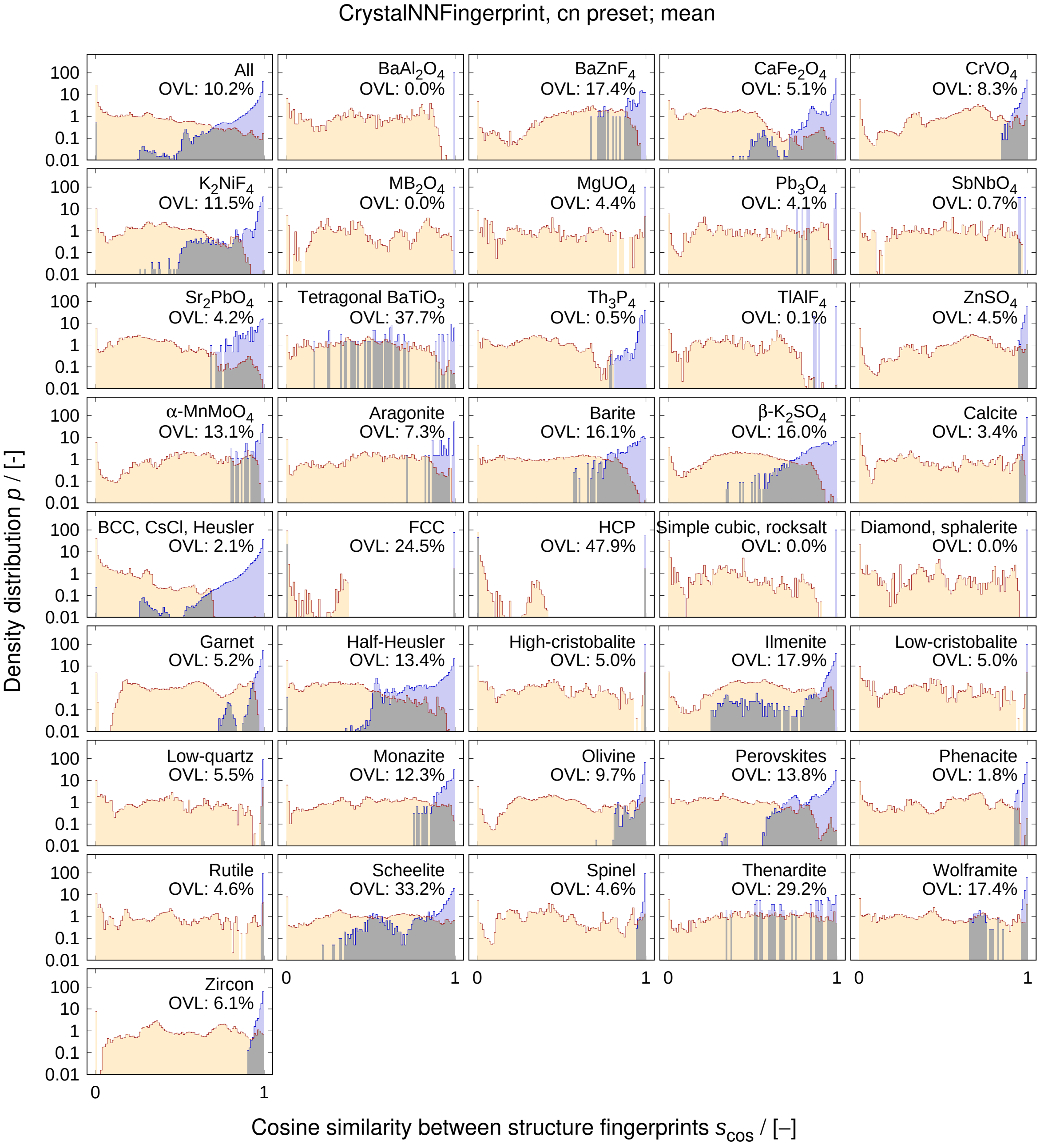

Fig. 1 Additional structure group (dis)similarity results.

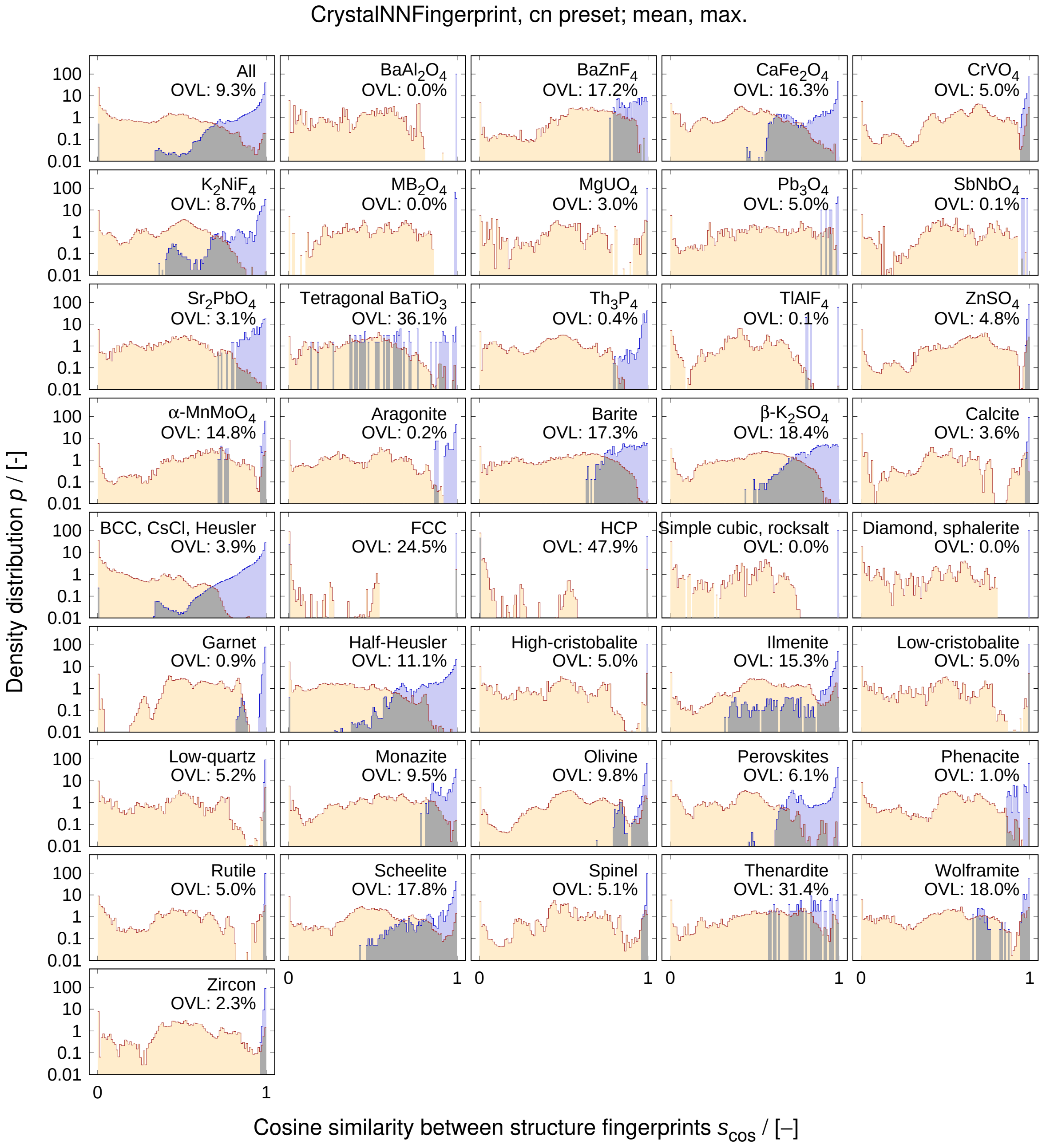

Fig. 2 Additional structure group (dis)similarity results.

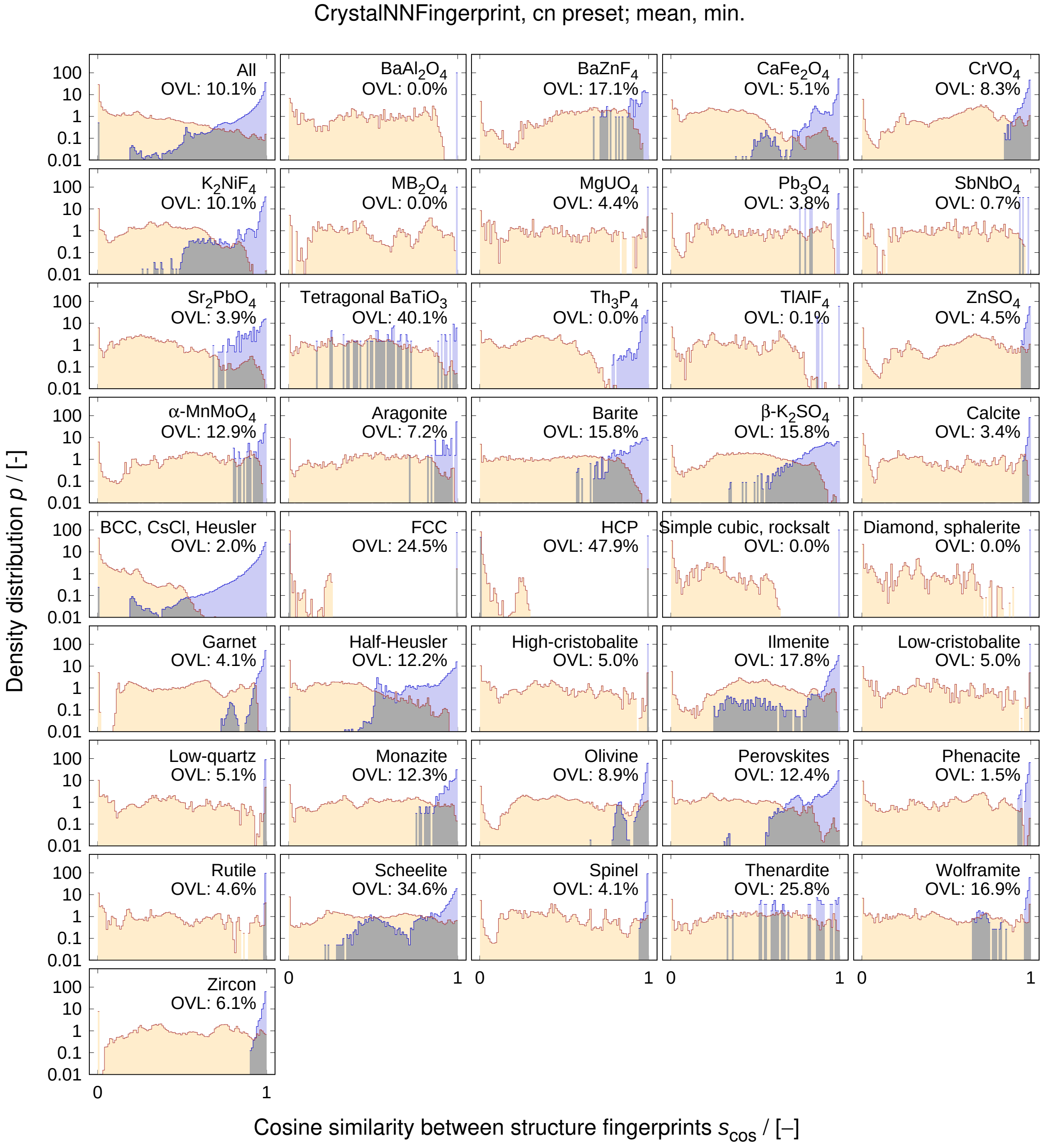

Fig. 3 Additional structure group (dis)similarity results.

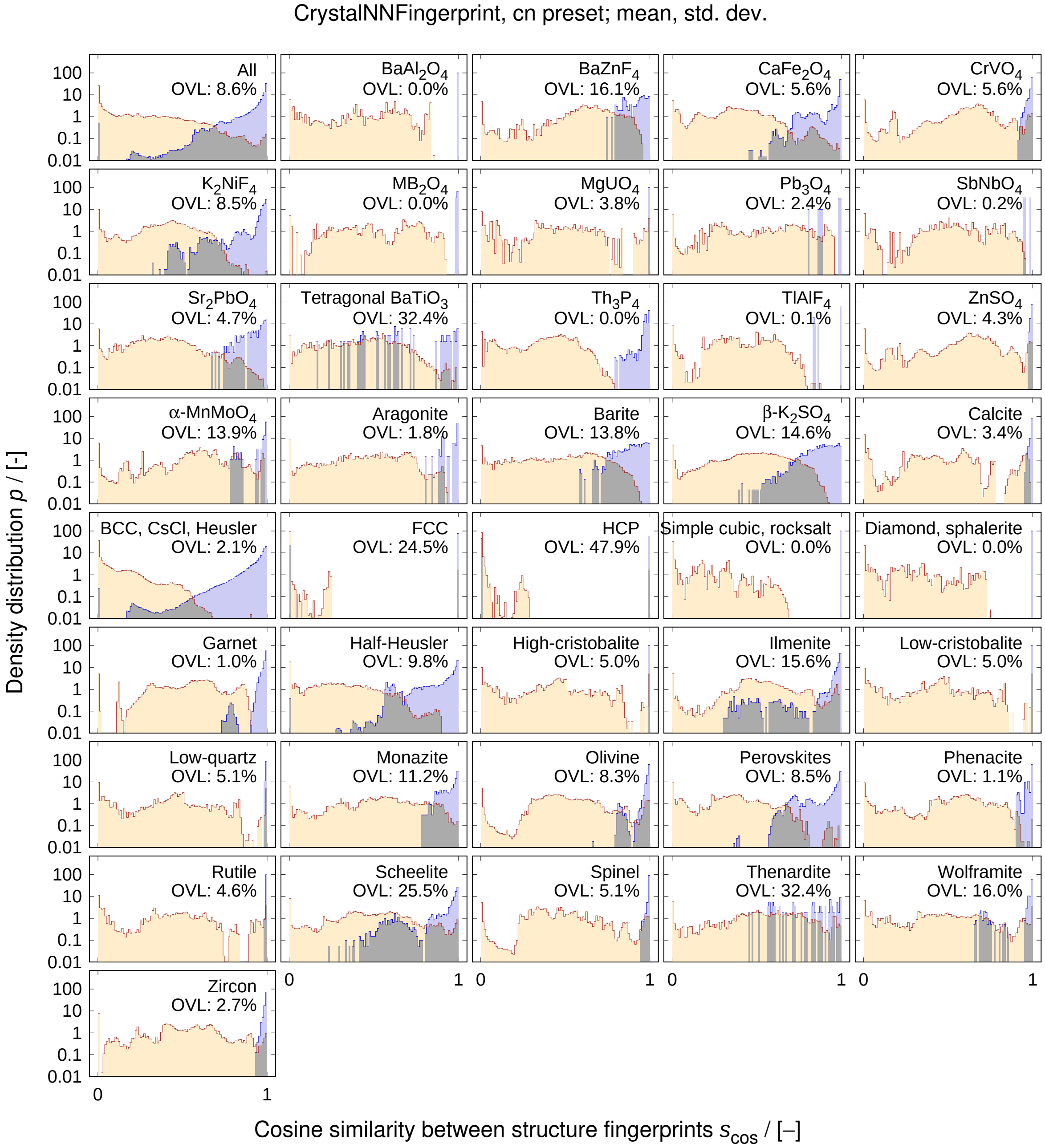

Fig. 4 Additional structure group (dis)similarity results.

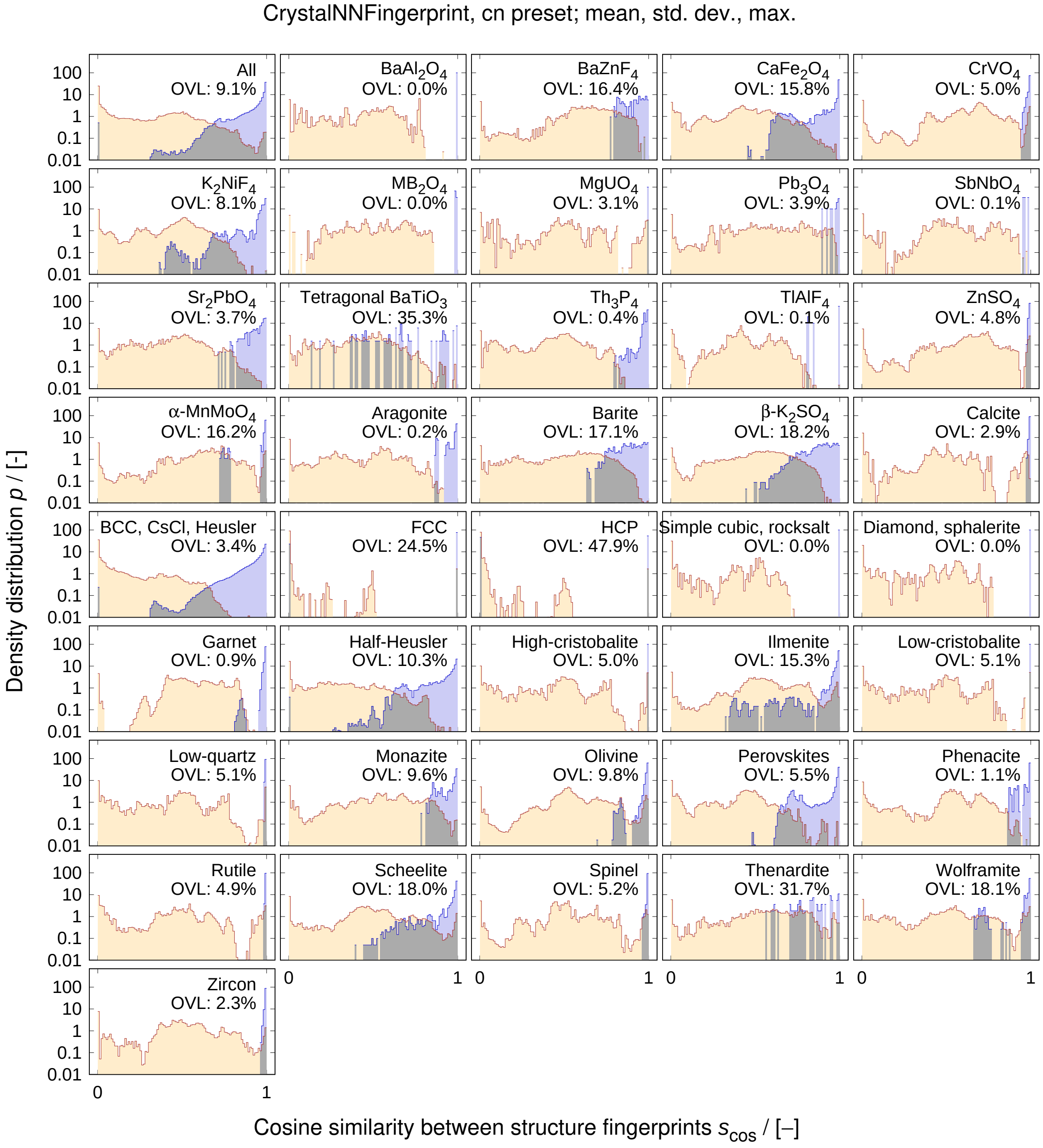

Fig. 5 Additional structure group (dis)similarity results.

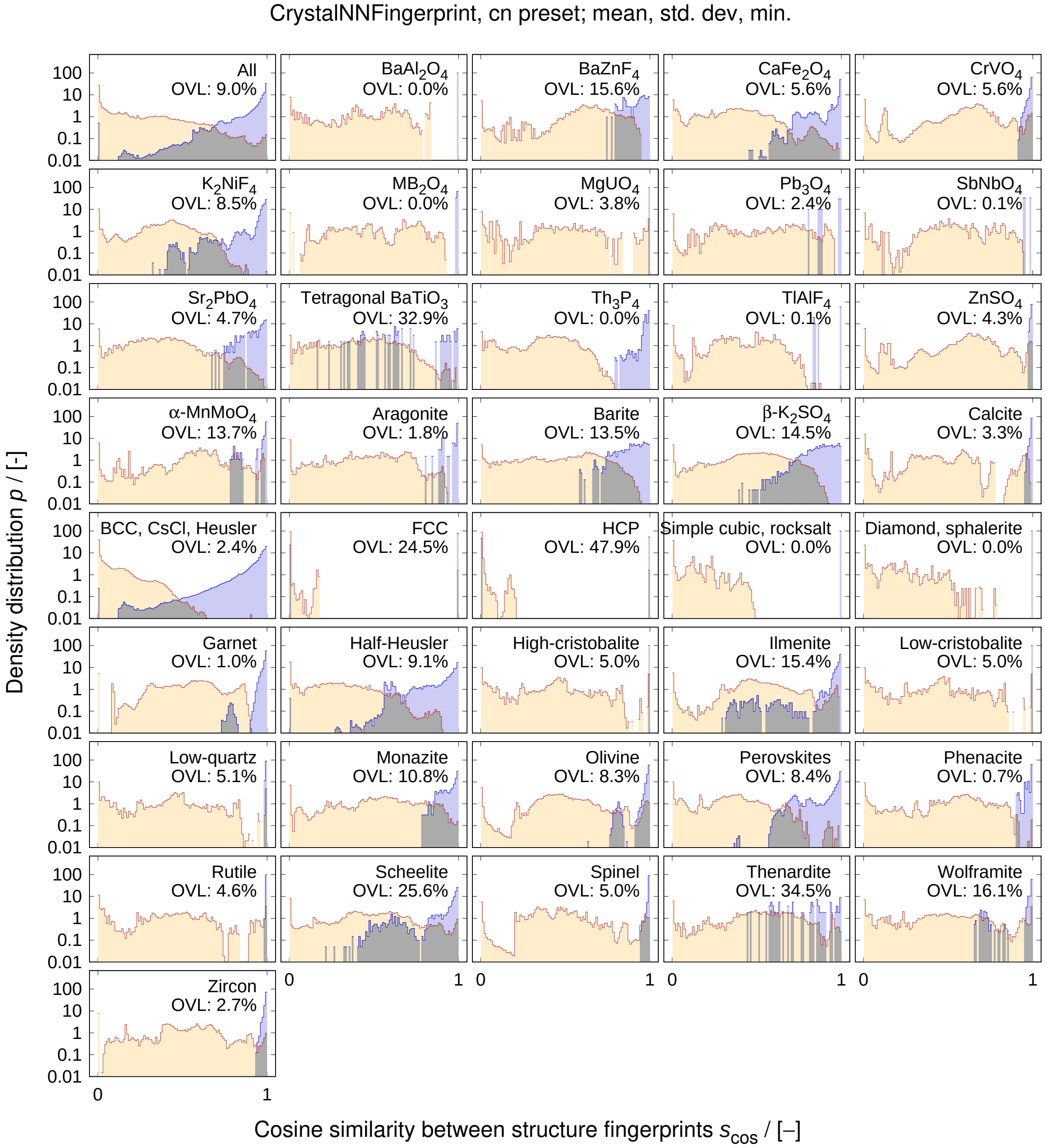

Fig. 6 Additional structure group (dis)similarity results.

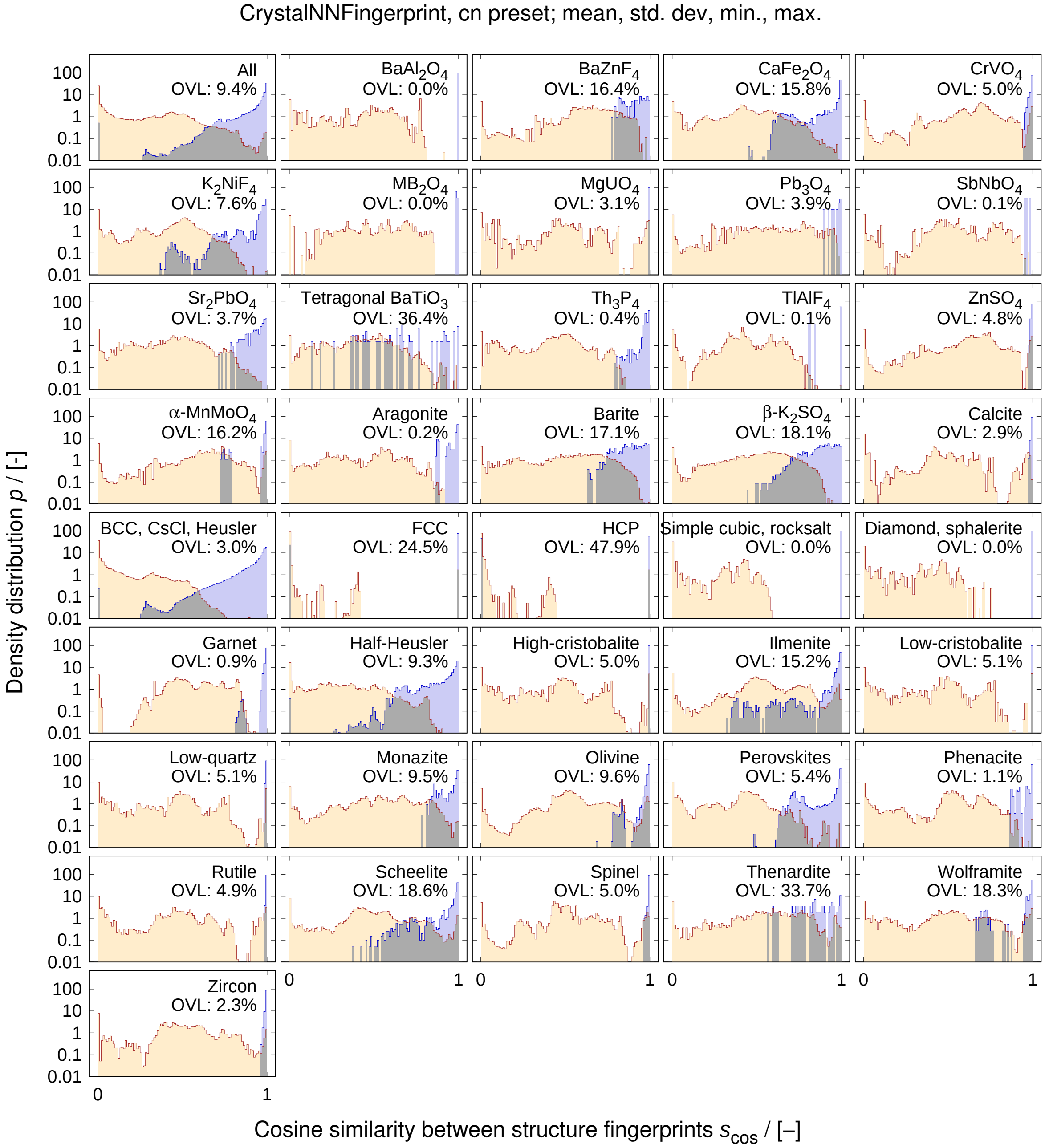

Fig. 7 Additional structure group (dis)similarity results.

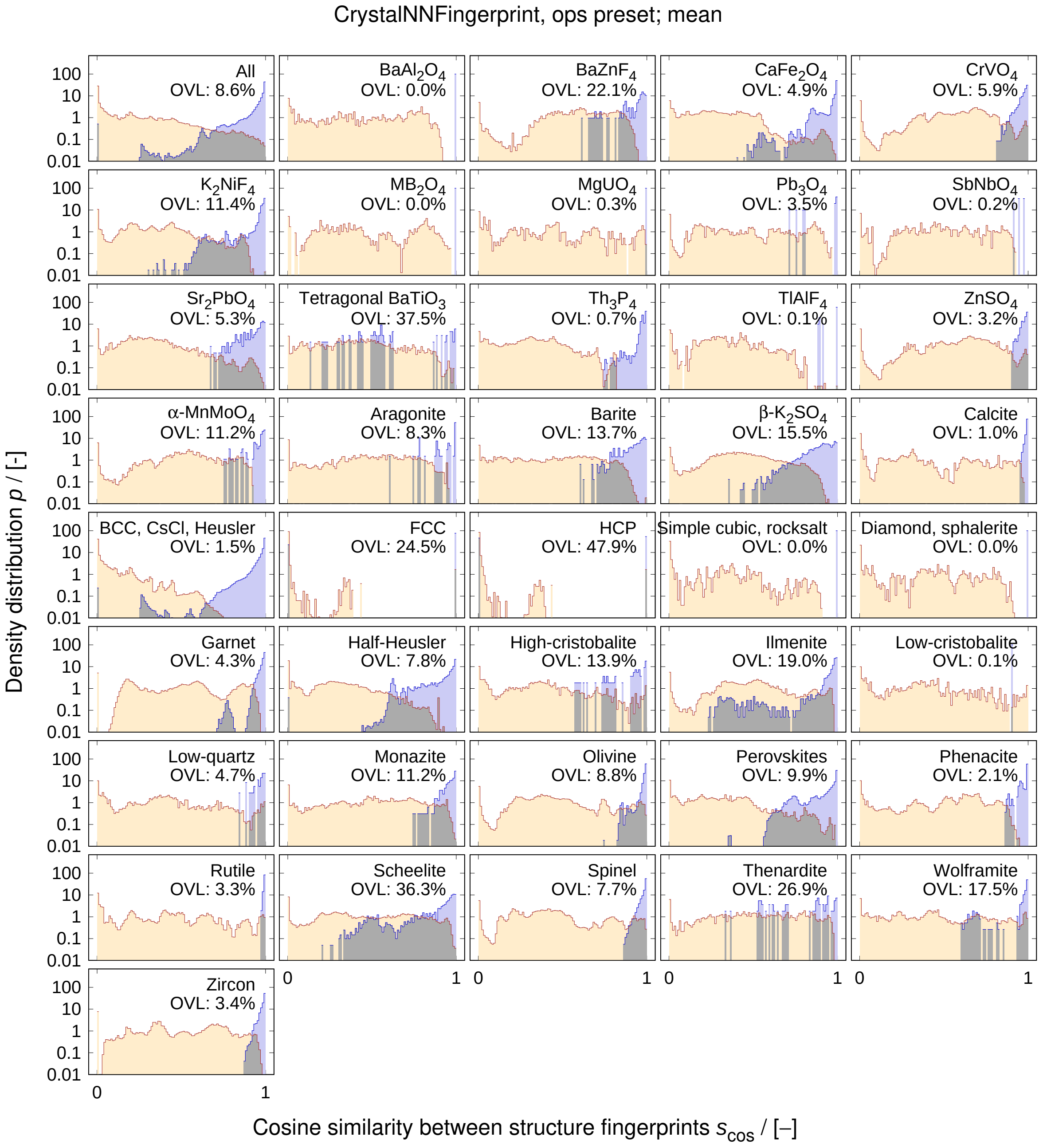

Fig. 8 Additional structure group (dis)similarity results.

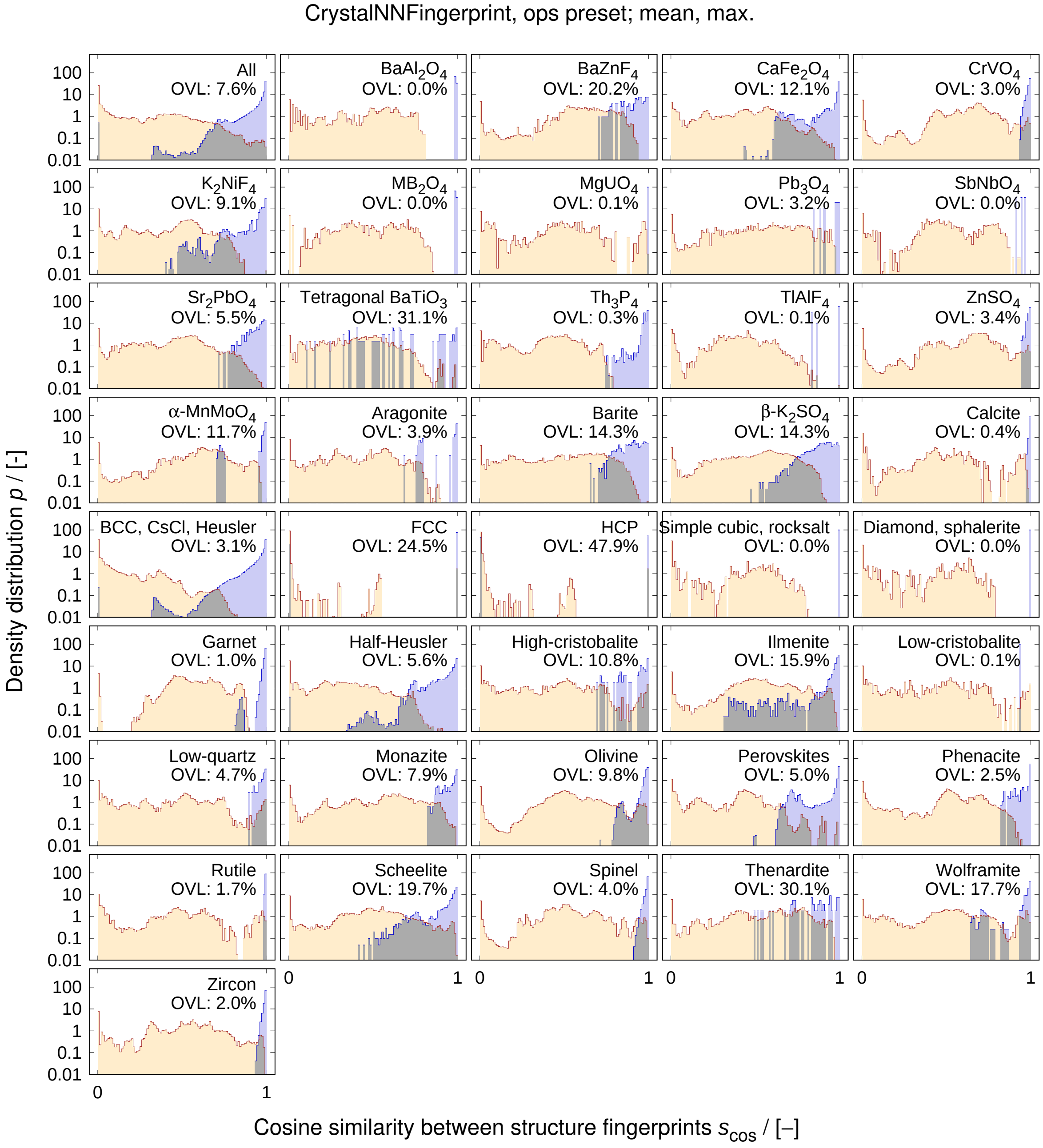

Fig. 9 Additional structure group (dis)similarity results.

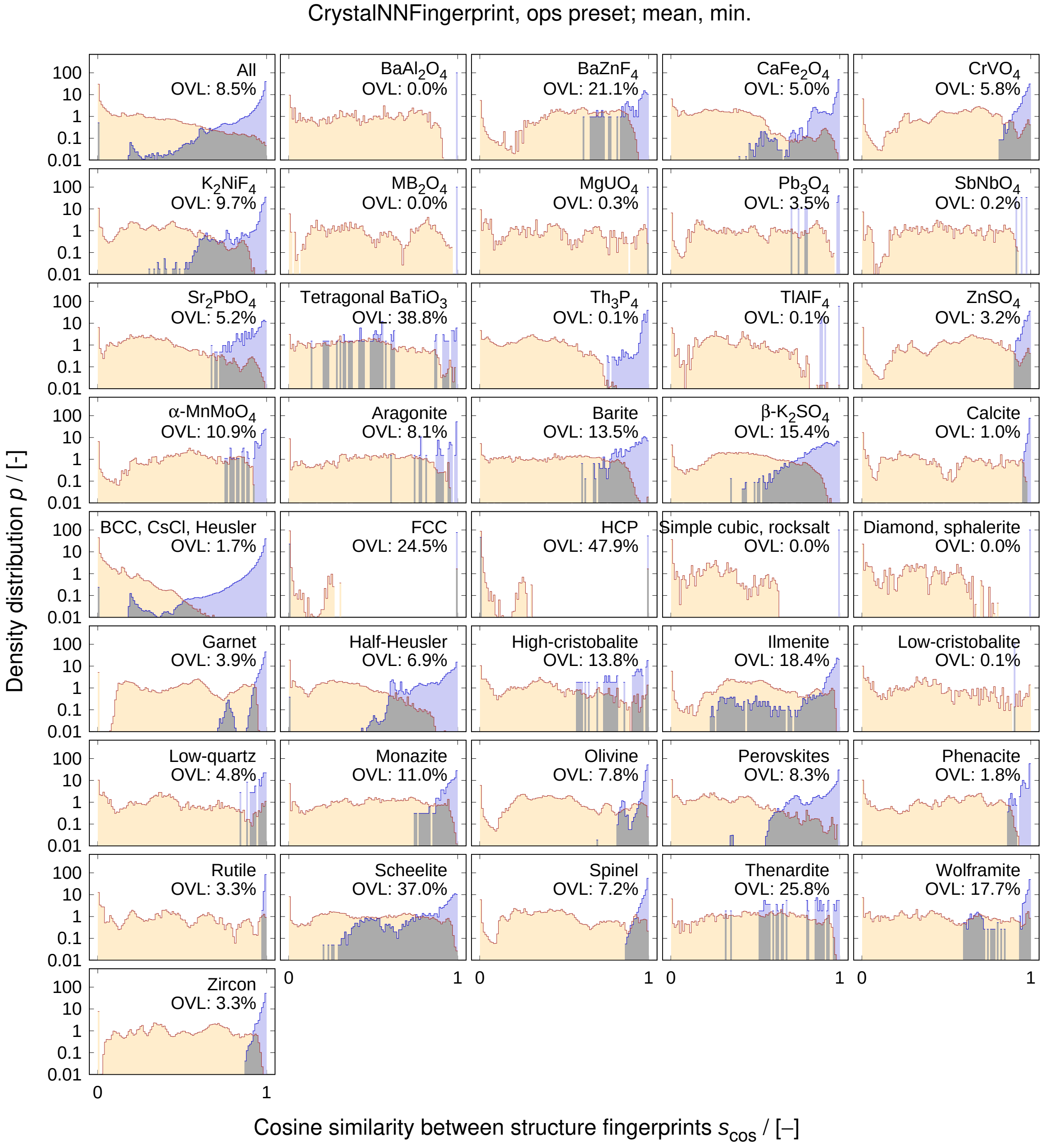

Fig. 10 Additional structure group (dis)similarity results.

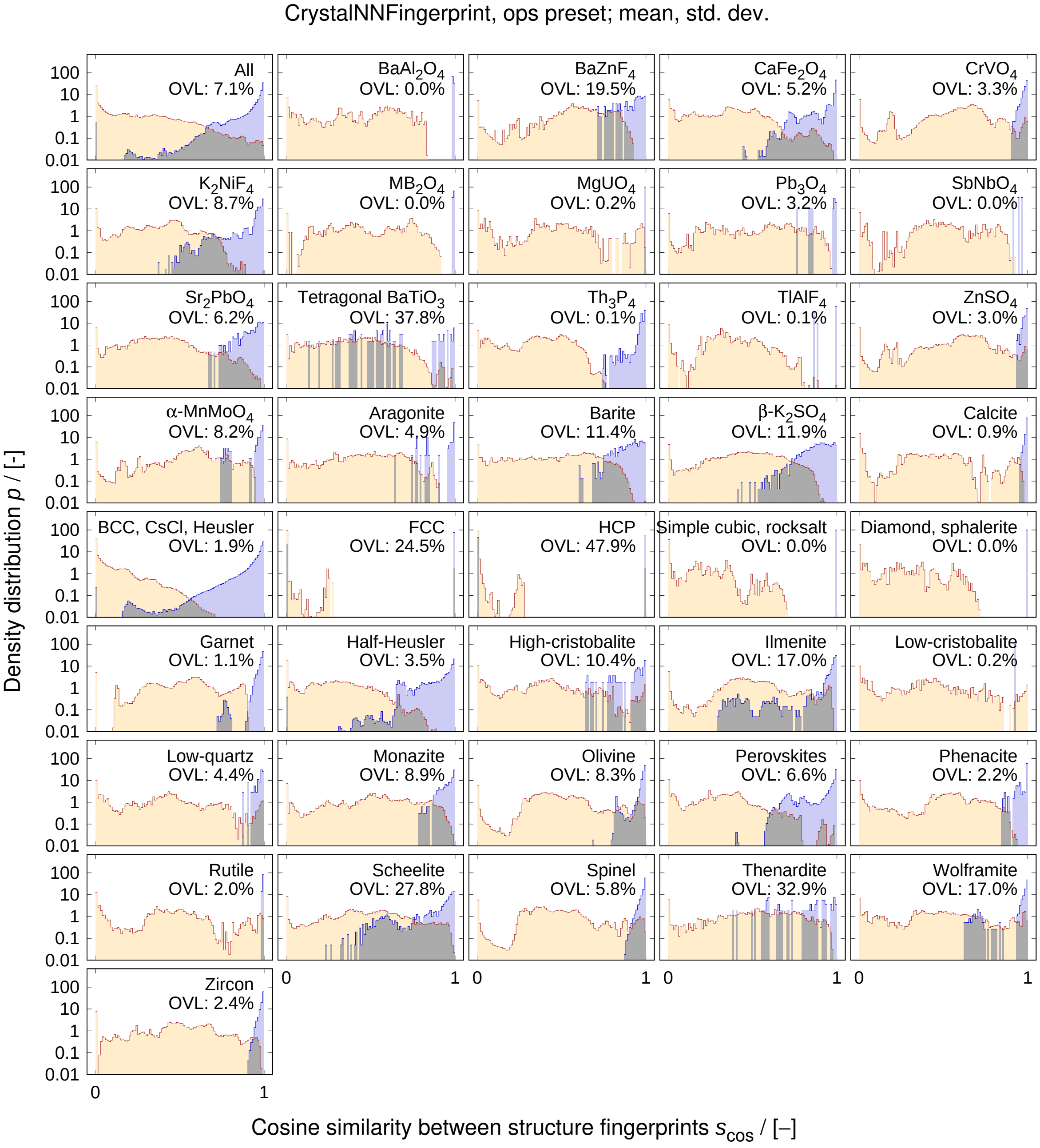

Fig. 11 Additional structure group (dis)similarity results.

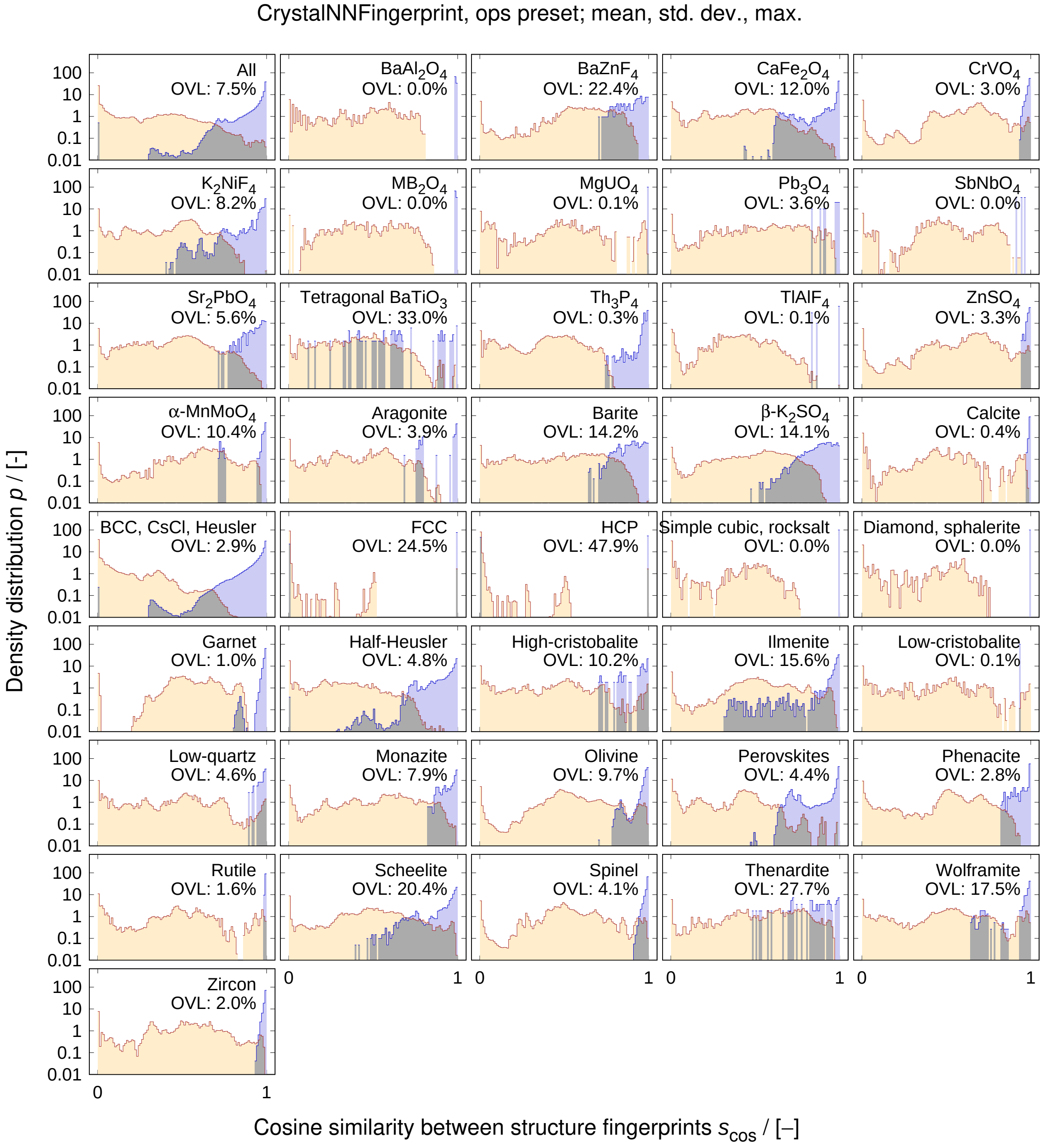

Fig. 12 Additional structure group (dis)similarity results.

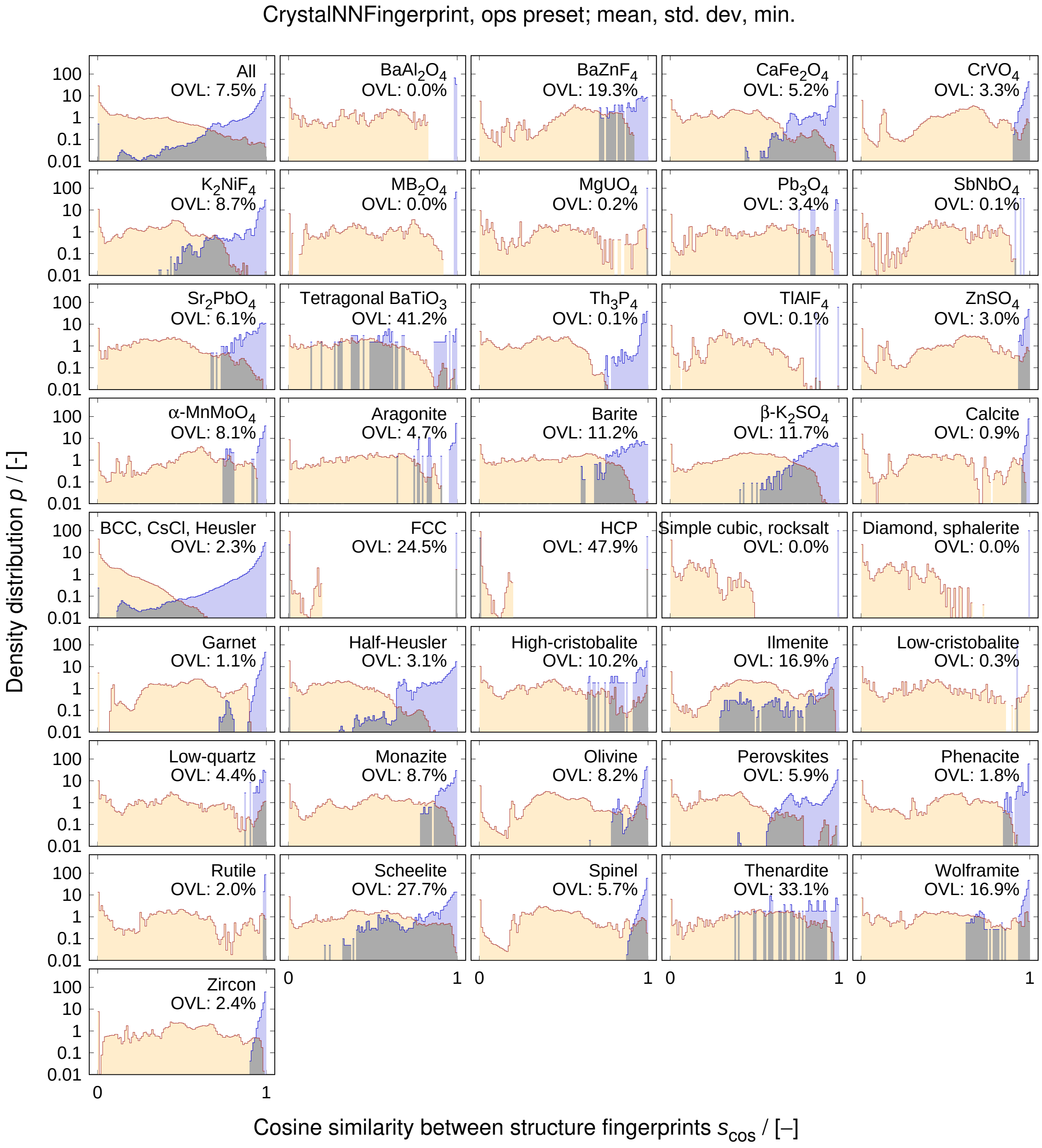

Fig. 13 Additional structure group (dis)similarity results.

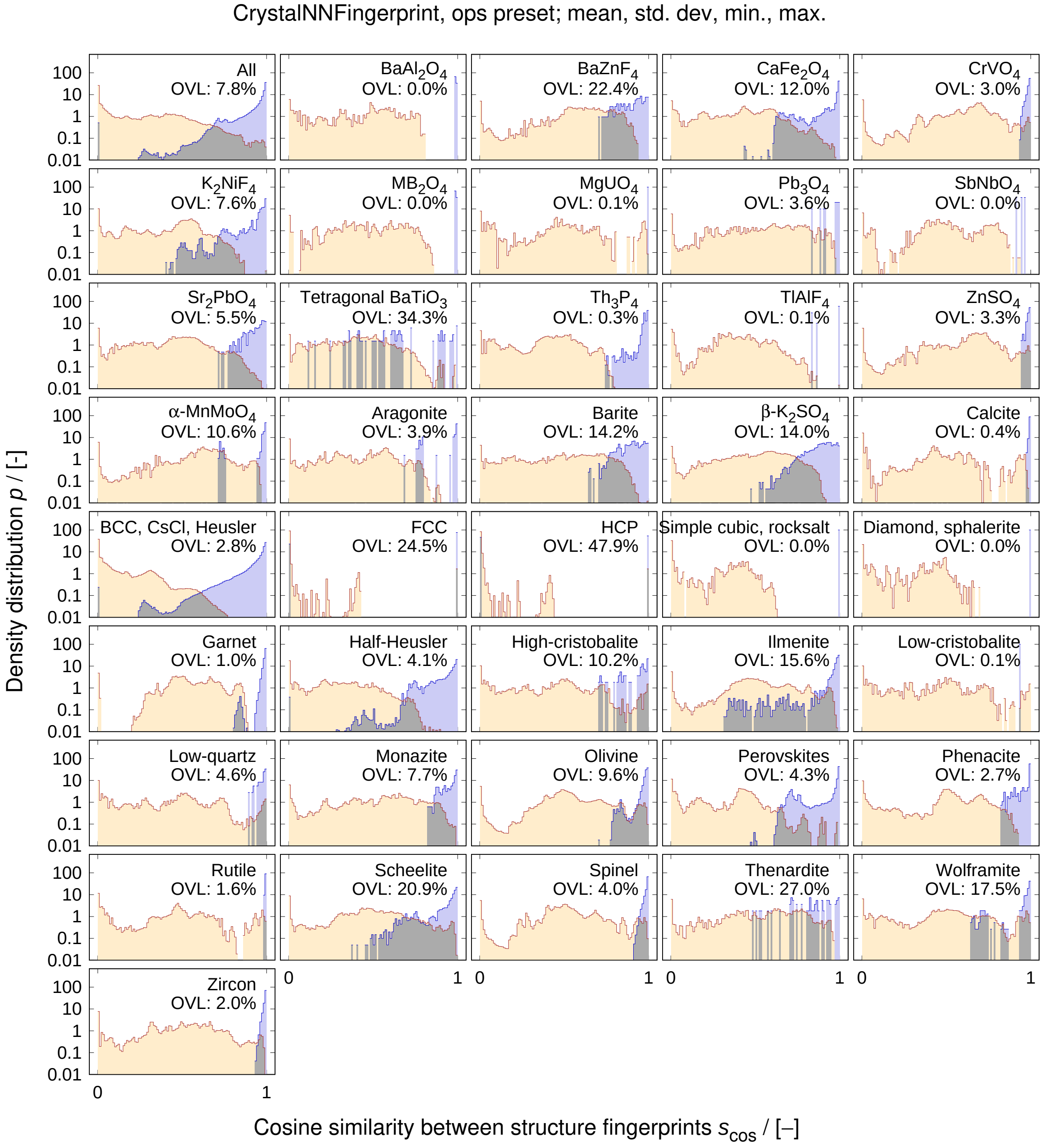

Fig. 14 Additional structure group (dis)similarity results.

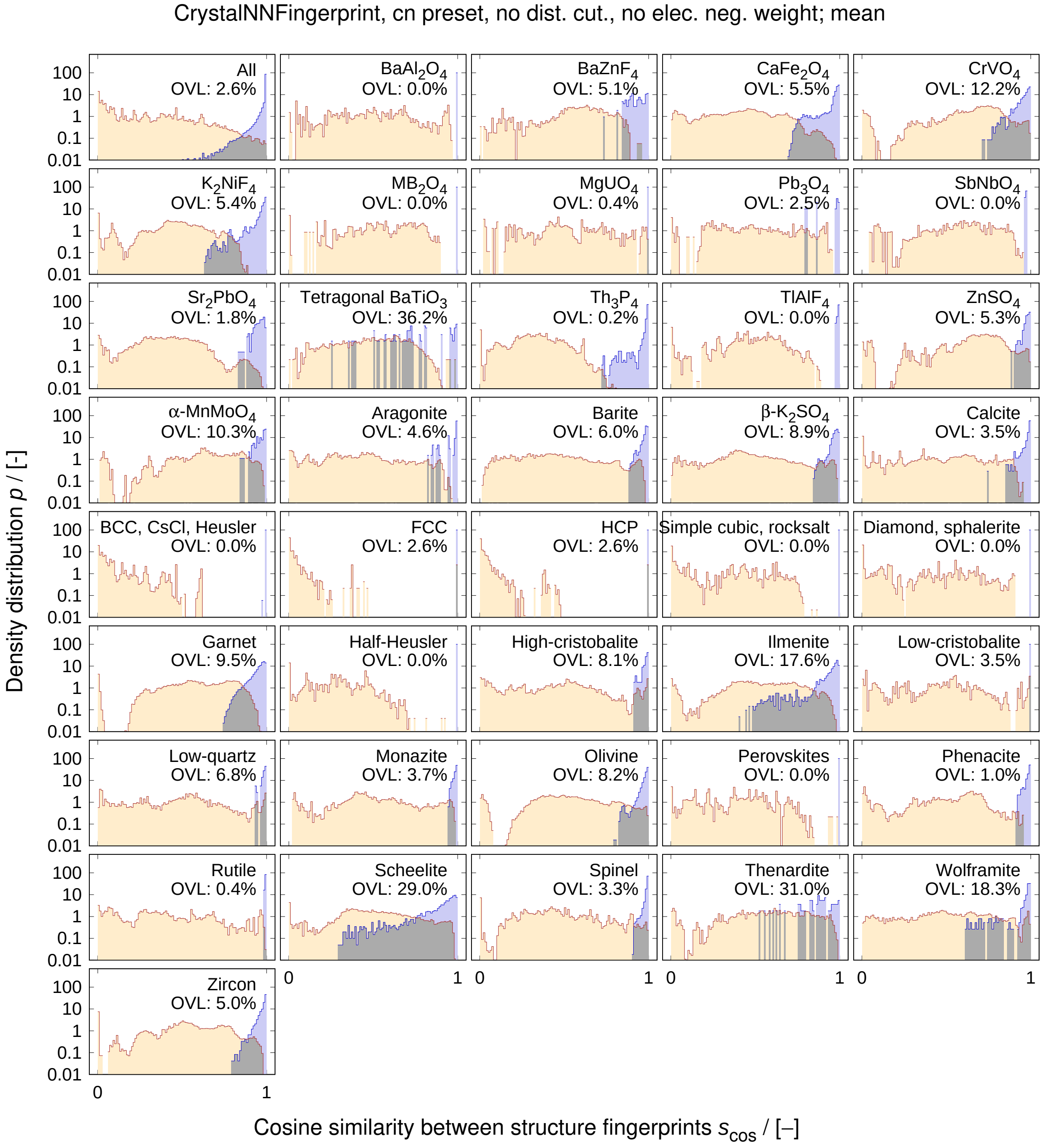

Fig. 15 Additional structure group (dis)similarity results.

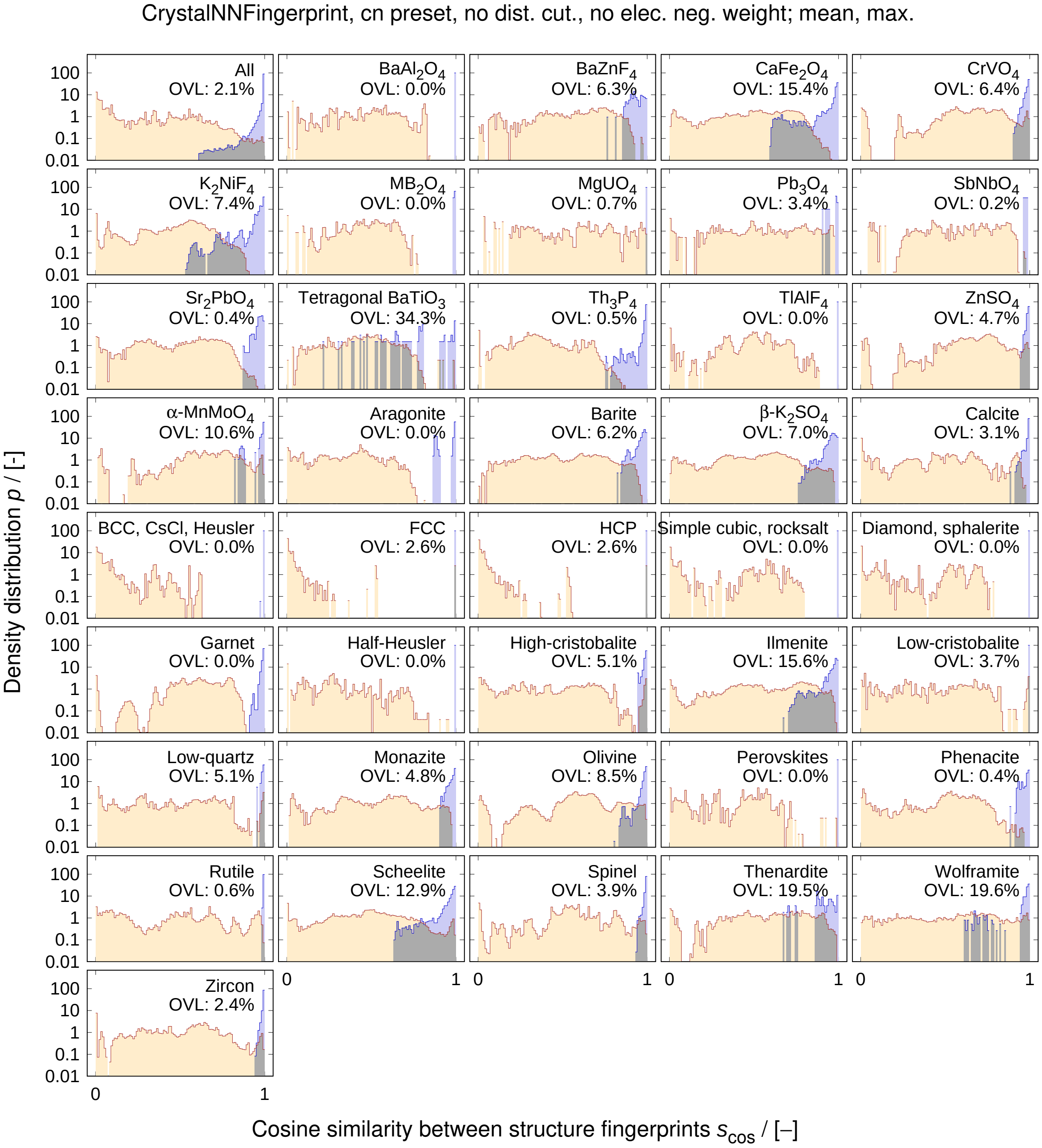

Fig. 16 Additional structure group (dis)similarity results.

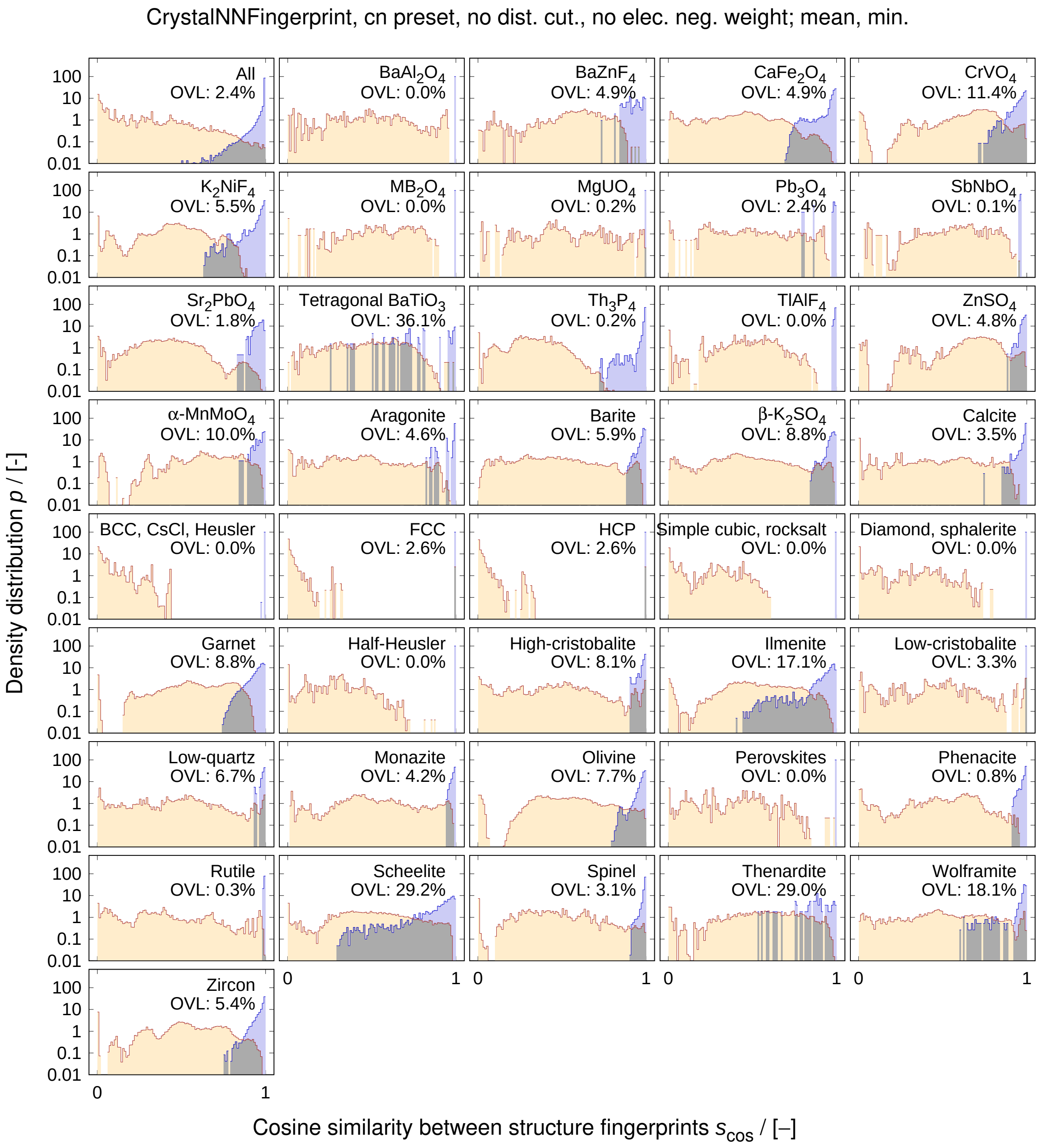

Fig. 17 Additional structure group (dis)similarity results.

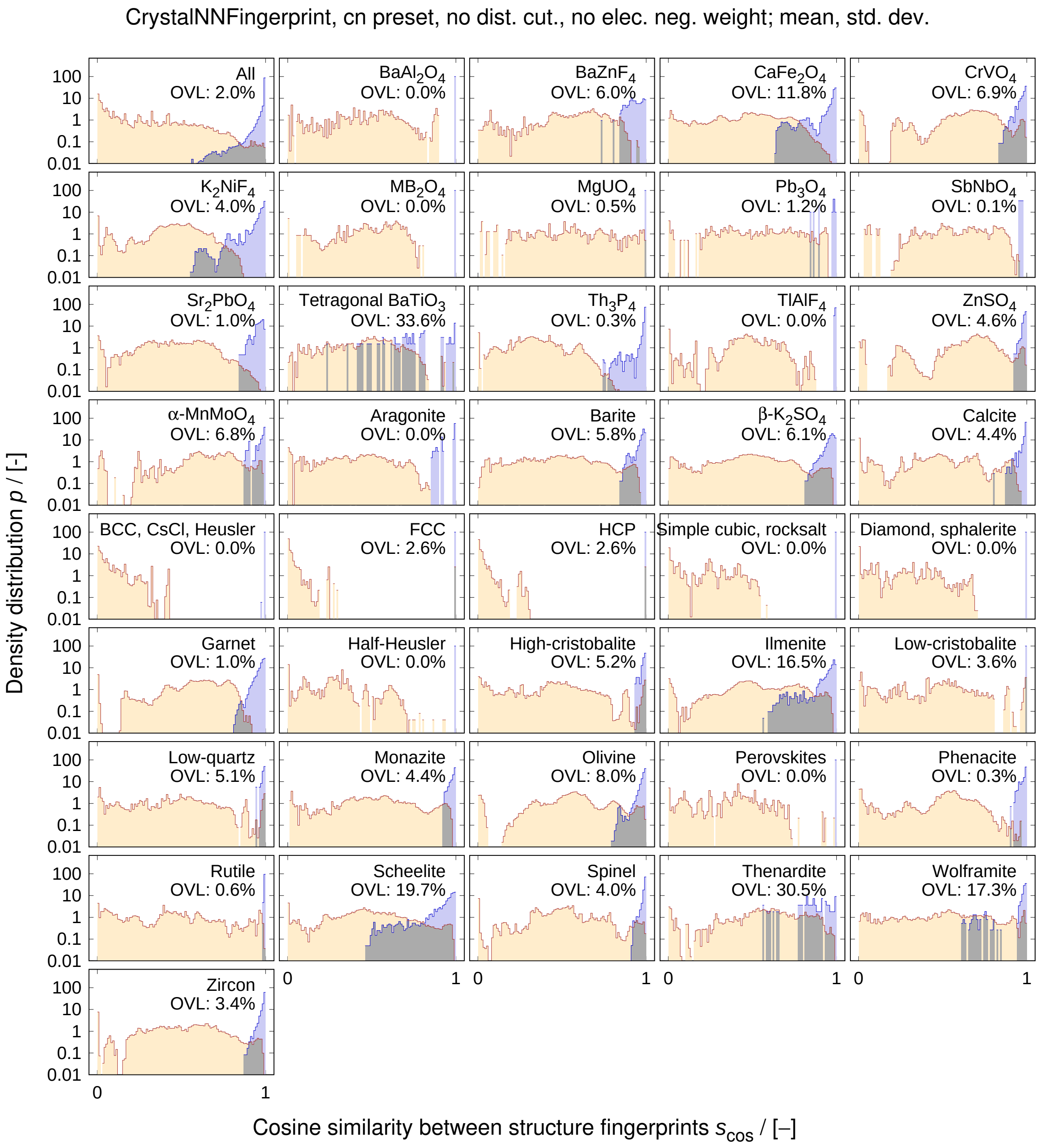

Fig. 18 Additional structure group (dis)similarity results.

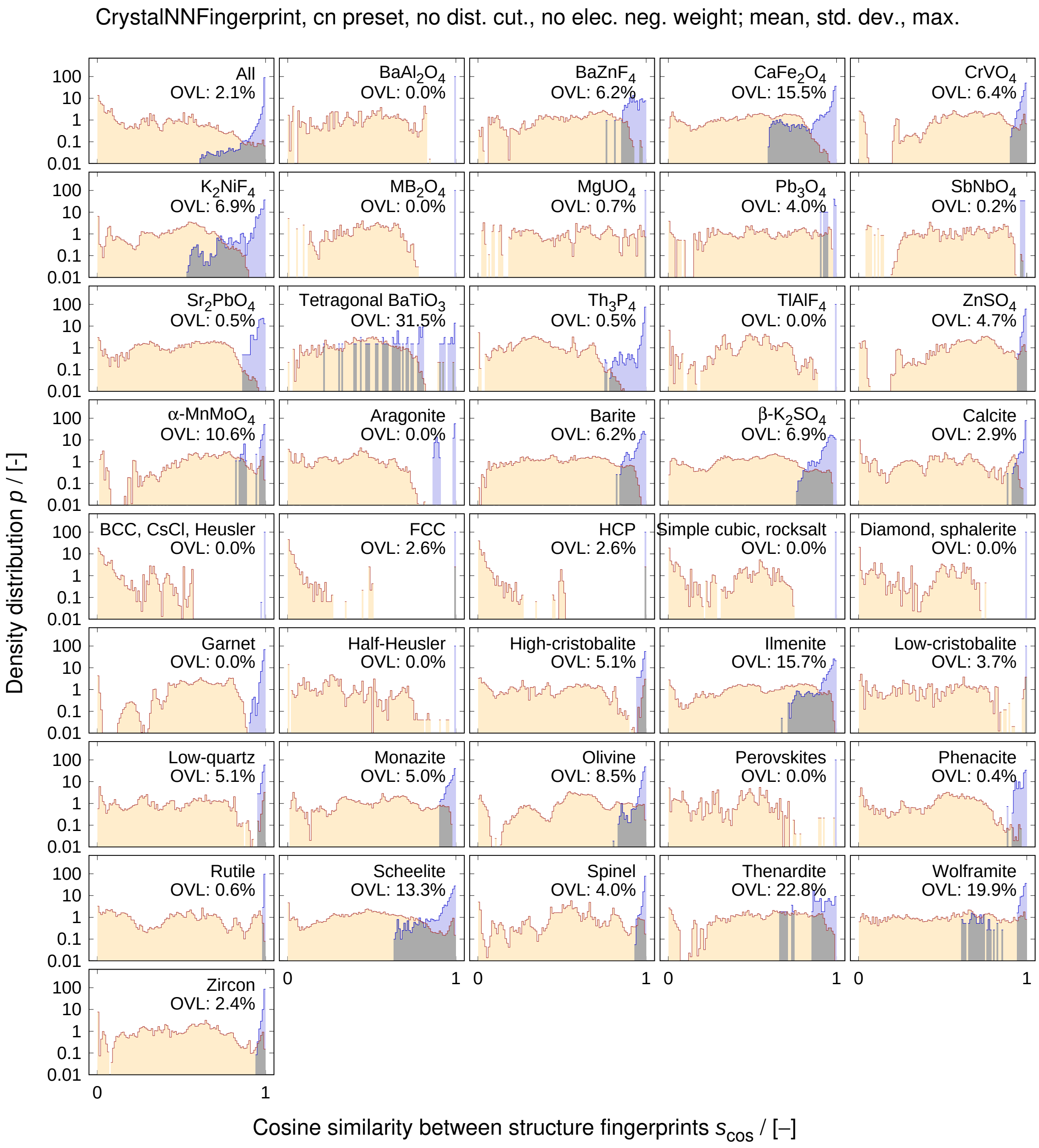

Fig. 19 Additional structure group (dis)similarity results.

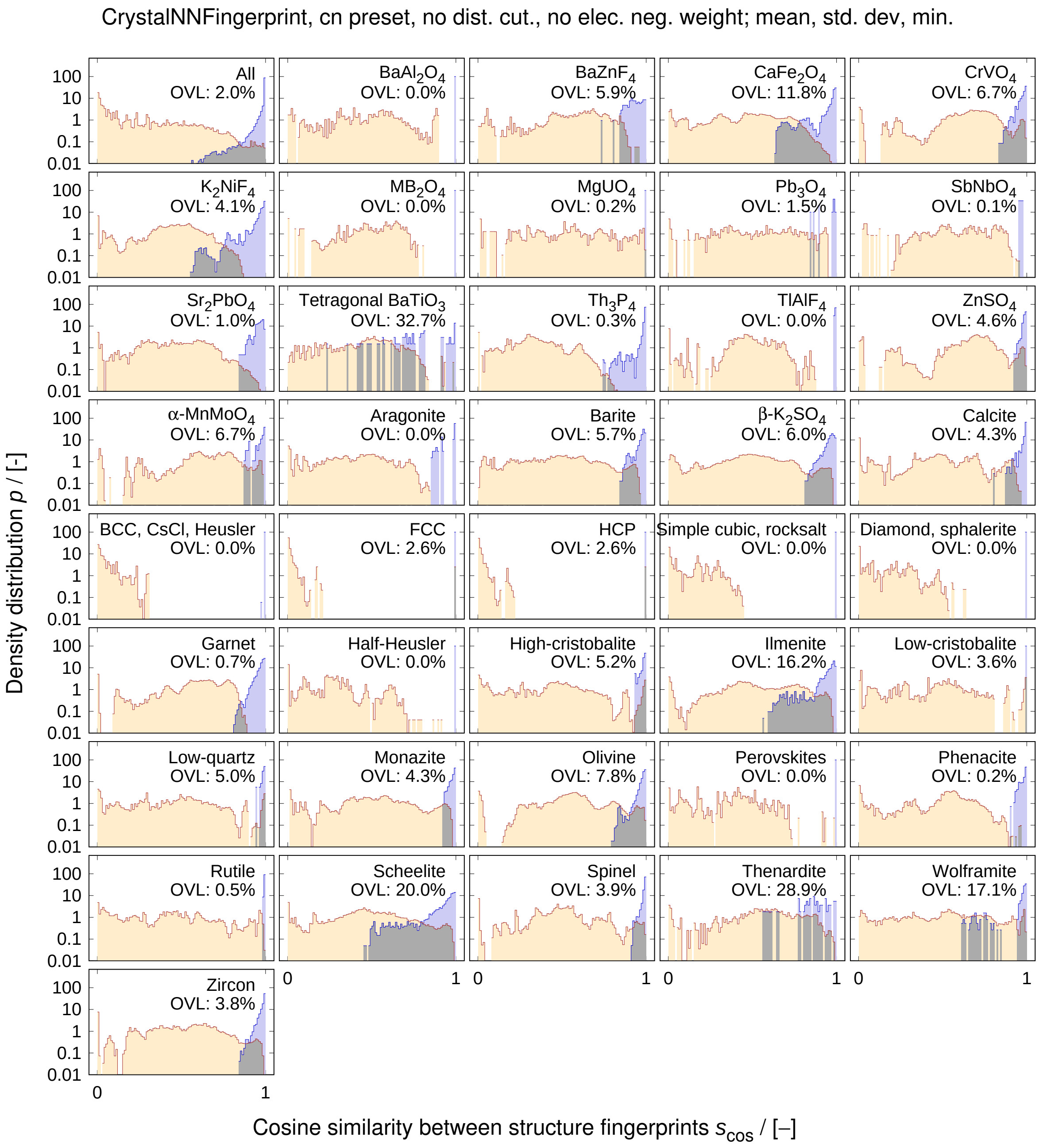

Fig. 20 Additional structure group (dis)similarity results.

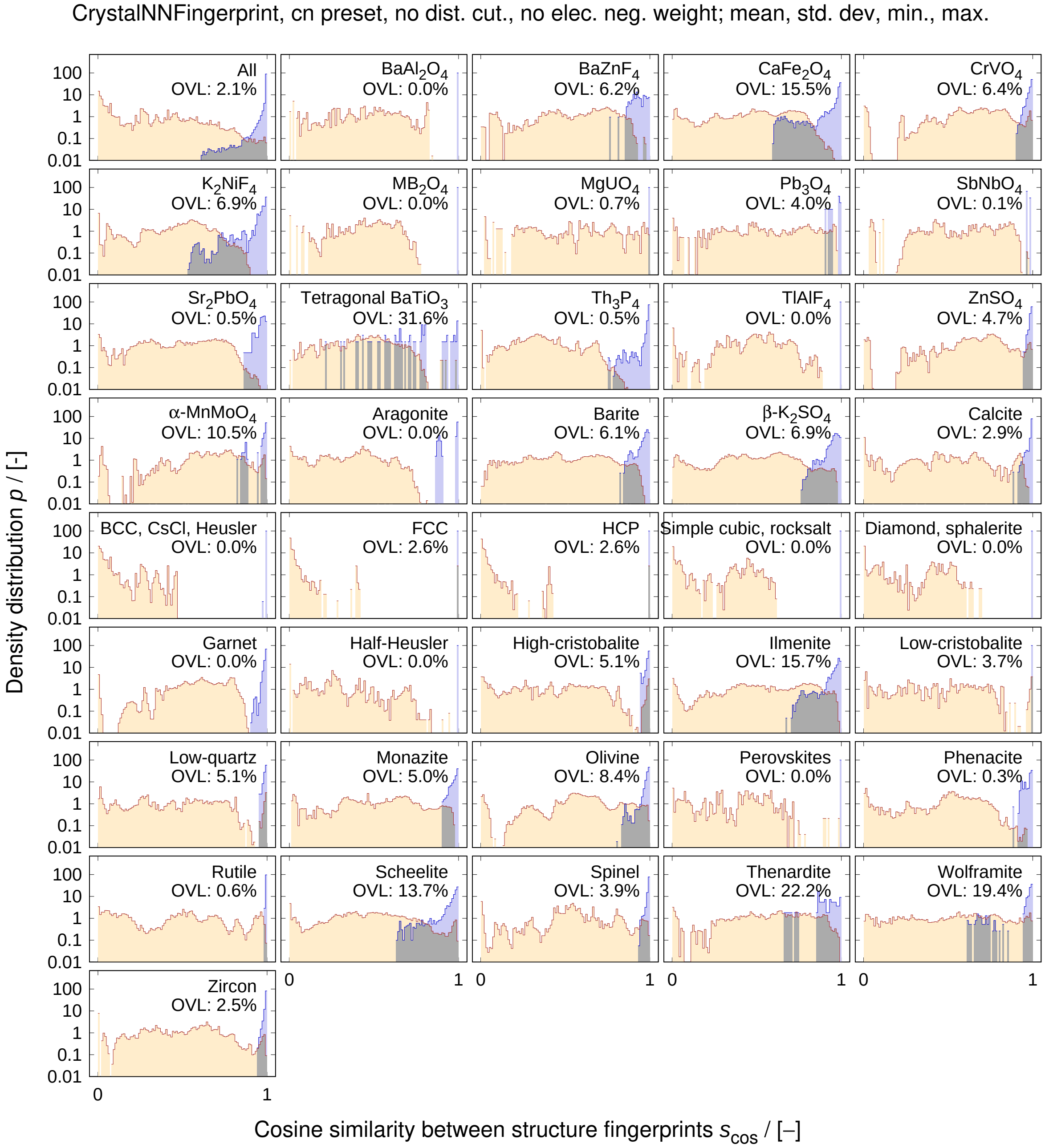

Fig. 21 Additional structure group (dis)similarity results.

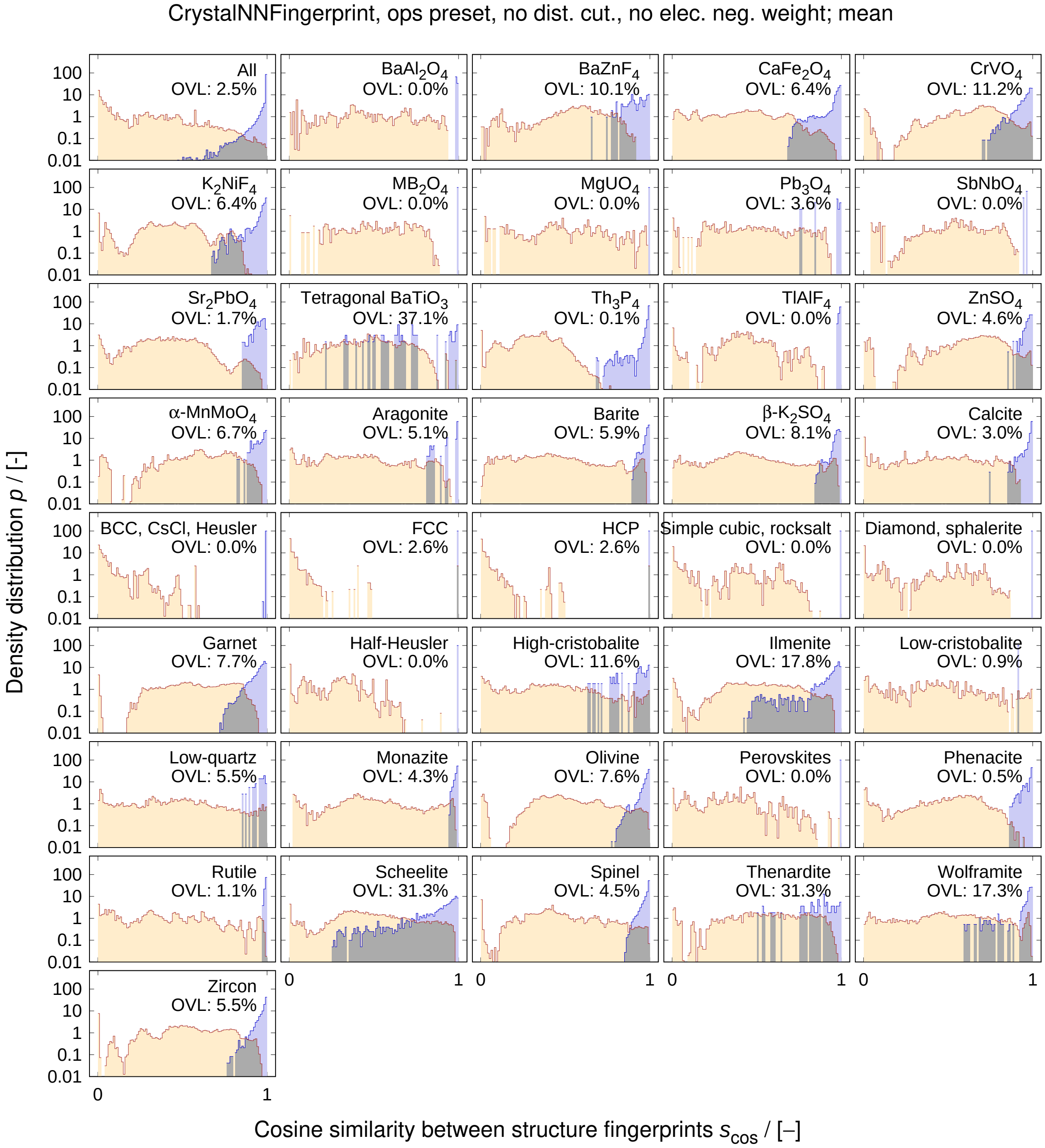

Fig. 22 Additional structure group (dis)similarity results.

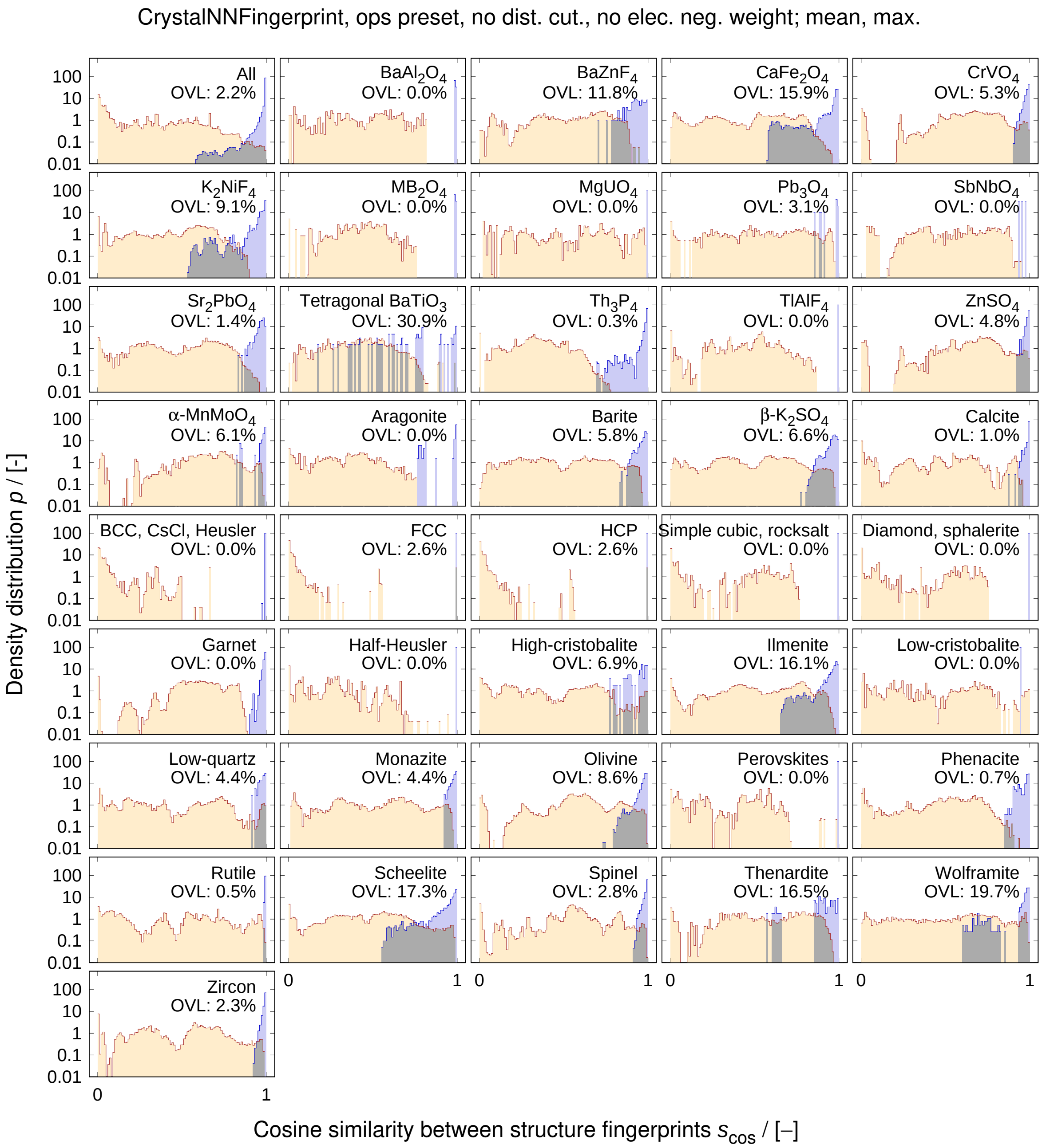

Fig. 23 Additional structure group (dis)similarity results.

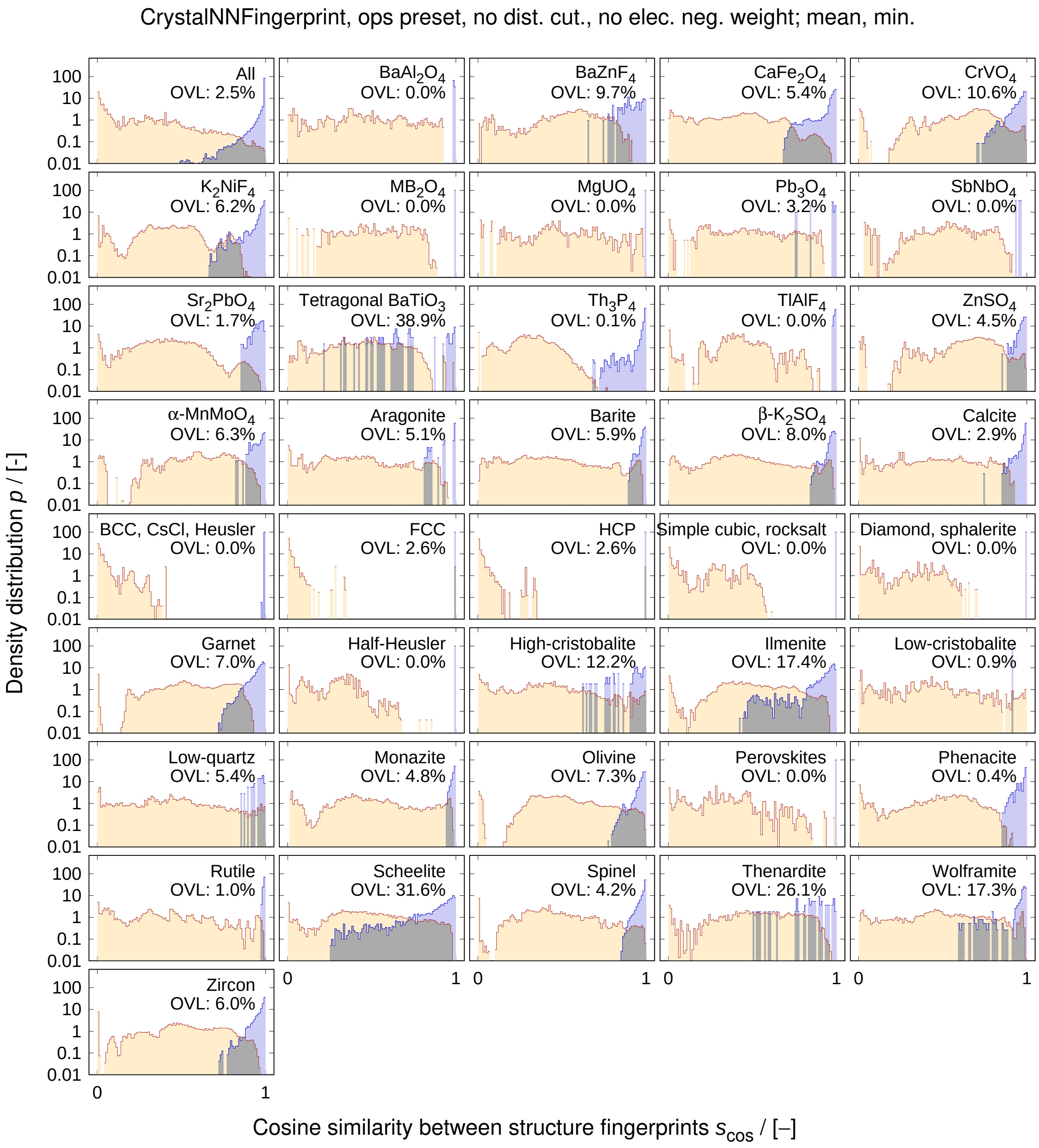

Fig. 24 Additional structure group (dis)similarity results.

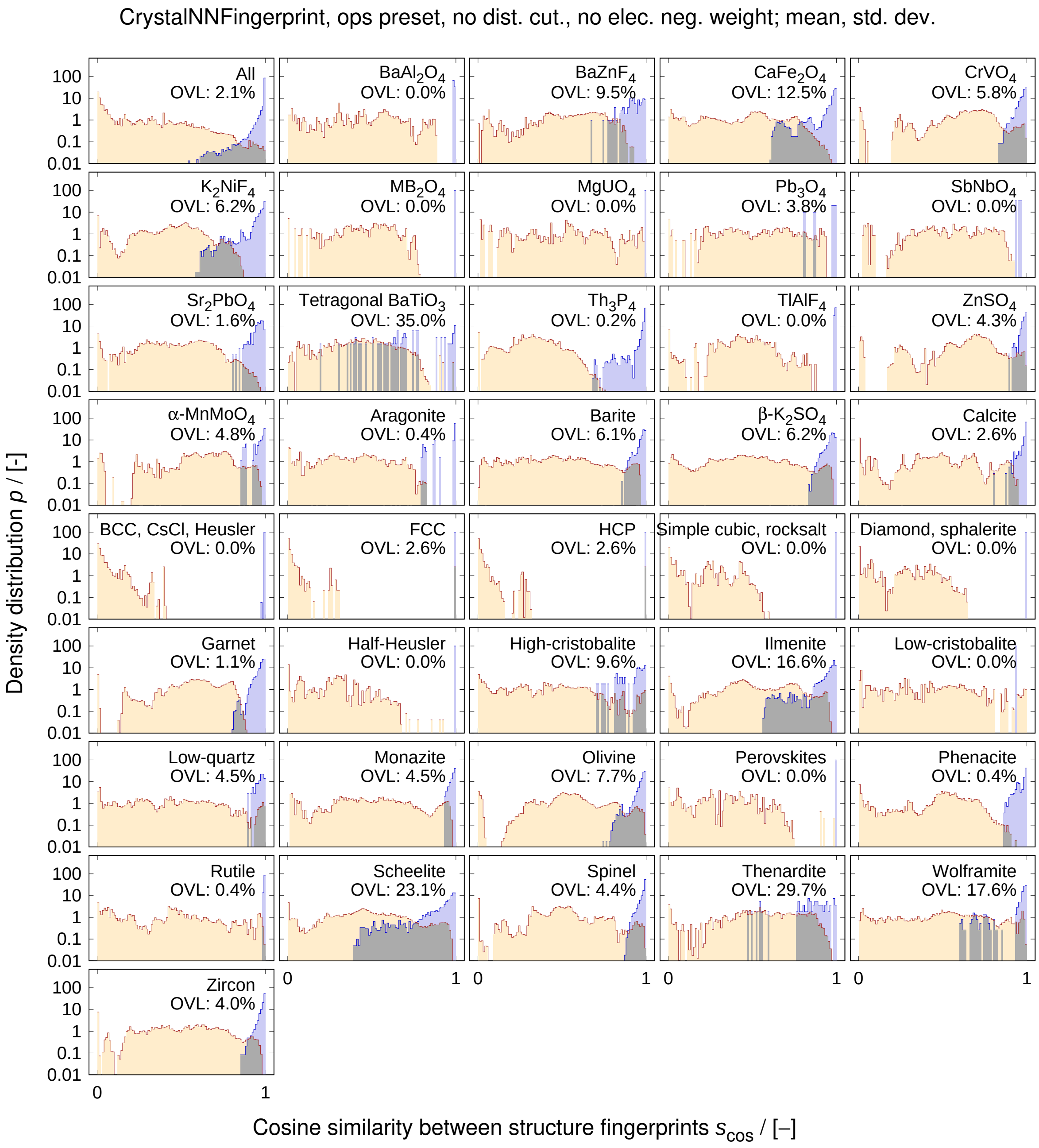

Fig. 25 Additional structure group (dis)similarity results.

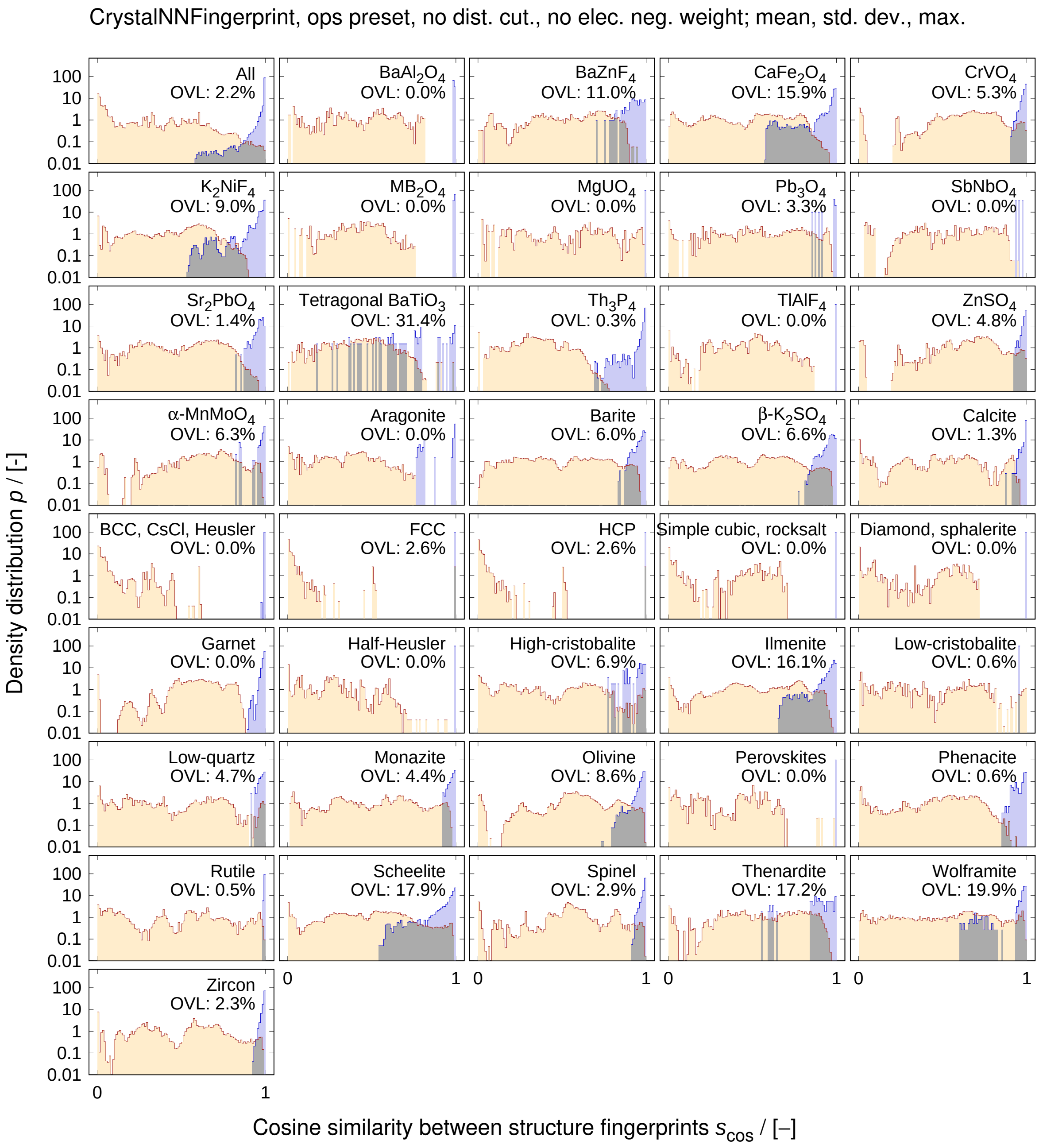

Fig. 26 Additional structure group (dis)similarity results.

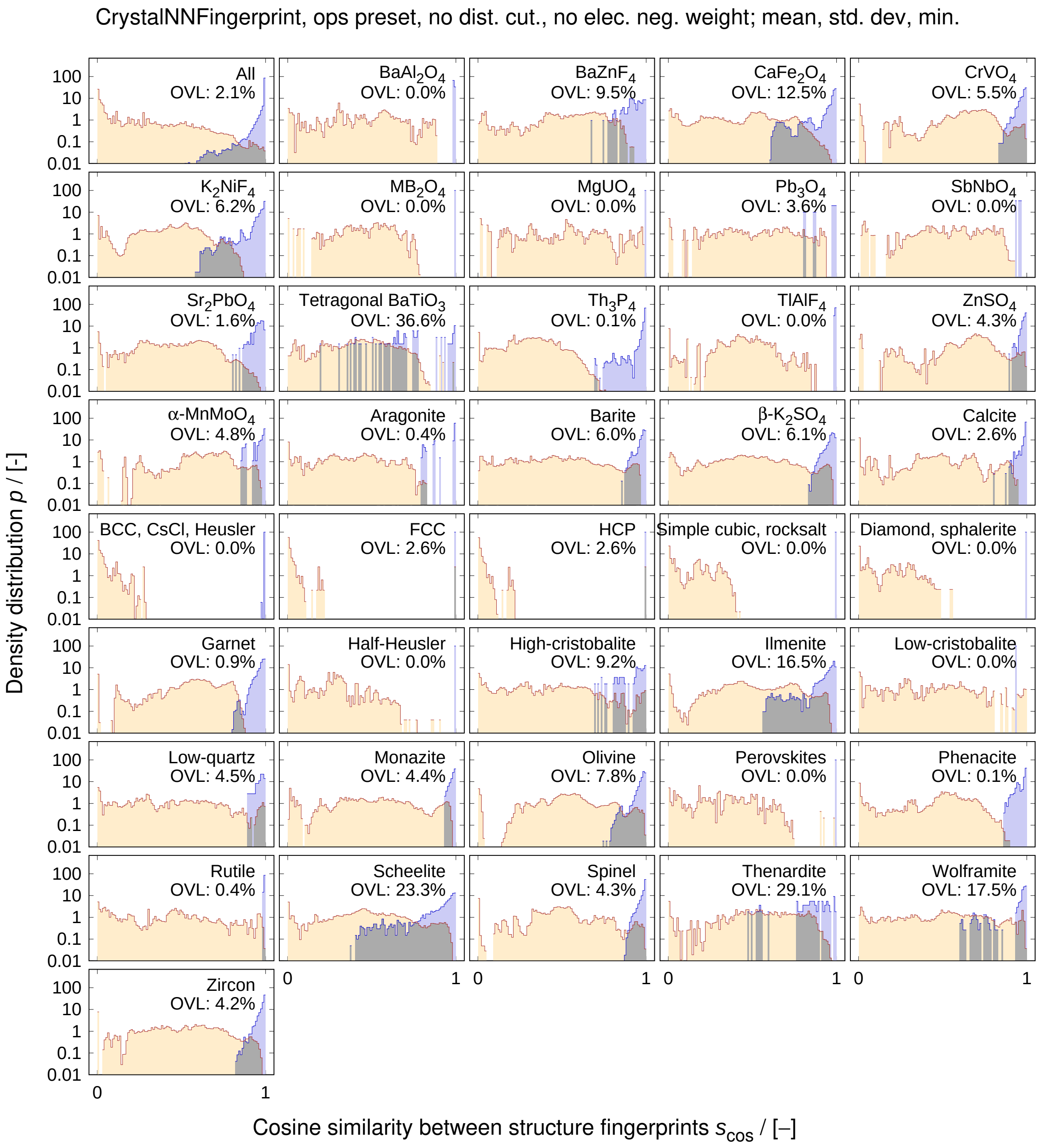

Fig. 27 Additional structure group (dis)similarity results.

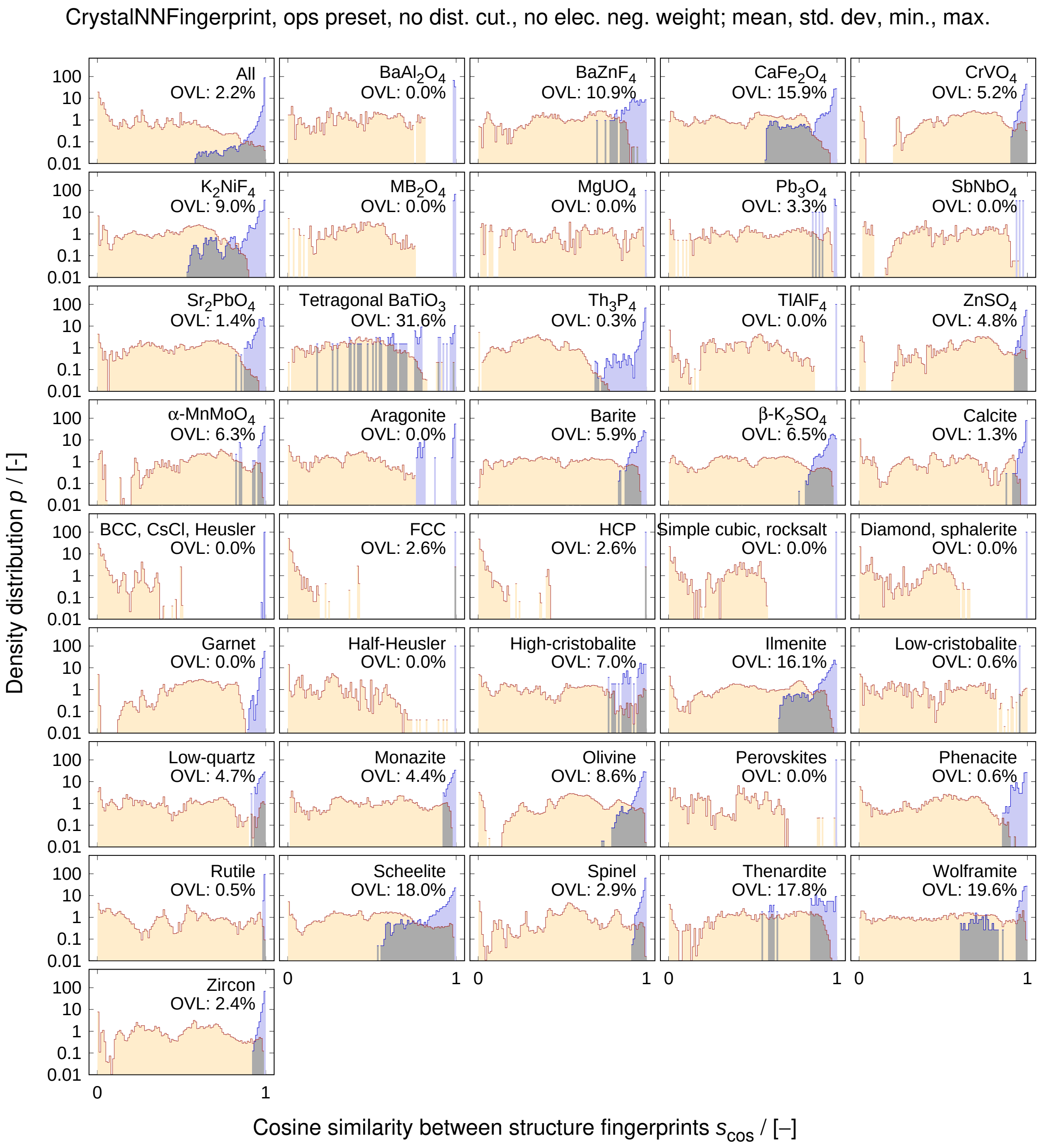

Fig. 28 Additional structure group (dis)similarity results.

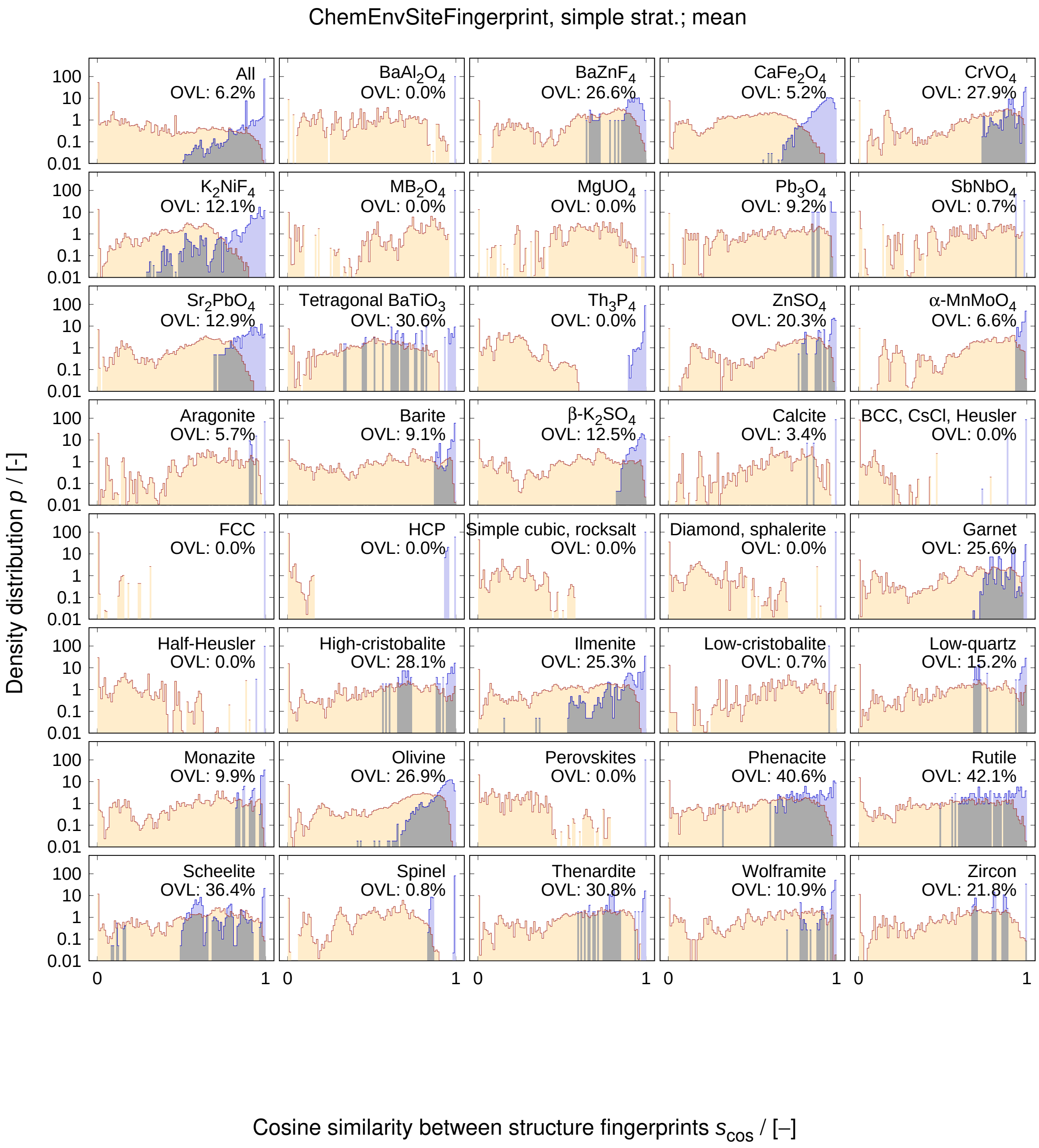

Fig. 29 Additional structure group (dis)similarity results.

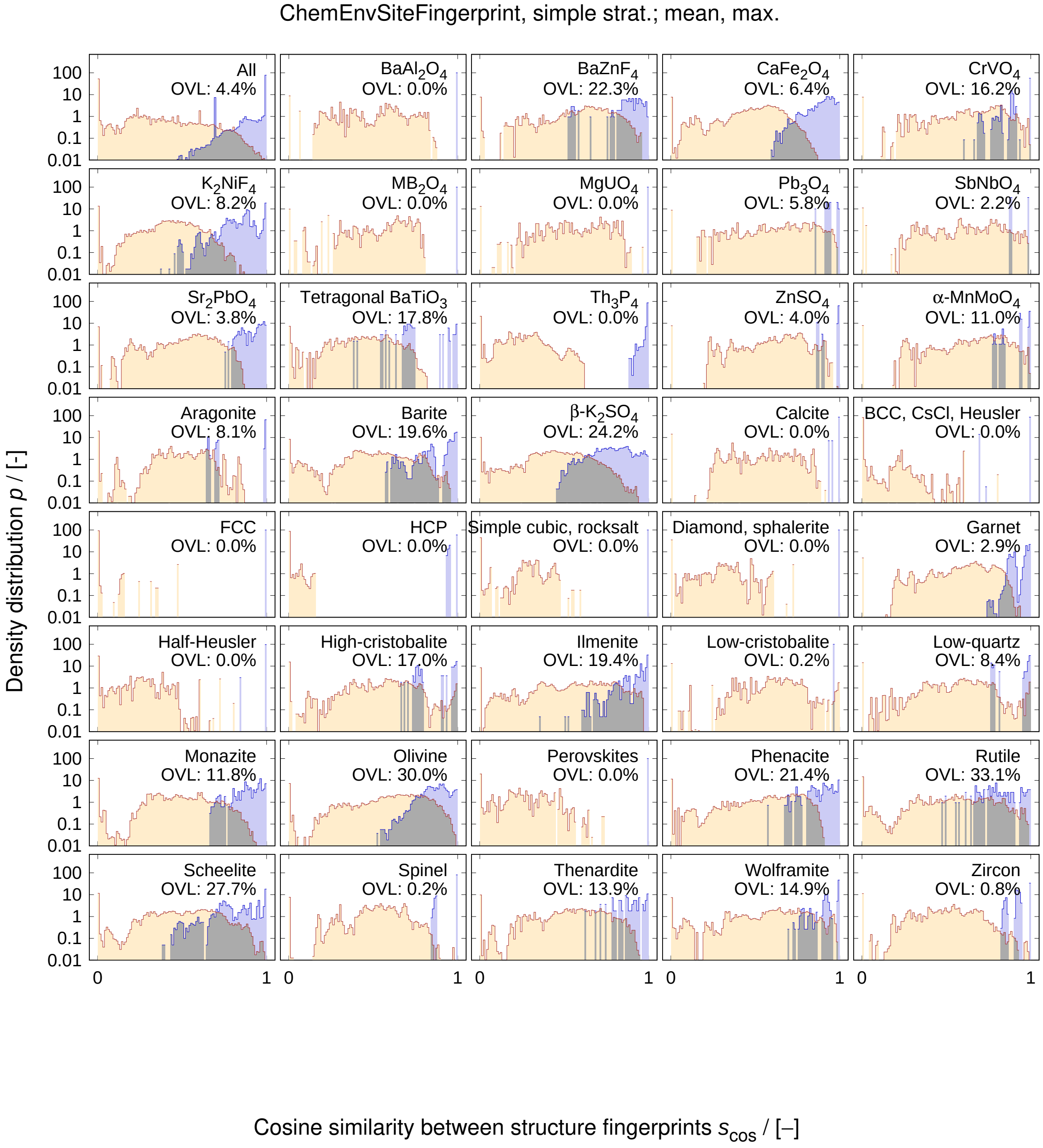

Fig. 30 Additional structure group (dis)similarity results.

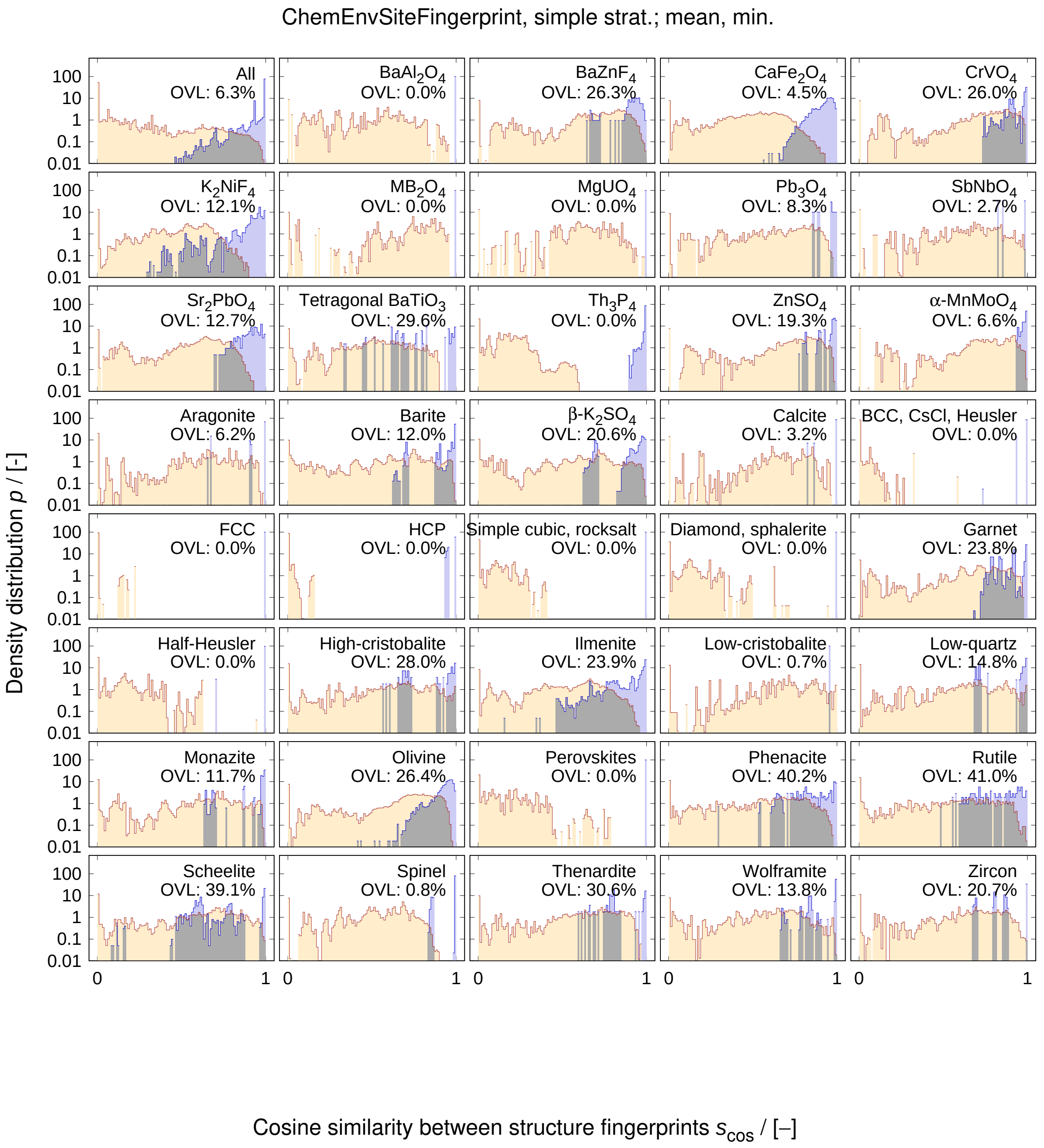

Fig. 31 Additional structure group (dis)similarity results.

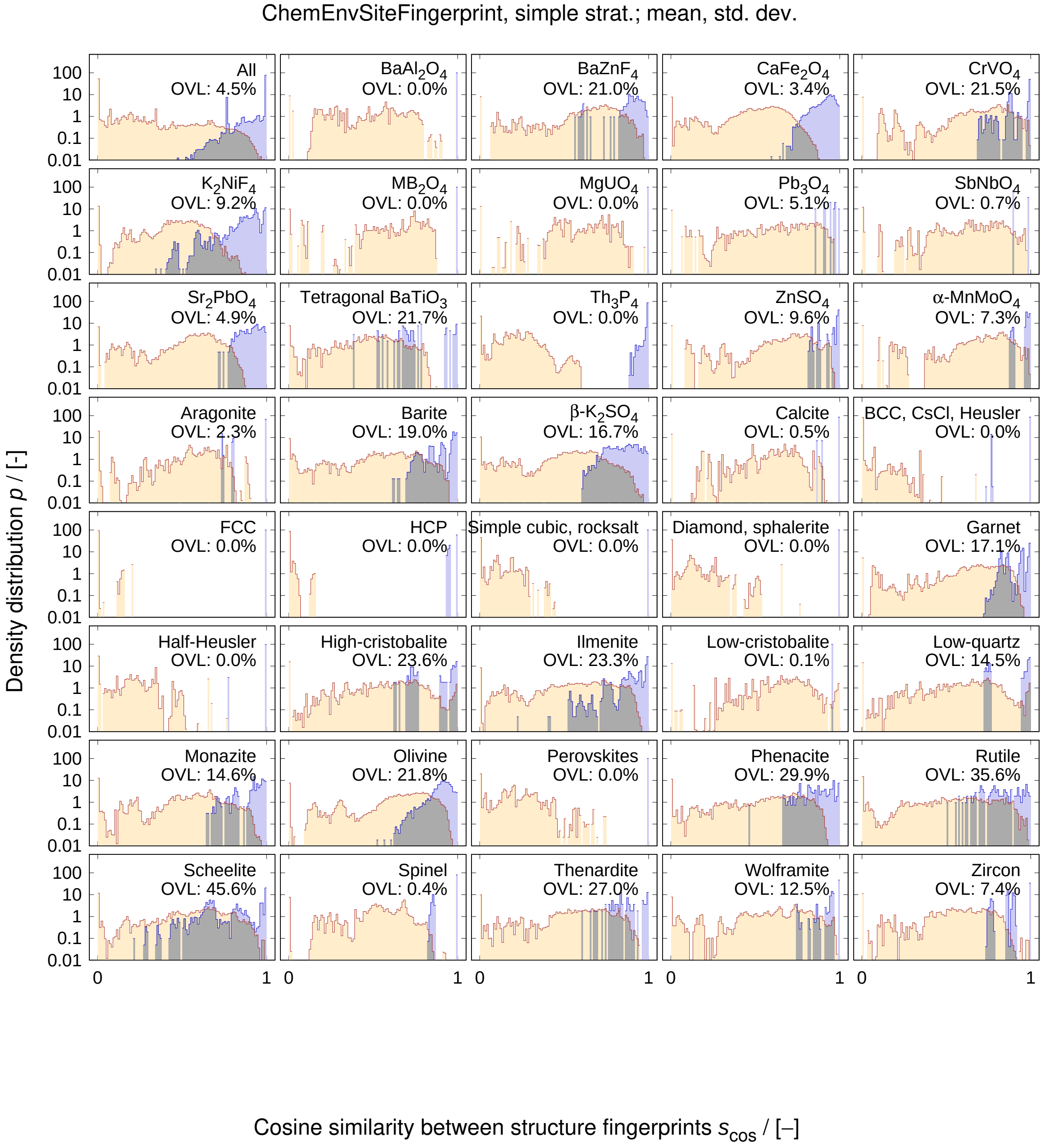

Fig. 32 Additional structure group (dis)similarity results.

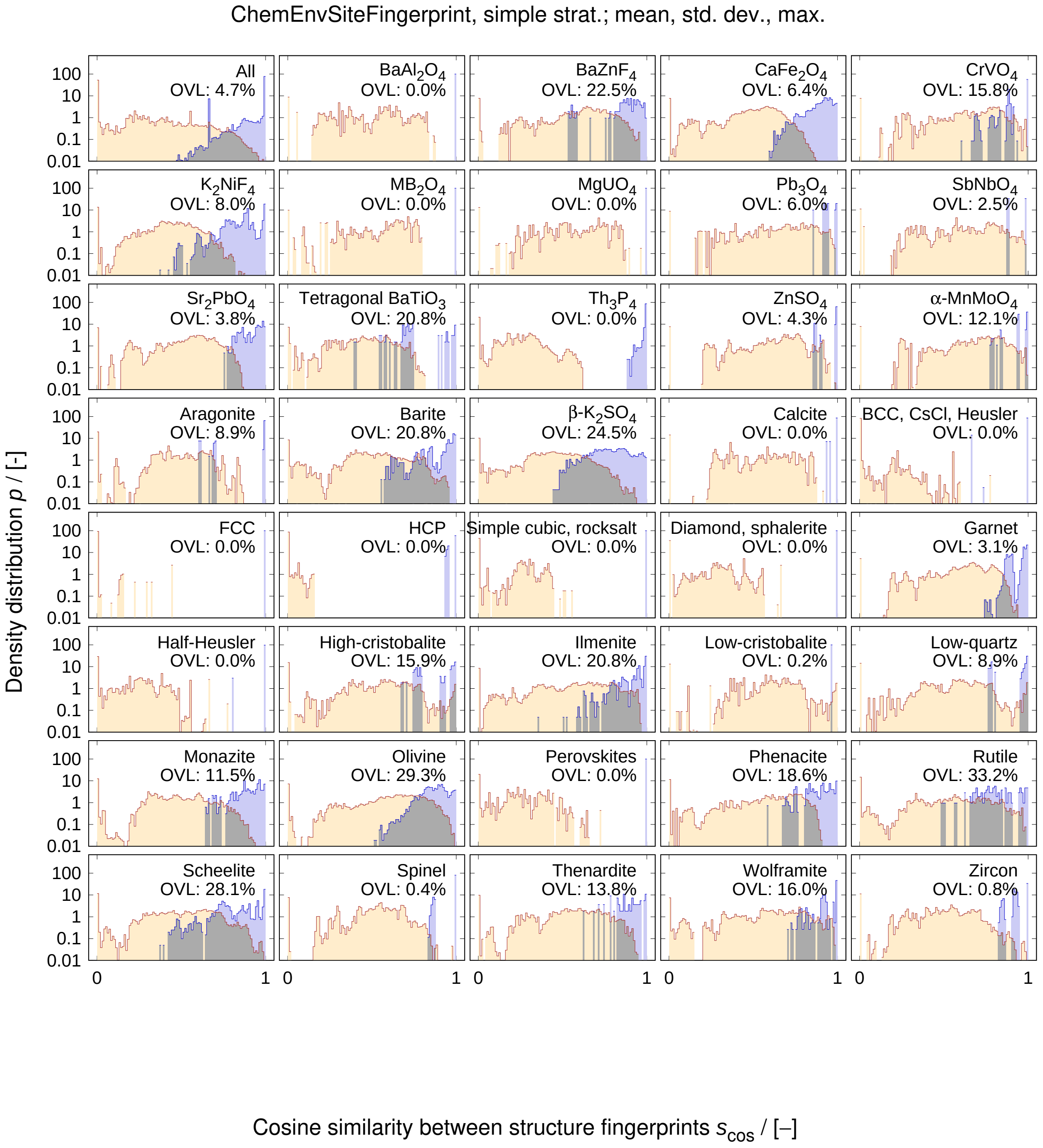

Fig. 33 Additional structure group (dis)similarity results.

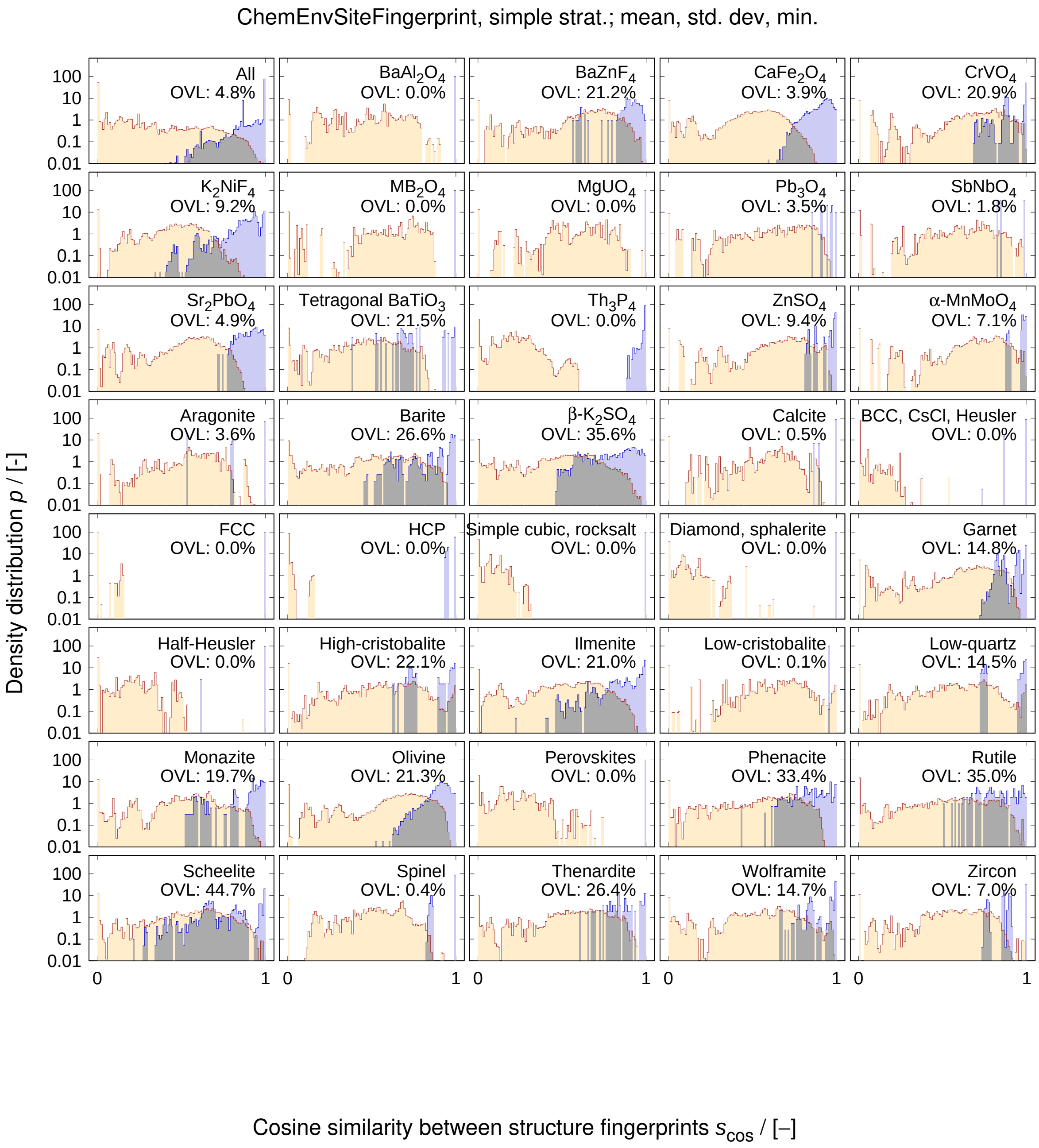

Fig. 34 Additional structure group (dis)similarity results.

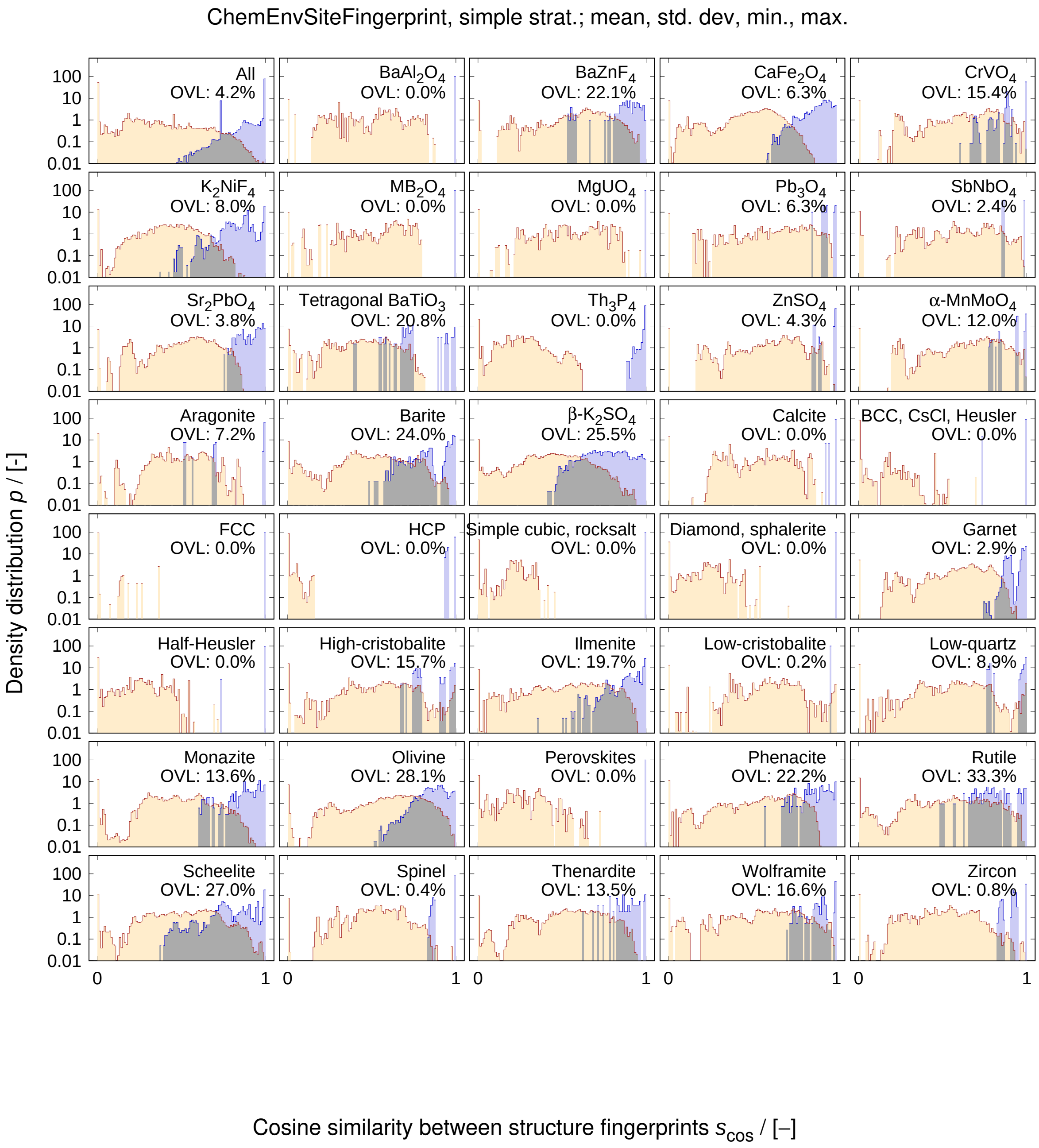

Fig. 35 Additional structure group (dis)similarity results.

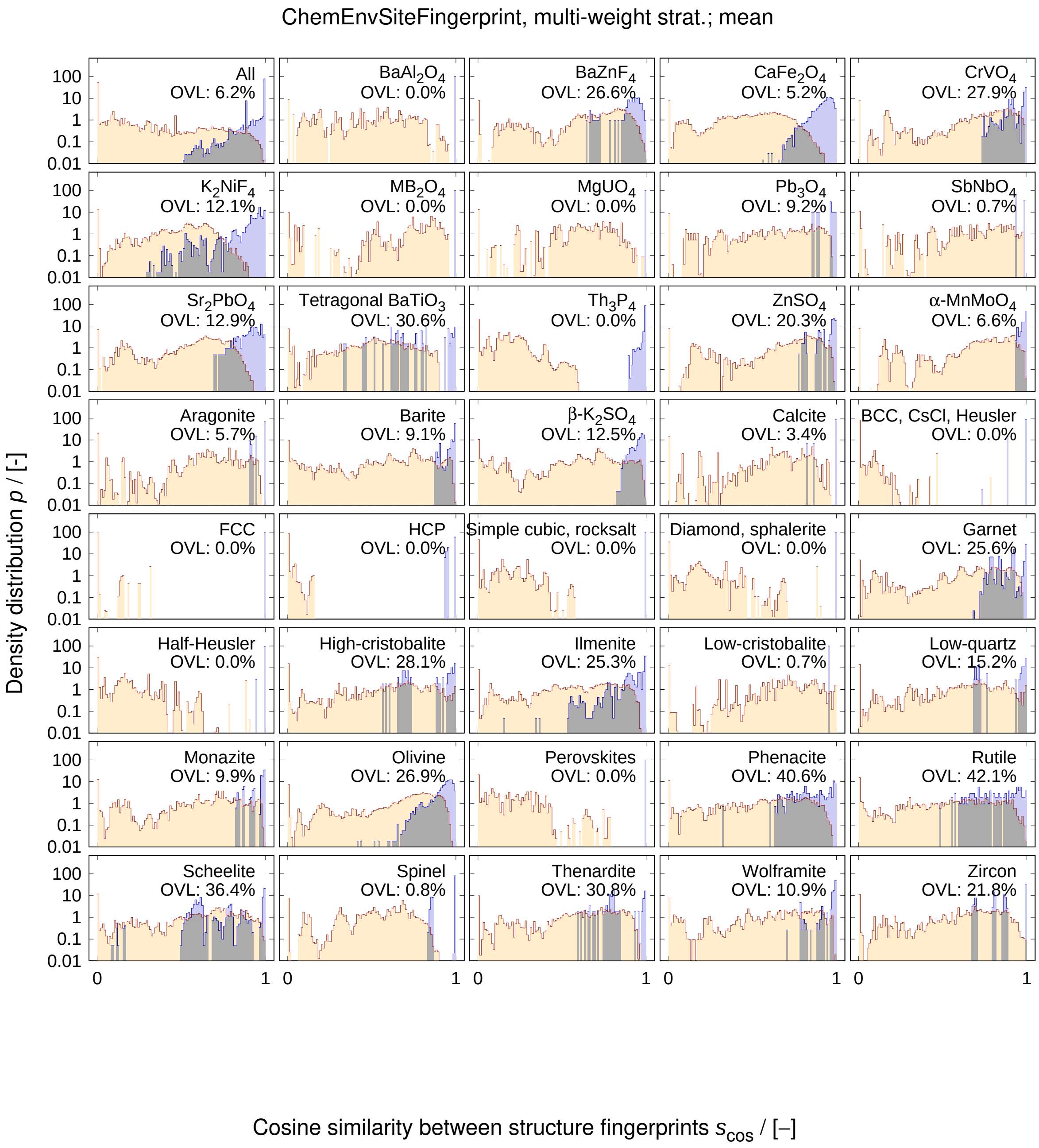

Fig. 36 Additional structure group (dis)similarity results.

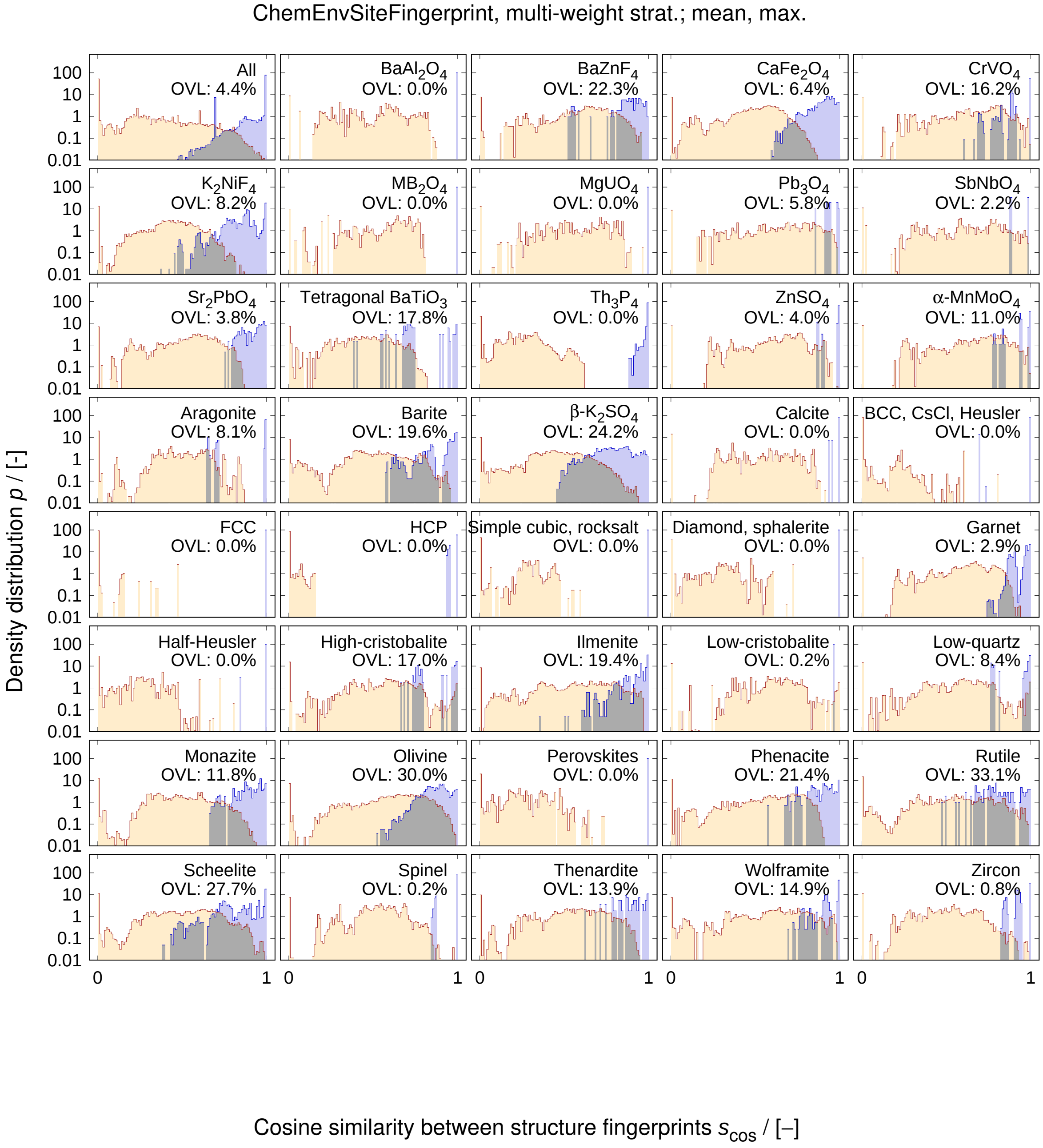

Fig. 37 Additional structure group (dis)similarity results.

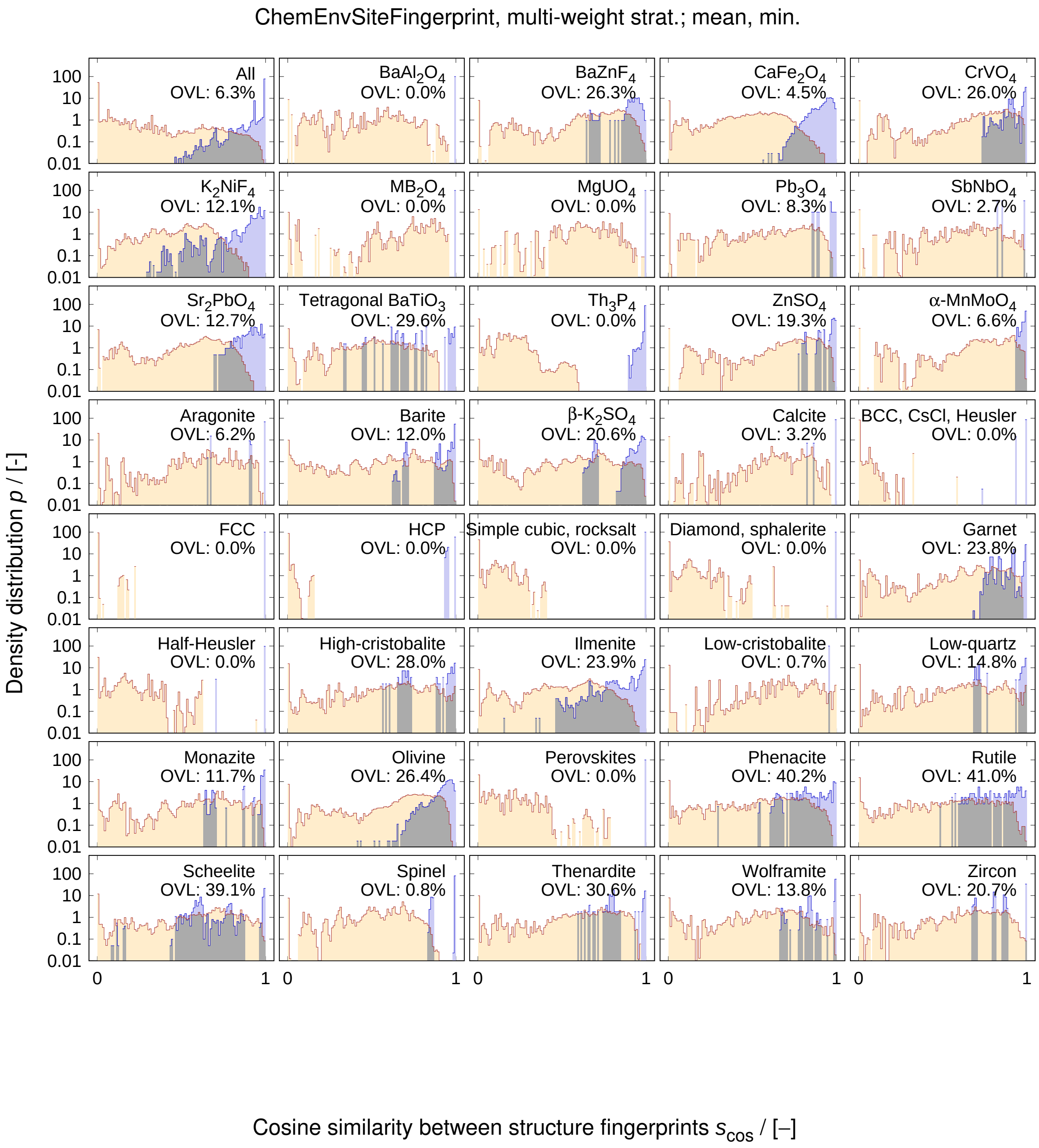

Fig. 38 Additional structure group (dis)similarity results.

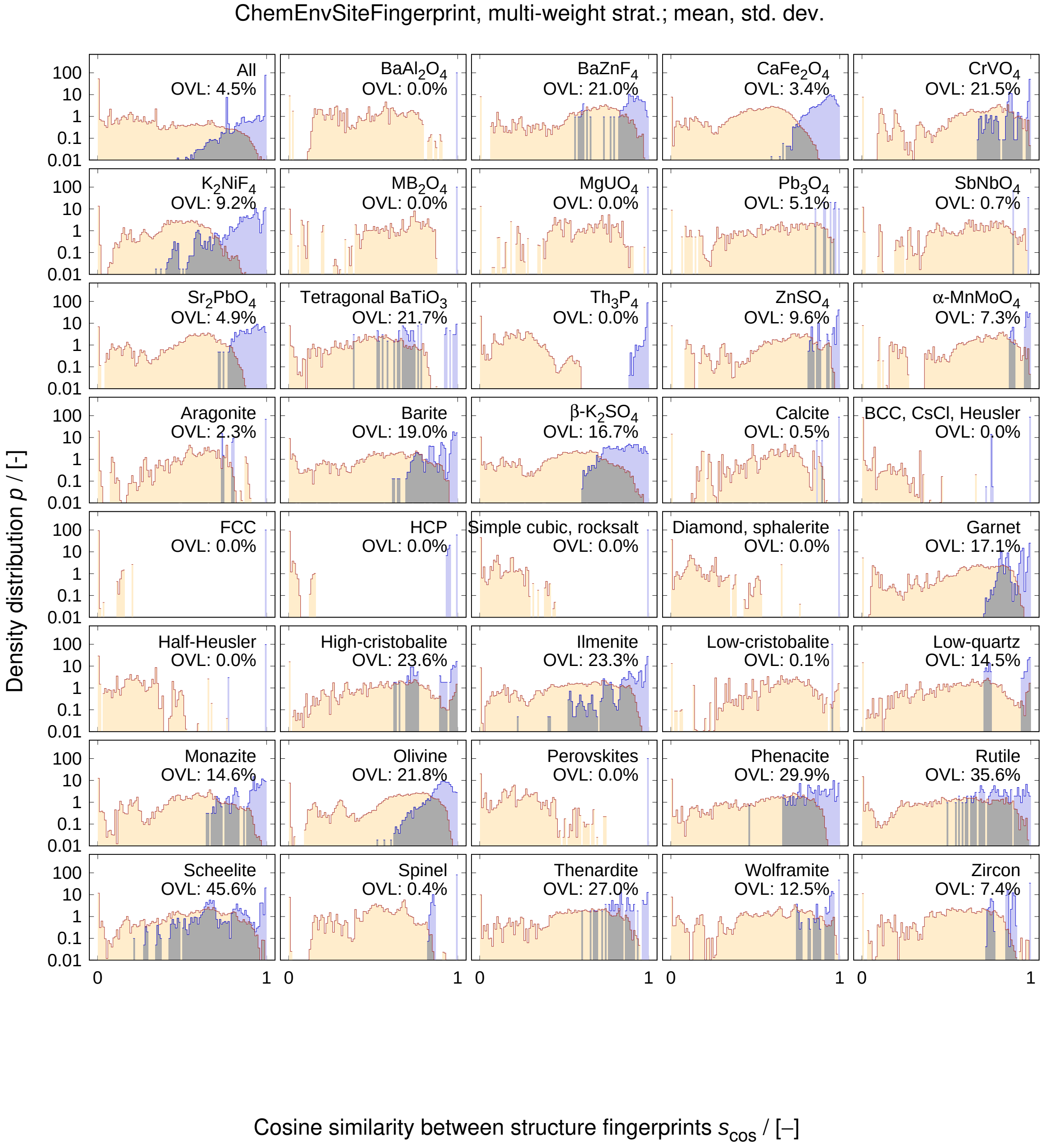

Fig. 39 Additional structure group (dis)similarity results.

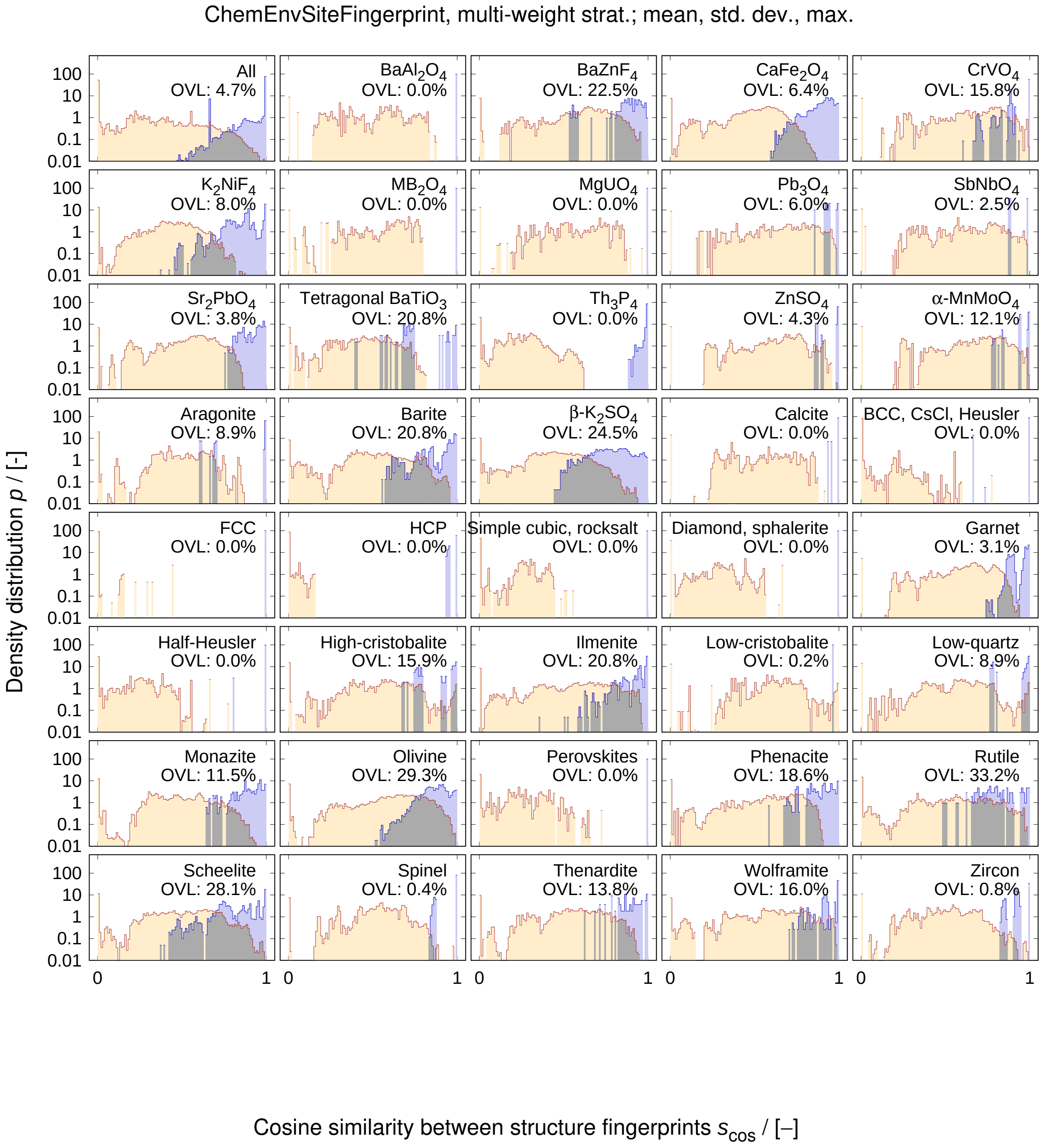

Fig. 40 Additional structure group (dis)similarity results.

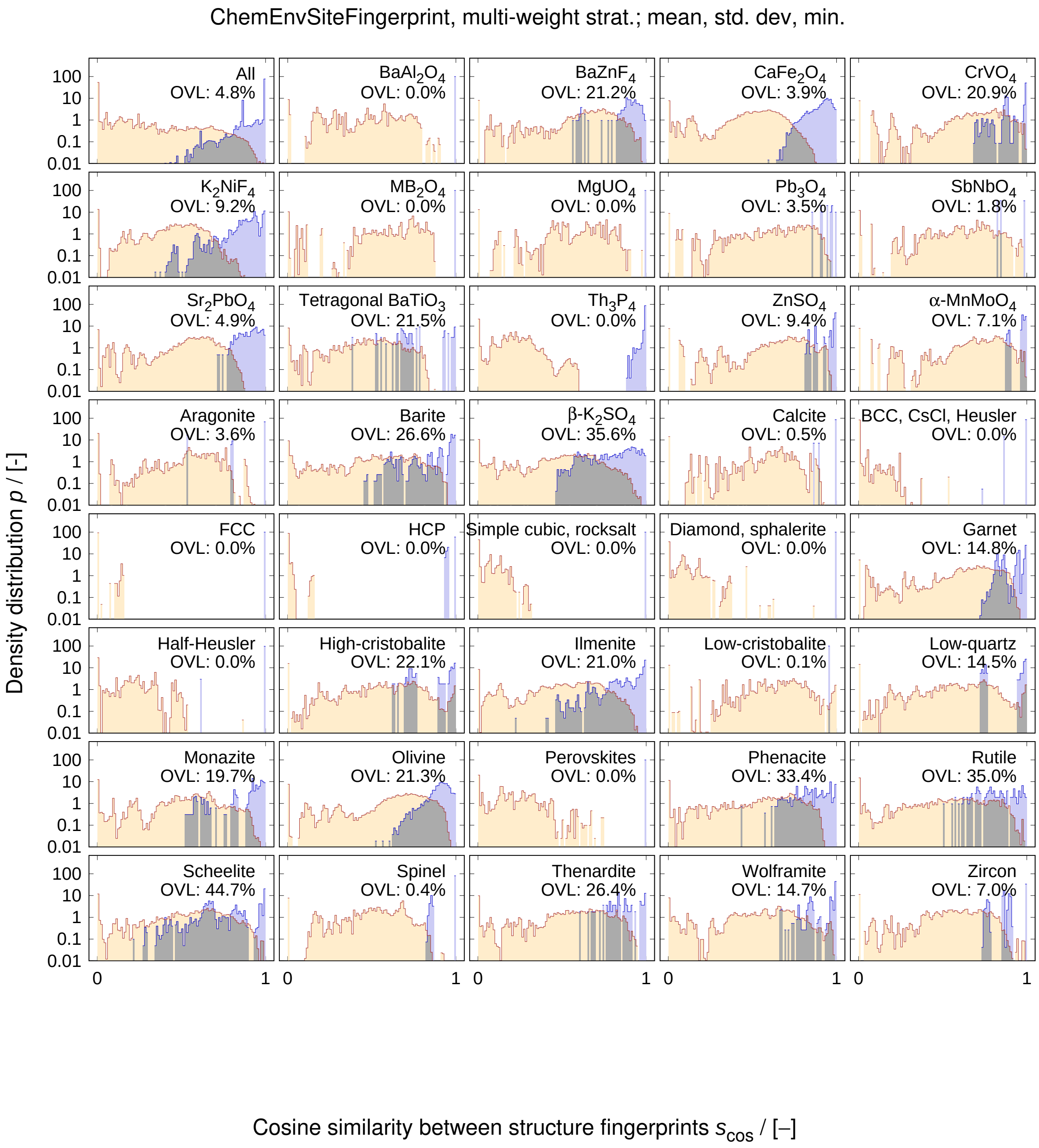

Fig. 41 Additional structure group (dis)similarity results.

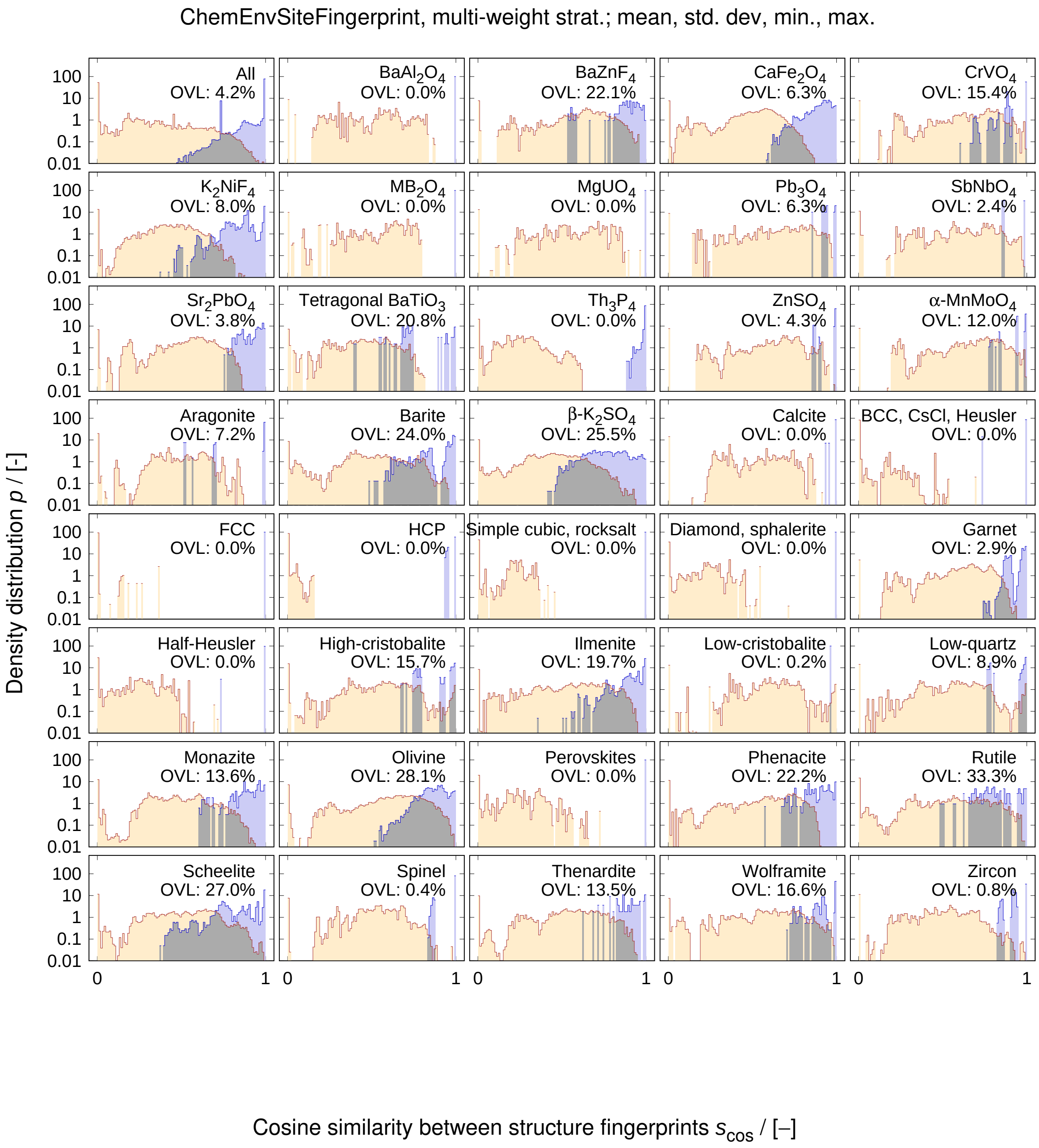

Fig. 42 Additional structure group (dis)similarity results.

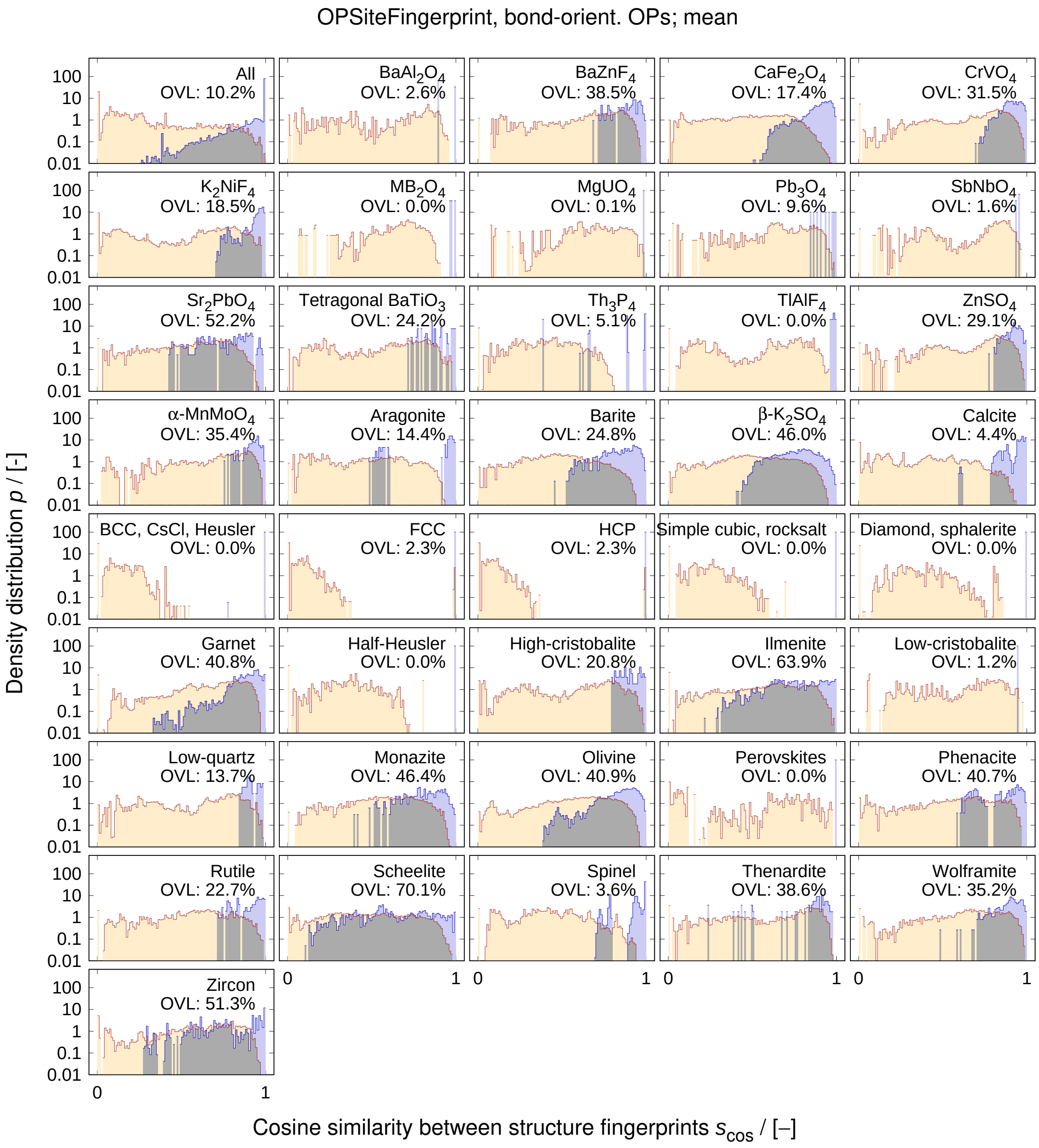

Fig. 43 Additional structure group (dis)similarity results.

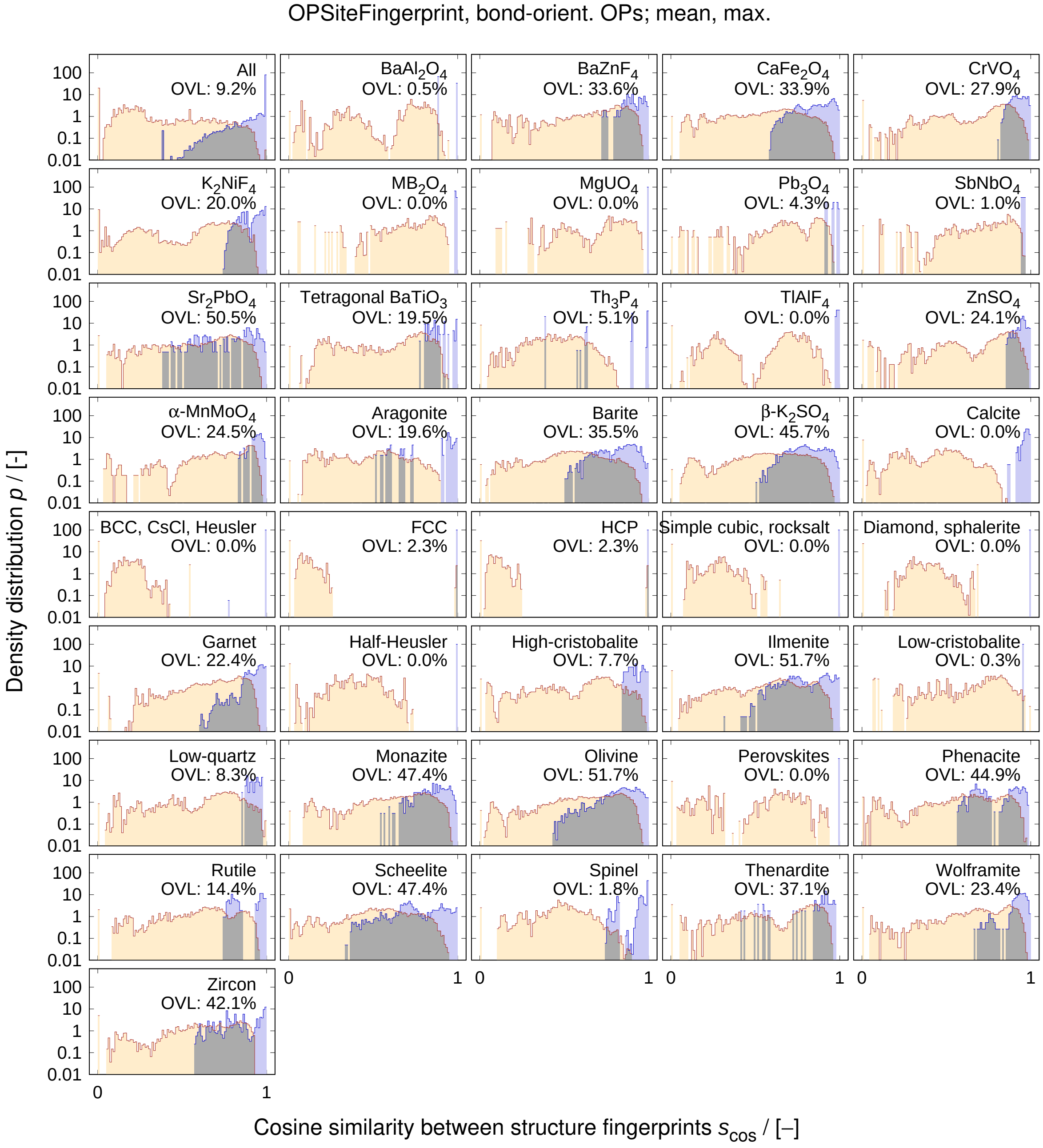

Fig. 44 Additional structure group (dis)similarity results.

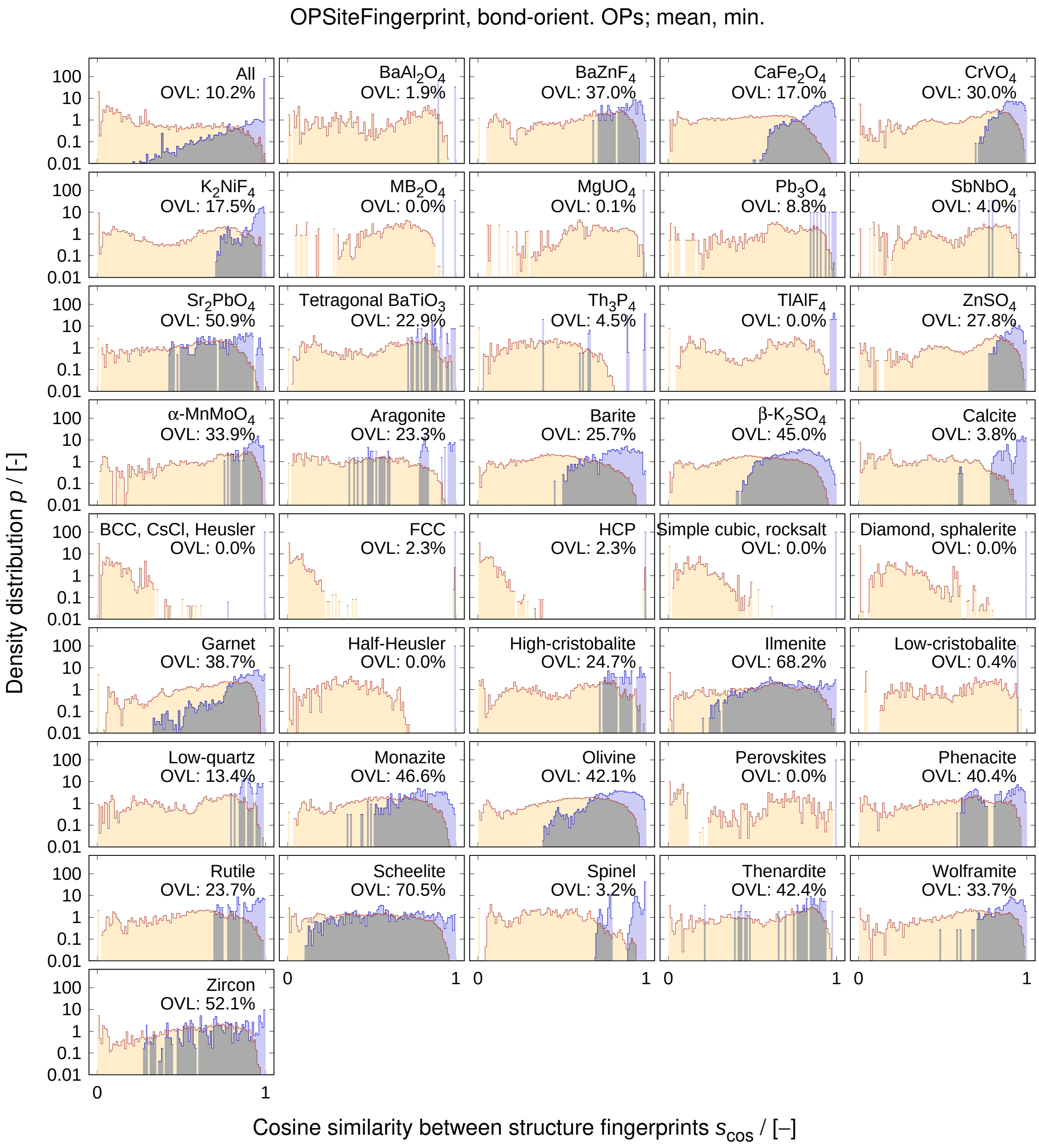

Fig. 45 Additional structure group (dis)similarity results.

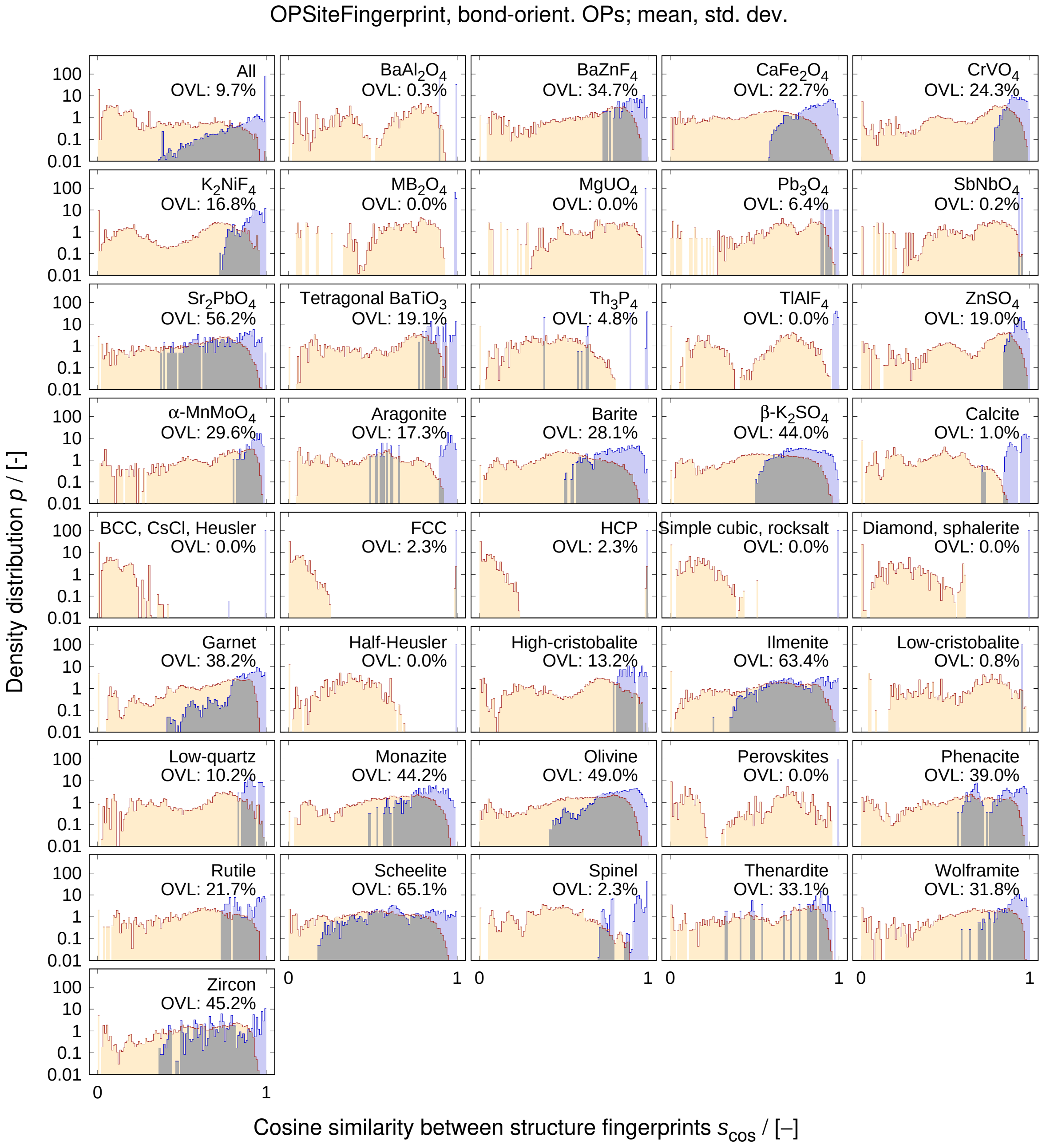

Fig. 46 Additional structure group (dis)similarity results.

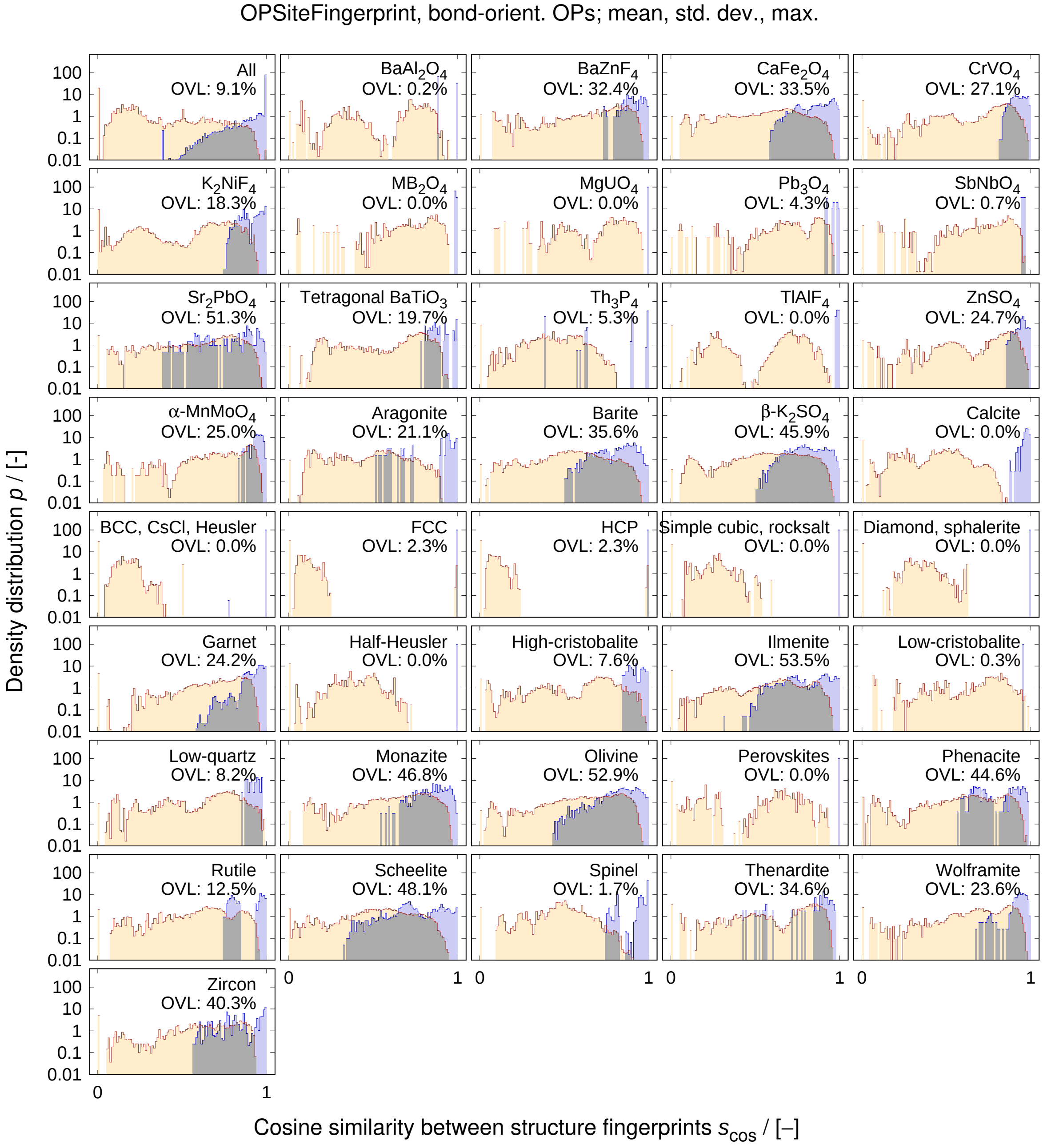

Fig. 47 Additional structure group (dis)similarity results.

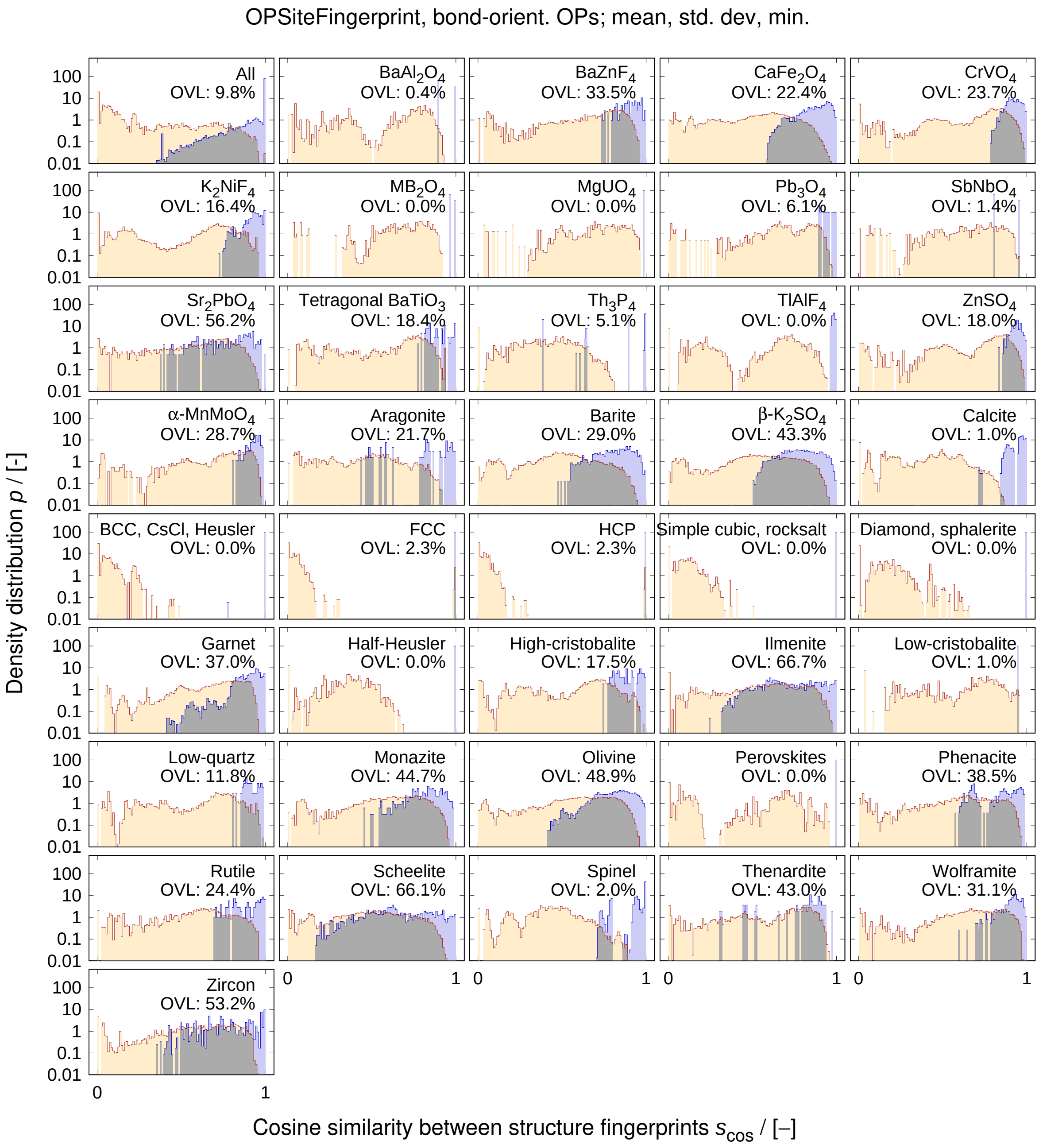

Fig. 48 Additional structure group (dis)similarity results.

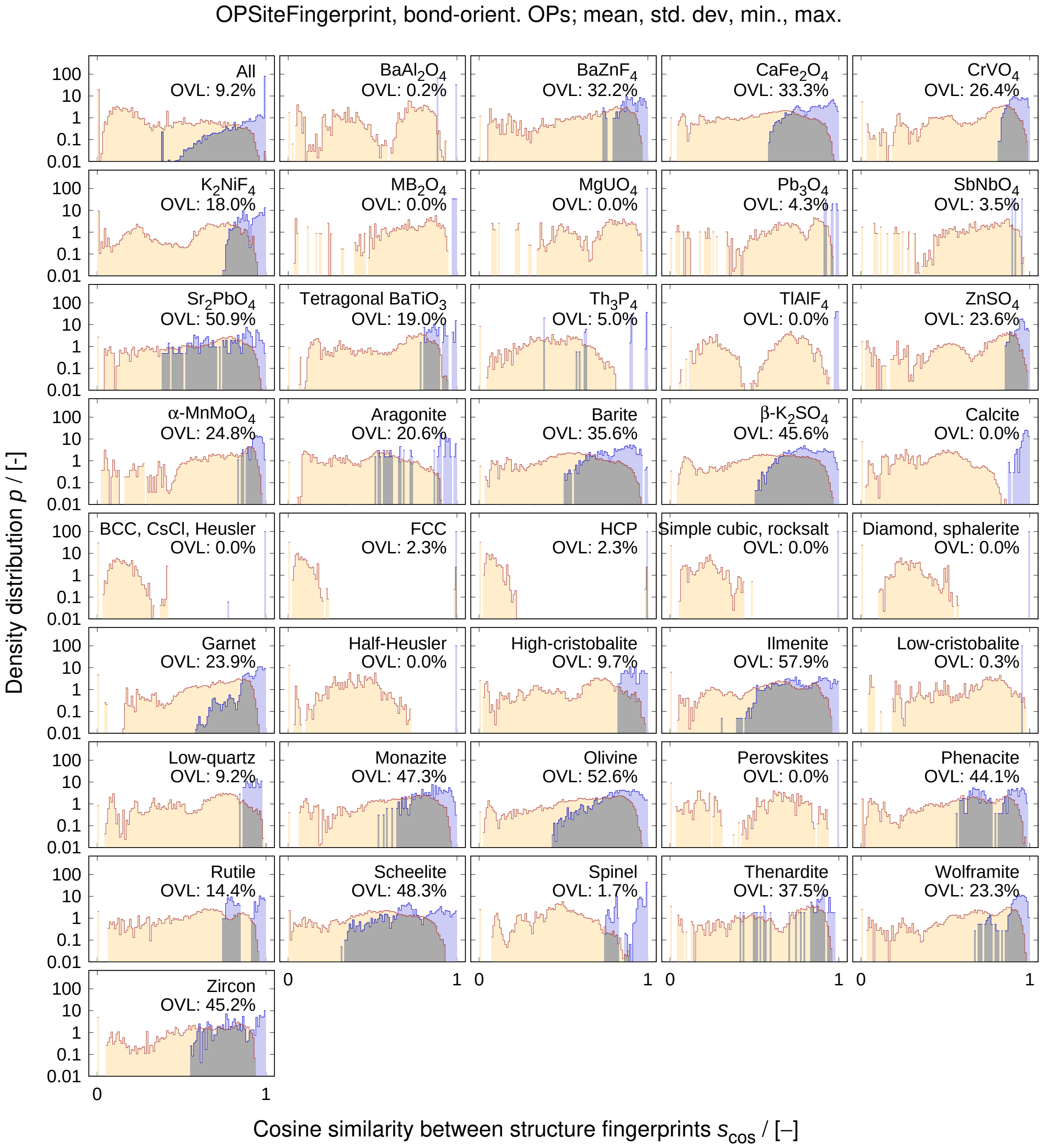

Fig. 49 Additional structure group (dis)similarity results.

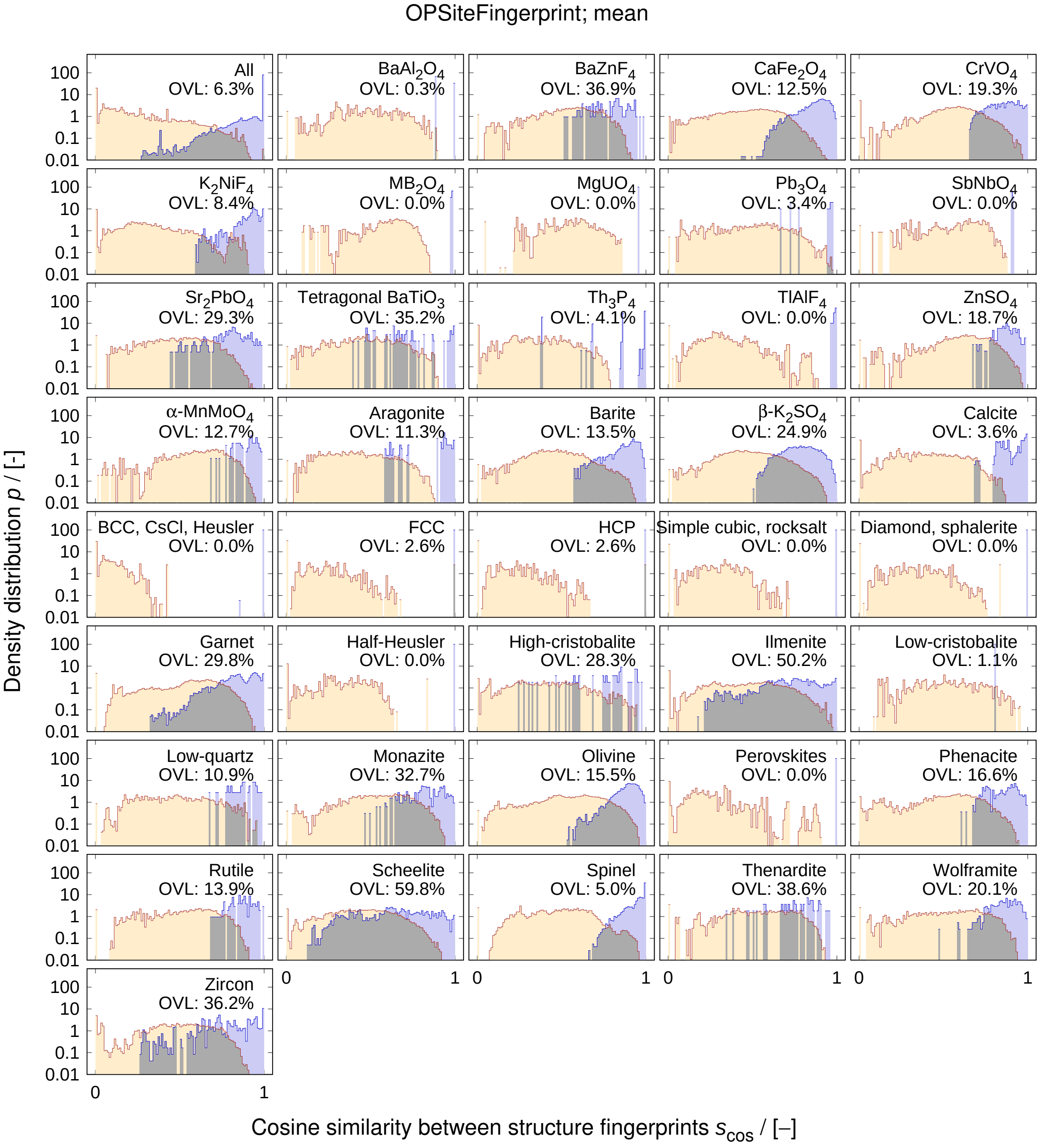

Fig. 50 Additional structure group (dis)similarity results.

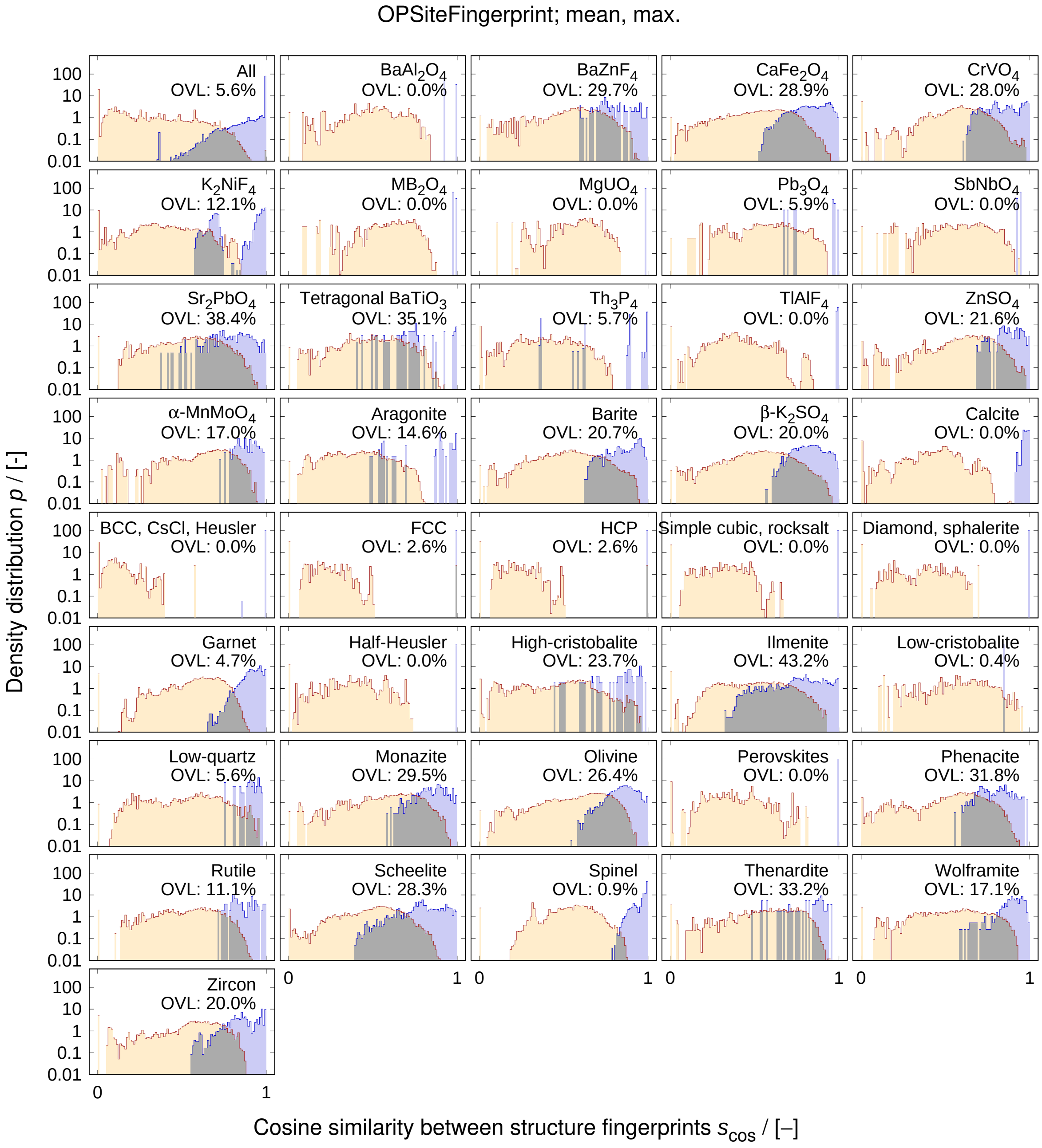

Fig. 51 Additional structure group (dis)similarity results.

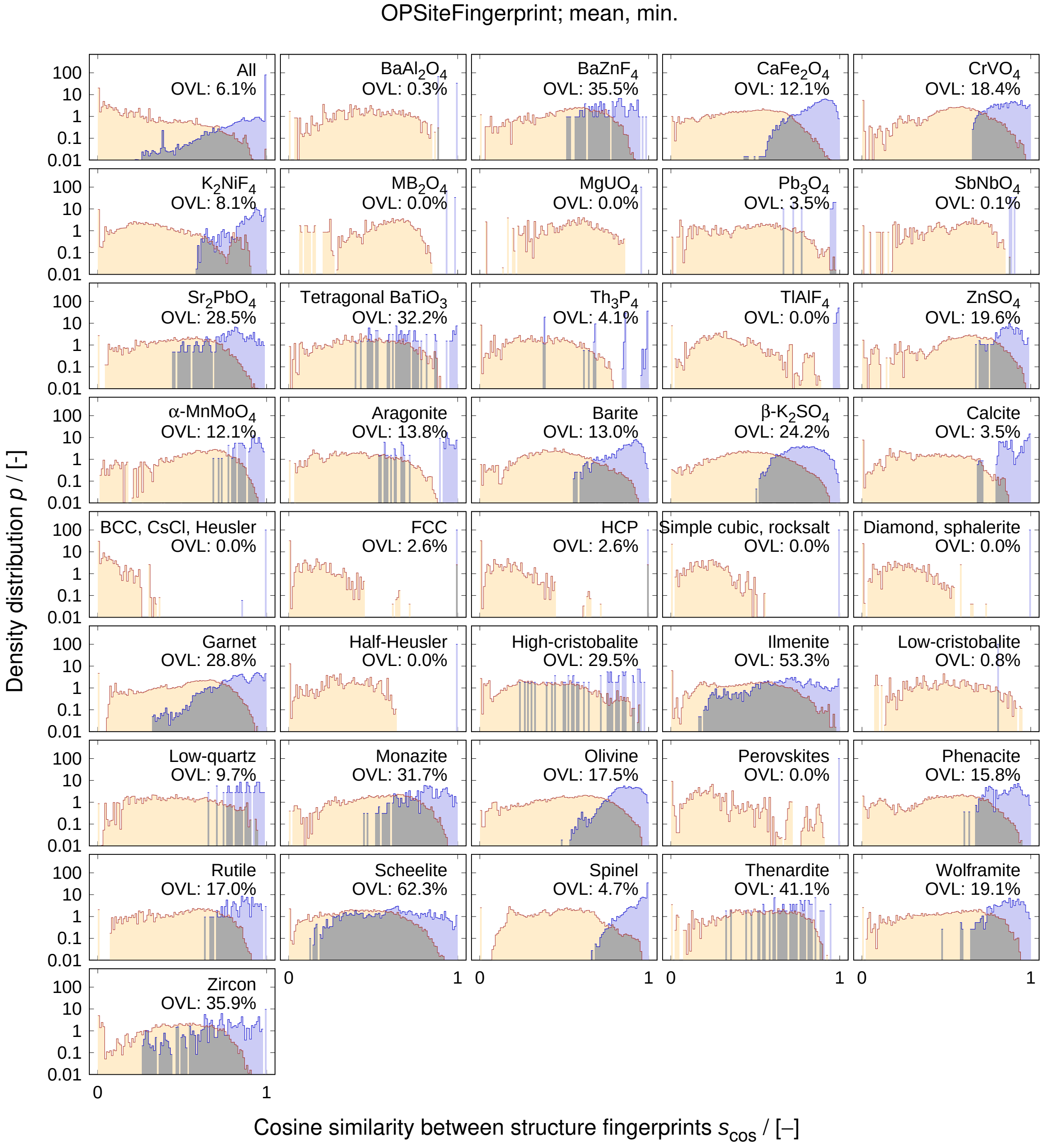

Fig. 52 Additional structure group (dis)similarity results.

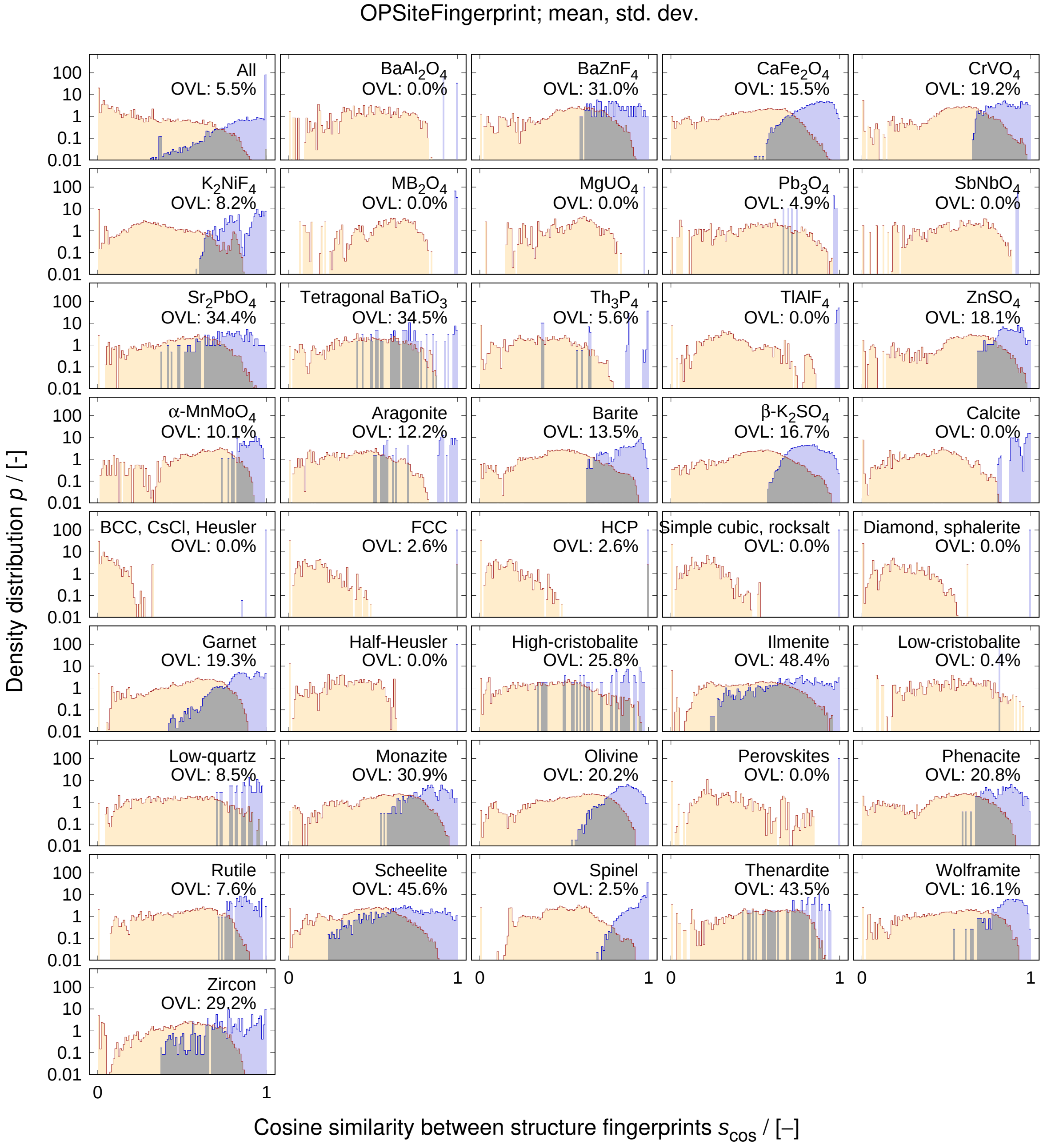

Fig. 53 Additional structure group (dis)similarity results.

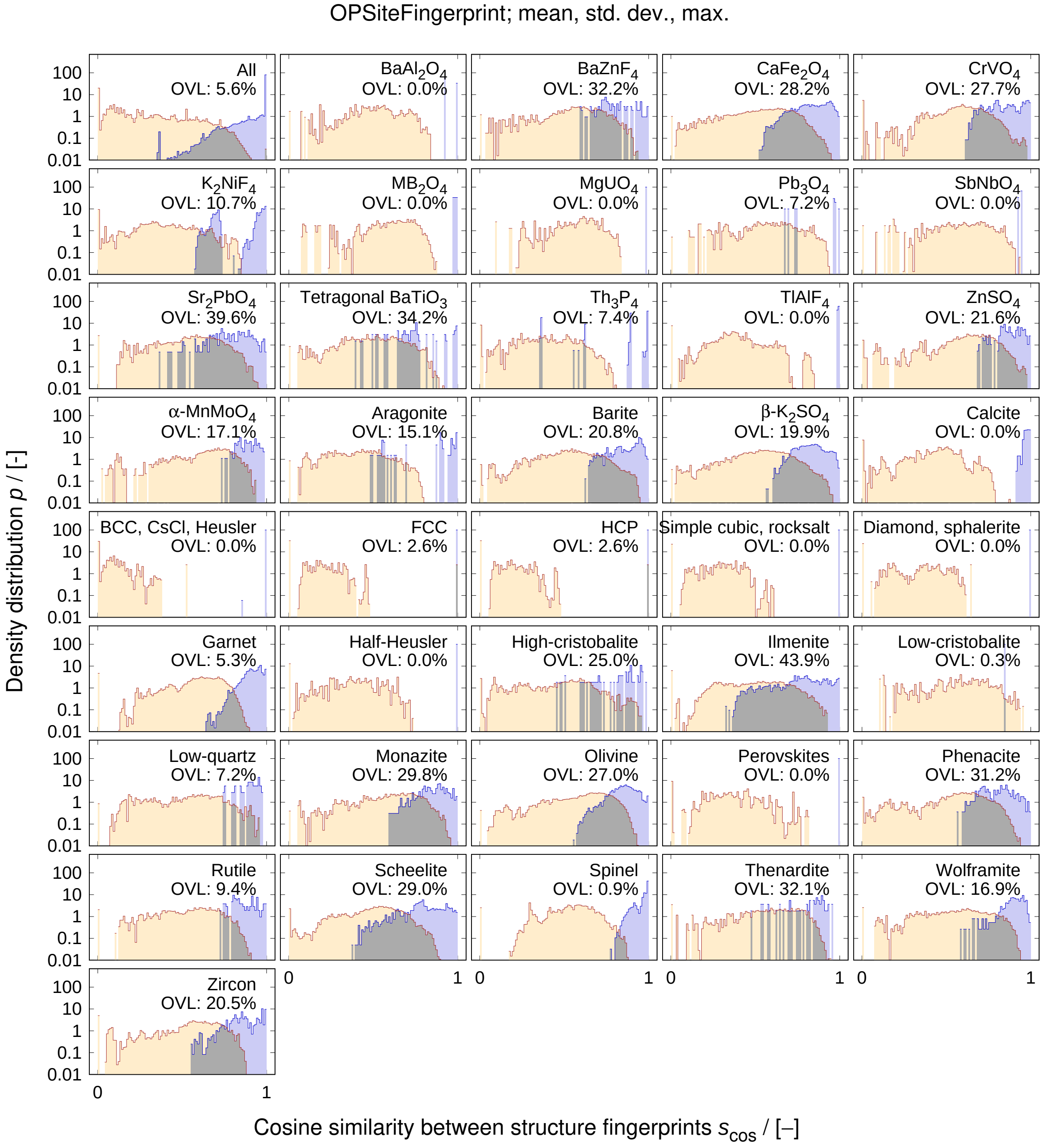

Fig. 54 Additional structure group (dis)similarity results.

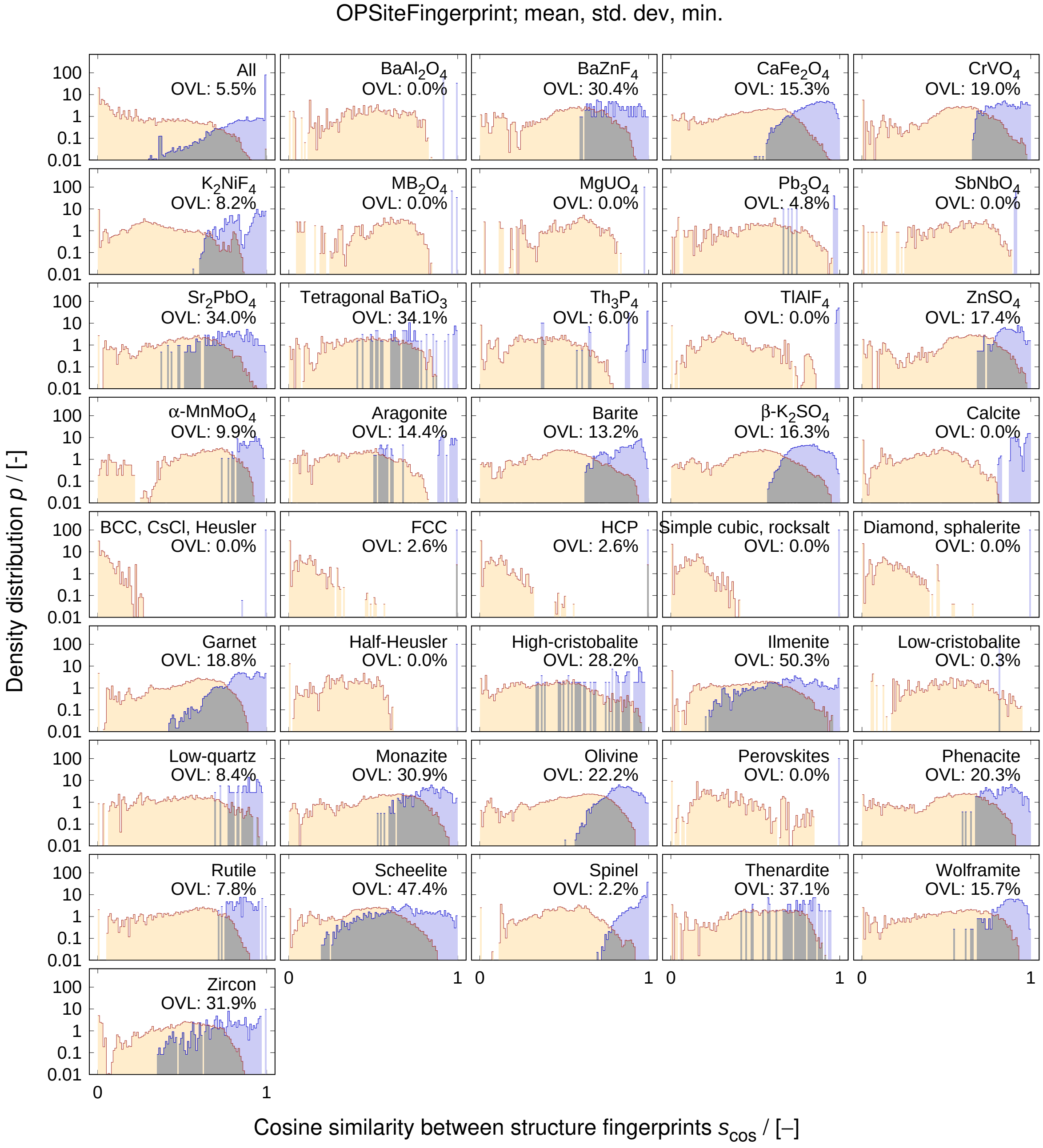

Fig. 55 Additional structure group (dis)similarity results.

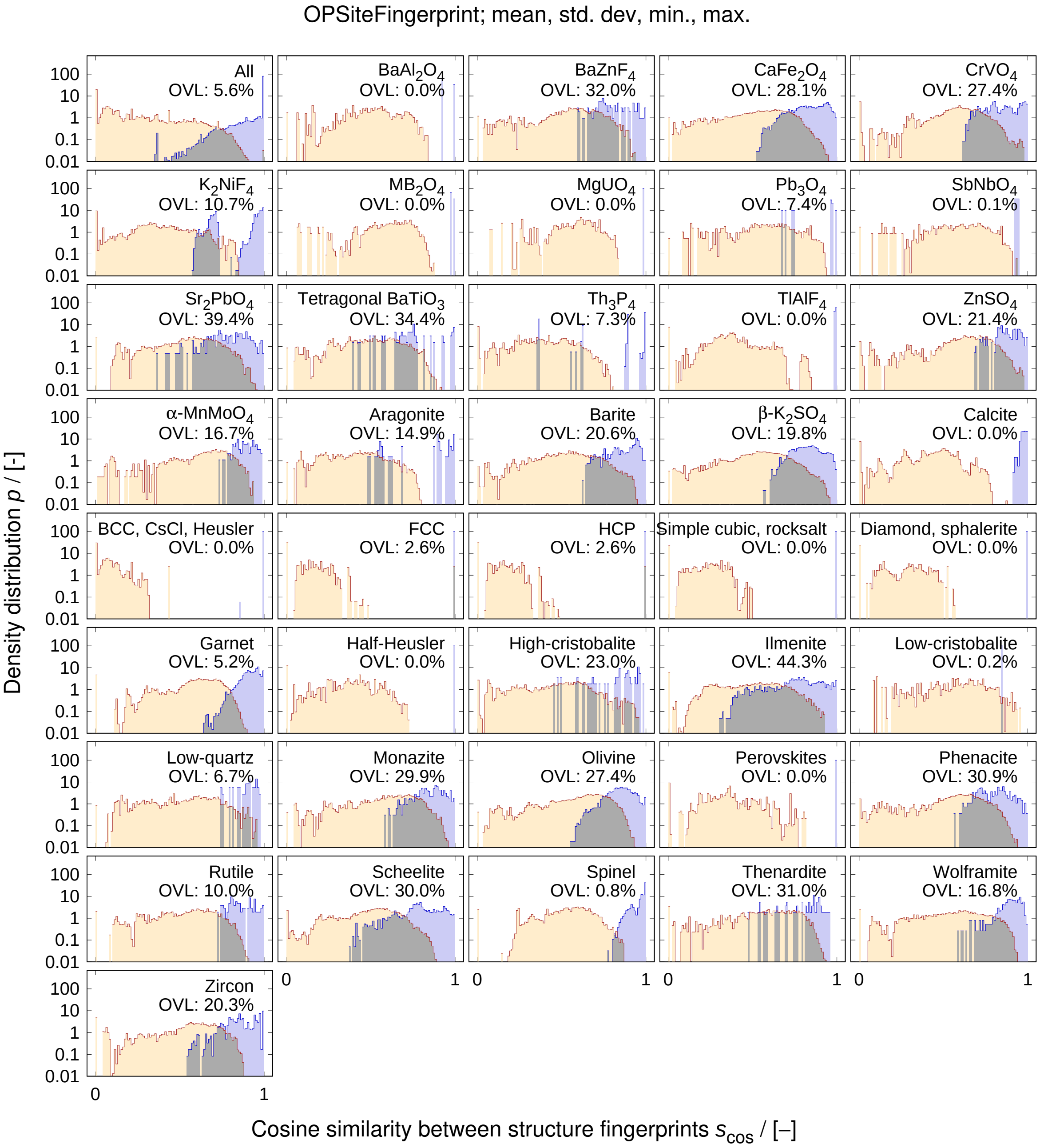

Fig. 56 Additional structure group (dis)similarity results.

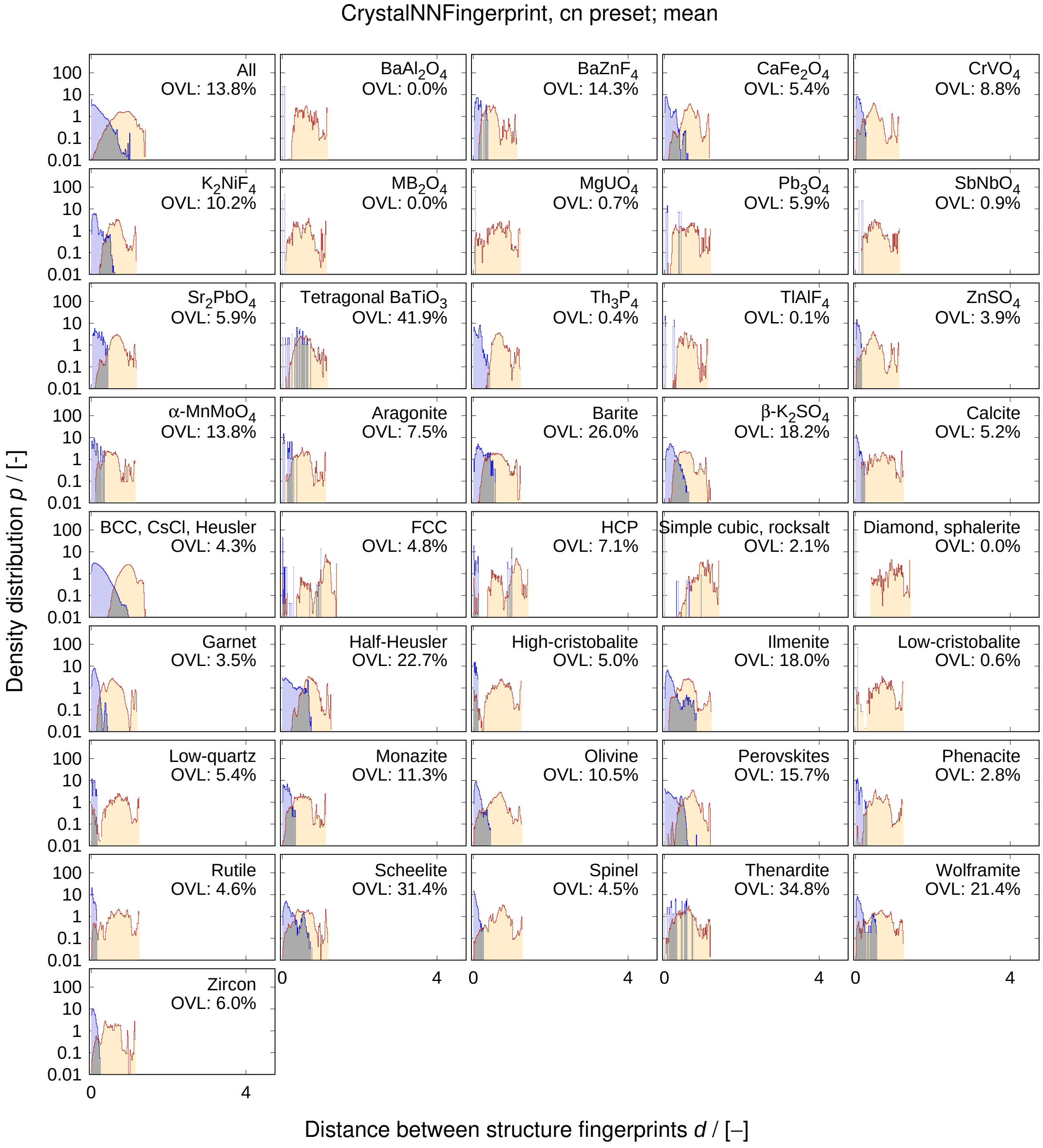

Fig. 57 Additional structure group (dis)similarity results.

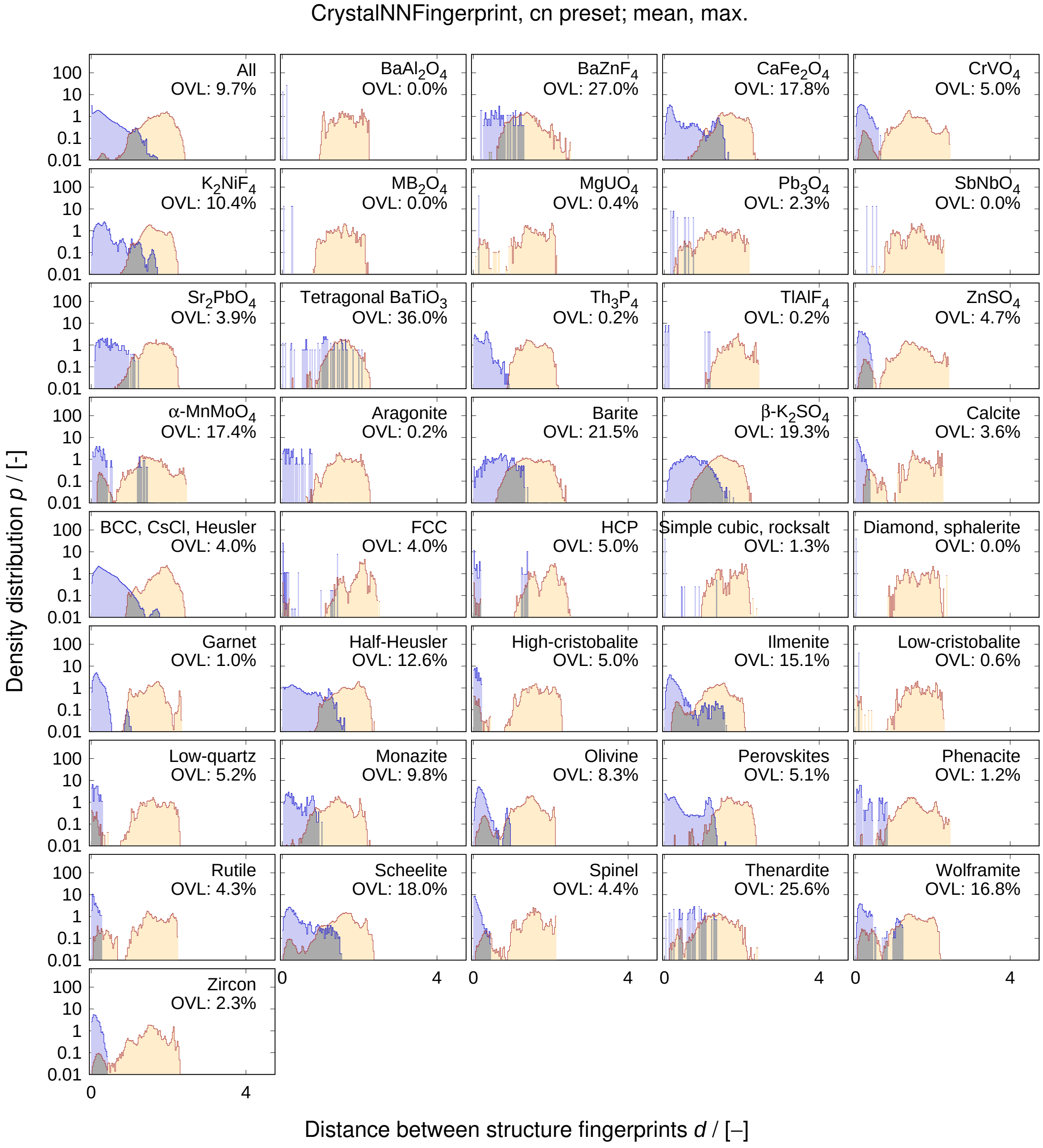

Fig. 58 Additional structure group (dis)similarity results.

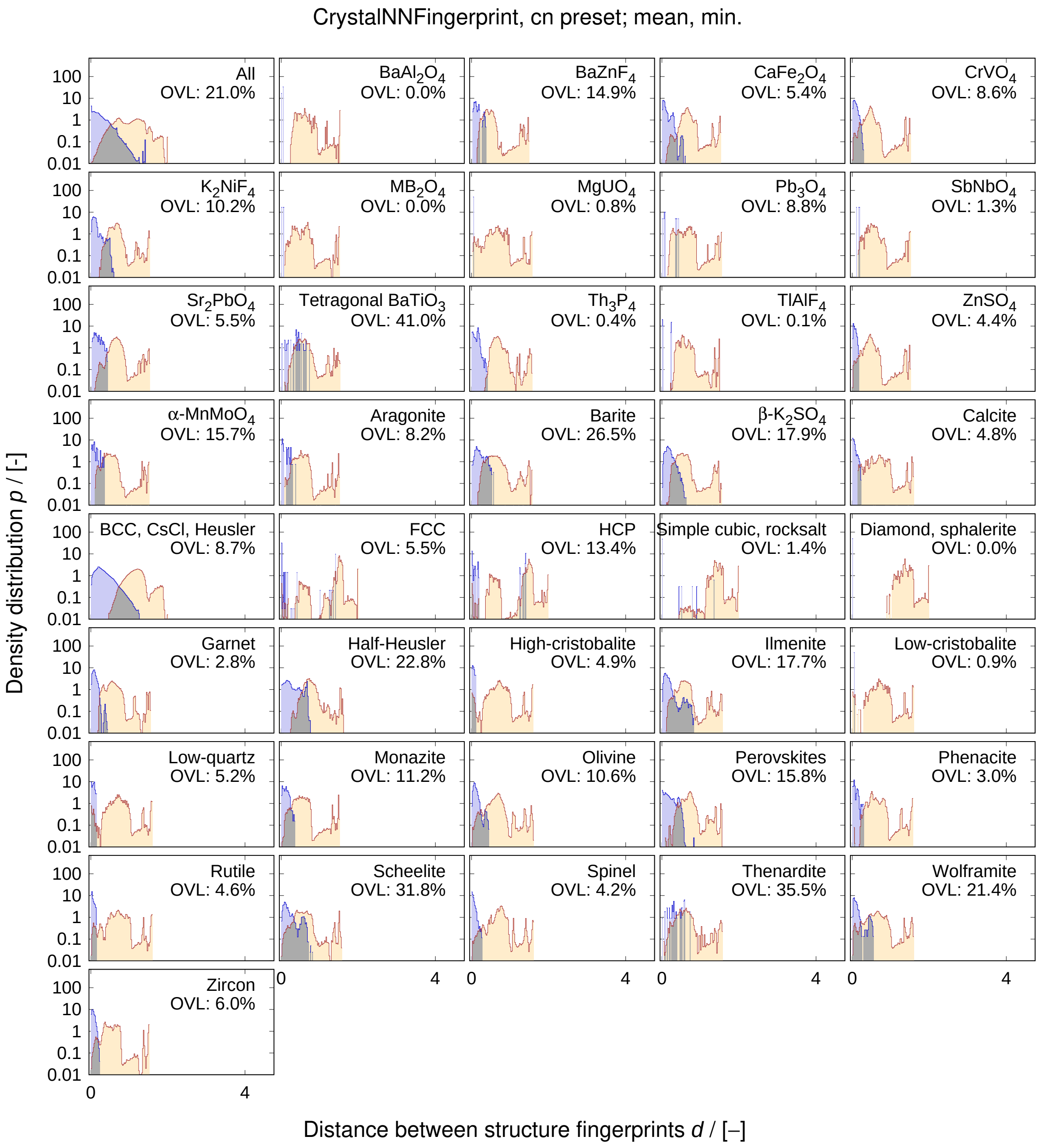

Fig. 59 Additional structure group (dis)similarity results.

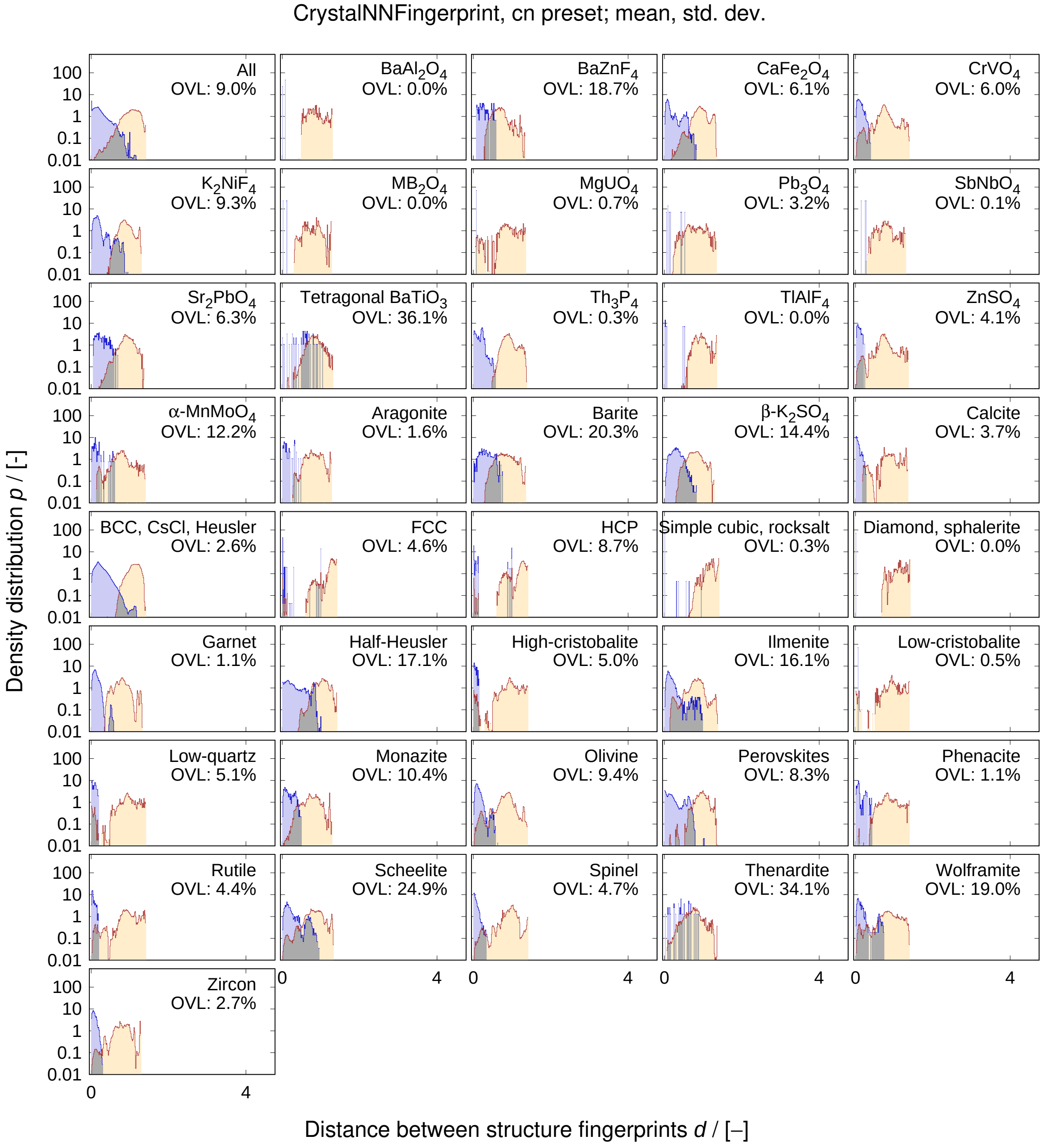

Fig. 60 Additional structure group (dis)similarity results.

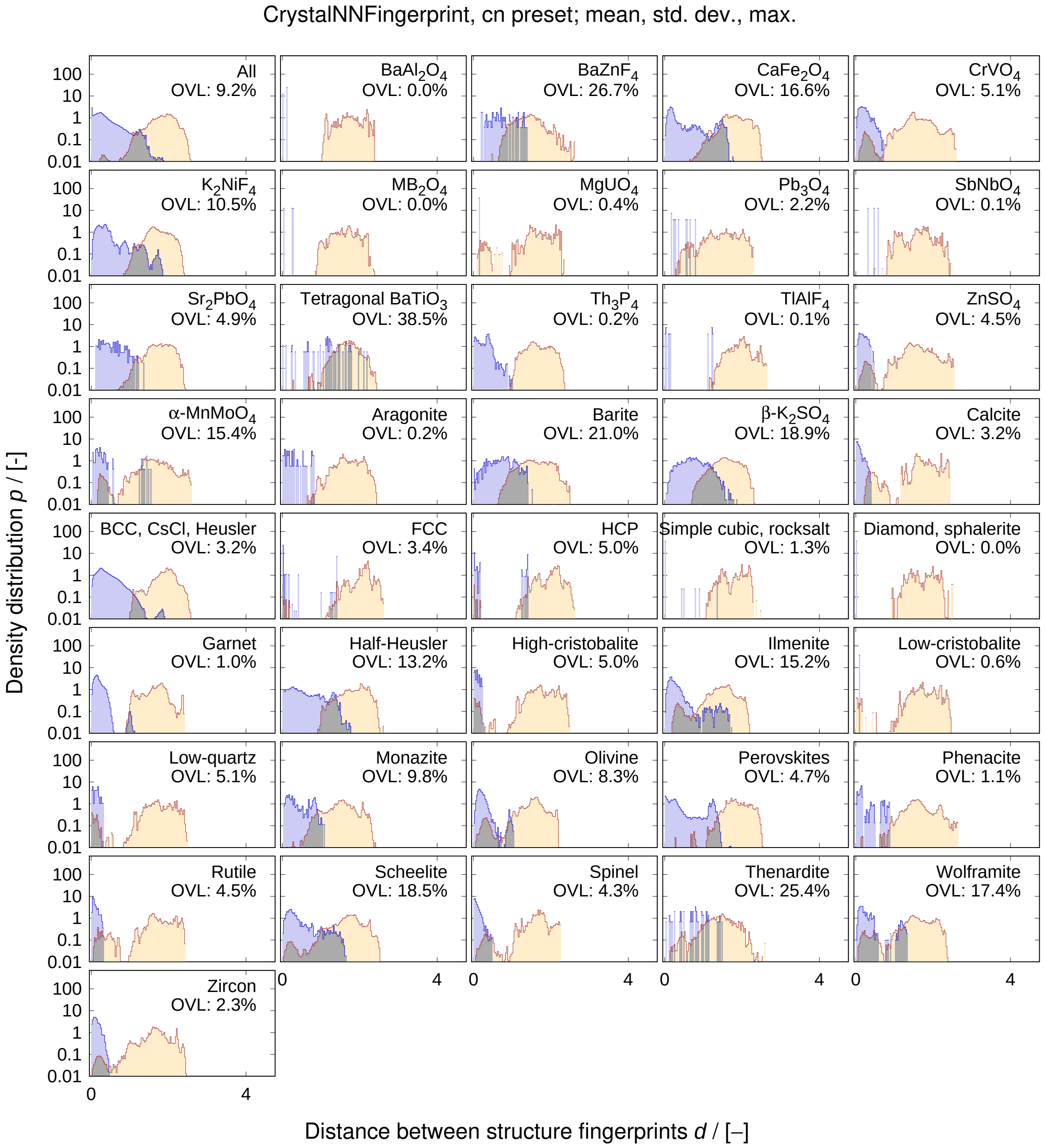

Fig. 61 Additional structure group (dis)similarity results.

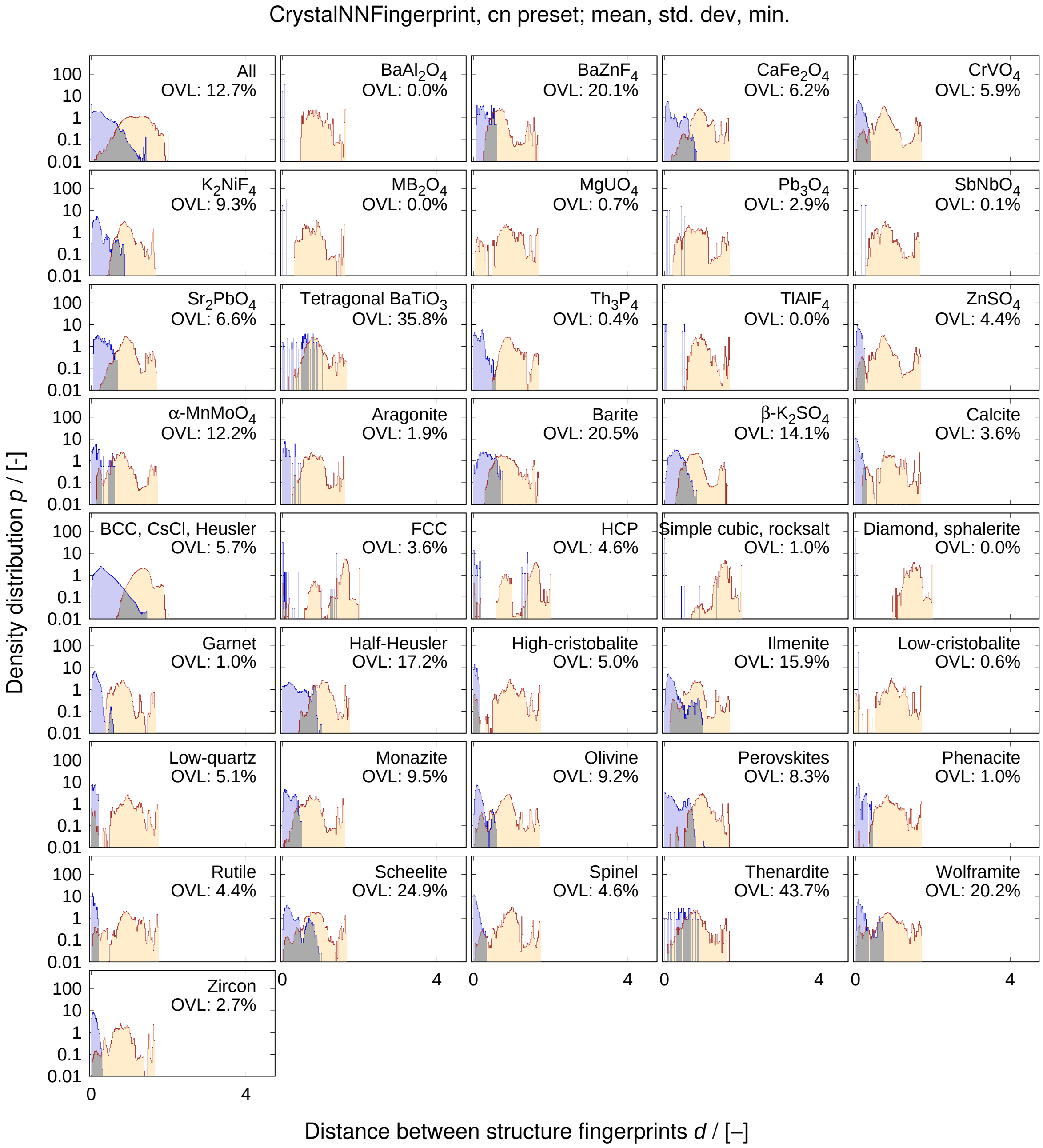

Fig. 62 Additional structure group (dis)similarity results.

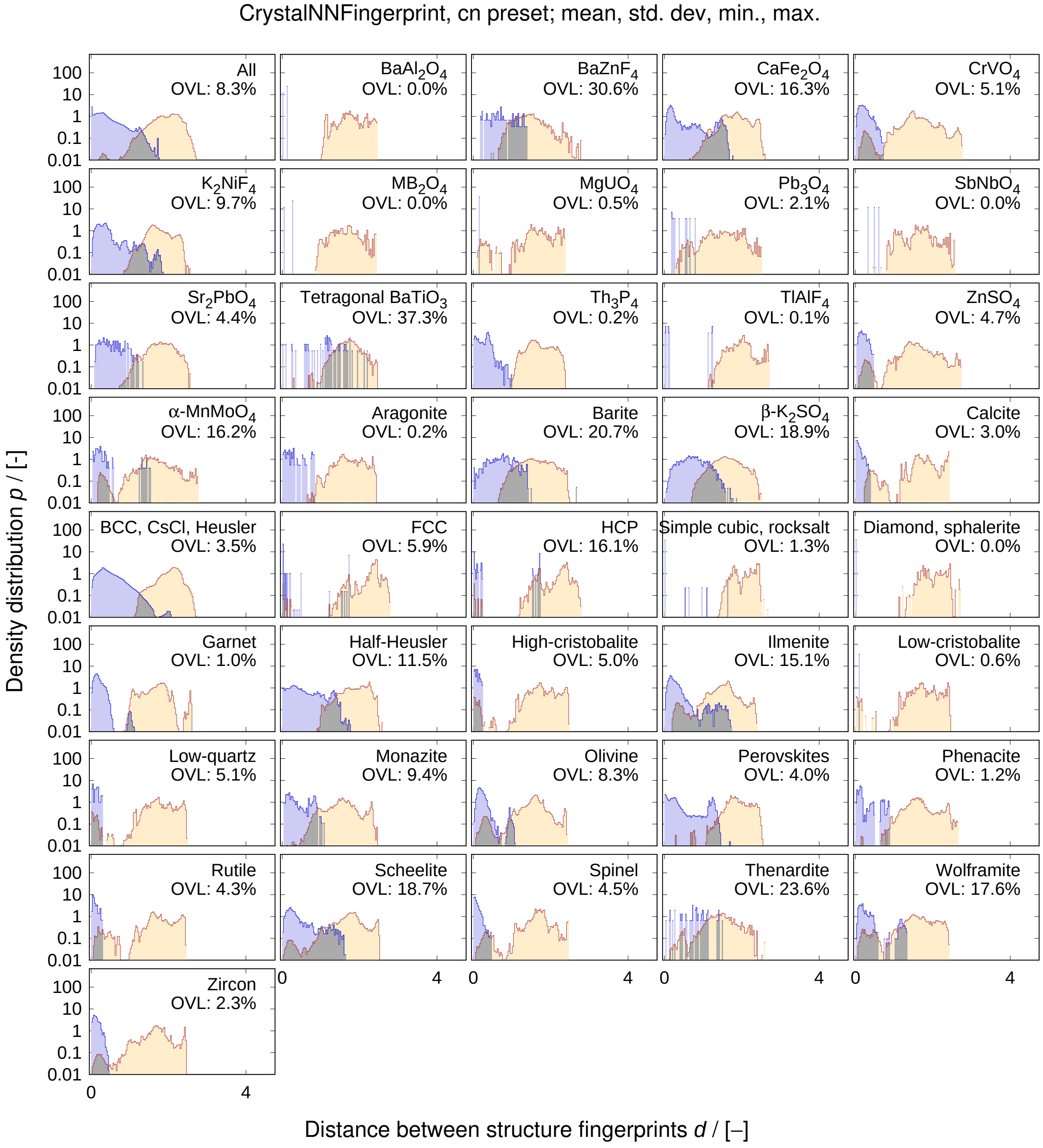

Fig. 63 Additional structure group (dis)similarity results.

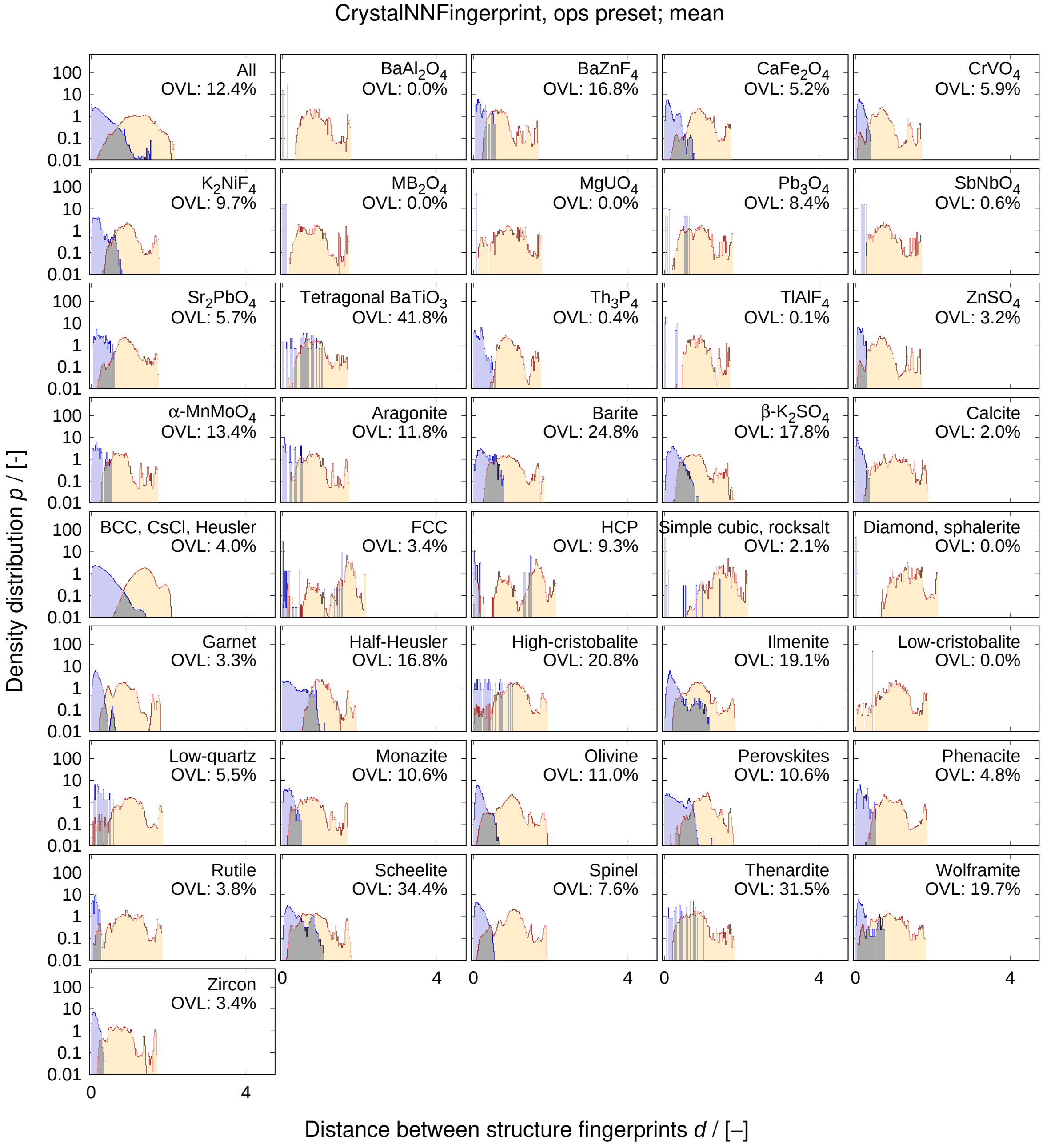

Fig. 64 Additional structure group (dis)similarity results.

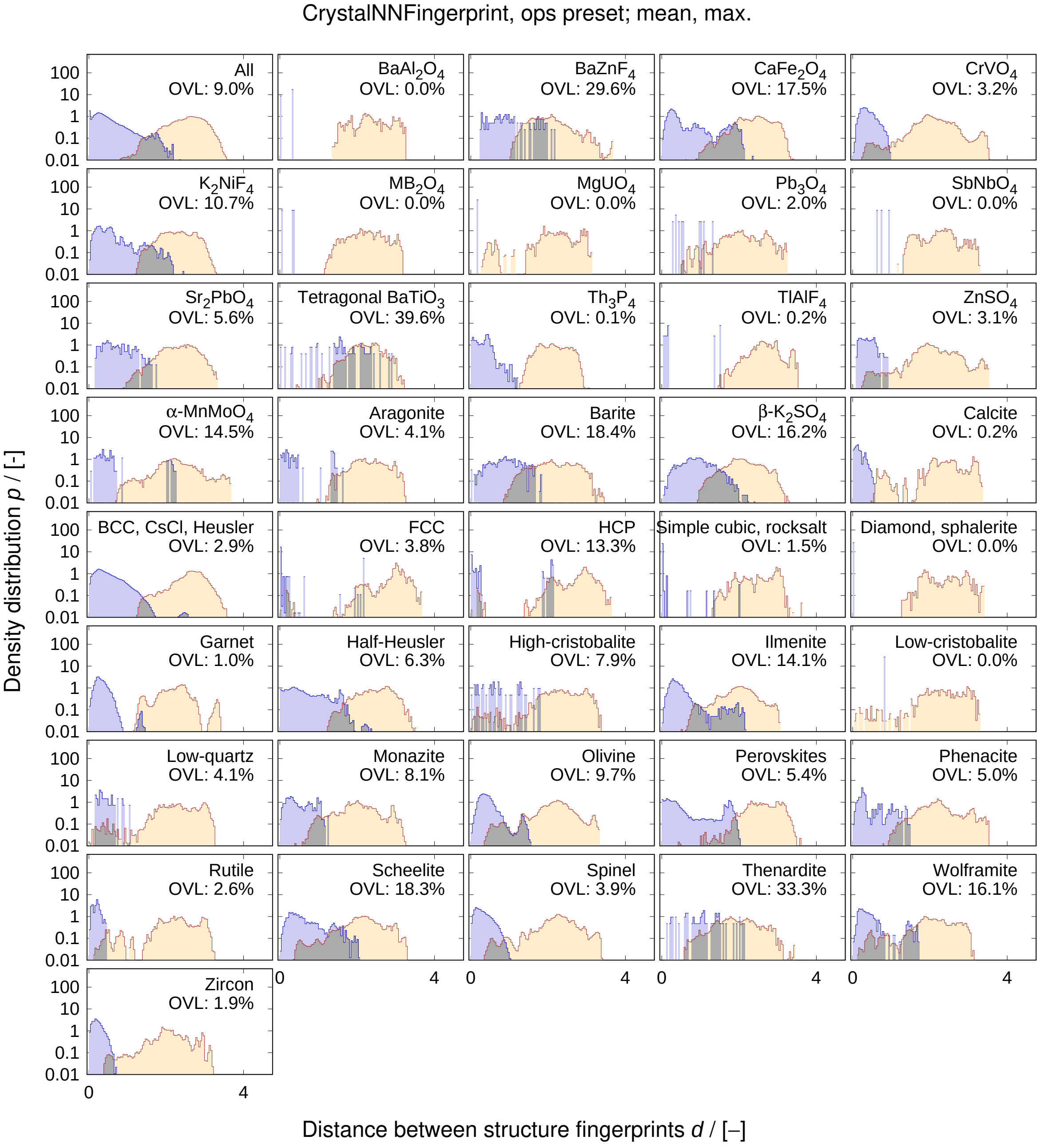

Fig. 65 Additional structure group (dis)similarity results.

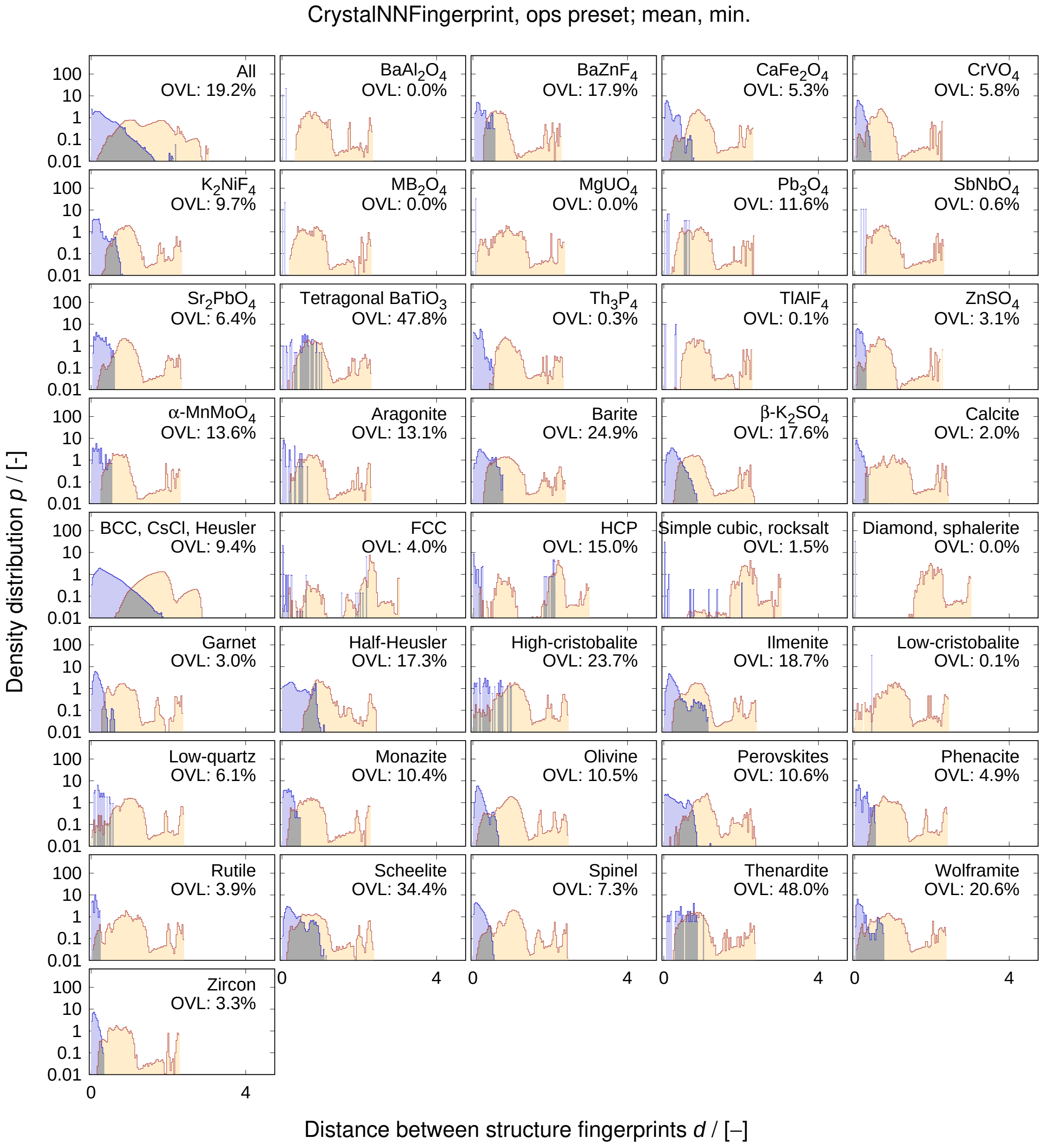

Fig. 66 Additional structure group (dis)similarity results.

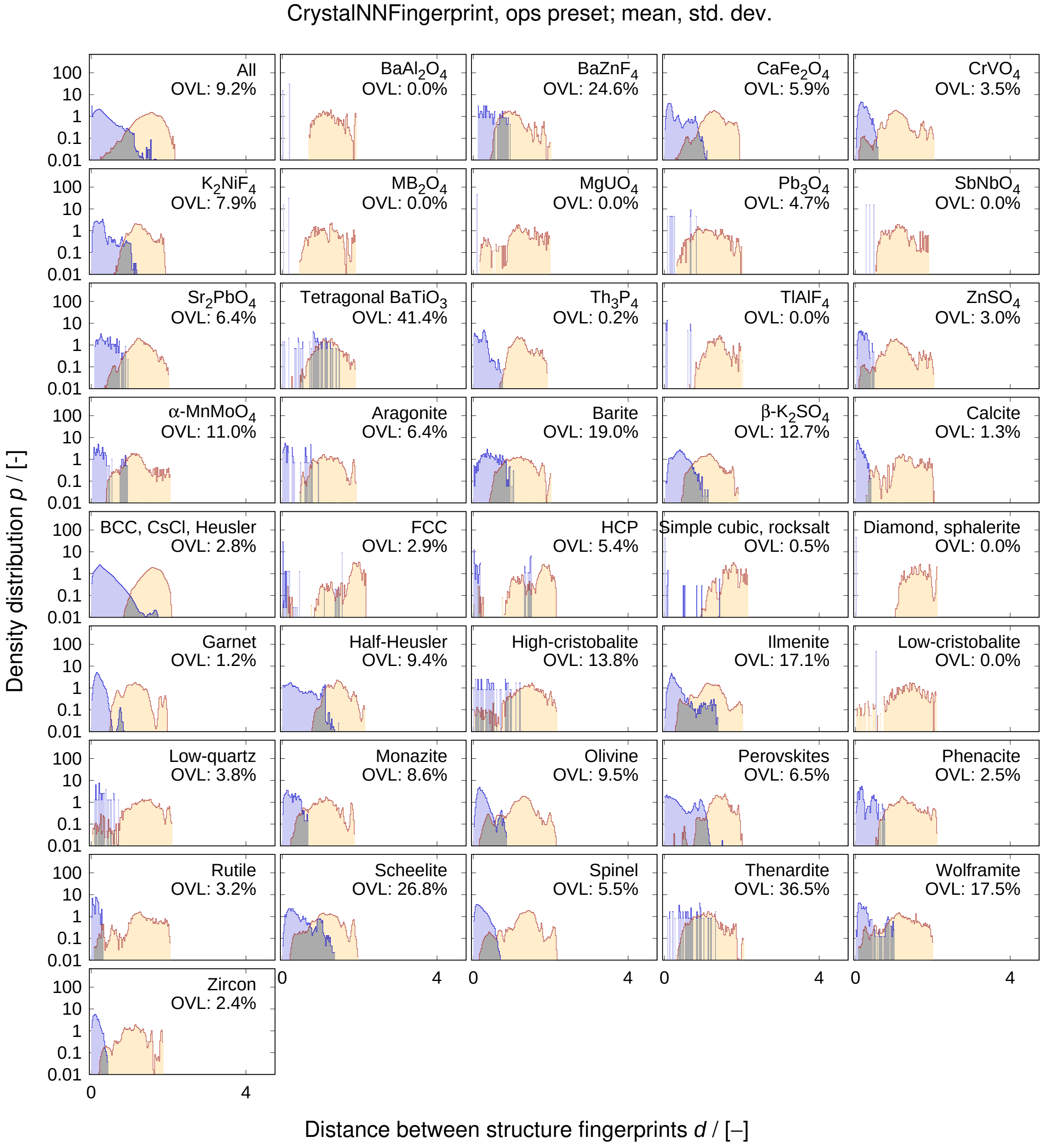

Fig. 67 Additional structure group (dis)similarity results.

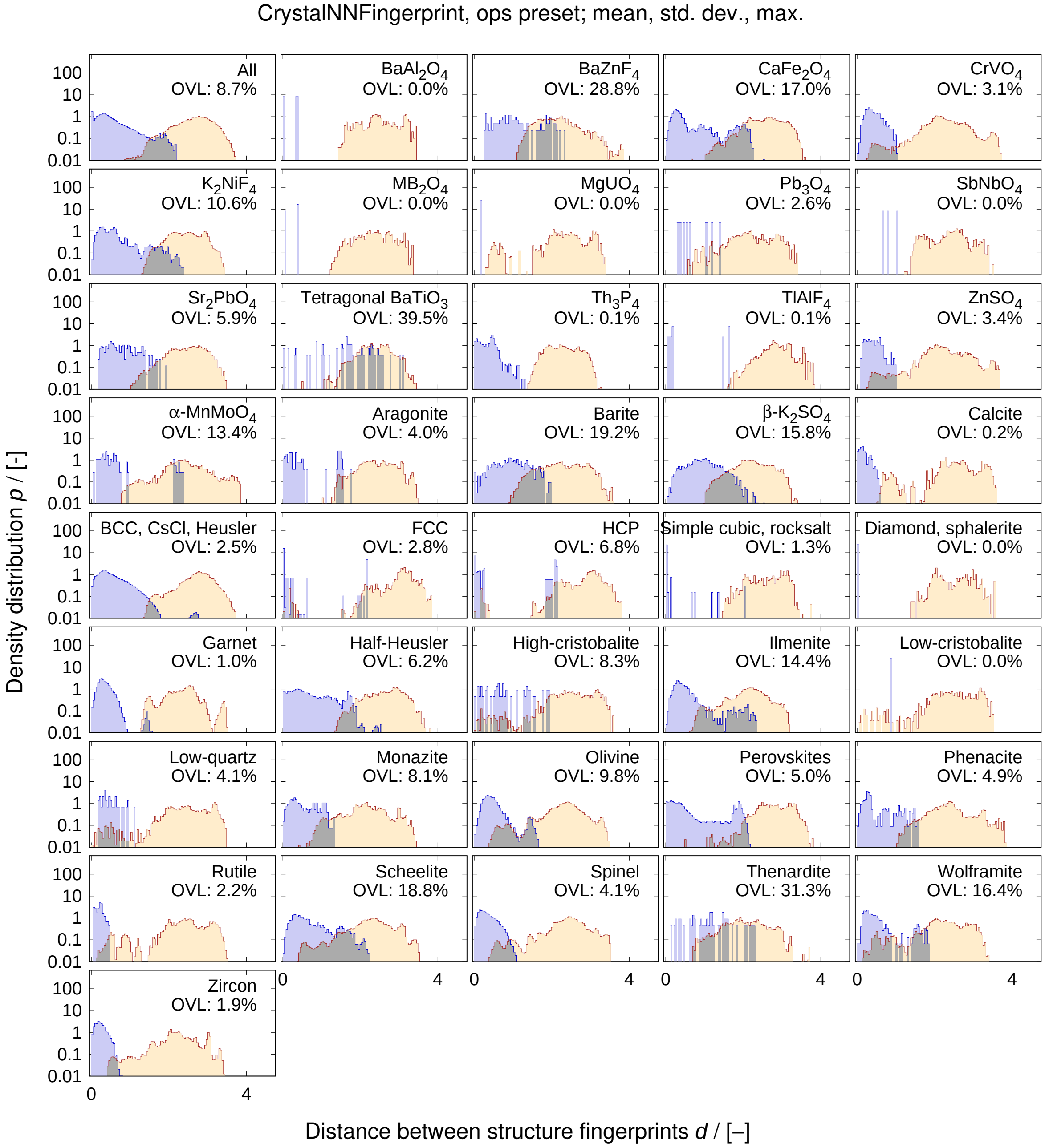

Fig. 68 Additional structure group (dis)similarity results.

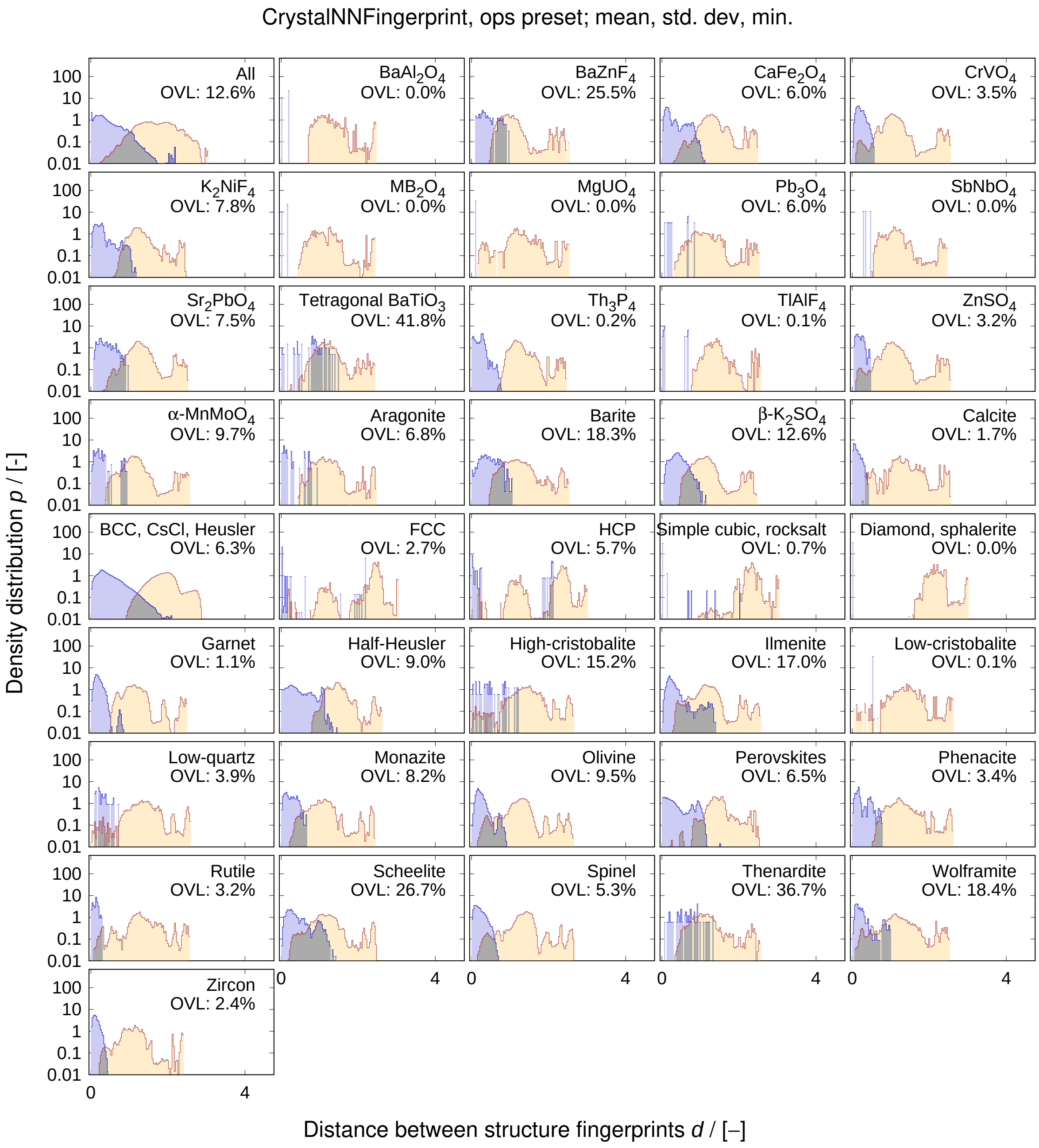

Fig. 69 Additional structure group (dis)similarity results.

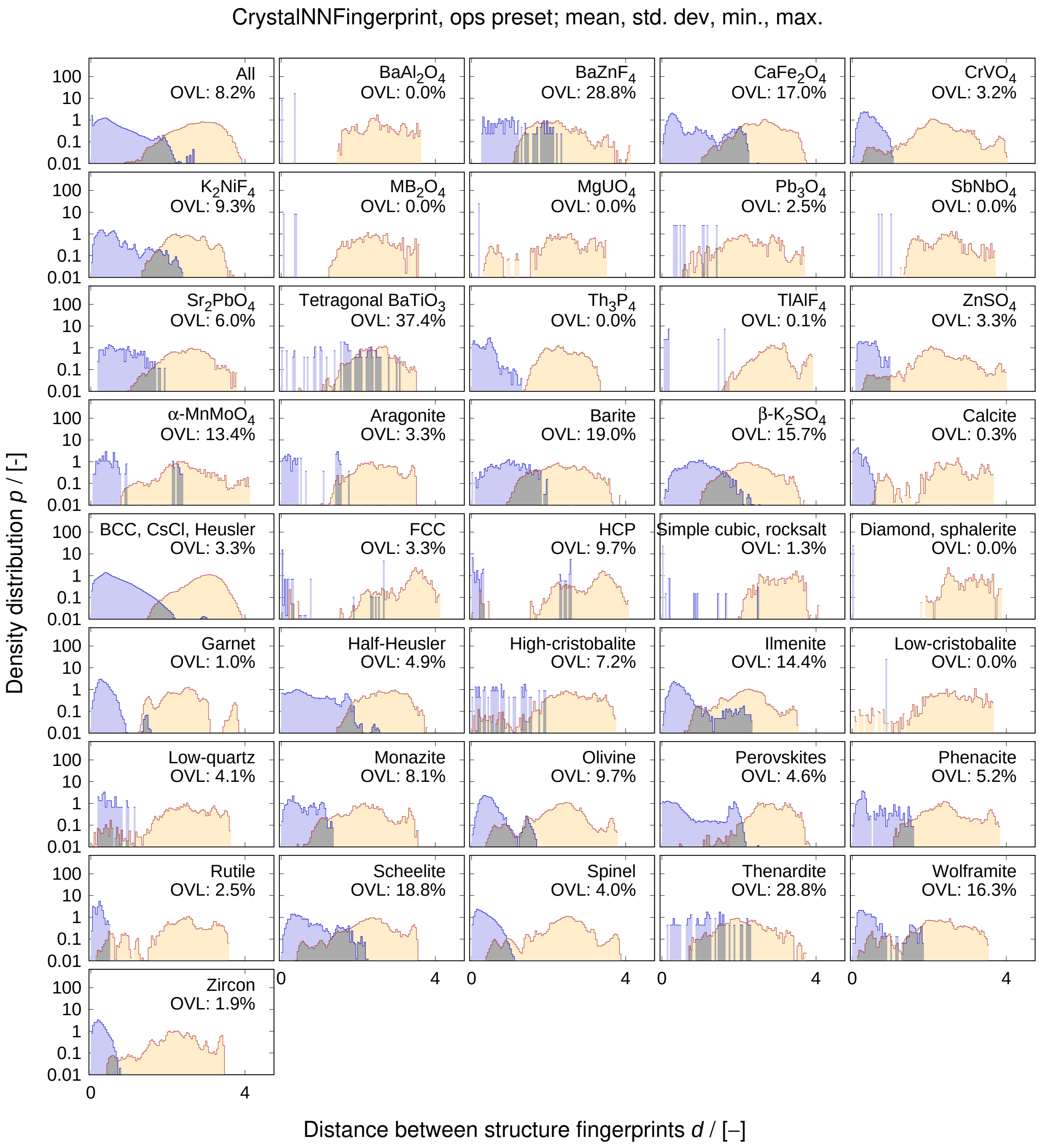

Fig. 70 Additional structure group (dis)similarity results.

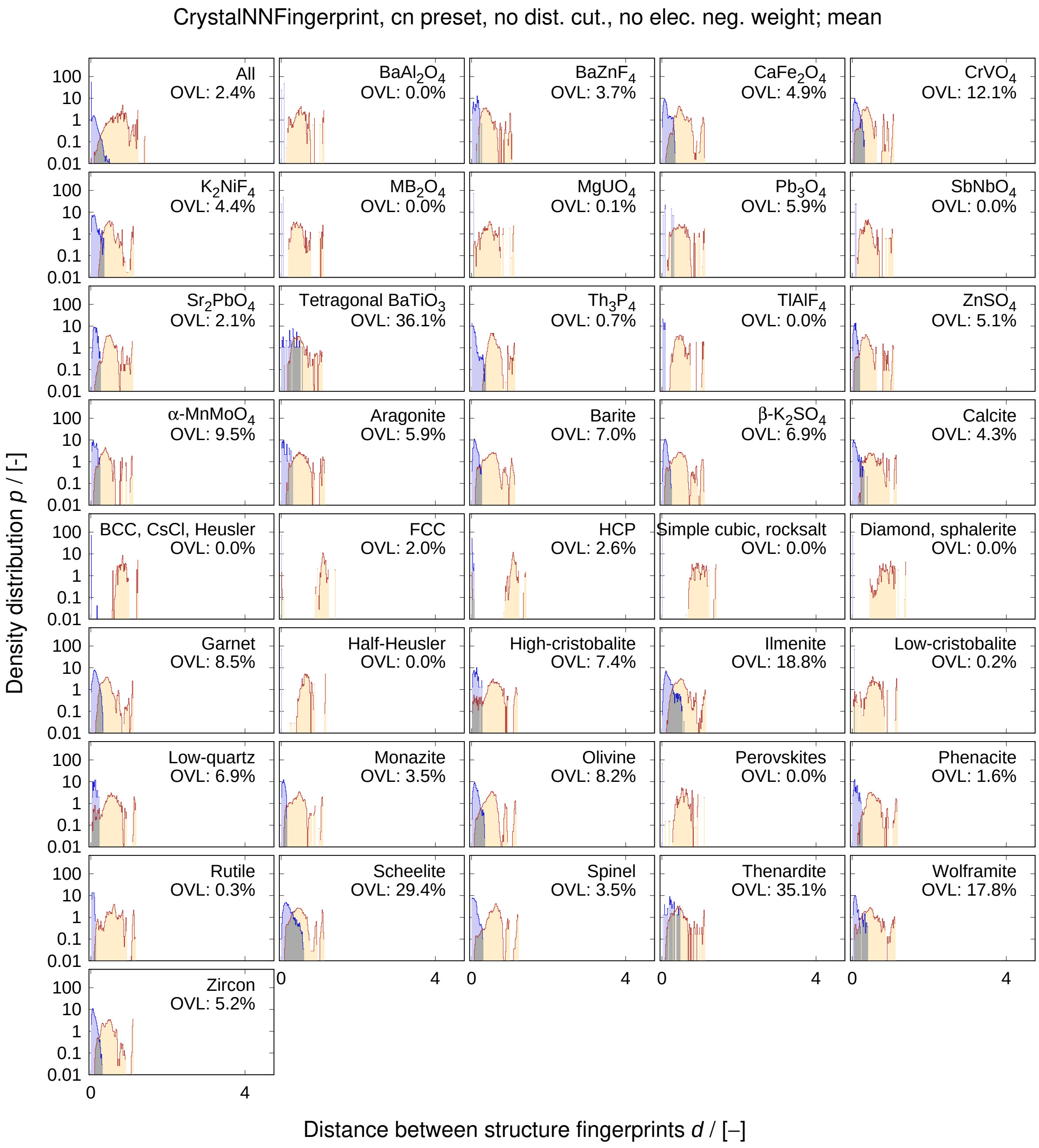

Fig. 71 Additional structure group (dis)similarity results.

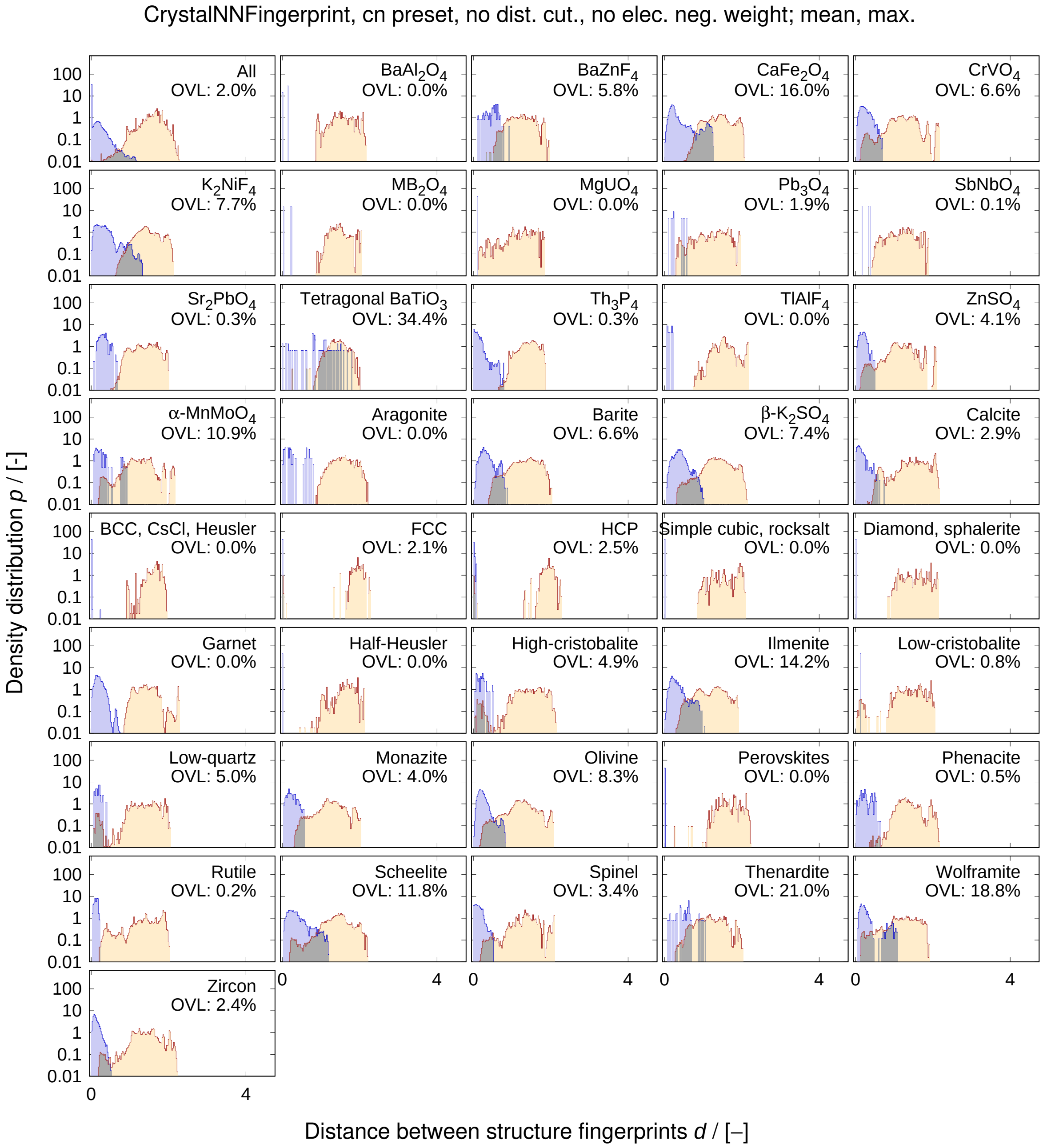

Fig. 72 Additional structure group (dis)similarity results.

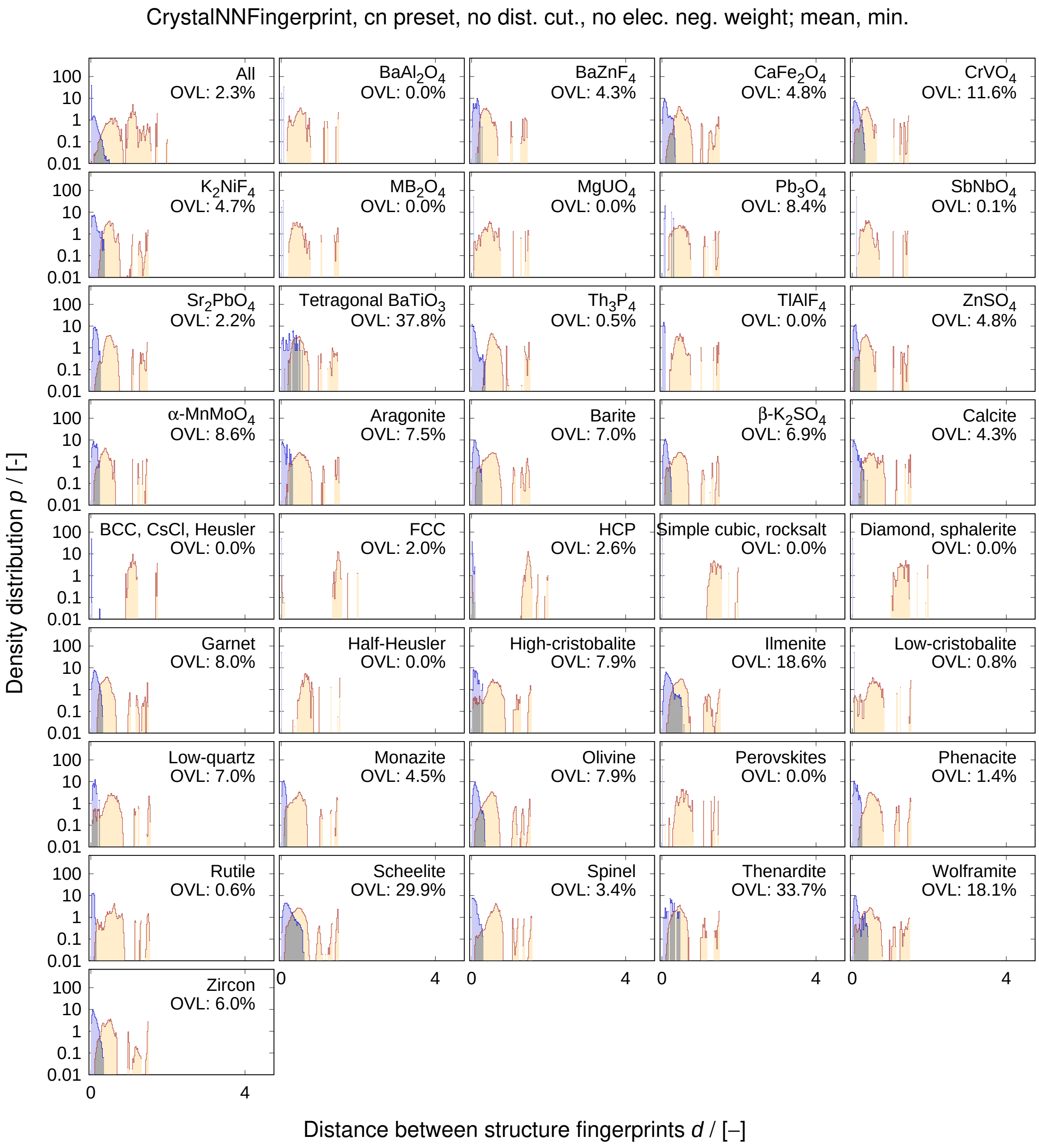

Fig. 73 Additional structure group (dis)similarity results.

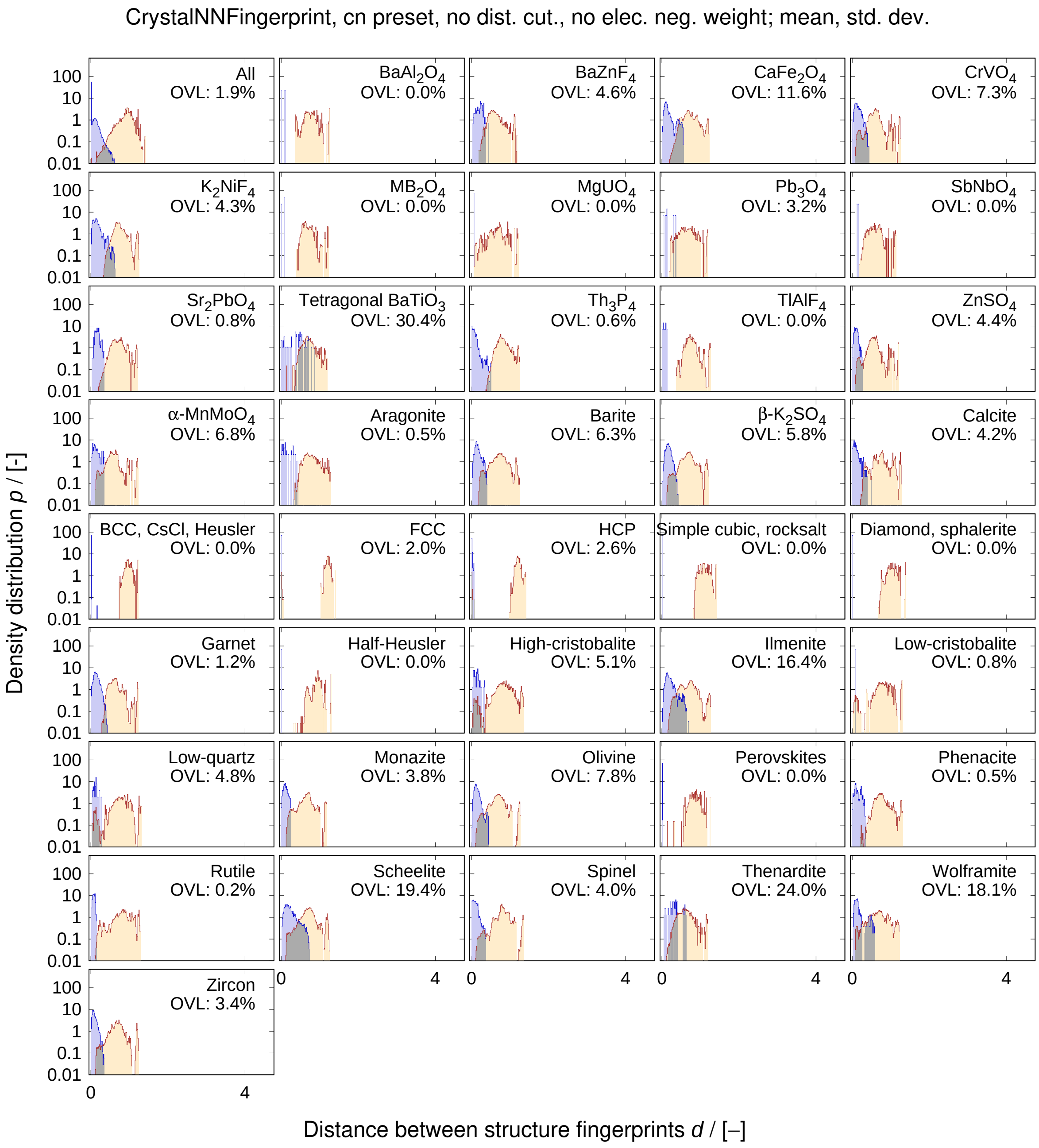

Fig. 74 Additional structure group (dis)similarity results.

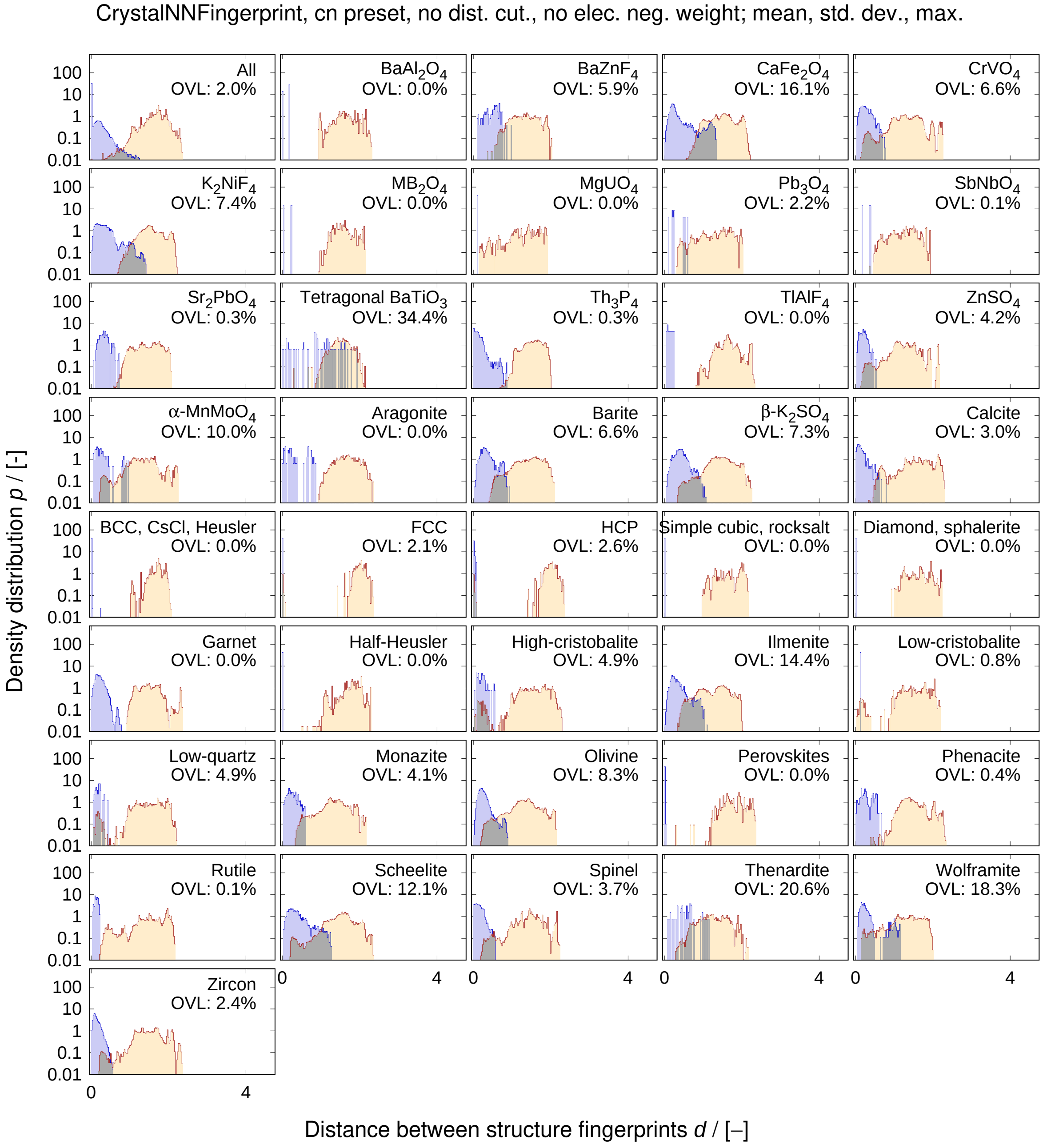

Fig. 75 Additional structure group (dis)similarity results.

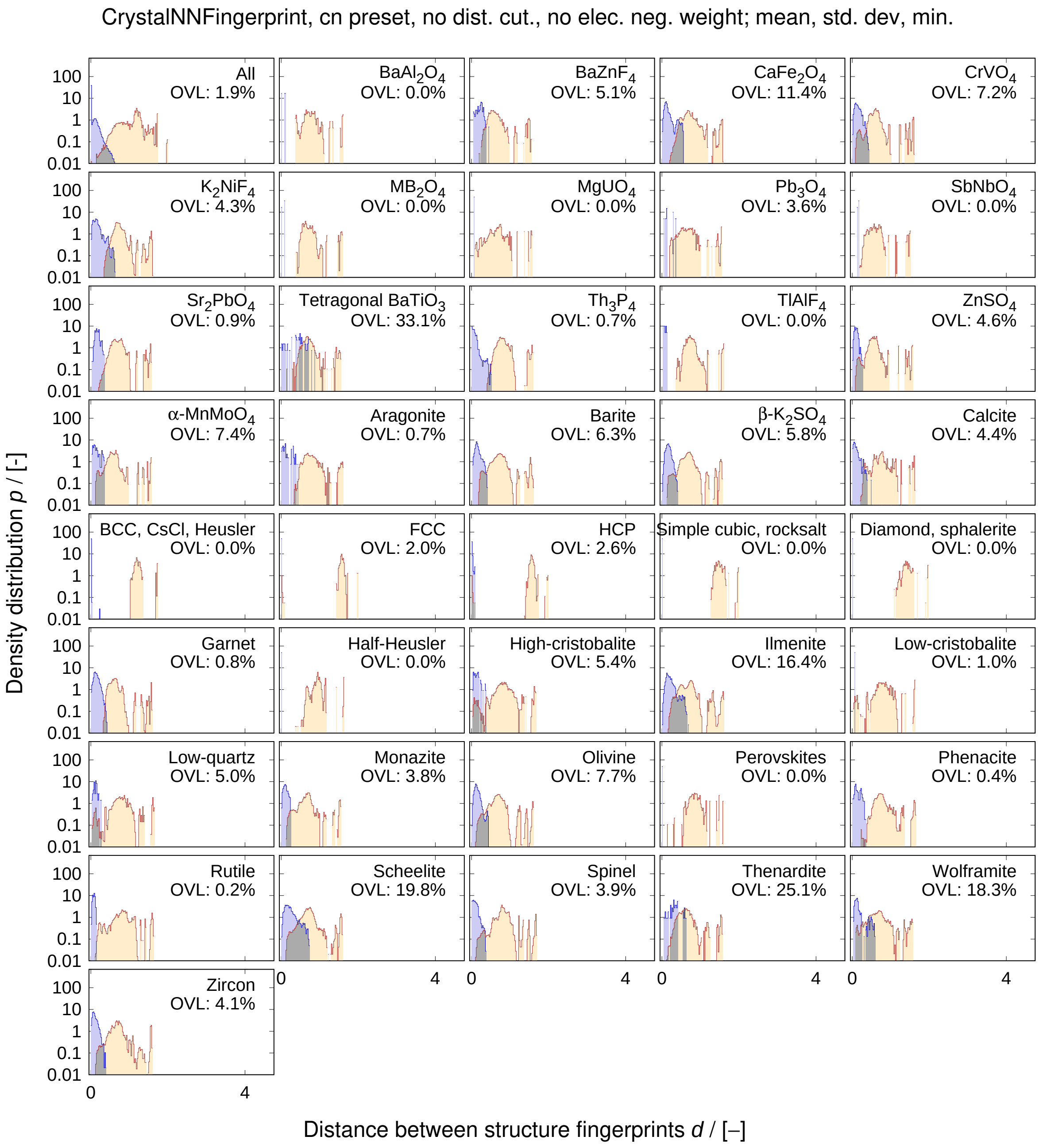

Fig. 76 Additional structure group (dis)similarity results.

CrystalNNFingerprint, cn preset, no dist. cut., no elec. neg. weight; mean, std. dev, min., max.

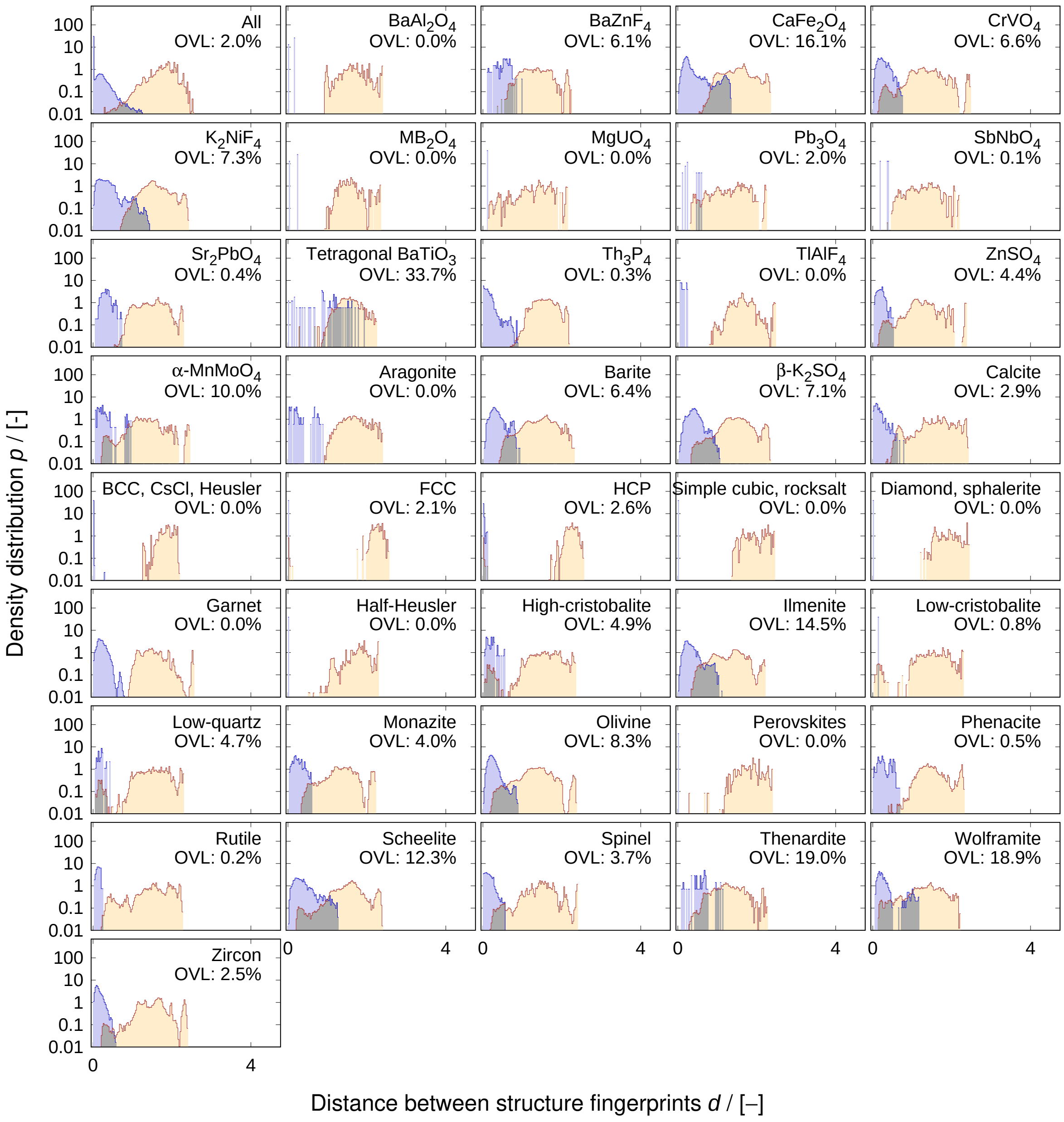

Fig. 77 Additional structure group (dis)similarity results.

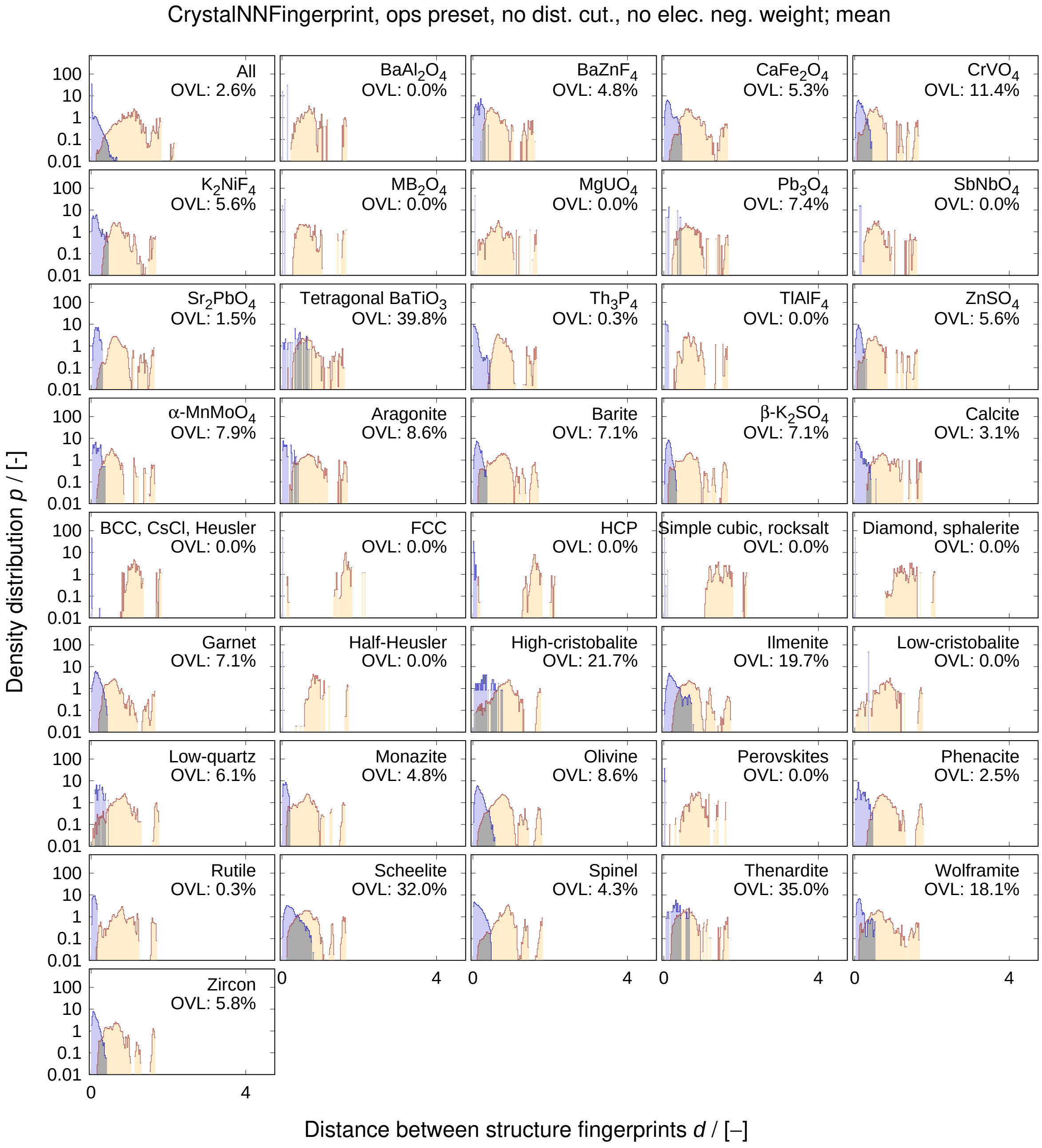

Fig. 78 Additional structure group (dis)similarity results.

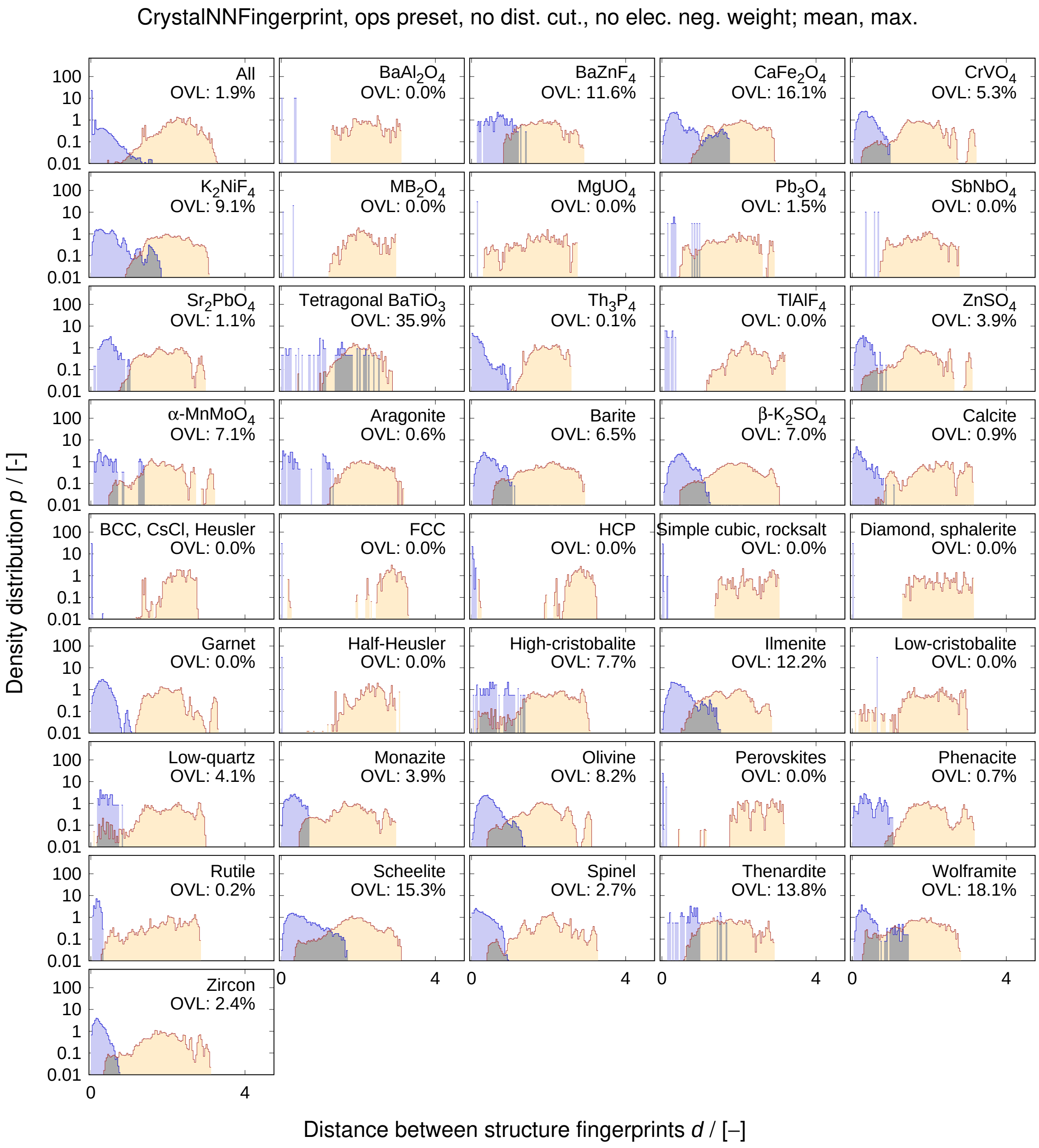

Fig. 79 Additional structure group (dis)similarity results.

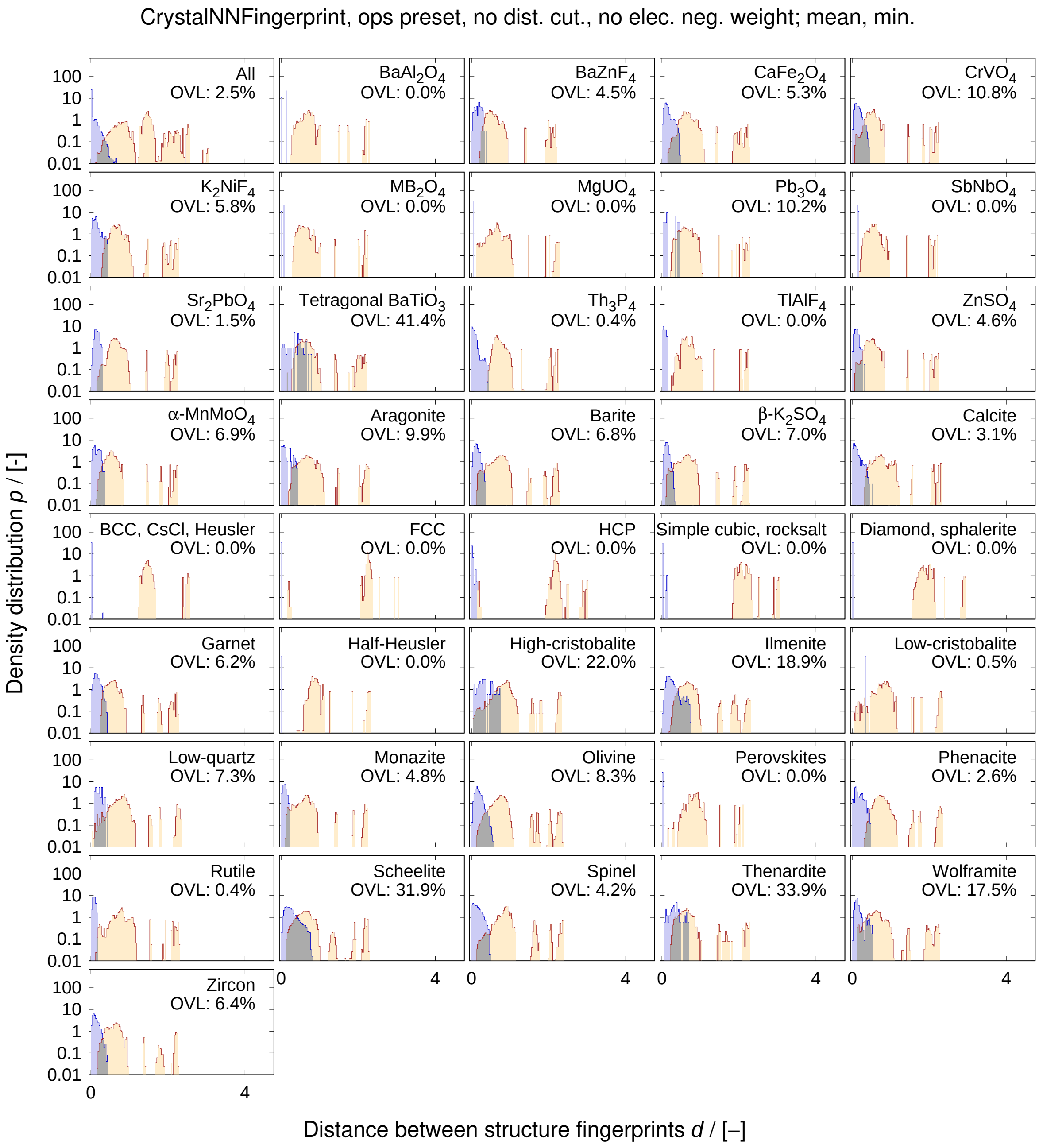

Fig. 80 Additional structure group (dis)similarity results.

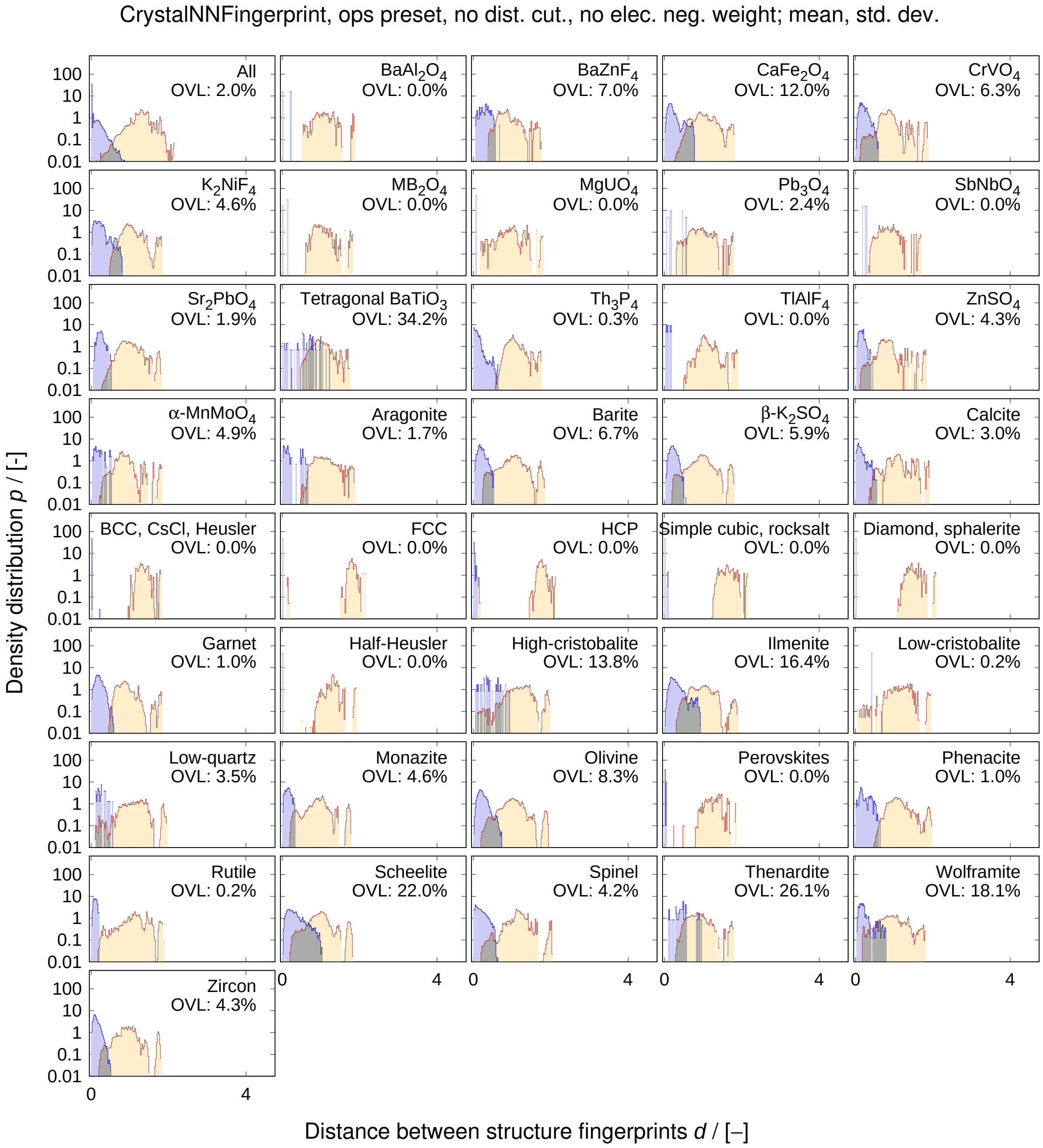

Fig. 81 Additional structure group (dis)similarity results.

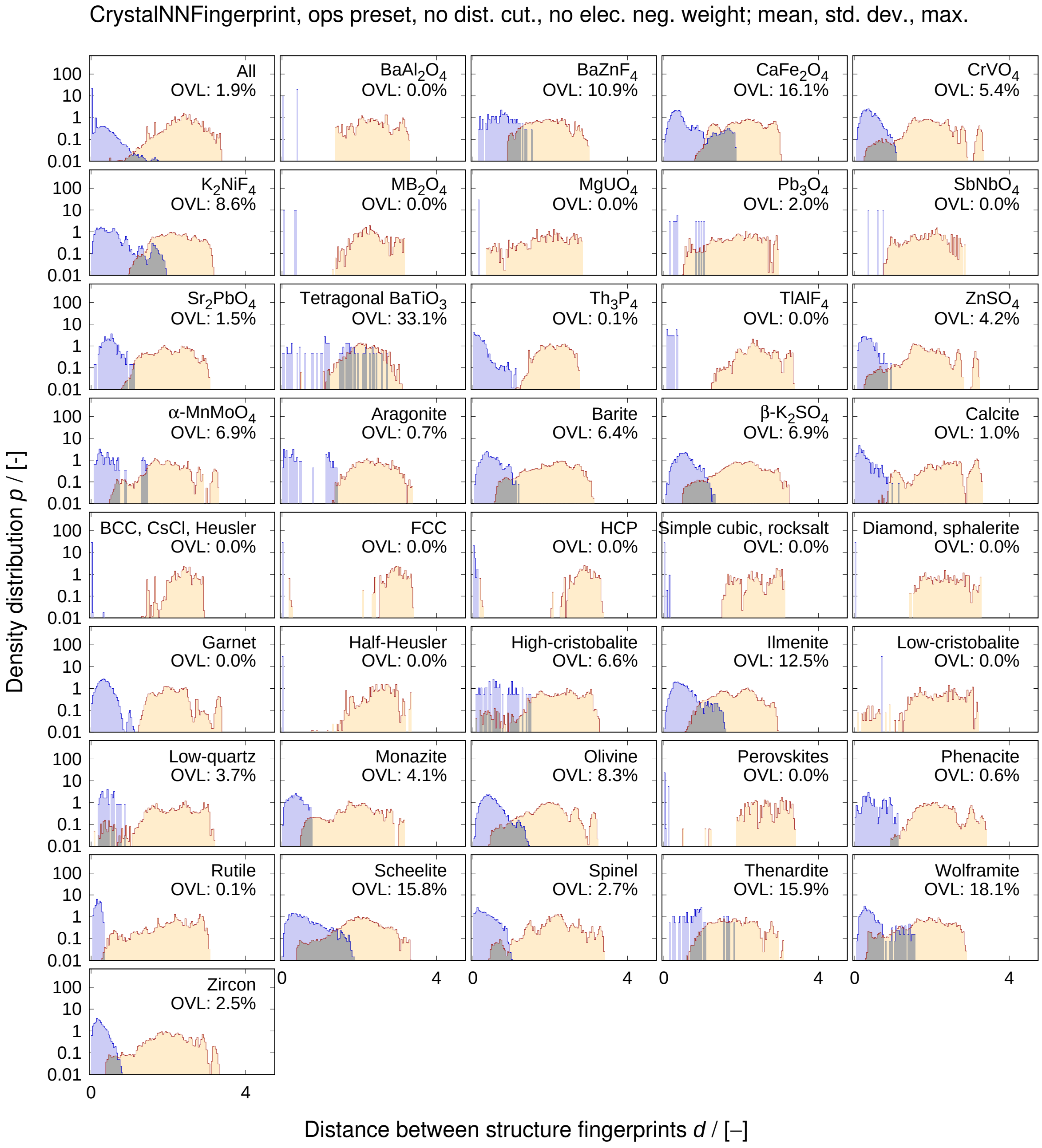

Fig. 82 Additional structure group (dis)similarity results.

CrystalNNFingerprint, ops preset, no dist. cut., no elec. neg. weight; mean, std. dev, min.

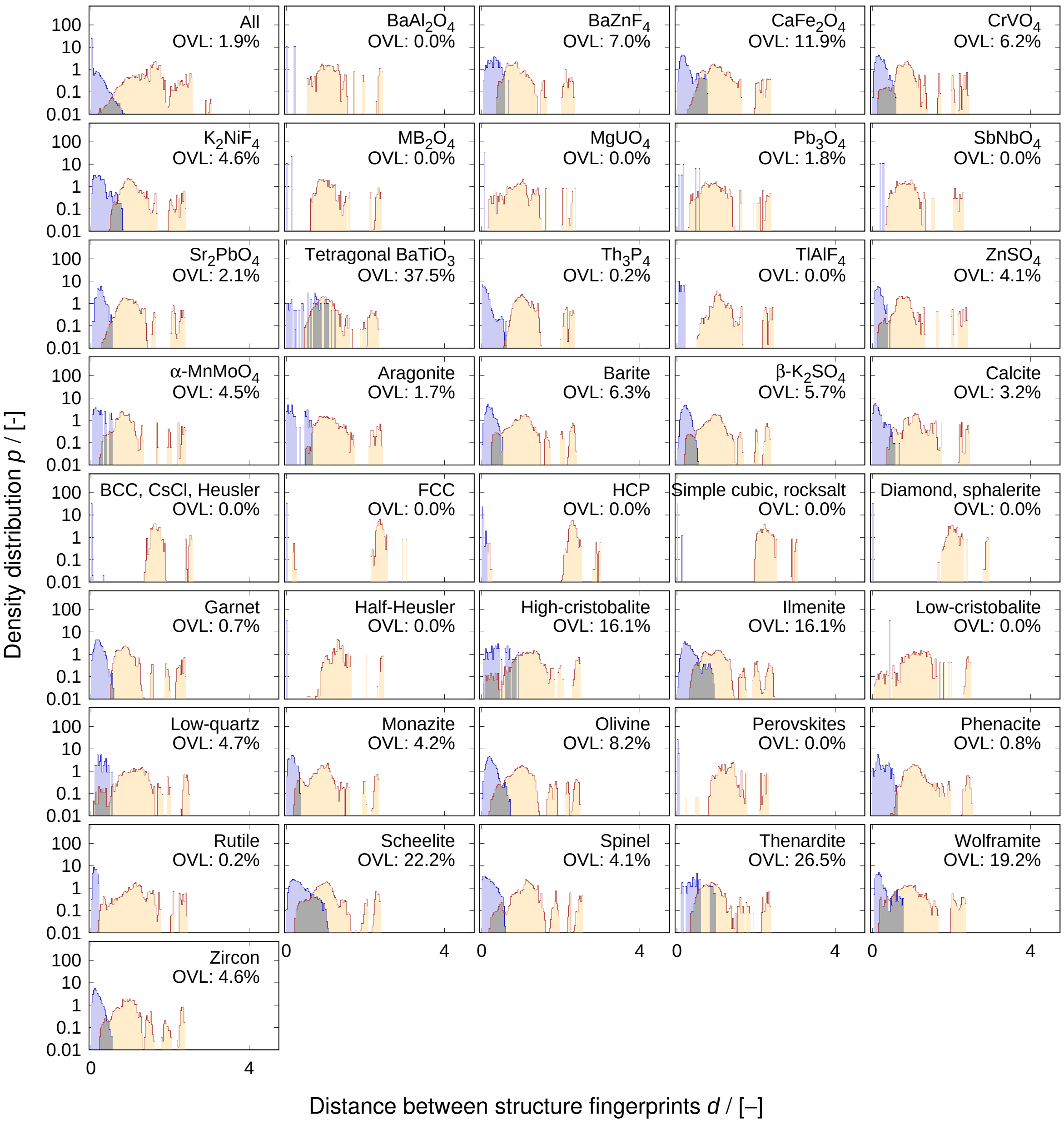

Fig. 83 Additional structure group (dis)similarity results.

CrystalNNFingerprint, ops preset, no dist. cut., no elec. neg. weight; mean, std. dev, min., max.

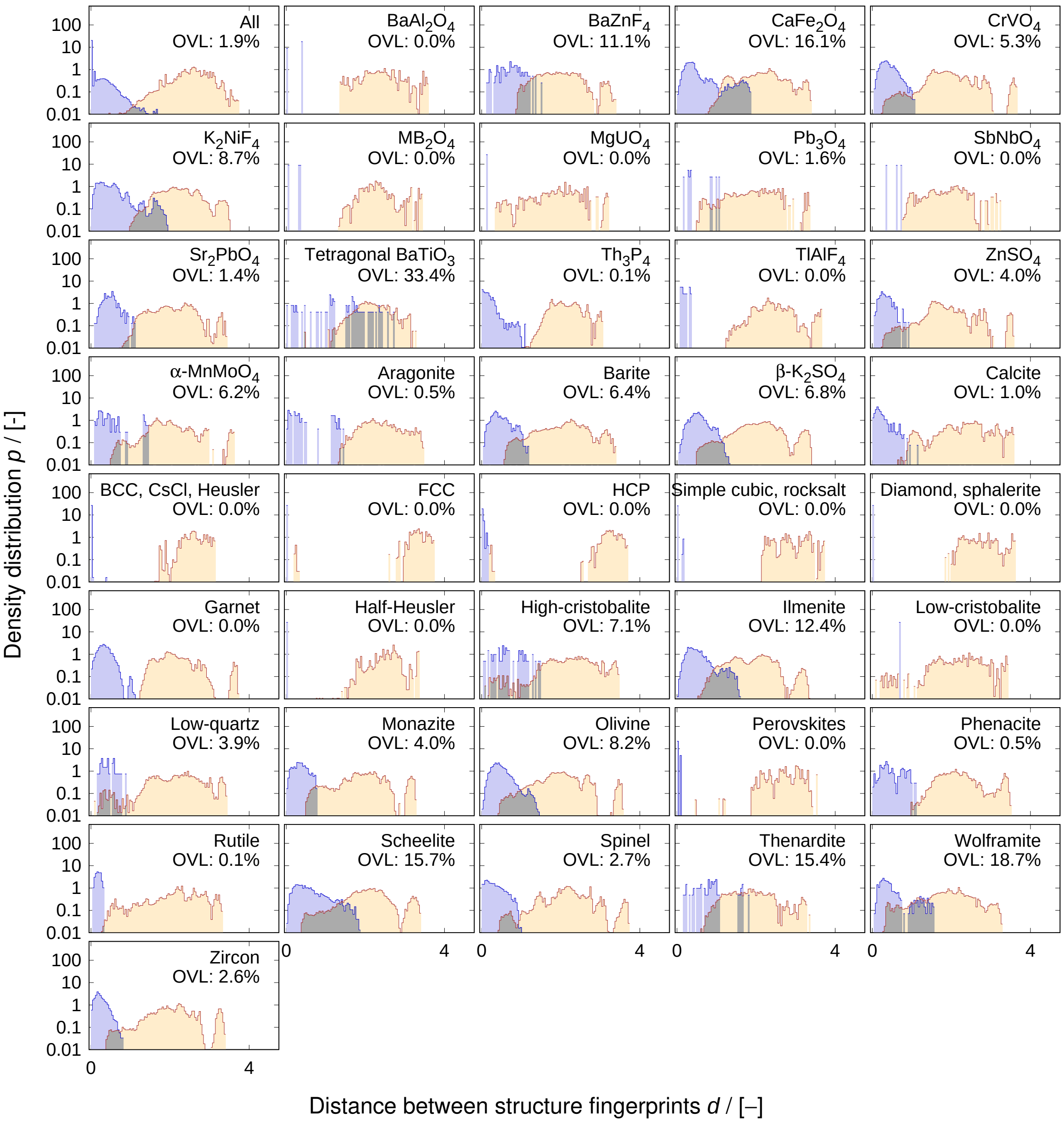

Fig. 84 Additional structure group (dis)similarity results.

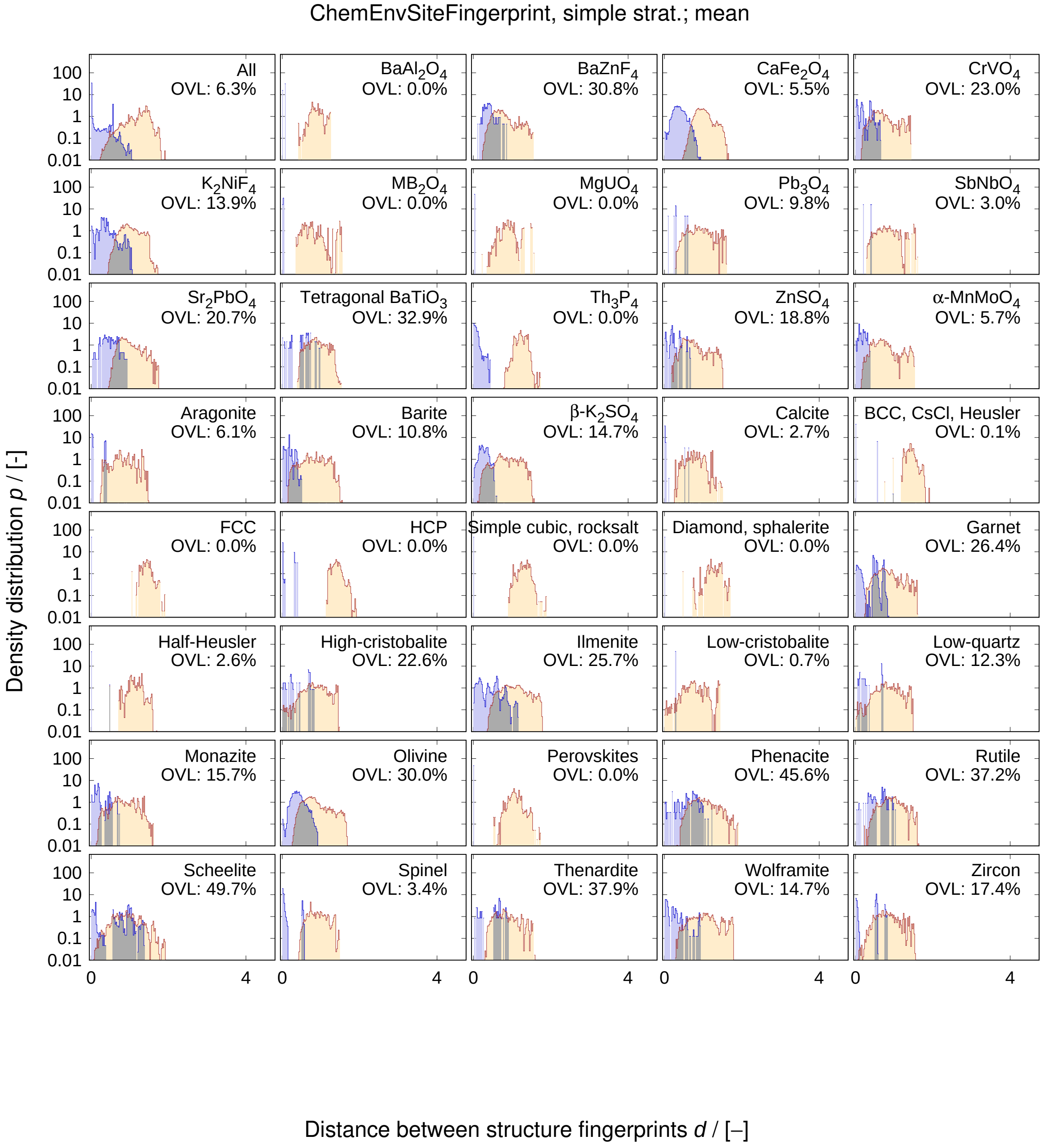

Fig. 85 Additional structure group (dis)similarity results.

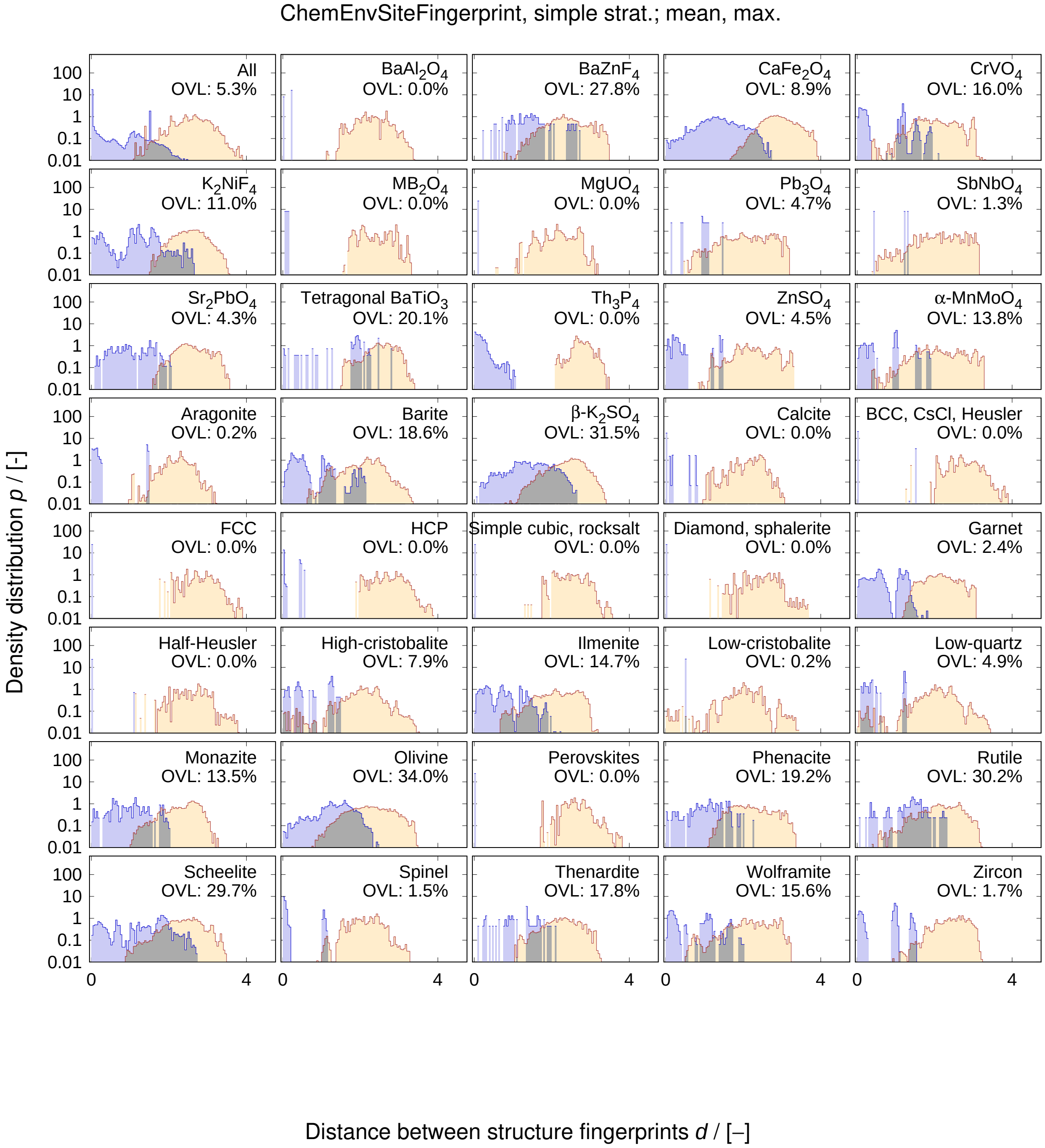

Fig. 86 Additional structure group (dis)similarity results.

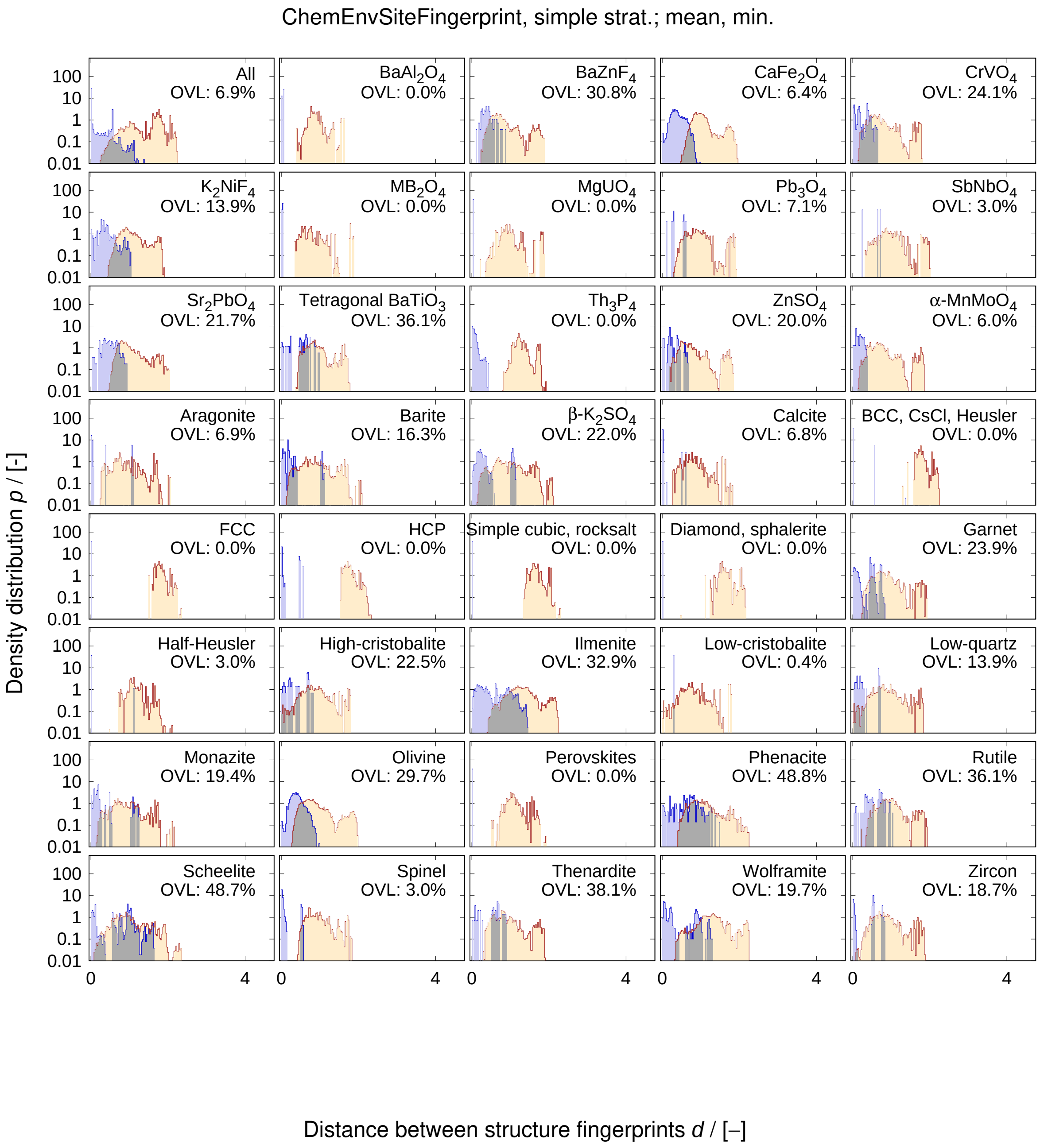

Fig. 87 Additional structure group (dis)similarity results.

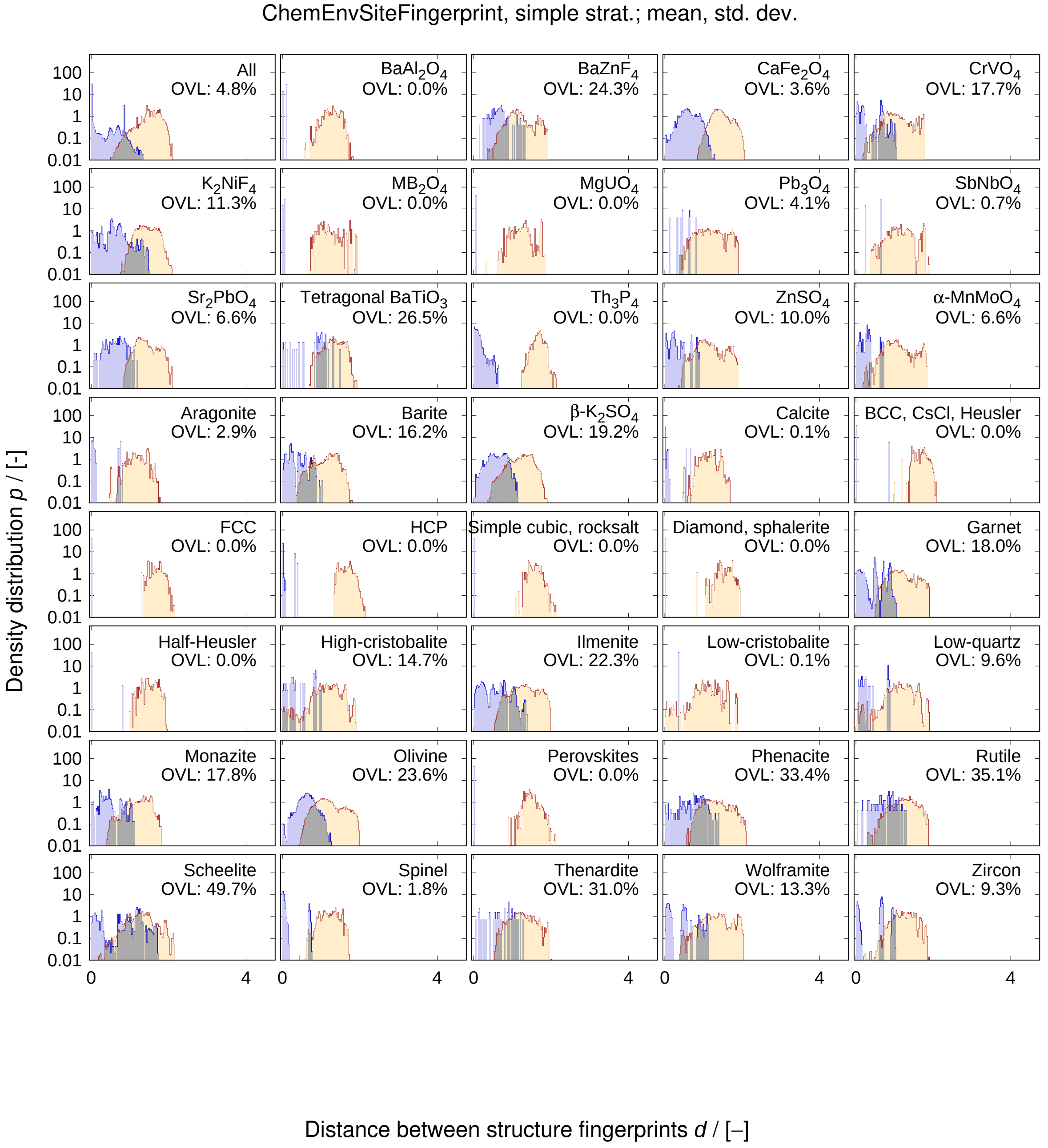

Fig. 88 Additional structure group (dis)similarity results.

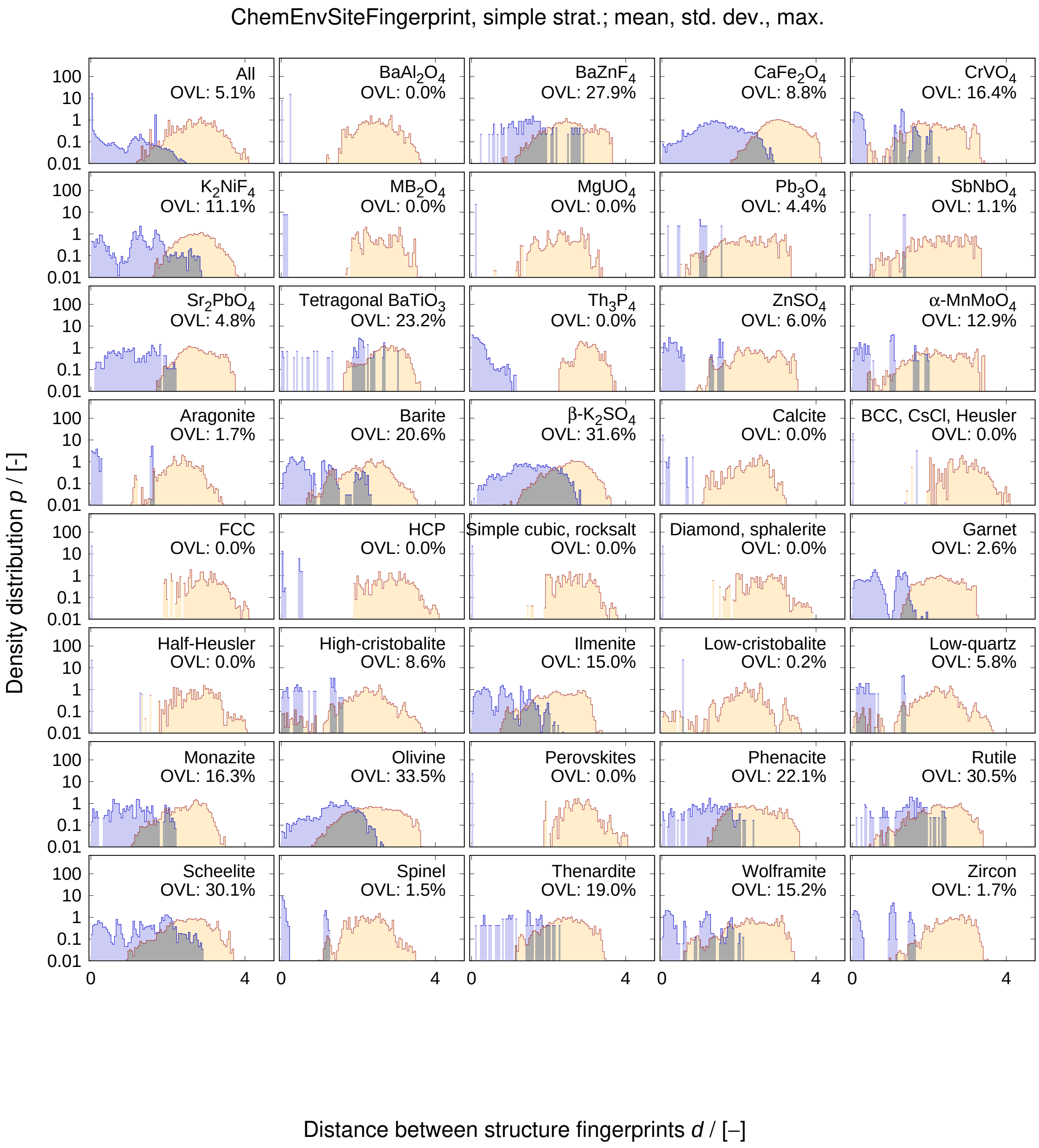

Fig. 89 Additional structure group (dis)similarity results.

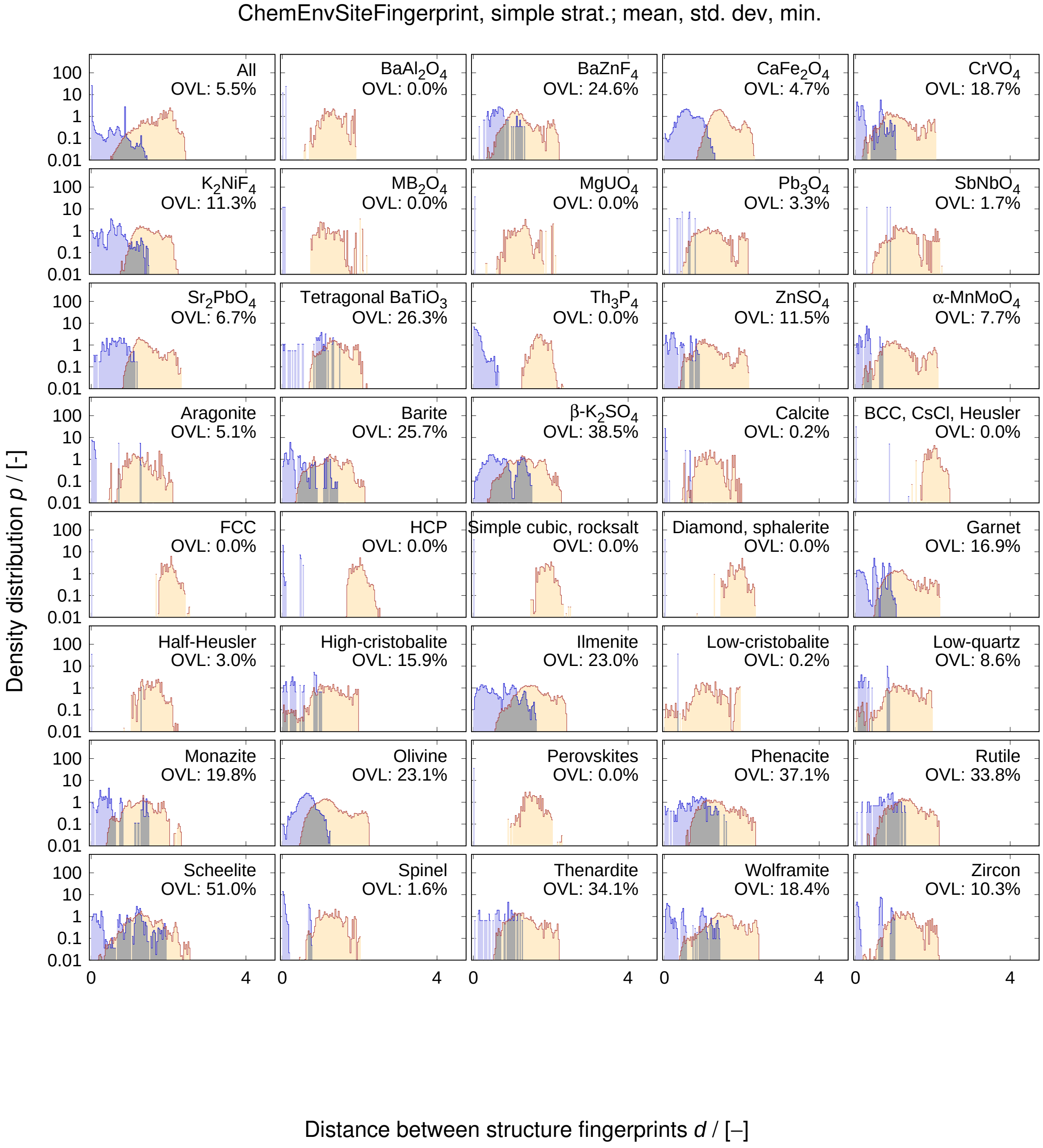

Fig. 90 Additional structure group (dis)similarity results.

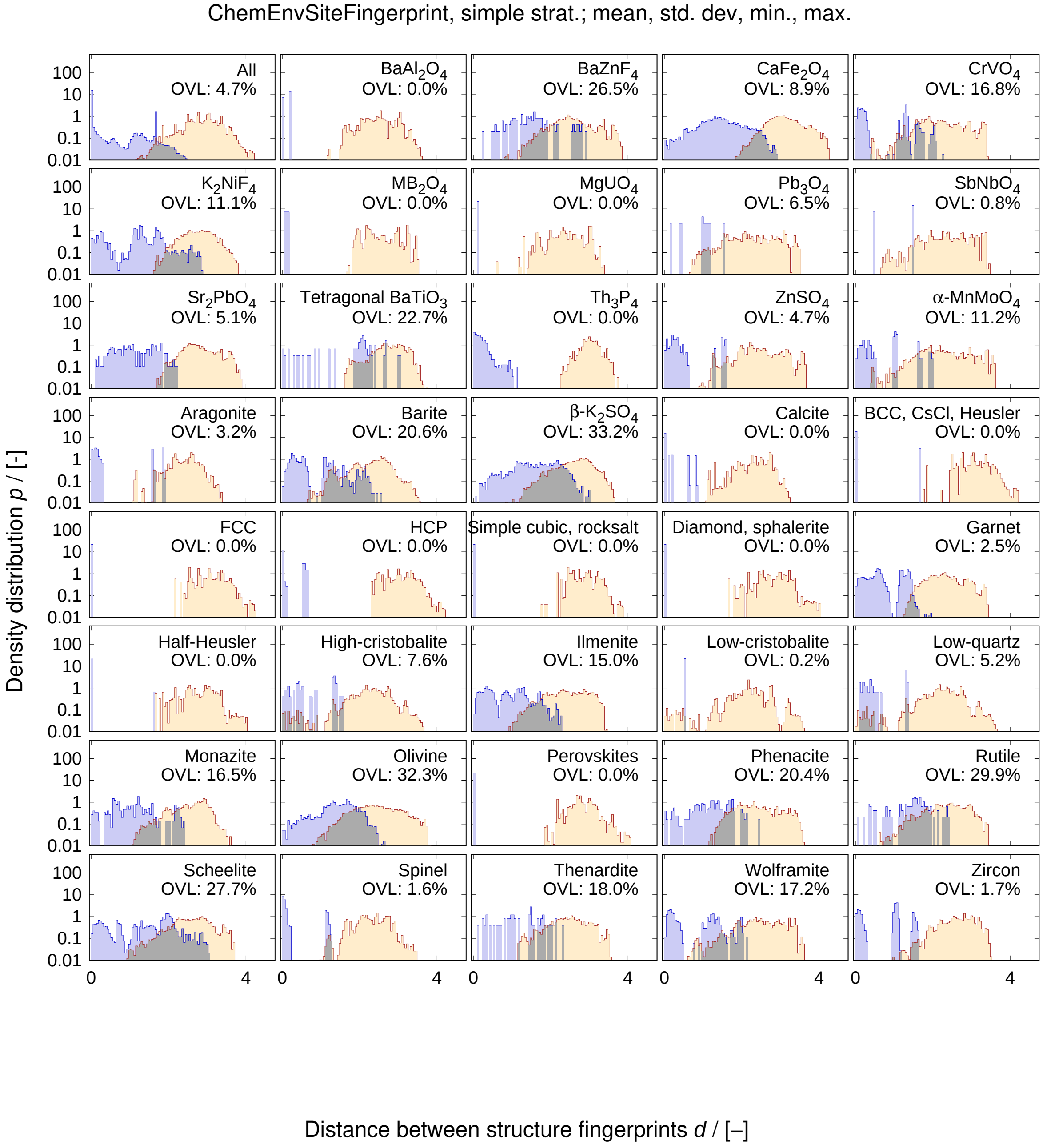

Fig. 91 Additional structure group (dis)similarity results.

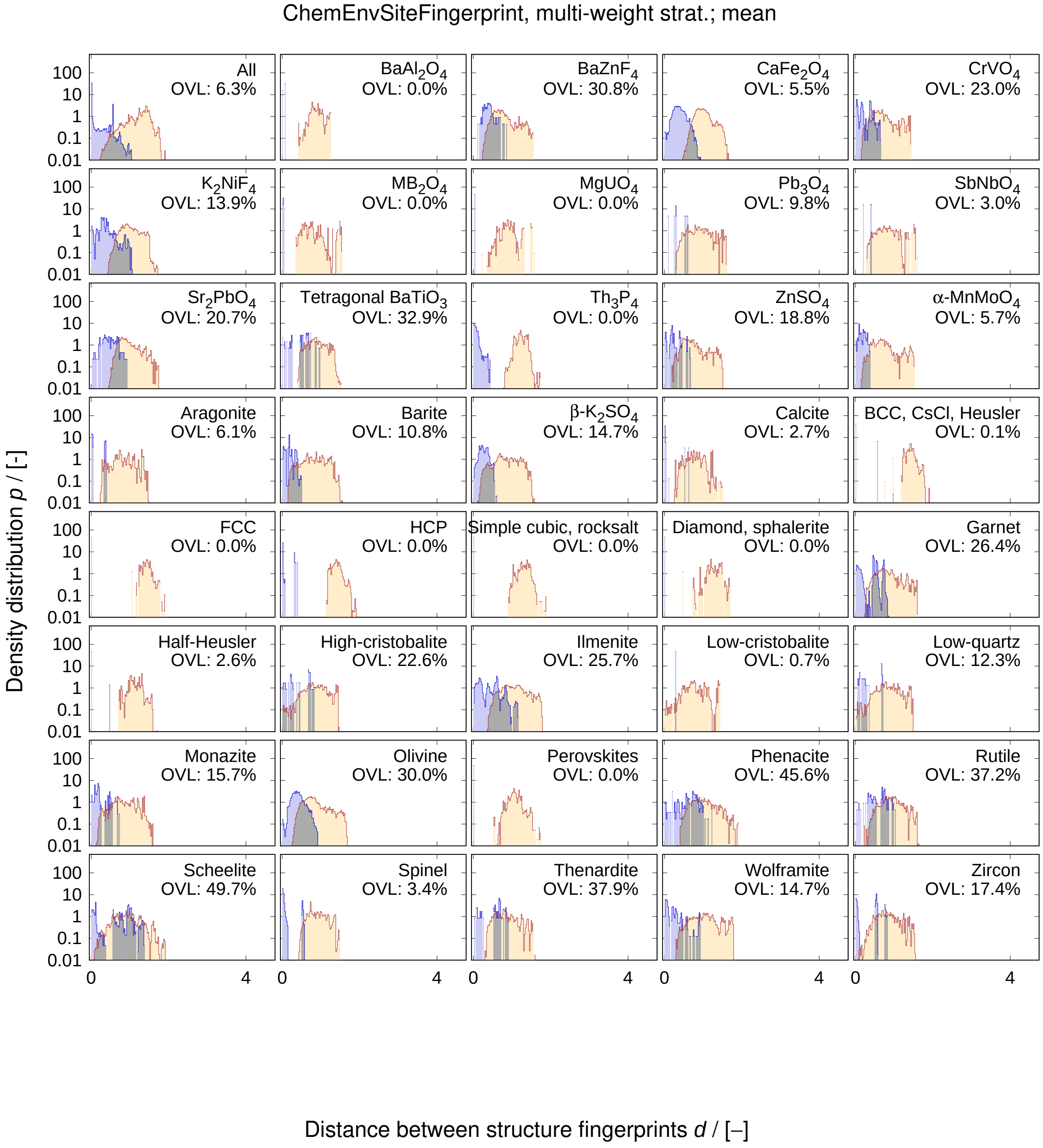

Fig. 92 Additional structure group (dis)similarity results.

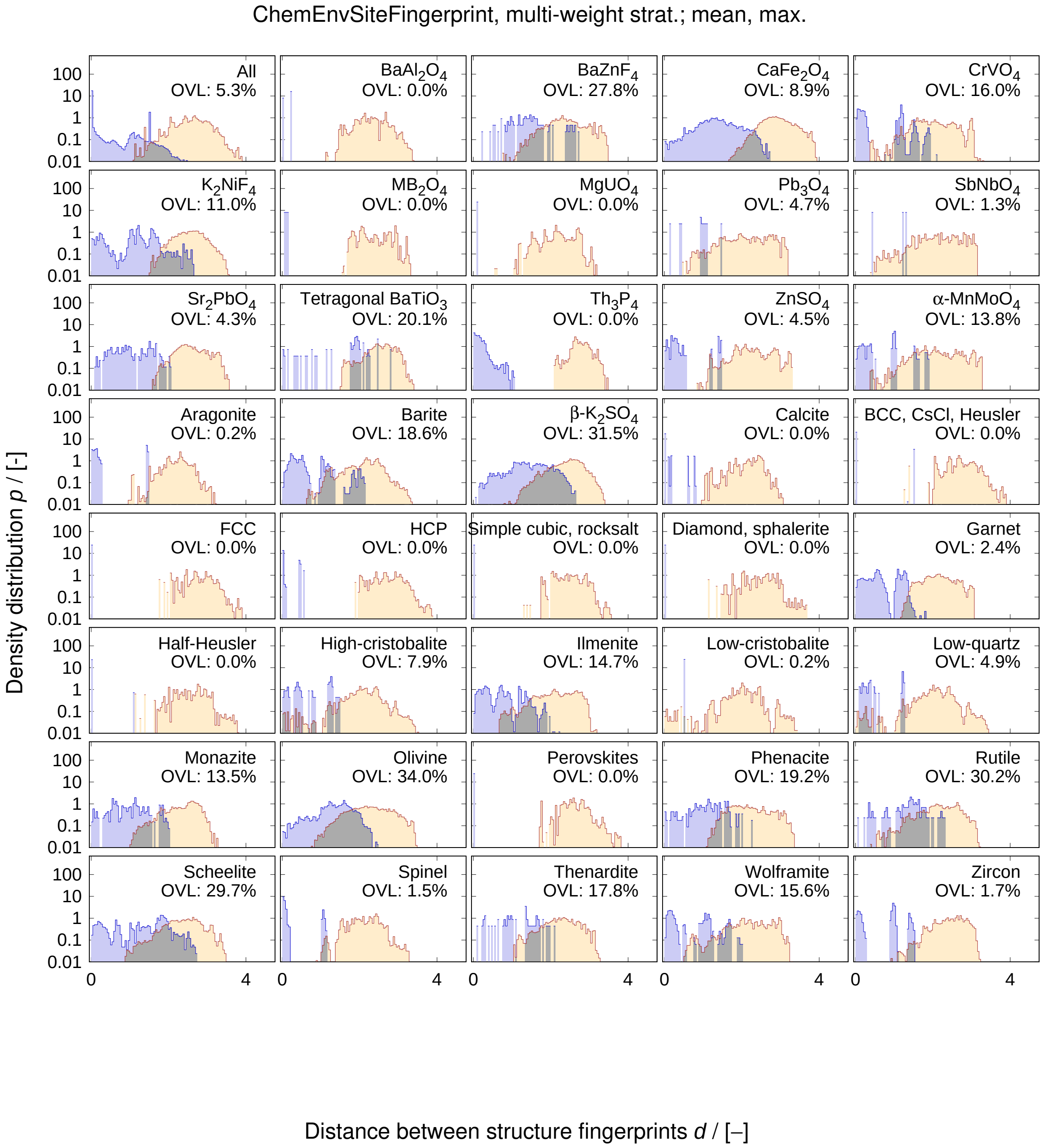

Fig. 93 Additional structure group (dis)similarity results.

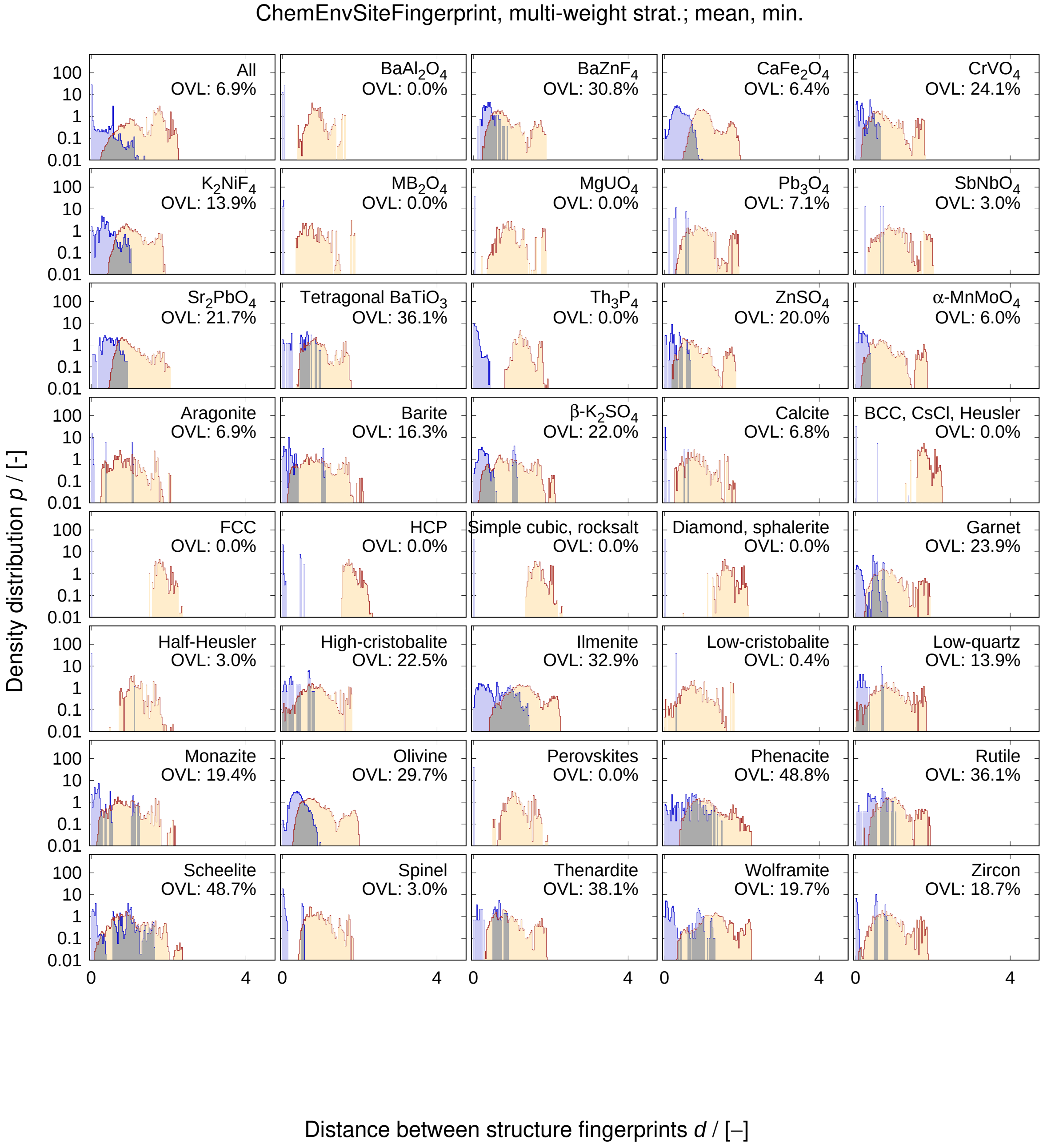

Fig. 94 Additional structure group (dis)similarity results.

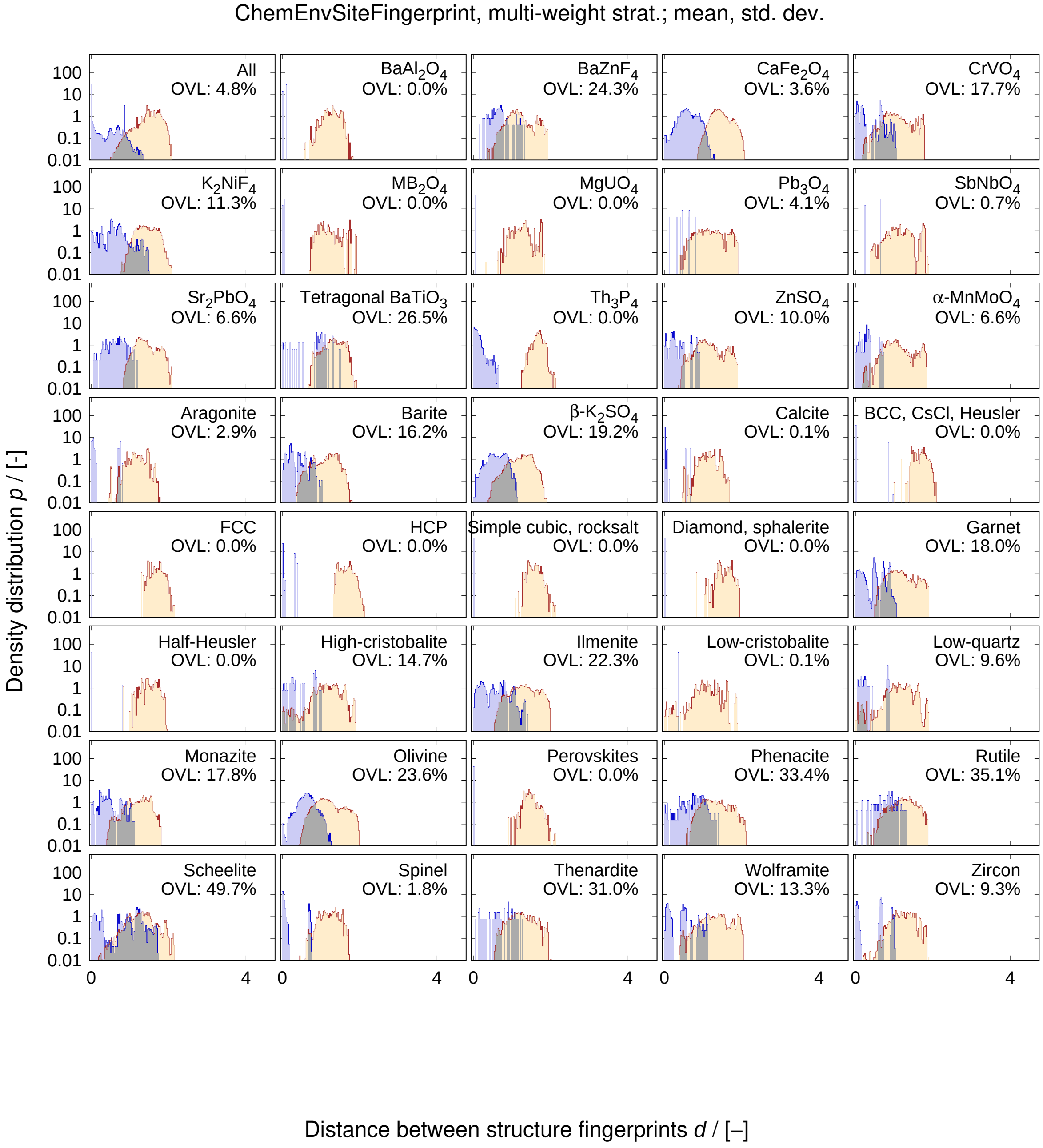

Fig. 95 Additional structure group (dis)similarity results.

ChemEnvSiteFingerprint, multi-weight strat.; mean, std. dev., max.

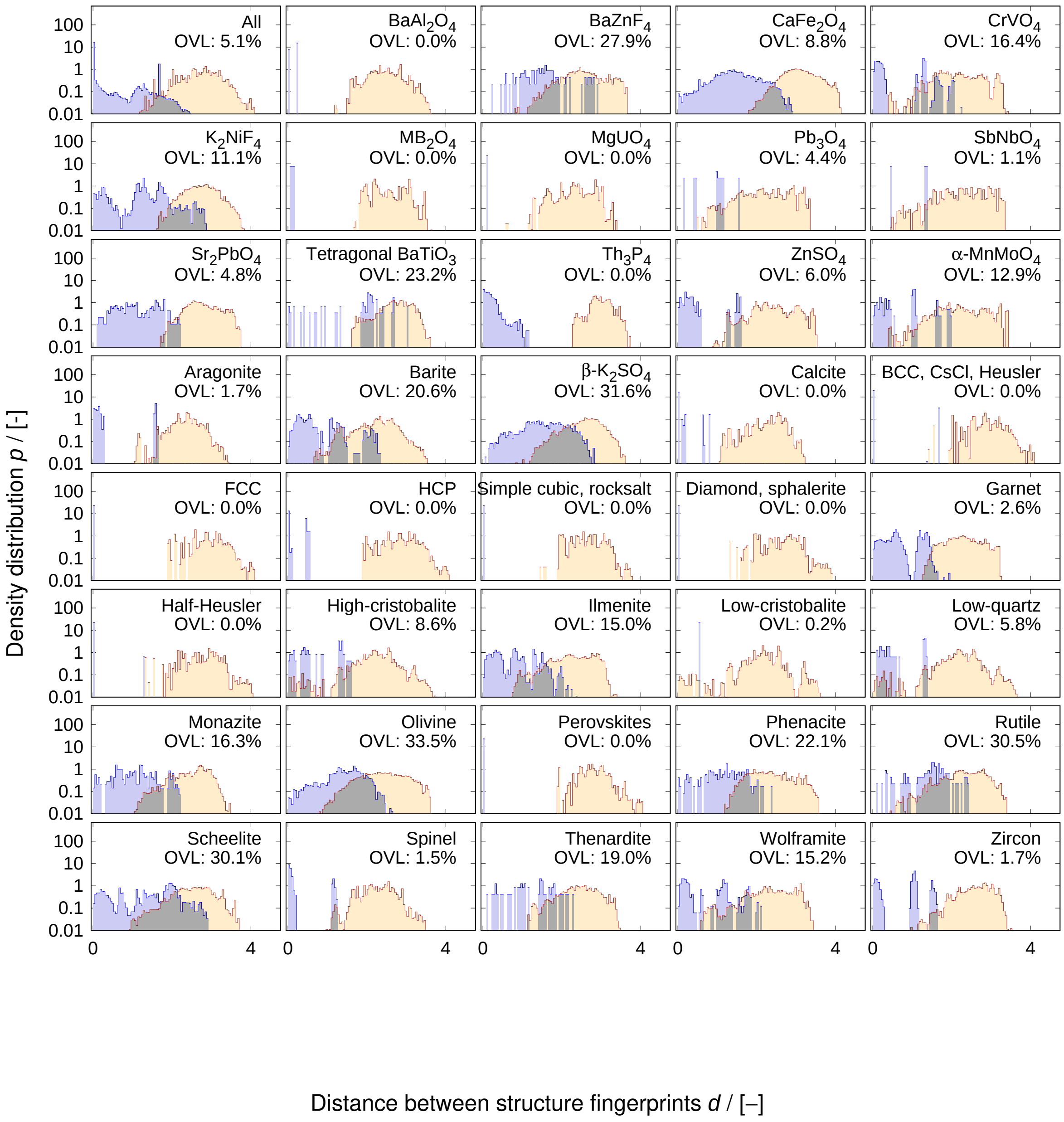

Fig. 96 Additional structure group (dis)similarity results.

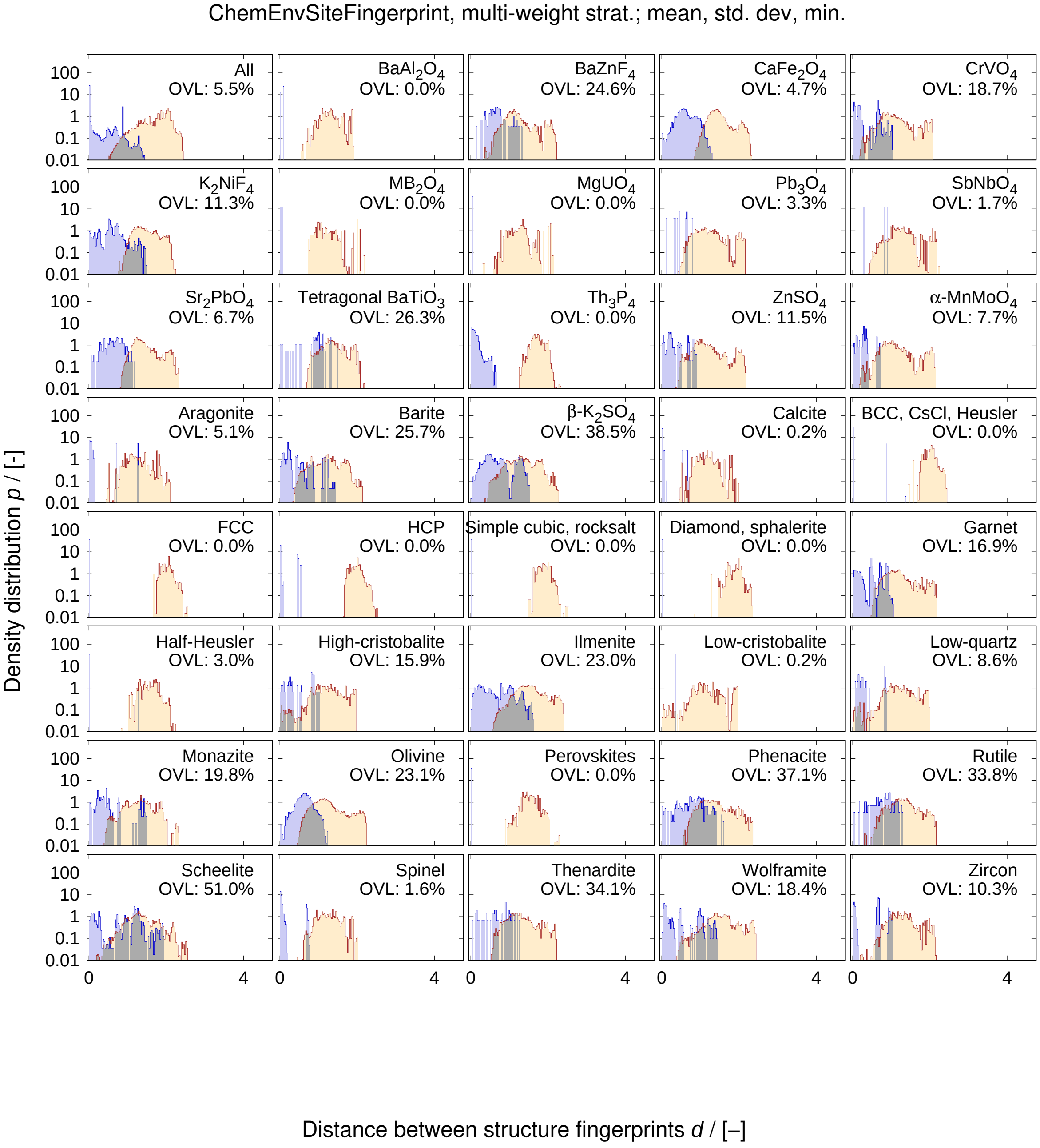

Fig. 97 Additional structure group (dis)similarity results.

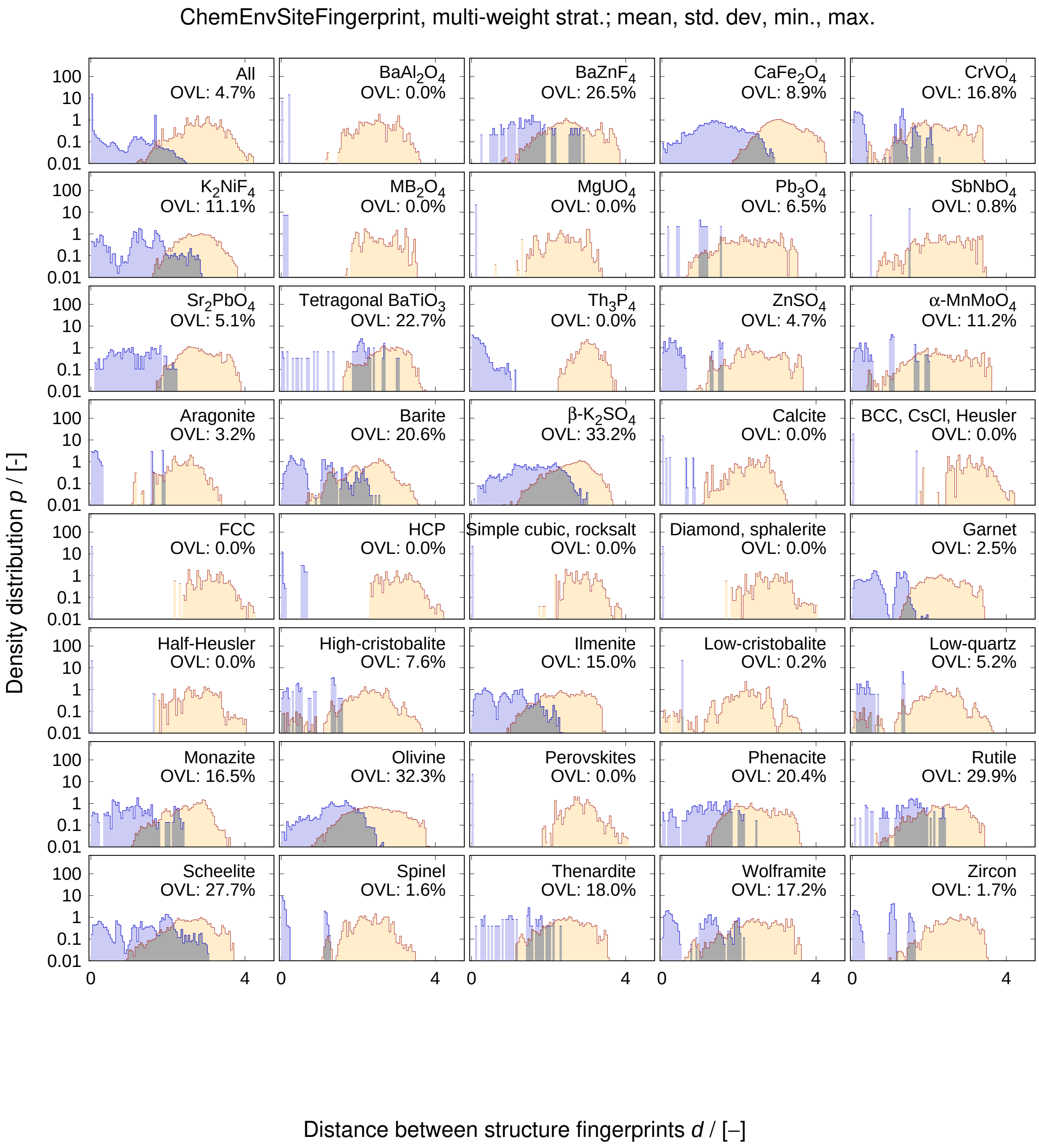

Fig. 98 Additional structure group (dis)similarity results.

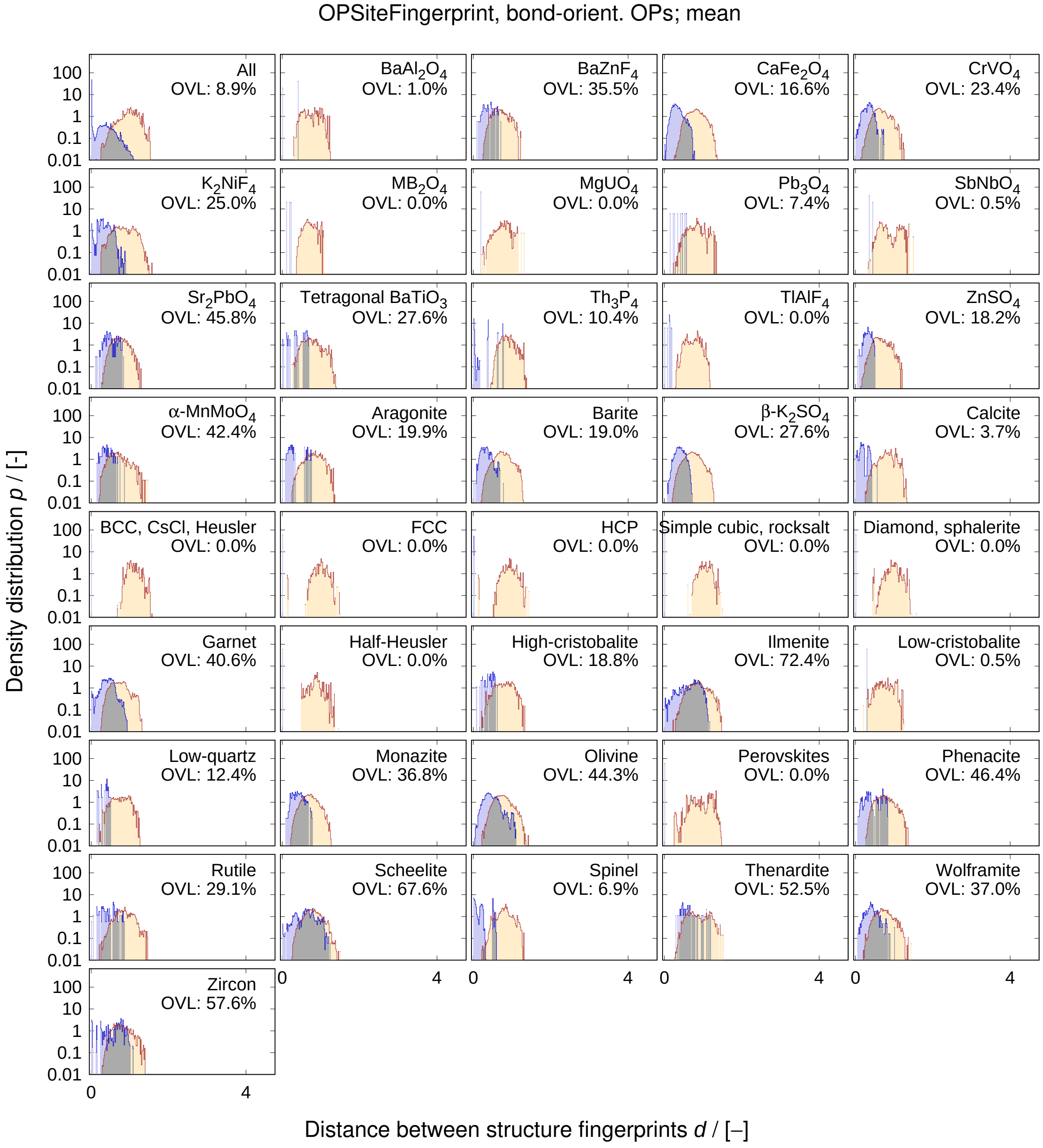

Fig. 99 Additional structure group (dis)similarity results.

OPSiteFingerprint, bond-orient. OPs; mean, max.

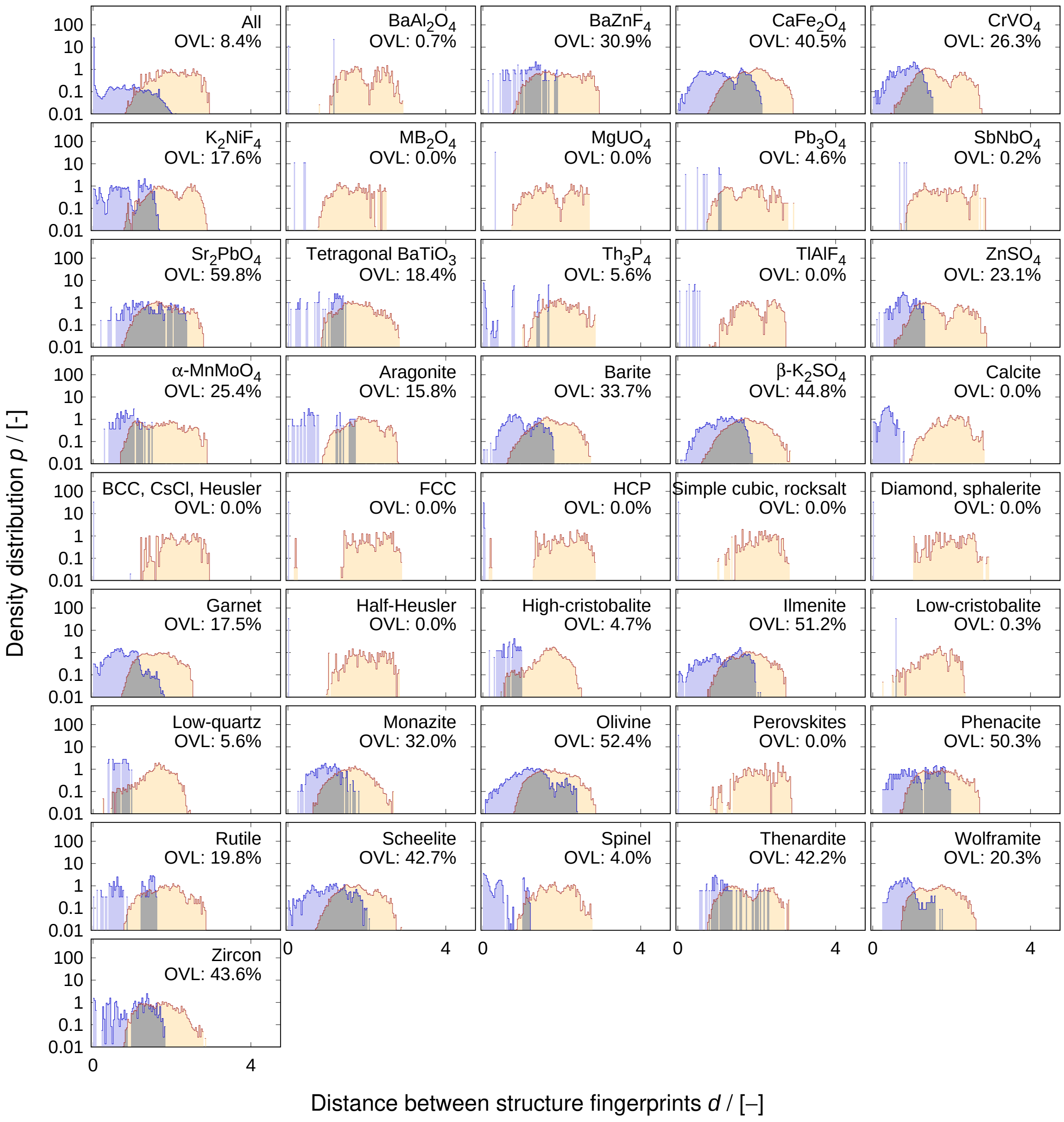

Fig. 100 Additional structure group (dis)similarity results.

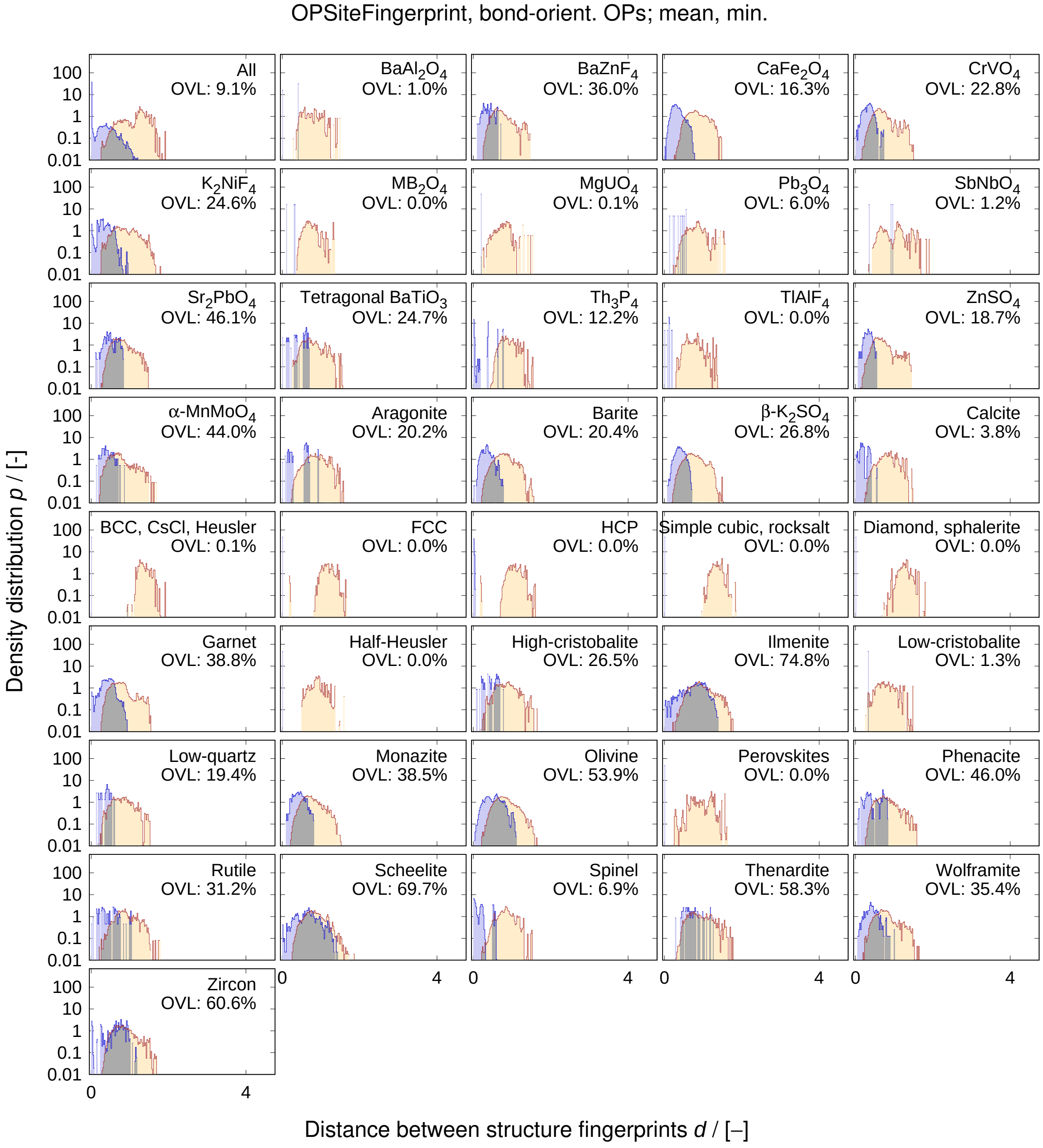

Fig. 101 Additional structure group (dis)similarity results.

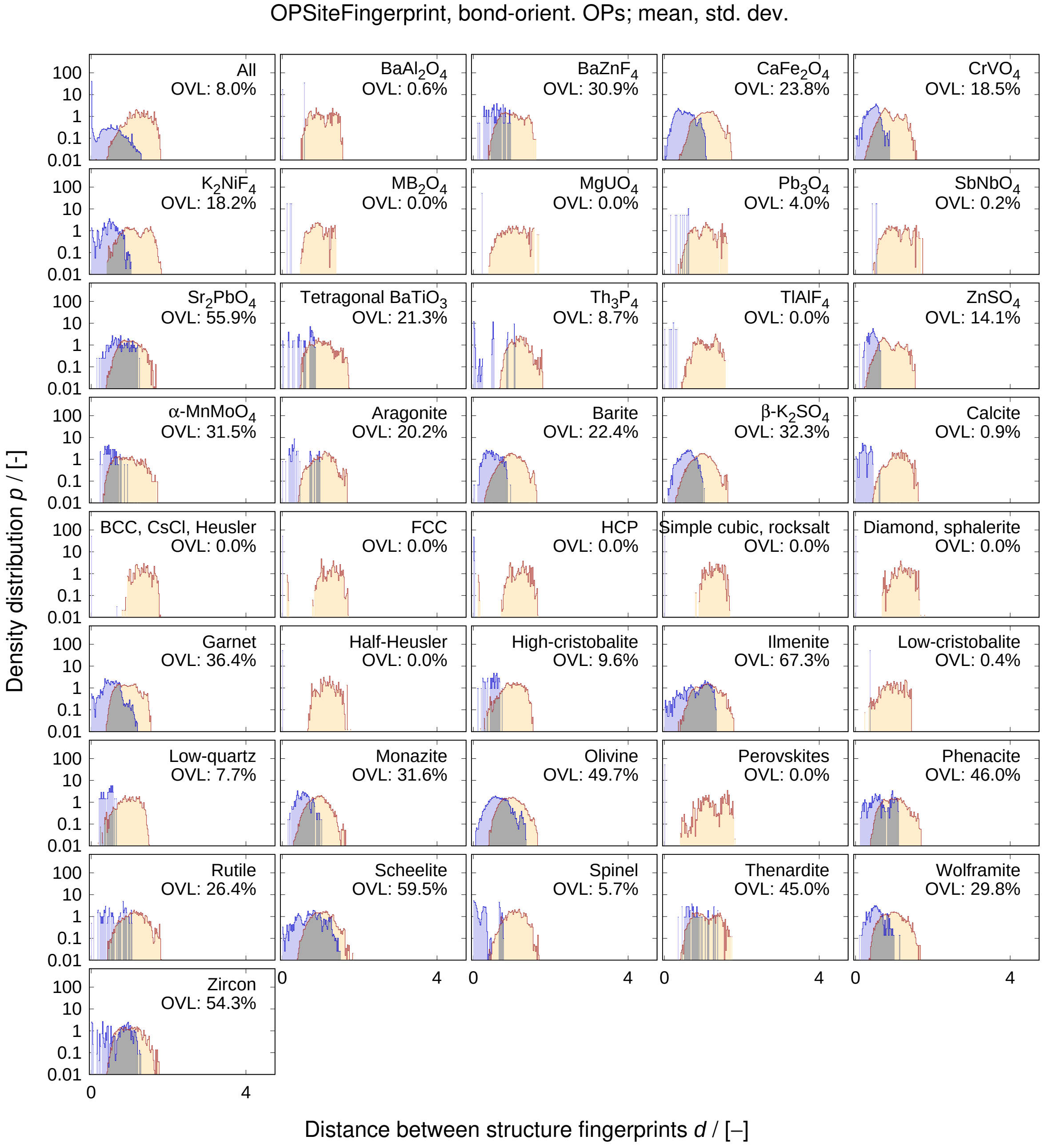

Fig. 102 Additional structure group (dis)similarity results.

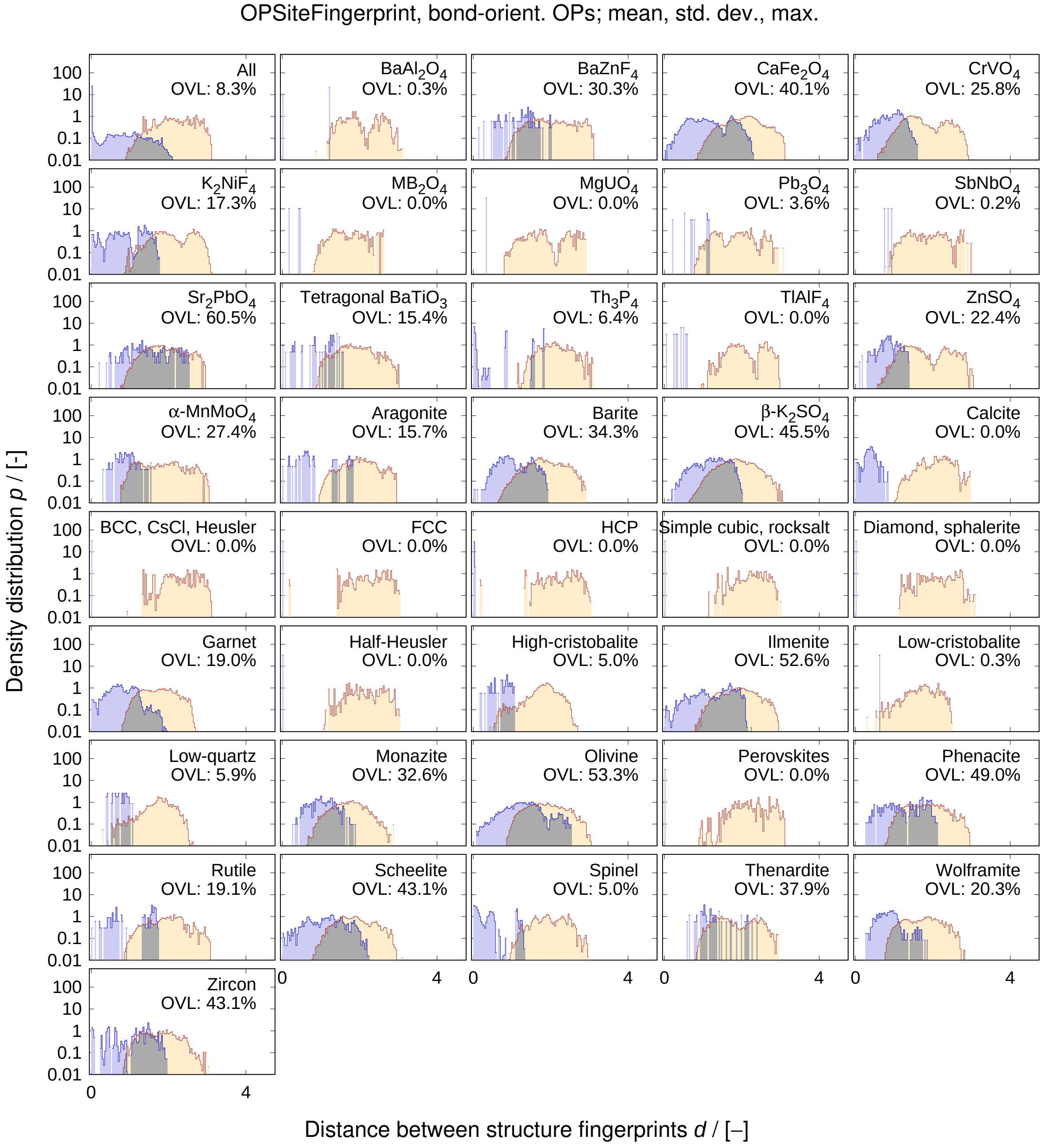

Fig. 103 Additional structure group (dis)similarity results.

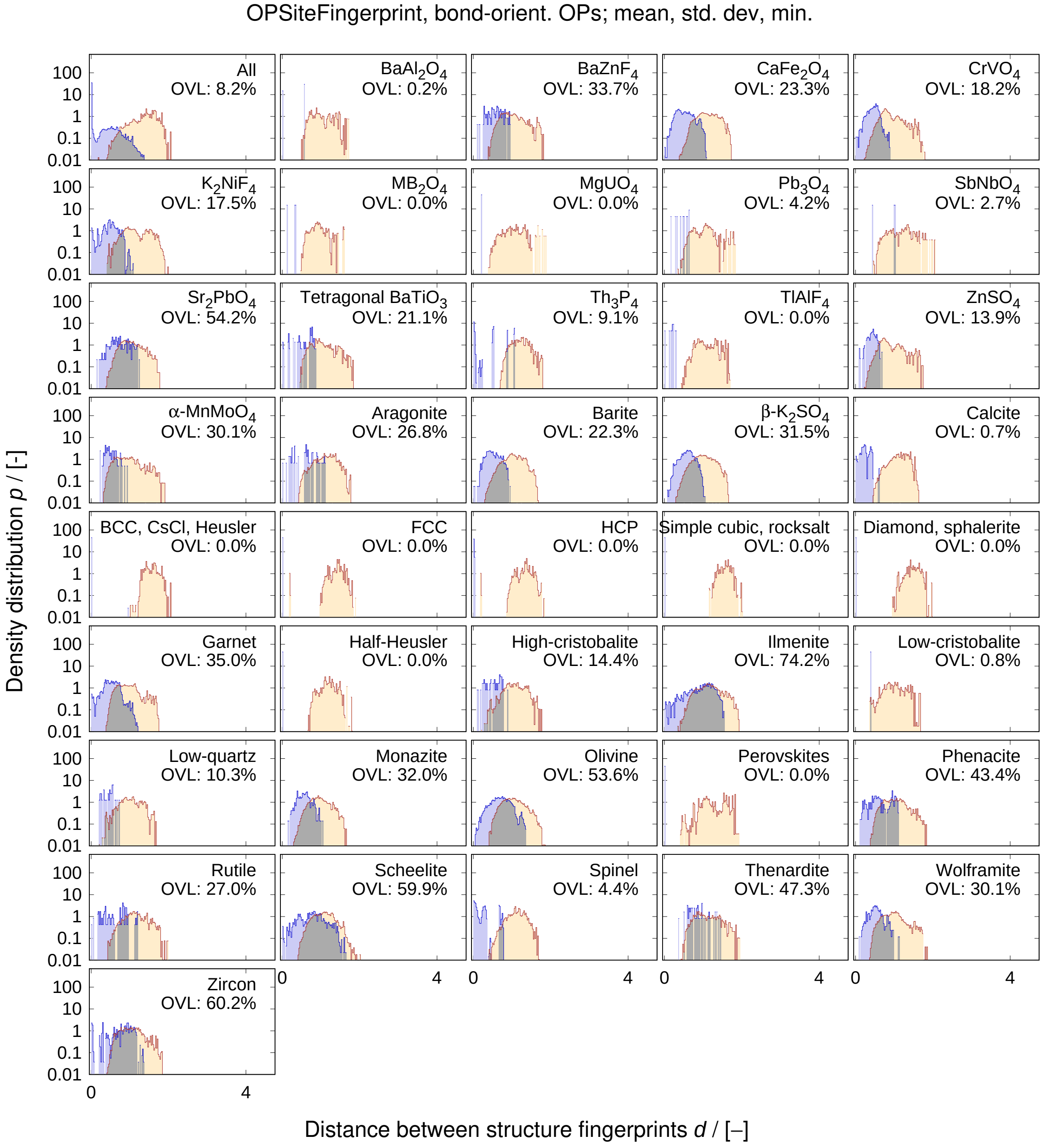

Fig. 104 Additional structure group (dis)similarity results.

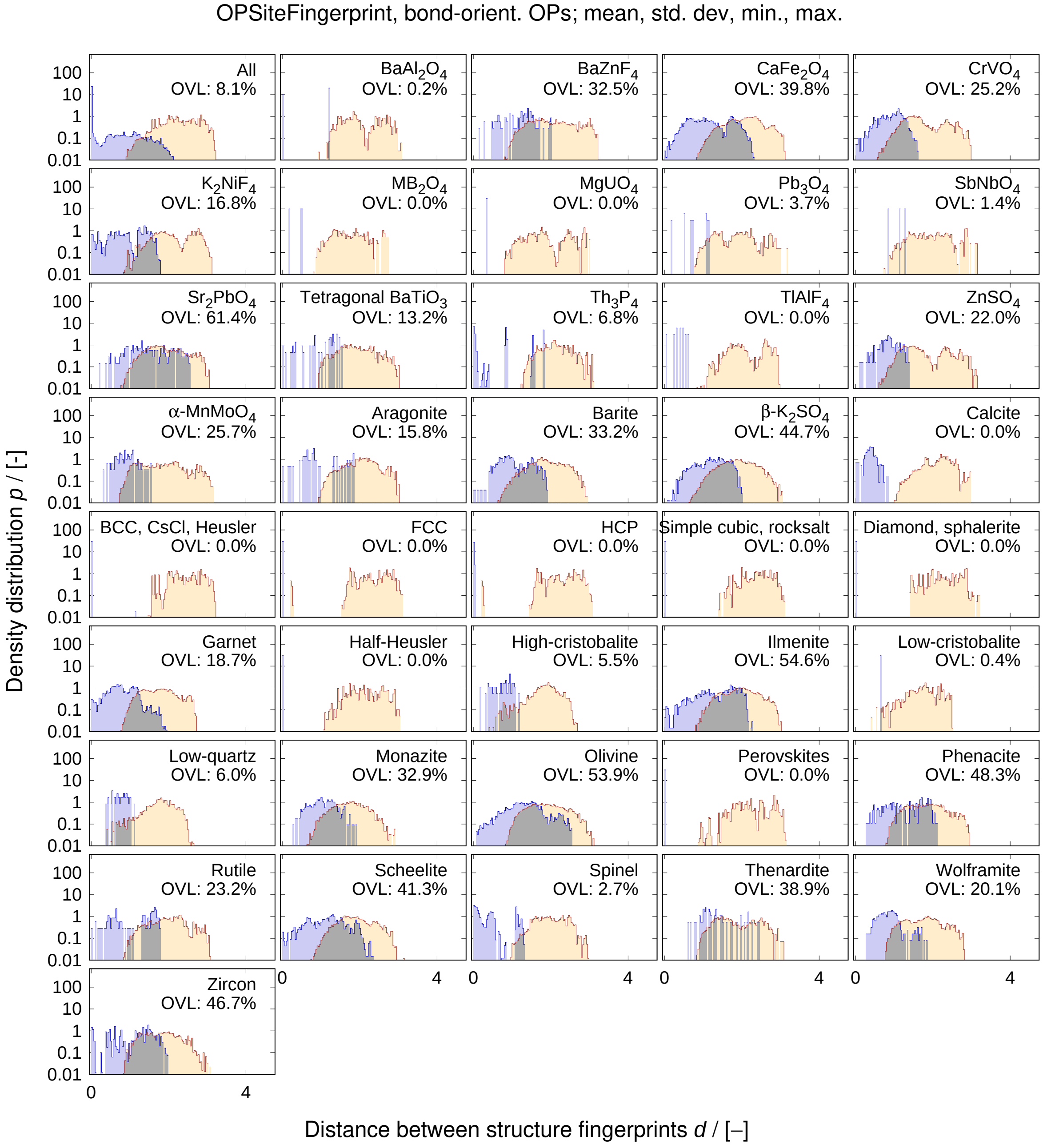

Fig. 105 Additional structure group (dis)similarity results.

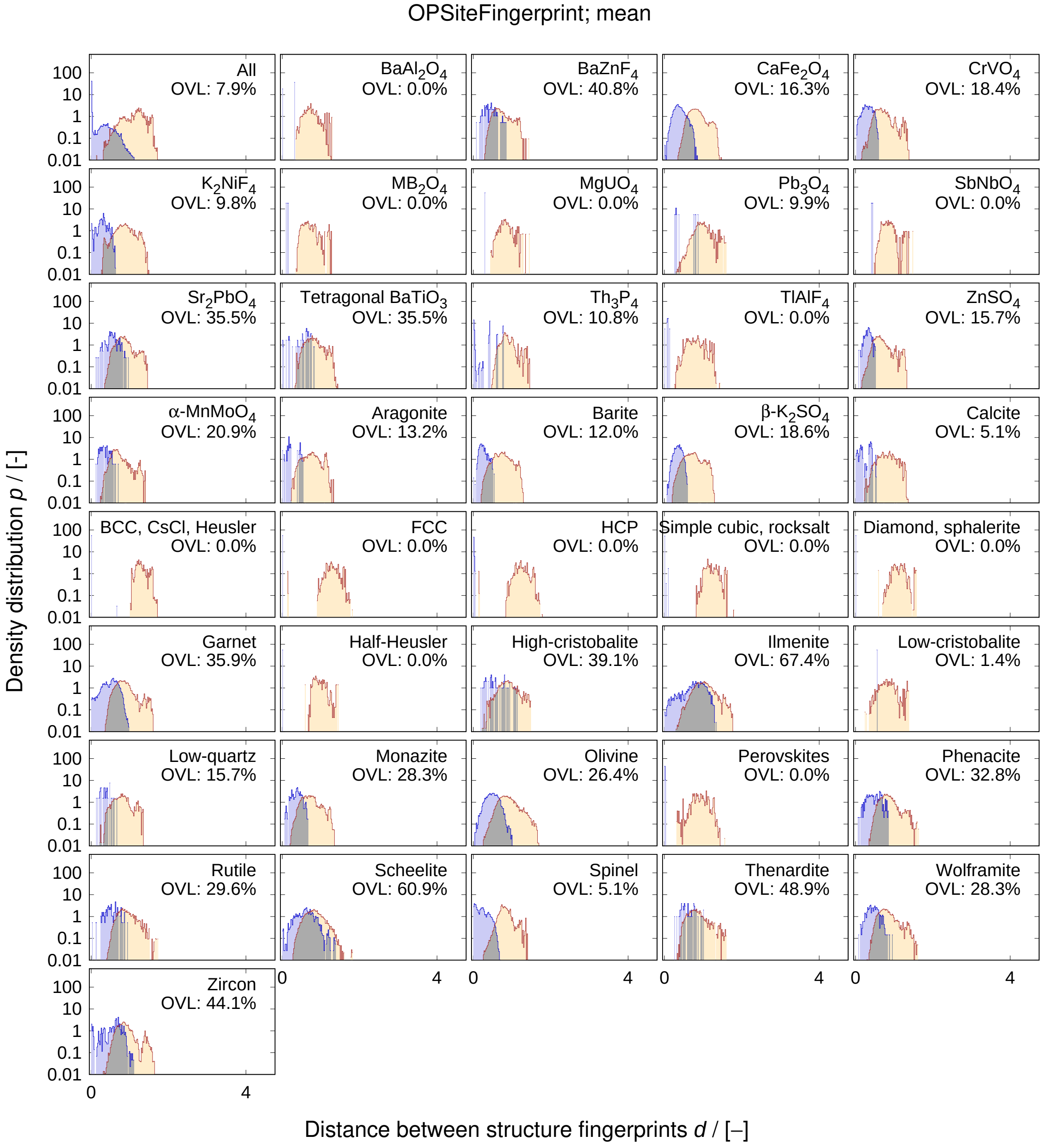

Fig. 106 Additional structure group (dis)similarity results.

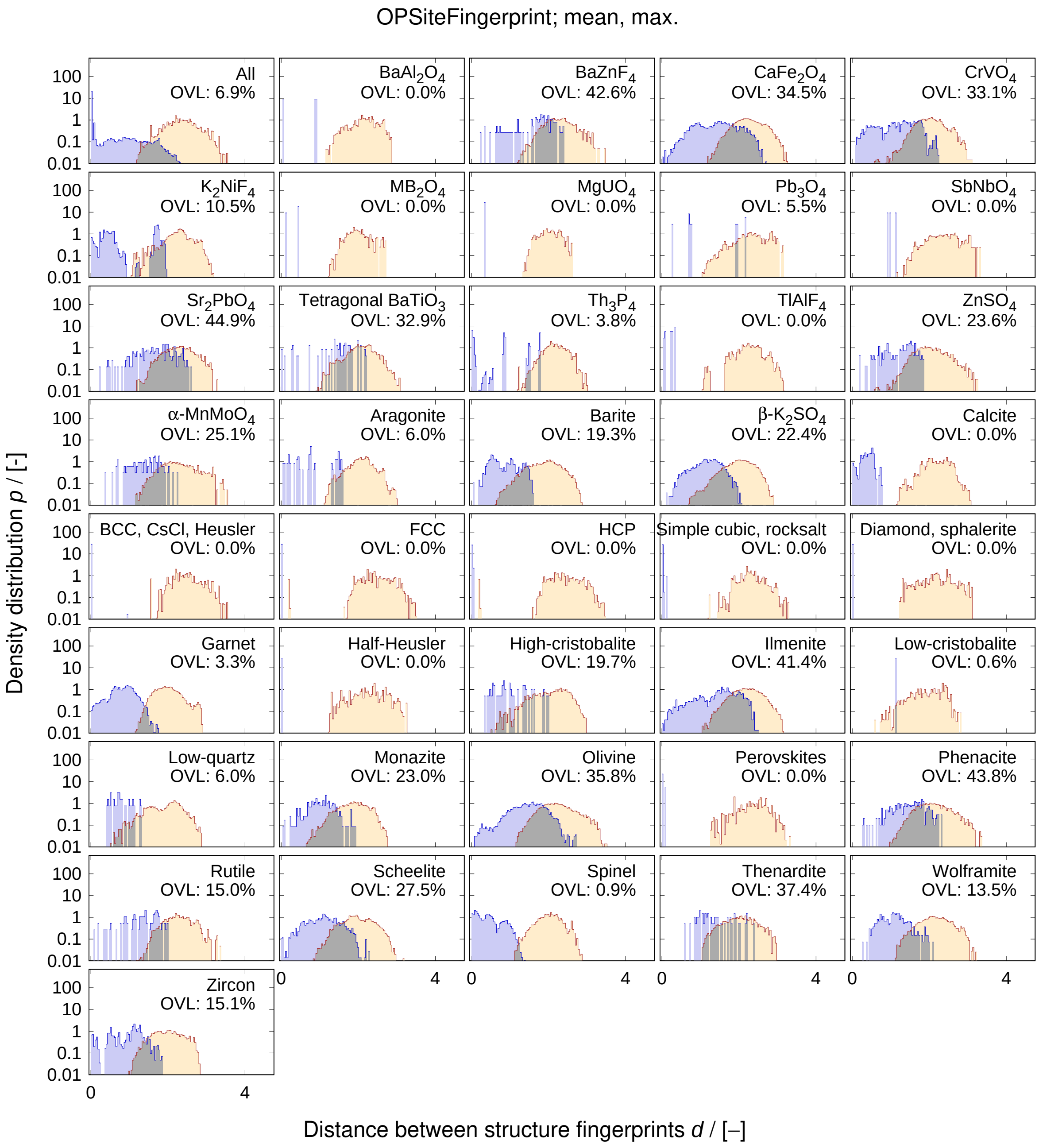

Fig. 107 Additional structure group (dis)similarity results.

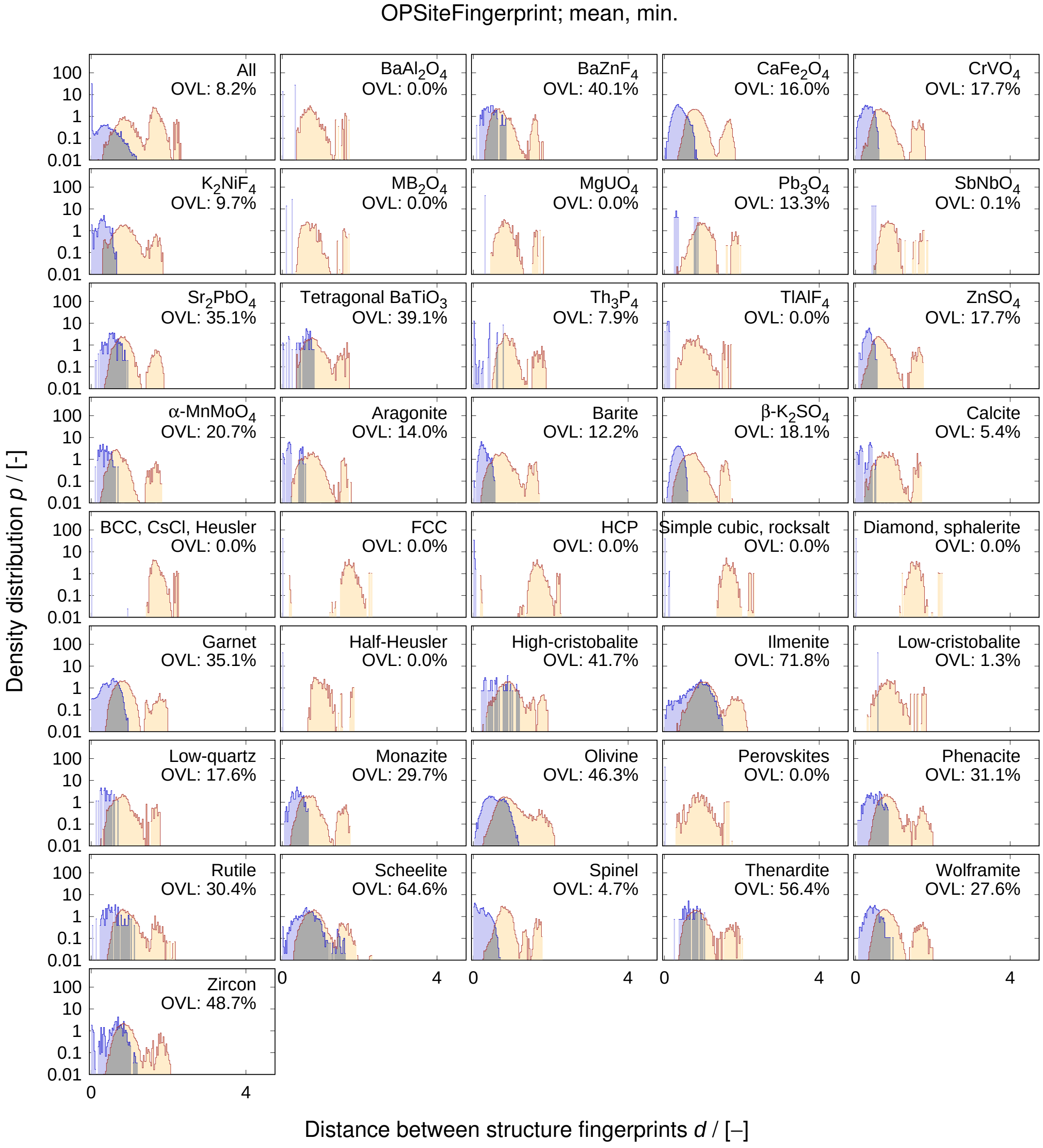

Fig. 108 Additional structure group (dis)similarity results.

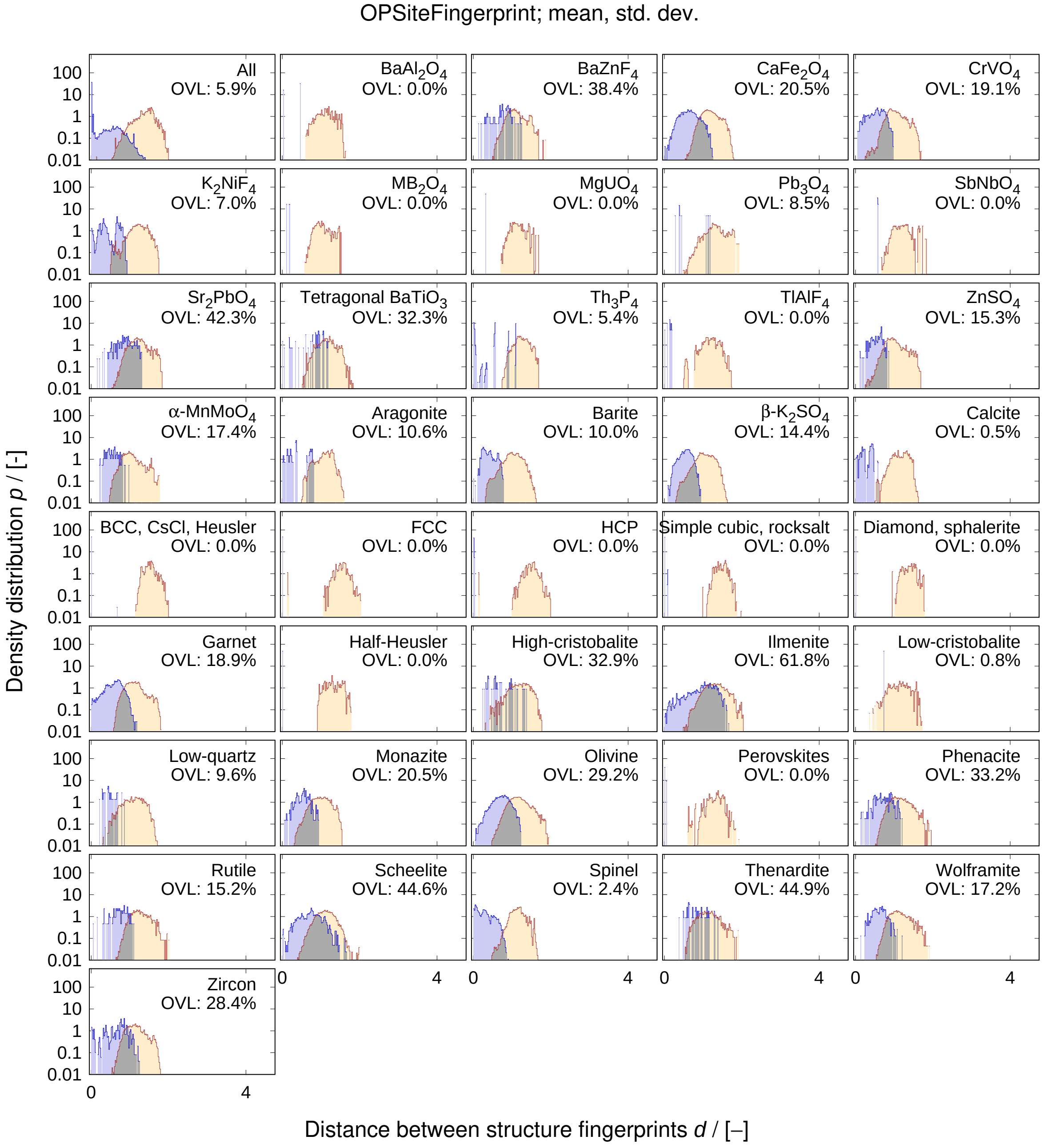

Fig. 109 Additional structure group (dis)similarity results.

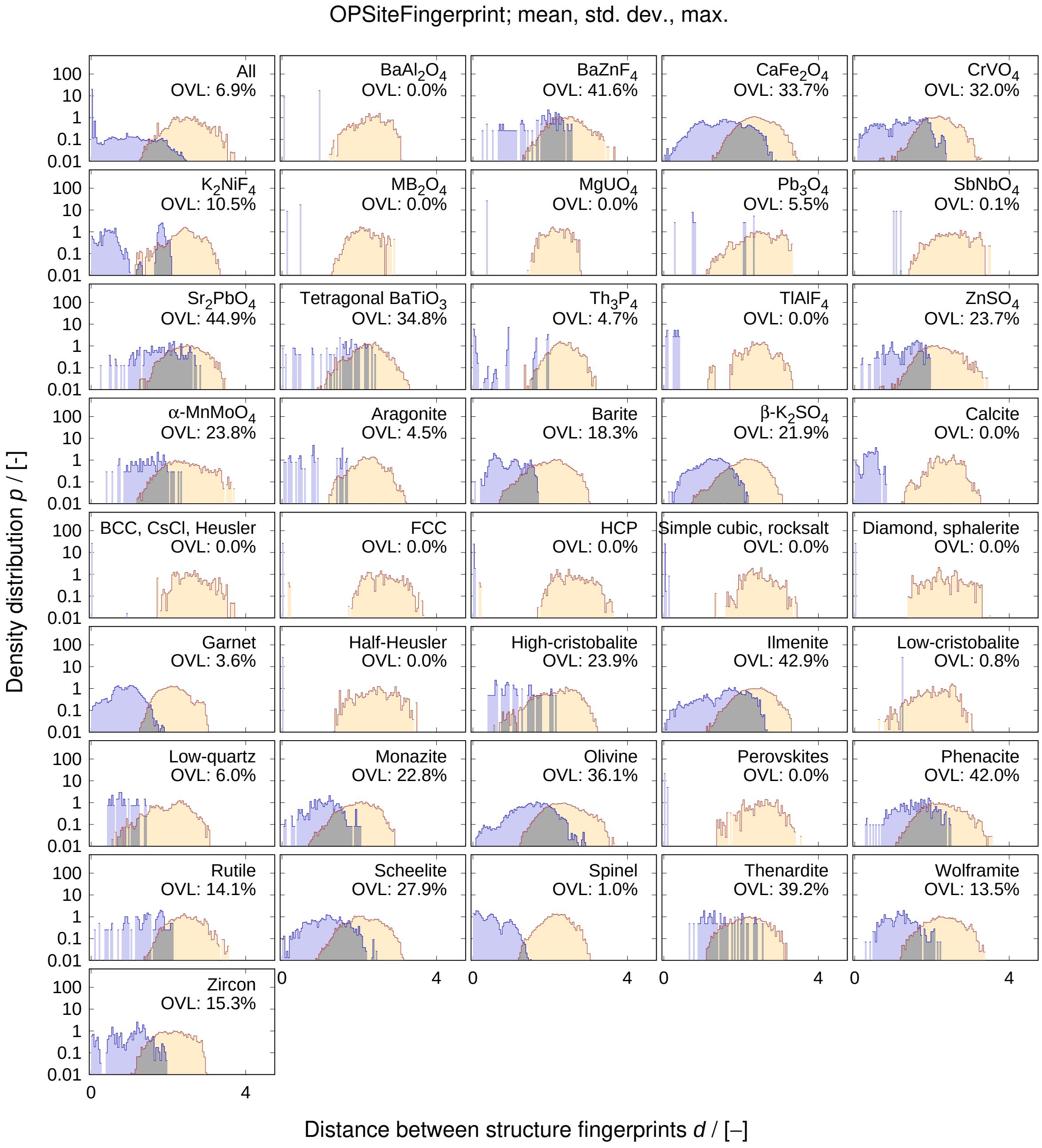

Fig. 110 Additional structure group (dis)similarity results.

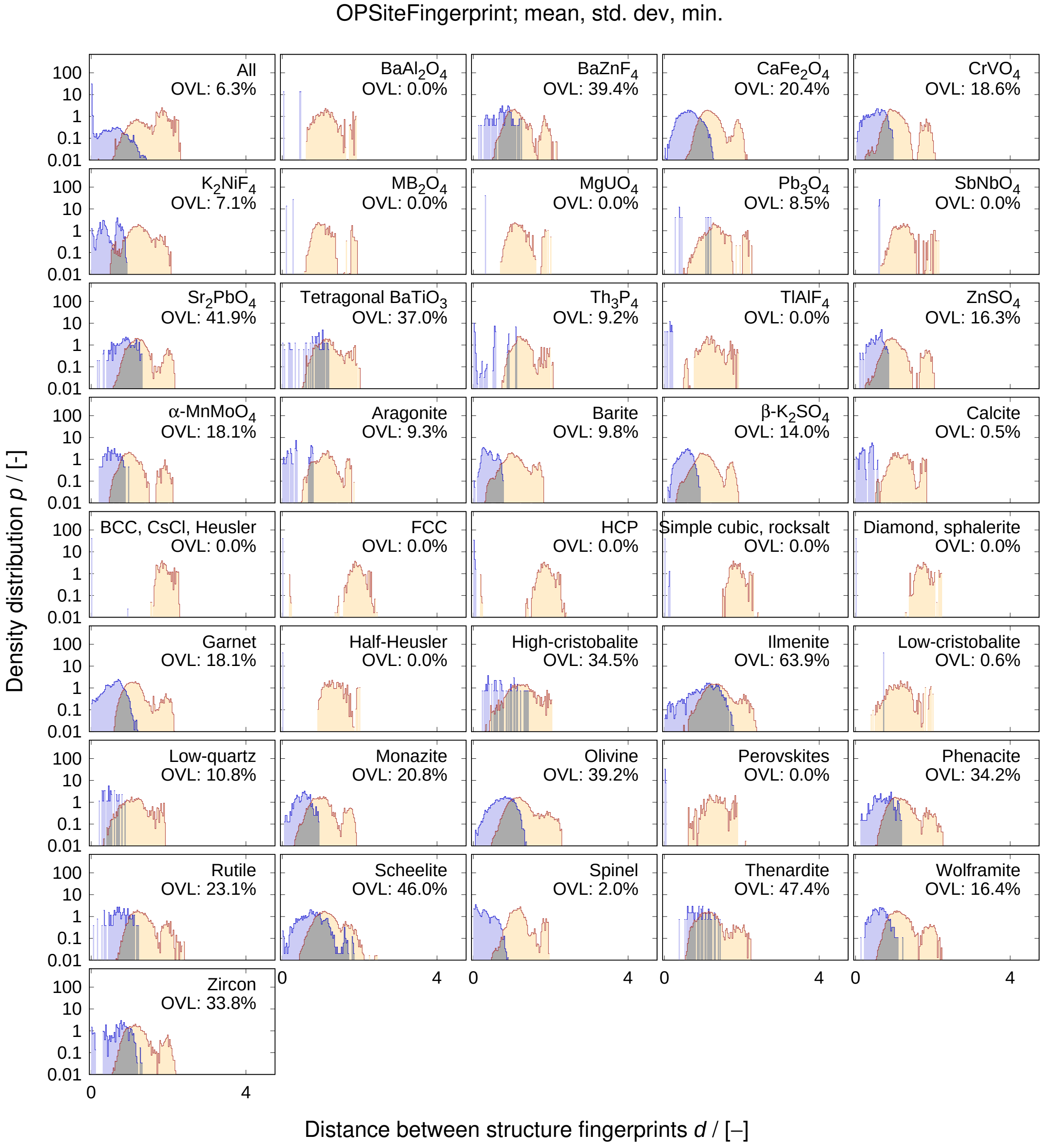

Fig. 111 Additional structure group (dis)similarity results.

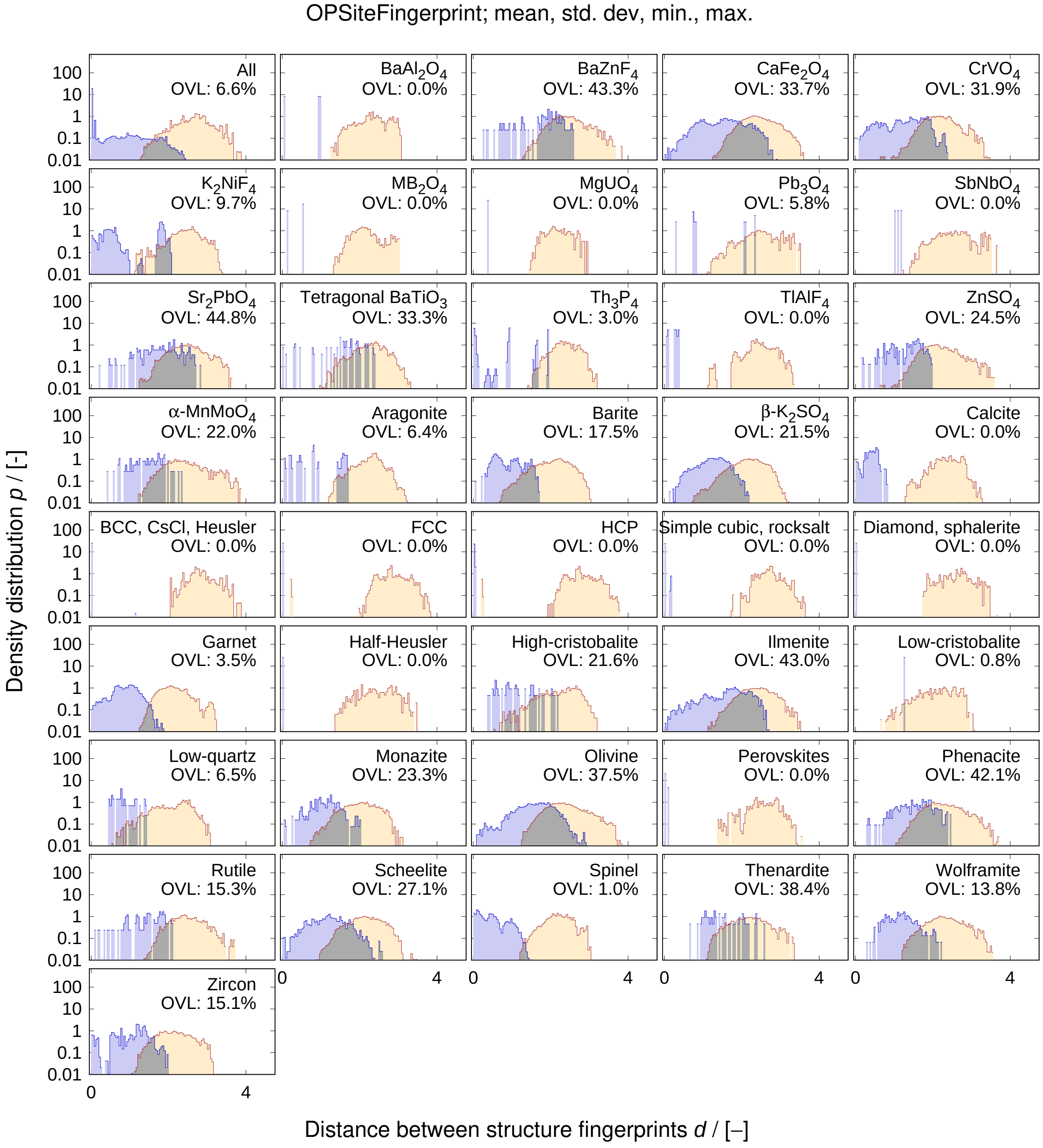

Fig. 112 Additional structure group (dis)similarity results.

2 List of symbols

Symbol	Description	Units
d	distance (d_{ij}: between atoms i and j)	m
$\tilde{d}$	dimensionless distance (i.e., d/l)	–
f_d	distance variation factor	–
i	index (or, degree) variable	–
j	index variable	–
k	index variable	–
k_{MC}	Monte Carlo fit parameter	–
l	index variable	–
m	index/type variable	–
N_{dim}	dimensionality	–
N_{nn}	number of near(est) neighbors	–
$\mathbf{p}$	point in 3-dimensional Euclidean space	m
P	(cumulative) probability	–
p	probability (density)	–
q_i	order parameter of type i (if i is an integer, then q is the bond orientational OP of degree i)	–
r	radius	m
$\mathbf{r}_{ij}$	(directional) vector from point $\mathbf{p}_i$ to point $\mathbf{p}_j$	m
s	similarity measure	–
$\mathbf{v}$	(site or structure) fingerprint	–
$w_{\mathrm{CN}=i}$	coordination likelihood for CN being i	–
x	some property (e.g., order parameter)	–
X	a generalized variable	–
$\bar{x}$	mean value of a set of some property x	–
α	angle	rad
δ	(fractional) tolerance	–
δ	mean absolute error	–
θ_{ijk}	(polar) angle between $\mathbf{r}_{ij}$ and $\mathbf{r}_{ik}$	–
φ_{ijkl}	(azimuth) angle	–
π	3.141592653589793	–
σ	empirical standard deviation or Gaussian width of a normal distribution	–

Superscript	Description
*	indicates a modified quantity
OP	reference to local structure order parameters
target	indicates a target number

Subscript	Description
bcc	reference to the bcc structure or a bcc-like coordination motif
bent	reference to a bent (i.e., non-colinear) coordination motif
cos	reference to the cosine function
cuboct	cuboctahedral coordination motif
dist	reference to a distance (similarity measure)
dot	reference to a dot product
EM	reference to Einstein crystal or molecule-like behavior
hex_bipyr	reference to a hexagonal bipyramidal coordination motif

To be continued on next page.

Subscript	Description
hex_plan	reference to a hexagonal planar coordination motif
hex_pyr	reference to a hexagonal pyramidal coordination motif
lin	reference to a colinear coordination motif
min	reference to a minimum value
new	reference to a new (i.e., trial) parameter state
oct	reference to an octahedral coordination motif
old	reference to an old (i.e., current) parameter state
pent_bipyr	reference to a pentagonal bipyramidal coordination motif
pent_plan	reference to a pentagonal planar coordination motif
pent_pyr	reference to a pentagonal pyramidal coordination motif
see_saw	reference to a (conventional) see-saw-shaped coordination motif
see_saw_rect	reference to a see-saw-shaped coordination motif with 90° instead of 120°
sgl_bd	reference to a single bond (motif)
site	reference to a site or coordination environment around a central site
sq	reference to a square non-coplanar coordination motif
sq_plan	reference to a square planar coordination motif
sq_pyr	reference to a square pyramidal coordination motif
struct	reference to a crystal structure
T	reference to a T-shape coordination motif
tet	reference to a tetrahedral coordination motif
tri_bipyr	reference to a trigonal bipyramidal coordination motif
tri_plan	reference to a trigonal planar coordination motif
tri_pyr	reference to a trigonal pyramidal coordination motif
trig_prism	reference to a trigonal prismatic coordination motif

Acrynom or Abbreviation	Description
AFLOW	Automatic FLOW for Materials Discovery
BCC	body centered cubic
CCDC	Cambridge Crystallographic Data Centre
CE	ChemEnv (fingerprint)
CEP	Harvard Clean Energy Project
CN	coordination number
CNN	“CrystalNN”-based neighbor finding
COD	Crystallography Open Database
CSM	continuous symmetry measure
FCC	face centered cubic
HCP	hexagonal closed packed
ICSD	Inorganic Crystal Structure Database
LoStOP	local structure order parameters
MAE	mean absolute error
MC	Monte Carlo
MDNF	“minimum distance” neighbor finding
MP	Materials Project
NOMAD	Novel Materials Discovery Laboratory
OPS	order parameter site (fingerprint)
OQMD	Open Quantum Materials Database
OVL	overlapping coefficient
PCD	Pearson’s Crystal Data

To be continued on next page.

Acrynom or Abbreviation	Description
PCOD	Predicted Crystallography Open Database
SML	statistical and machine learning
VNF	Voronoi decomposition-based neighbor finding
